# *Cryptophyllium*, the hidden leaf insects – descriptions of a new leaf insect genus and thirteen species from the former celebicum species group (Phasmatodea, Phylliidae)

**DOI:** 10.3897/zookeys.1018.61033

**Published:** 2021-02-18

**Authors:** Royce T. Cumming, Sarah Bank, Joachim Bresseel, Jérôme Constant, Stéphane Le Tirant, Zhiwei Dong, Gontran Sonet, Sven Bradler

**Affiliations:** 1 Montreal Insectarium, 4581 rue Sherbrooke est, Montréal, Québec, Canada, H1X 2B2 Montreal Insectarium Montréal Canada; 2 Richard Gilder Graduate School, American Museum of Natural History, New York, NY 10024, USA American Museum of Natural History New York United States of America; 3 Biology, Graduate Center, City University of New York, NY, USA City University of New York New York United States of America; 4 Department of Animal Evolution and Biodiversity, Johann-Friedrich-Blumenbach Institute for Zoology and Anthropology, University of Göttingen, Untere Karspüle 2, 37073, Göttingen, Germany University of Göttingen Göttingen Germany; 5 Royal Belgian Institute of Natural Sciences, O.D. Taxonomy and Phylogeny and JEMU, rue Vautier 29, B-1000, Brussels, Belgium Royal Belgian Institute of Natural Sciences Brussels Belgium; 6 State Key Laboratory of Genetic Resources and Evolution, Kunming Institute of Zoology, Chinese Academy of Sciences, Kunming, Yunnan, 650223, China Institute of Zoology, Chinese Academy of Sciences Kunming China

**Keywords:** Description, Greek Mythology, new species, Phasmida, Phylliini, *Phyllium*, Southeast Asia, Vietnam

## Abstract

While the leaf insects (Phylliidae) are a well-supported group within Phasmatodea, the genus *Phyllium* Illiger, 1798 has repeatedly been recovered as paraphyletic. Here, the Phyllium (Phyllium) celebicum species group is reviewed and its distinctiveness from the remaining Phylliini genera and subgenera in a phylogenetic context based on morphological review and a phylogenetic analysis of three genes (nuclear gene 28S and mitochondrial genes COI and 16S) from most known and multiple undescribed species is shown. A new genus, *Cryptophyllium***gen. nov.**, is erected to partially accommodate the former members of the *celebicum* species group. Two species, *Phylliumericoriai*Hennemann et al., 2009 and *Phylliumbonifacioi* Lit & Eusebio, 2014 morphologically and molecularly do not fall within this clade and are therefore left within Phyllium (Phyllium). The transfer of the remaining *celebicum* group members from *Phyllium* Illiger, 1798 to this new genus creates the following new combinations; *Cryptophylliumathanysus* (Westwood, 1859), **comb. nov.**; *Cryptophylliumcelebicum* (de Haan, 1842), **comb. nov.**; *Cryptophylliumchrisangi* (Seow-Choen, 2017), **comb. nov.**; *Cryptophylliumdrunganum* (Yang, 1995), **comb. nov.**; *Cryptophylliumoyae* (Cumming & Le Tirant, 2020), **comb. nov.**; *Cryptophylliumparum* (Liu, 1993), **comb. nov.**; *Cryptophylliumrarum* (Liu, 1993), **comb. nov.**; *Cryptophylliumtibetense* (Liu, 1993), **comb. nov.**; *Cryptophylliumwestwoodii* (Wood-Mason, 1875), **comb. nov.**; *Cryptophylliumyapicum* (Cumming & Teemsma, 2018), **comb. nov.**; and *Cryptophylliumyunnanense* (Liu, 1993), **comb. nov.**

The review of specimens belonging to this clade also revealed 13 undescribed species, which are described within as: *Cryptophylliumanimatum***gen. et sp. nov.** from Vietnam: Quang Nam Province; *Cryptophylliumbankoi***gen. et sp. nov.** from Vietnam: Quang Ngai, Thua Thien Hue, Da Nang, Gia Lai, Quang Nam, and Dak Nong Provinces; *Cryptophylliumbollensi***gen. et sp. nov.** from Vietnam: Ninh Thuan Province; *Cryptophylliumdaparo***gen. et sp. nov.** from China: Yunnan Province; *Cryptophylliumechidna***gen. et sp. nov.** from Indonesia: Wangi-wangi Island; *Cryptophylliumfaulkneri***gen. et sp. nov.** from Vietnam: Quang Ngai and Lam Dong Provinces; *Cryptophylliumicarus***gen. et sp. nov.** from Vietnam: Lam Dong and Dak Lak Provinces; *Cryptophylliumkhmer***gen. et sp. nov.** from Cambodia: Koh Kong and Siem Reap Provinces; *Cryptophylliumlimogesi***gen. et sp. nov.** from Vietnam: Lam Dong, Dak Lak, and Dak Nong Provinces; *Cryptophylliumliyananae***gen. et sp. nov.** from China: Guangxi Province; *Cryptophylliumnuichuaense***gen. et sp. nov.** from Vietnam: Ninh Thuan Province; *Cryptophylliumphami***gen. et sp. nov.** from Vietnam: Dong Nai and Ninh Thuan Provinces; and *Cryptophylliumwennae***gen. et sp. nov.** from China: Yunnan Province. All newly described species are morphologically described, illustrated, and molecularly compared to congenerics.

With the molecular results revealing cryptic taxa, it was found necessary for *Cryptophylliumwestwoodii* (Wood-Mason, 1875), **comb. nov.** to have a neotype specimen designated to allow accurate differentiation from congenerics. To conclude, male and female dichotomous keys to species for the *Cryptophyllium***gen. nov.** are presented.

## Introduction

**Figure 1.**
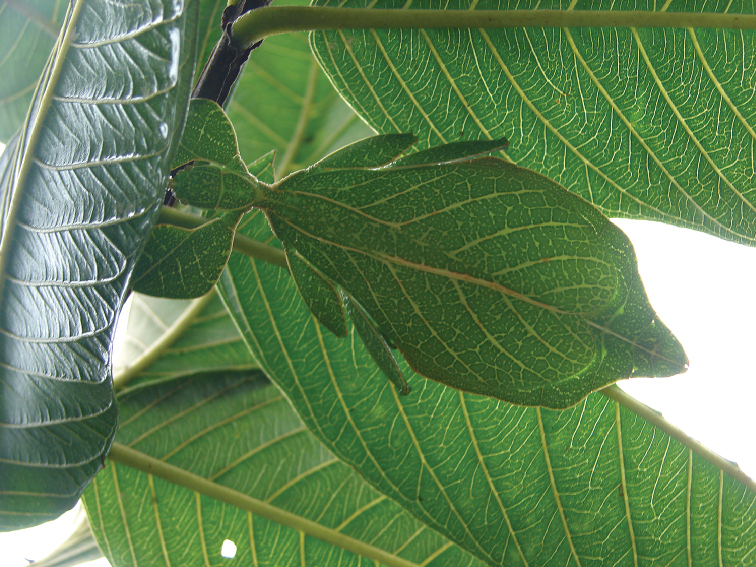
Paratype *Cryptophylliumphami* sp. nov. where it was found in Cat Tien N.P. (Vietnam) feeding on a Guava tree (*Psidiumguajava*) in July 2012 by Jérôme Constant (RBINS) and Joachim Bresseel (RBINS). The photograph was taken without flash to show how they appear naturally from underneath with the light passing through their thin bodies like the light passing through a leaf.

Phylliidae comprise the true leaf insects, a subordinated clade within the plant mimicking lineage of Phasmatodea (Bradler and Buckley 2018; Simon et al. 2019). While most phasmatodeans exhibit an elongated and slender body form to camouflage perfectly in the foliage, among branches or on bark (Bedford 1978), leaf insects mastered the imitation of angiosperm leaves (Fig. 1).

Of the five phylliid genera, the majority of species are attributed to *Phyllium*, which comprises 65 of the 89 currently described and valid Phylliidae species (Brock et al. 2020). In previous studies on Phasmatodea, the Phylliidae were always recovered as monophyletic whereas *Phyllium* itself appeared to be paraphyletic (Buckley et al. 2009; Bradler et al. 2015; Robertson et al. 2018). Thus, the *Phyllium* are in need of a thorough phylogenetic analysis. Taxonomically, *Phyllium* is further divided into four subgenera: *Phyllium*, *Pulchriphyllium*, and the recently described *Comptaphyllium* and *Walaphyllium* (Cumming et al. 2019; Cumming et al. 2020b). For the two traditional subgenera *Phyllium* and *Pulchriphyllium*, an intra-generic systematization had been proposed in order to facilitate differentiation in a taxonomical context (Hennemann et al. 2009). The subdivision of the most diverse subgenus Phyllium (Phyllium) into the *siccifolium* and *celebicum* species groups was mainly based on the presence of developed alae in females of the latter, a problematic character in a group with strong sexual dimorphism and several species only known from single sexes (e.g., Liu 1993; Yang 1995).

One method that has allowed confident matching of opposite sexes is through captive cultures as was demonstrated for the *Nanophyllium* (described only from males) whose opposite sex was found to be already described in the *Phyllium* (Cumming et al. 2020c). Although laboratory rearing is substantial to ascertain the useful taxonomic trio of female, male, and egg morphology, and therefore an important factor for taxonomic classification, it has its limitations. With the aid of molecular data, in addition to matching up sexes and identifying new species, it is also possible to uncover potential cryptic species (Cumming et al. 2020a).

Since 2010, staff from the Royal Belgian Institute of Natural Sciences (RBINS), led by Jérôme Constant, have participated in expeditions to document the phasmid fauna of Vietnam and Cambodia in the framework of the Global Taxonomy Initiative (GTI) projects “A step further in the entomodiversity of Vietnam” (ten expeditions, 2010–2019) and “A step further in the entomodiversity of Cambodia” (three expeditions, 2016–2018) (Constant et al. 2018). The expeditions were jointly organized with the Institute of Ecology and Biological Resources (2010–2014) and the Vietnam National Museum of Nature (2015–2019), which are both part of the Vietnam Academy of Science and Technology, in Vietnam, and with the Royal University of Phnom Penh in Cambodia. For each expedition, the local institutions organized the collecting permits and official paperwork. GTI was created in the framework of the Convention on Biological Diversity to remove the ‘taxonomic impediment’ (www.cbd.int/gti). The goals of these GTI projects organized by RBINS and partners are to document and describe the biodiversity, to provide taxonomy capacity building (in the field and in the lab) for Vietnamese and Cambodian students, and to assist and advise about the management of insect collections of the participating institutions. Through the efforts of the many participants of these expeditions, many scientifically significant specimens from the *celebicum* species group were discovered and utilized herein.

**Figure 2.**
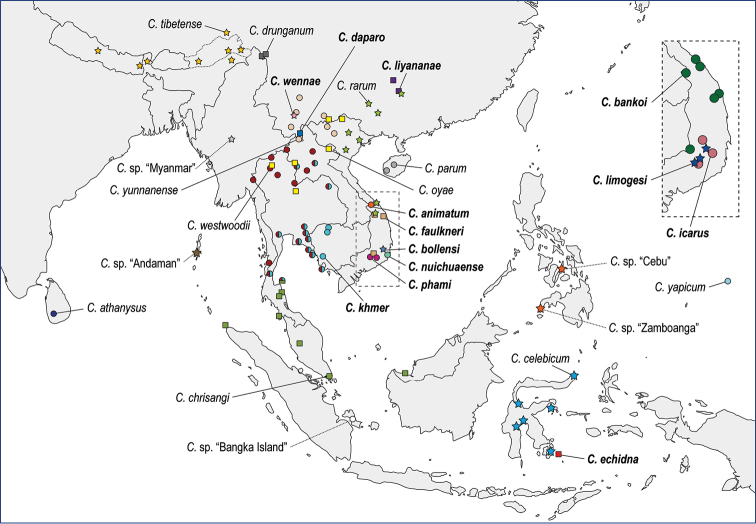
Distribution map for the 24 *Cryptophyllium* gen. nov. species presently known (with solid lines from their name pointing to the type locality) as well as additional *Cryptophyllium* gen. nov. species which we could not herein describe/differentiate (indicated by dashed lines). Note that the line for *Cryptophylliumwestwoodii* comb. nov. is pointing to the neotype locality and the type locality for *Cryptophylliumathanysus* comb. nov. is simply “Ceylon” therefore the line is pointing to the present-day localities we are aware of. Inset is of southern Vietnam showing the distributions of three additional species which could not fit within the main map. The colors in this map are noted to the left of the names within the phylogenetic tree in Fig. 4. Newly herein described species have names noted in bold. Note that with *Cryptophylliumkhmer* sp. nov. not easily distinguishable from *Cryptophylliumwestwoodii* comb. nov. from photos alone, only the locations for these two species where they were genetically sampled are solid colored, all observational images without genetic sampling have bicolored circles and could represent either of these species. Additionally, one symbol is split three ways for *Cryptophyllium* gen. nov. specimens from the Thai offshore islands of Ko Phangan and Ko Samui which could not be differentiated from *C.chrisangi* comb. nov., *C.westwoodii* comb. nov., and *C.khmer* sp. nov. from images alone. See Suppl. material 4 for a full list of the specimens/observations utilized to make the distribution map with deposition data for specimens and links to observational records>.

The *celebicum* species group currently contains 13 of the 42 species of the Phyllium (Phyllium) with a distribution spanning from Sri Lanka over southern China and mainland Southeast Asia to the Philippines, Sulawesi, and Micronesia (Fig. 2). Here, we aim to review the Phyllium (Phyllium) celebicum species group by extensive investigation of morphology as well as analysis of molecular data to test whether the taxon represents a monophyletic group, to confirm matching of sexes, to identify undescribed species, and reveal potential hidden (cryptic) diversity within the lineage. We also formally describe any new species for which species status appears warranted based on the obtained morphological and/or molecular data.

## Materials and methods

### Specimens

We examined and sequenced numerous specimens from both institutional and private collections for this review. Many of the specimens, which were sequenced for this work, are illustrated but not all specimens that were sequenced are included as figures. To ensure reproducibility for future investigators, images and institution accession numbers of additional specimens which were sequenced but not illustrated within the main body of this work can be found in Suppl. material 1. For each species discussed within we explicitly state the number of specimens reviewed, their collection data, and the collection they are contained within to allow the specimens utilized for this work to be located by other interested researchers to ensure reproducibility of our work. In multiple cases we were presented with photographs of individuals which we based morphological comparisons upon, in these instances many of these images were reproduced and referenced herein or their source is stated to allow the reviewed images to be located. Additionally, we utilized the extensive collection of high-quality images of captive reared specimens available publicly on Phasmatodea.com “The World of Stick Insects” (http://www.phasmatodea.com/taxonomy/term/1788) as well as the many type material images available on “Phasmida Species Files Online” (http://phasmida.speciesfile.org/HomePage/Phasmida/HomePage.aspx).

Measurements of specimens were made to the nearest 0.1 mm using digital calipers. Measurements for paratype specimens are given with a minimum to maximum range. Holotype and paratype specimens are deposited within several institutions which are explicitly listed within the type material information of the new species descriptions. The following collection acronyms are used. Specimen data listed within quotations is verbatim and is therefore in a variety of non-standard formats, but is presented as is to ensure traceability of specimens utilized.

**CAS**California Academy of Sciences, San Francisco, California, USA;

**BPBM**Bishop Museum, Honolulu, Hawaii, USA;

**IMQC** Insectarium de Montréal, Montréal, Québec, Canada;

**KIZ**Kunming Institute of Zoology, Yunnan, China;

**LKCNHM** Lee Kong Chian Natural History Museum, Singapore;

**MNHN**Muséum national d’Histoire naturelle, Paris, France;

**NHMUK**Natural History Museum United Kingdom, London, United Kingdom;

**NZSI**Zoological Survey of India, National Zoological Collection, Calcutta, India;

**OUMNH** University Museum of Natural History, Oxford, United Kingdom;

**RBINS**Royal Belgian Institute of Natural Sciences, Brussels, Belgium;

**RMNH**Naturalis Biodiversity Centre, Leiden, Netherlands;

**RUPP**Royal University of Phnom Penh, Phnom Penh, Cambodia;

**SDEI**Senckenberg Deutsches Entomologisches Institut, Müncheberg, Germany;

**UCR** University of California Riverside, California, USA;

**VNMN**Vietnam National Museum of Nature, Hanoi, Vietnam;

**GTI** Global Taxonomy Initiative (https://www.cbd.int/gti/);

**Coll FH** Private collection of Frank H. Hennemann, Germany;

**Coll MO** Private collection of Maxime Ortiz, France;

**Coll OC** Private collection of Oskar V. Conle, Duisburg, Germany;

**Coll RC** Private collection of Royce T. Cumming, California, USA;

**Coll SLT** Private collection of Stéphane Le Tirant, Québec, Canada;

**Coll TB** Private collection of Thies Büscher, Kiel, Germany;

**Coll ZD** Private collection of Zhiwei Dong, Yunnan, China.

### Photography

Photographs of specimens deposited within the IMQC collection were taken by René Limoges using a Nikon D850 DSLR camera (Nikon Corporation, Tokyo, Japan) with Nikon Micro-Nikkor 200mm f/4 lens on Manfrotto 454 micrometric positioning sliding plate (Manfrotto, Casolla, Italy). Lighting was provided by two Nikon SB-25 flash units with a Cameron Digital diffusion photo box (Henry’s, Vancouver, Canada). Adobe Photoshop Elements 13 (Adobe Inc., San Jose, USA) was used as post-processing software.

Photographs of specimens within the RBINS collection were taken by Jérôme Constant (RBINS). For each specimen a number of photographs were taken with a Canon 700D camera (Canon Inc., Ota City, Tokyo, Japan) equipped with a Sigma 50 mm Macro lens (Sigma Corporation, Kawasaki, Japan) for adults, or with a Leica EZ4W stereomicroscope (Leica Microsystems Ltd., Wetzlar, Germany) with integrated camera for eggs and vomers, and stacked with CombineZ software (https://combinezp.software.informer.com) and optimized with Adobe Photoshop software.

Additional photographs used which are not from these two above institutions are explicitly listed within the figure captions with citation to the photographers.

### Literature reviewed

All original species descriptions for relevant species were reviewed, and in several cases when not originally published in English the works were translated. To translate the relevant works of Liu (1993), Yang (1995), and Sorpongpaisal and Thanasinchayakul (2006), we used the most recent version of a phone-based application “Google Translate’’ (offered by Google LLC: version 6.2.0.RC07.268294262).

### Rules of zoological nomenclature

We here follow the International Code of Zoological Nomenclature (ICZN 1999) as a means of describing new phylliid taxa and designating the neotype *Cryptophylliumwestwoodii* (Wood-Mason, 1875), comb. nov.

### Molecular laboratory work and phylogenetic analysis

Tissue samples from 56 specimens of the Phyllium (Phyllium) celebicum species group as well as two outgroup samples (*Microphylliumhaskelli* and *Pseudomicrophylliumfaulkneri*) were obtained to generate molecular data for phylogenetic analysis. DNA extraction, PCR amplification of cytochrome oxidase subunit I (COI), 16S rRNA (16S) and parts of 28S rRNA (28S), and subsequent Sanger-sequencing was carried out following the protocol given by Cumming et al. (2020a). For two samples (RBINS13 and RBINS14), DNA was extracted using the NucleoSpin Tissue kit (MACHEREY-NAGEL, Düren, Germany) and eluted in 100 µl elution buffer and Sanger-sequencing was run on an ABI3130xl Genetic Analyzer (Applied Biosystems, Foster City, USA; Laboratory of Molecular Systematics of the RBINS, Brussels, Belgium). We decided that for most samples, especially possible duplicates, it is sufficient to sequence only one gene. DNA sequences were checked for quality and edited in Geneious Prime version 2020.0.4 (www.geneious.com) prior to deposition in GenBank (accession numbers MW161173–MW161229, MW165169–MW165218, see Suppl. material 5).

For the phylogenetic inference, we furthermore included sequence data of nine additional phylliid species and the three outgroup species published by Cumming et al. (2020a). Nucleotide sequences of a total of 70 specimens were aligned separately for each gene using the G-INS-I method of MAFFT version 7.453 (Katoh and Standley 2013). The alignments were manually trimmed at the beginning and end before concatenation with FASconCAT (Kück and Meusemann 2010). The phylogenetic inference was conducted in IQ-TREE version 2.1.1 (Minh et al. 2020) with a random starting tree and the best-fit substitution model selected by ModelFinder under AICc (GTR+F+R5, Kalyaanamoorthy et al. 2017). Node support was estimated using the Ultrafast Bootstrap approximation and 10,000 pseudo-replicates (UFBoot, Hoang et al. 2018). The resulting tree was visualized in FigTree v. 1.4.4 (https://github.com/rambaut/figtree) and rooted with Aschiphasmatinae (*Orthomeriakangi* Vallotto, Bresseel, Heitzmann & Gottardo, 2016), which has been repeatedly recovered as sister group to the remaining Euphasmatodea (= Neophasmatodea) (e.g., Robertson et al. 2018; Simon et al. 2019).

### Global Taxonomy Initiative (GTI) projects in Vietnam and Cambodia

**Figure 3.**
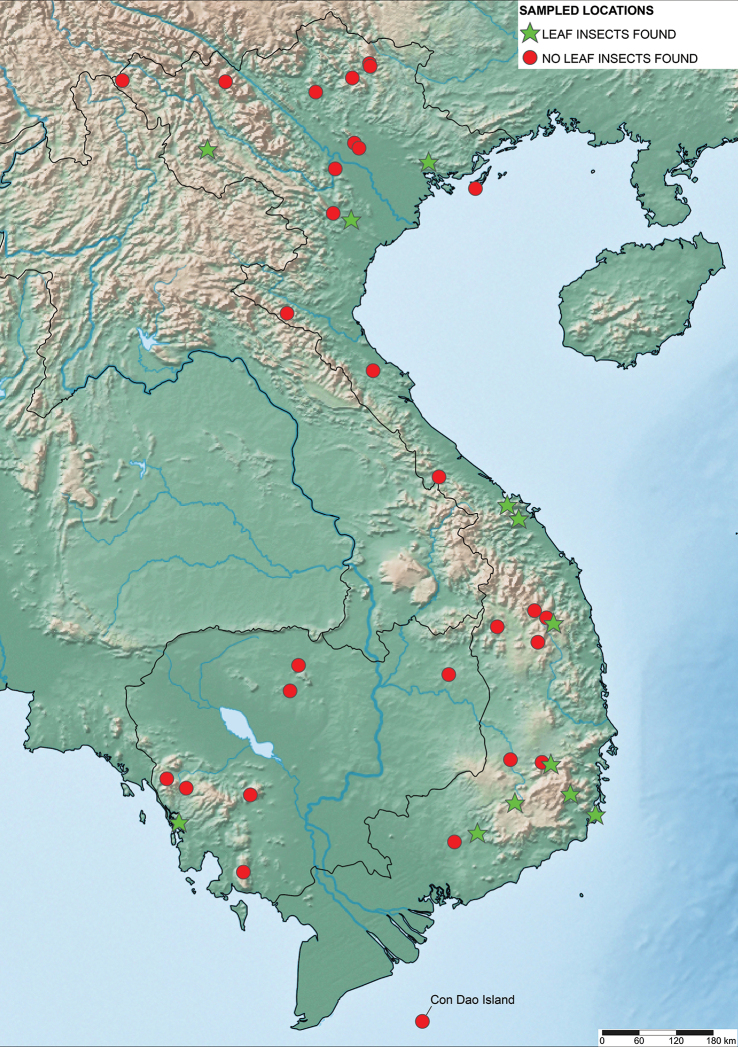
Map of Vietnam and Cambodia where GTI joint expeditions between the teams from RBINS, the Institute of Ecology and Biological Resources (Vietnam), the VNMN, and the RUPP in Cambodia have conducted fieldwork between 2010 and 2019. All sampled locations are marked, with the green stars indicating locations where leaf insect specimens were located and the red circles denoting sampled sites where leaf insects were not encountered.

Since 2010 a total of 206 days was spent in the field in Vietnam, with 32 locations sampled for stick/leaf insects leading to eleven collecting events (place and time where collecting occurred) involving leaf insects; in Cambodia 36 days of fieldwork in eight locations provided a single collecting event (Fig. 3). In total, eight species of leaf insect were collected, including seven yet to be described, and an additional young nymph that was unidentifiable, which did not survive. The collecting of stick and leaf insects is always better at night as these insects are mostly nocturnal. However, it has also been possible to find them during the day when keeping in mind important aspects of their biology, for example that guava trees (*Psidiumguajava* L., Myrtaceae) are an attractive food plant for the leaf insects which can be located by searching for their telltale feeding damage amongst the leaves (Fig. 1). Additionally, knowing the local name of the guava tree (cây ổi in Vietnamese; ដ ើមត្របែក “derm trobaek / derm trorbek” in Khmer) and of the leaf insects (bọ lá in Vietnamese; កណ្ដូបឈើ or កណ្ដូបស្លឹក “kandob chheu” or “kandob sloek” in Khmer) (H.T. Pham, P. Heang, and B. Doeurk pers. comm.) and interacting with local people proved a useful way to locate potential leaf insect habitats (Fig. 1). Being canopy dwellers, leaf insects are usually out of reach in the forest and have proven easier to find in anthropized habitats such as along roads (Fig. 32), on regrowth in recently burnt or logged areas (Figs 16, 48), or even on cultivated trees (mostly guava trees) near houses (Fig. 37). Adult male specimens, the only sex able to fly, were found attracted to lights a night. During the expeditions, males of *Cryptophylliumphami* gen. et sp. nov., *Cryptophylliumkhmer* gen. et sp. nov., *Cryptophylliumrarum* comb. nov., and *Cryptophylliumwestwoodii* comb. nov. were sampled using a light trap with mercury vapor lamps. One male of *Cryptophylliumrarum* comb. nov. was collected when it was attracted to the streetlights in Cuc Phuong N.P. Field experience has shown that it is important to check the surroundings of the light trap as it seems that males are attracted to the lights, but not necessarily likely to alight on the white sheet like other nocturnal insects. Due to the somewhat feeble nature of males’ flight, we expect that only males in the vicinity of the light trap are attracted, not flying from great distances to reach it.

Whenever possible the female specimens were kept alive for several days to allow them to lay eggs which provide supplemental morphological characters to differentiate species. Additionally, these eggs were shared with enthusiastic phasmid breeders in Belgium and Switzerland who were instrumental in documenting the nymphal stages and in obtaining important specimens such as males of species of which only the female was found in the wild, rearing additional specimens to study intraspecific variation, or when species were only collected as nymphs from the wild these breeders helped by rearing them to adulthood.

## Results

### Phylogenetic analysis

**Figure 4.**
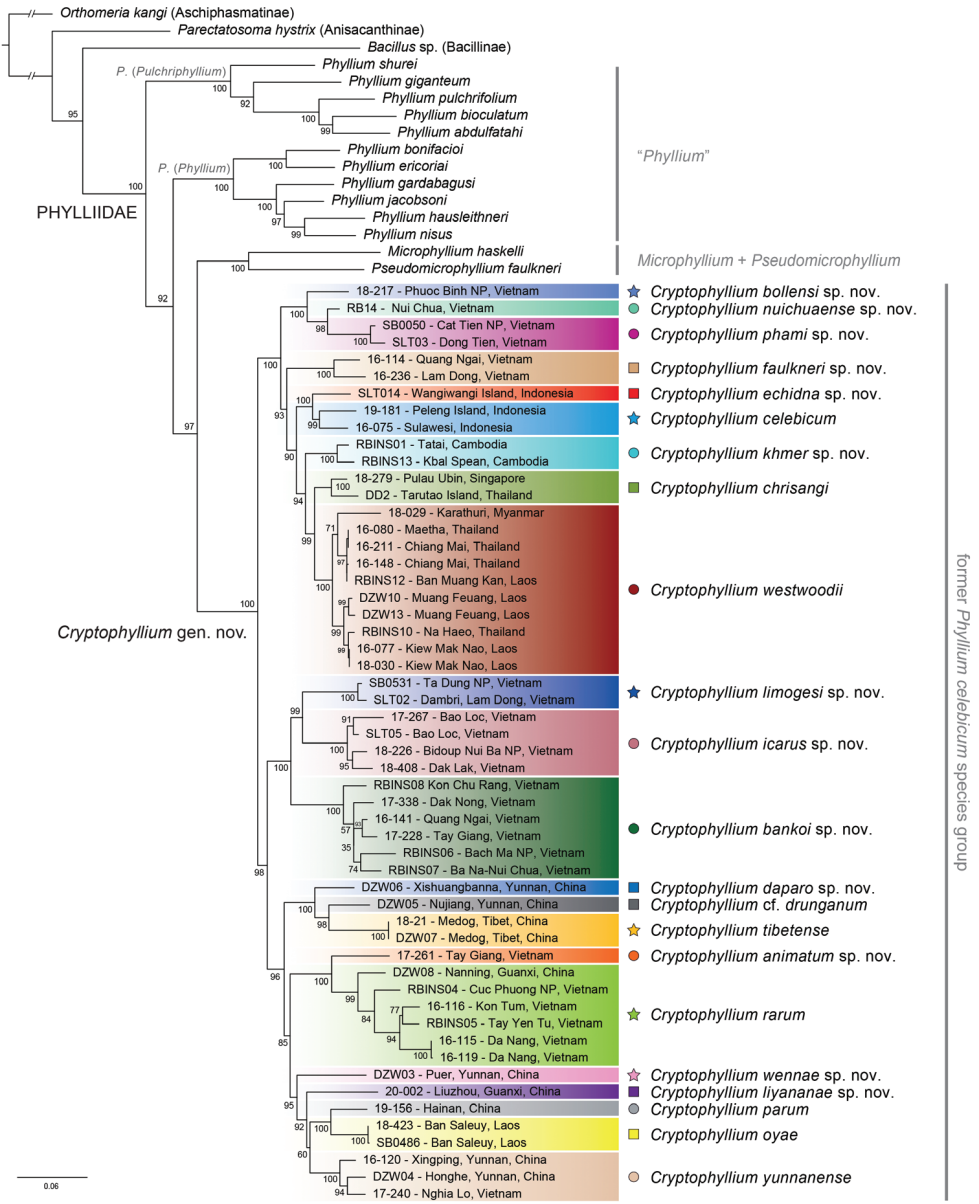
Phylogenetic relationships among 35 phylliid species (67 specimens). The species of the *Cryptophyllium* gen. nov. are highlighted with colors and symbols corresponding to the distribution map (Fig. 2). Support values based on maximum likelihood (UFBoot, IQ-TREE) are indicated at each node.

The morphological review of all examined specimens revealed 20 species of which eleven could be differentiated from already described species. In order to confirm our assumptions, we conducted a phylogenetic analysis based on a molecular dataset comprising 1804 characters resulting from the concatenation of 69 sequences of the COI, 39 of the 16S, and 33 of the 28S gene. The phylogenetic inference under Maximum Likelihood (Figure 4) recovered Phylliidae as monophyletic with maximum support. The *Phyllium* was recovered paraphyletic with *Microphyllium* + *Pseudomicrophyllium* as sister group to the Phyllium (Phyllium) celebicum species group. The specimens assigned to this group, here described as the *Cryptophyllium* gen. nov. based on morphology, formed a strongly supported monophyletic group. Species were recovered as distinct clades as initially proposed by morphology. In two cases, the molecular phylogeny revealed cryptic species.

### Taxonomy

#### Phylliidae Brunner von Wattenwyl, 1893

Phylliidae can be differentiated from other phasmids by the broad, dorsolaterally flattened abdomen and flattened femoral lobes (and for many taxa lobes on the tibia as well), giving them a distinct leaf-like appearance; a protuberance on the attachment site in the head capsule from the dorsal tentorial arms; and dorsoventrally flattened maxillary- and labial palps (Bedford 1978; and see other notable works for additional discussion on phylliid apomorphies: Bradler 2009; Klug and Bradler 2006; Friedemann et al. 2012). Additionally, to add believability to the leaf shape, the phylliids come in leaf-like colors such as green, brown, yellow, and a wide variety of mixed color forms and even sway as they walk like leaves in the wind. Currently, there are two divisions of leaf insects within the singular Phylliinae, the Phylliini Brunner von Wattenwyl, 1893 (which contains the bulk of genera and species) and the Nanophylliini Zompro & Groesser, 2003 (which only contains the *Nanophyllium* Redtenbacher, 1906) (Brock et al. 2020). Our molecular and morphological review has warranted the description of a novel genus of leaf insect to accommodate the former *celebicum* species group (sensu Hennemann et al. 2009), which is erected herein.

#### Species boundary interpretations

We determined our species boundaries based upon molecular, morphological, and geographic data. Although this was not done objectively through the use of species delimitation programs, we felt that due to the gaps in our sampling, a case-by-case delimitation was necessary based on a thorough understanding of the taxa in question.

In some instances, species boundaries were easy to interpret as molecular and morphological data clearly agreed with little room for abstract interpretation. For example, note the clade in Fig. 4 containing *Cryptophylliumlimogesi* gen. et sp. nov., *Cryptophylliumicarus* gen. et sp. nov., and *Cryptophylliumbankoi* gen. et sp. nov. where these three species are 1) molecularly unique, 2) morphologically easily distinguishable, and 3) geographically sympatric (see inset within Fig. 2).

However, not all species boundary interpretations were as clearly observed, and our boundary lines are less distinctly defined for some clades (e.g., see Fig. 4 with our treatment of *Cryptophylliumwestwoodii* comb. nov., *Cryptophylliumrarum* comb. nov., or the clade formed by *Cryptophylliumbollensi* gen. et sp. nov., *Cryptophylliumnuichuaense* gen. et sp. nov., and *Cryptophylliumphami* gen. et sp. nov.). In instances where our molecular results gave ambiguous delimitations, we turned to morphology and biogeography to help clarify our species boundaries for this work.

Within the instances of *Cryptophylliumwestwoodii* comb. nov. and *Cryptophylliumrarum* comb. nov. we found these to be geographically widespread populations with notable molecular variability (which one would expect from a widespread population of not particularly mobile insects). When reviewing the morphology of these two groups we were unable to identify a consistent and reliable morphological feature for differentiation to warrant splitting of these clades into multiple species. Additionally, we maintain these somewhat molecularly variable clades due to our present lack of extensive molecular sampling from throughout their large geographic distributions. For example, in Figure 4 note the *Cryptophylliumwestwoodii* comb. nov. sample 18-029 from Karathuri, Myanmar. Although this sample has a significant molecular distance from the northern Thailand samples to which it is most closely related, we lack additional molecular data from the over 800 kilometers between this location and the samples from the north. Considering the lack of morphological features for differentiation and the possibility that within this significant 800 kilometer range is a continuous population filling in the molecular distances between these north/south extremes, we felt a conservative approach was best. Therefore, even though these expansive clades may in fact represent more than one species in reality, we felt it premature to split these clades apart and hope that future molecular analyses from these missing geographic areas can eventually bring greater clarity to these populations.

In contrast, similar molecular distances were observed within the clade formed by *Cryptophylliumbollensi* gen. et sp. nov., *Cryptophylliumnuichuaense* gen. et sp. nov., and *Cryptophylliumphami* gen. et sp. nov. as were observed within the diverse *Cryptophylliumwestwoodii* comb. nov. or *Cryptophylliumrarum* comb. nov. but with the important caveats that 1) these three species had notable molecular distance between them, 2) these three could reliably and consistently be morphologically separated, and 3) due to their geographic proximity and extensive sampling within this area we do not have large areas of unsampled uncertainty (with the distance between these three species ranging from only 60–100 kilometers apart).

Therefore, although our interpretations at the present may not perfectly represent biological reality, we feel that due to limited data a conservative and individual approach was best for species delimitation within this work. For additional details on our species boundary determinations please see the individual species sections below for individual discussions where warranted.

##### Cryptophyllium gen. nov.

Taxon classificationAnimalia

4AF24F80-7CB0-58BD-9C2B-DB85EC87C7C1

http://zoobank.org/35EF6752-C08C-4583-8516-A7AA3167B704

###### Type species.

*Phylliumcelebicum* de Haan, 1842: 111, herein designated.

###### Differentiation.

**Figure 5.**
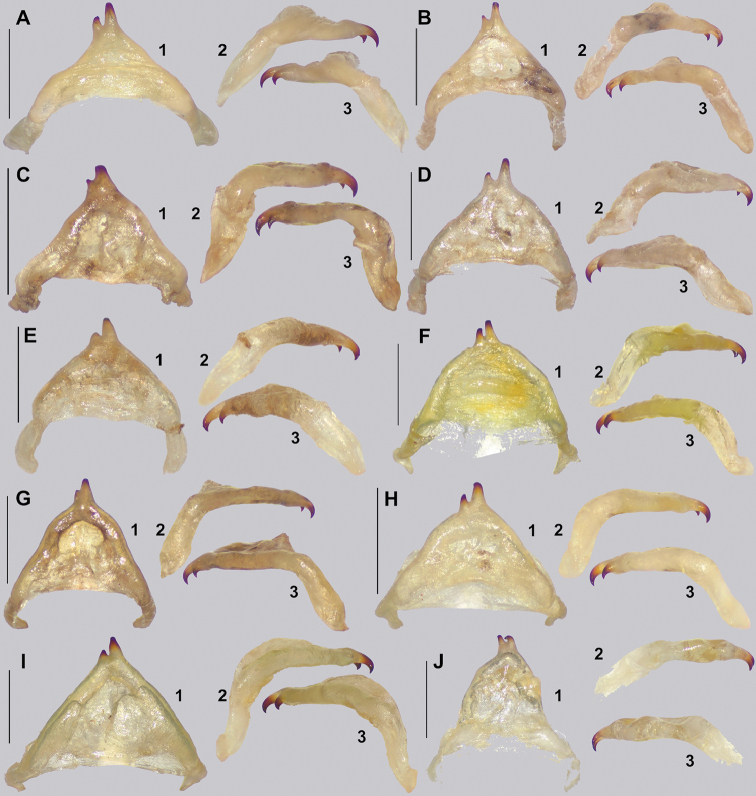
Details of the *Cryptophyllium* gen. nov. male vomer. All from the RBINS collection, prepared and photographed by Jérôme Constant (RBINS). Views **1** ventral **2** right lateral of the ventrally oriented vomer **3** left lateral of the ventrally oriented vomer **A***Cryptophylliumwestwoodii* comb. nov., Thailand, Na Haeo **B***Cryptophylliumkhmer* gen. et sp. nov. Cambodia, Kbal Spean **C***Cryptophylliumphami* gen. et sp. nov., Vietnam, Cat Tien **D***Cryptophylliumkhmer* gen. et sp. nov., Cambodia, Tatai **E***Cryptophylliumbollensi* gen. et sp. nov., Vietnam, Phuoc Binh **F***Cryptophylliumoyae* comb. nov., Laos, Mt. Phu Phan **G***Cryptophylliumbankoi* gen. et sp. nov., Vietnam, Ba Na-Nui Chua **H***Cryptophylliumicarus* gen. et sp. nov., Vietnam, Bidoup-Nui Ba **I***Cryptophylliumrarum* comb. nov., Vietnam, Cuc Phuong **J***Cryptophylliumlimogesi* gen. et sp. nov., Vietnam, Ta Dung. Scale bars, 1.0 mm.

The only single feature that alone can distinguish *Cryptophyllium* gen. nov. from others phylliids is the vomer of the males, which has two apical hooks (Fig. 5). Größer (2011) was the first to review the vomer morphology across a wide sampling of species and noted that the vomer of males in this group is two-hooked, whereas all other known phylliid males have a singularly hooked vomer. Besides this distinct feature, it is a combination of other features, not a singular feature, which allows differentiation of *Cryptophyllium* gen. nov. from the other phylliid genera. Morphologically, *Cryptophyllium* gen. nov. falls within the Phylliini due to the posteromedial tubercle being singular, not split into two lobes (a feature which differentiates Phylliini and Nanophylliini; Cumming et al. 2020c).

Genera and subgenera included within the Phylliini Brunner von Wattenwyl, 1893 are: *Chitoniscus* Stål, 1875; *Microphyllium* Zompro, 2001; *Pseudomicrophyllium* Cumming, 2017; Phyllium (Phyllium) Illiger, 1798; Phyllium (Pulchriphyllium) Griffini, 1898; Phyllium (Comptaphyllium) Cumming, Le Tirant, & Hennemann, 2019; Phyllium (Walaphyllium) Cumming, Thurman, Youngdale & Le Tirant, 2020, and the herein described *Cryptophyllium* gen. nov. To differentiate *Cryptophyllium* gen. nov. from other Phylliini and place it taxonomically we present a key to genera for each sex individually as well as a key to egg morphology.

### Key to females of the genera of the Phylliini Brunner von Wattenwyl, 1893

Unknown for *Pseudomicrophyllium* Cumming, 2017

**Table:** 

1	Tegmina venation with the posterior cubitus split into an anterior cubitus (CuA), first posterior cubitus (first posterior cubitus), and second posterior cubitus (CuP2)	**Phyllium (Walaphyllium) Cumming et al. 2020**
–	Tegmina cubitus venation simple (unsplit) or bifurcate (into an anterior cubitus (CuA) and posterior cubitus (CuP1) only)	**2**
2	Terminal antennomere as long as the preceding four or five segments combined	**3**
–	Terminal antennomere as long as the preceding one or two segments combined	**4**
3	Prescutum sagittal plane with two prominent spines, one on the anterior rim and another along the sagittal plane in the middle of the length; small species (42.0–47.0 mm long); restricted to the Philippines	***Microphyllium* Zompro, 2001**
–	Prescutum anterior rim with a prominent singular spine, the remainder of the sagittal plane of the prescutum with small nodes; small to large species (56.0–102.0 mm long)	**Phyllium (Comptaphyllium) Cumming et al. 2019**
4	Prescutum which is wider than long (ca. 2× wider)	***Chitoniscus* Stål, 1875**
–	Prescutum which is the same width as length, or notably longer than wide	**5**
5	Tegmina with media and cubitus veins running side by side and touching throughout the majority of their lengths	**Phyllium (Pulchriphyllium) Griffini, 1898**
–	Tegmina with media and cubitus veins distinctly separated with several vein widths distance between them throughout the length, not touching	**6**
6	Antennae typically with the fourth segment short and disk-like at least 3× wider than long and notably shorter than any of the following three segments, or rarely a similar length to the following segment, but still at least 2× as wide as long	***Cryptophyllium* gen. nov.**
–	Antennae with the fourth antennal segment as tall as wide and of a similar height to each of the following three segments length, not notably shorter	**Phyllium (Phyllium) Illiger, 1798**

### Key to males of the genera of the Phylliini Brunner von Wattenwyl, 1893

**Table:** 

1	Small (< 30.0 mm in length); protibiae lacking an interior lobe; restricted to the Philippines	**2**
–	Medium to large (35.0 mm to > 80.0 mm); protibiae almost always with a half to fully developed interior lobe, or rarely highly reduced to a sliver on the proximal half only	**3**
2	Antennae short (only ca. the length of the outstretched front legs), with bead-like antennomeres that are no more than 2× longer than they are wide	***Microphyllium* Zompro, 2001**
–	Antennae notably longer than the outstretched front legs, with antennomeres 4–5× longer than wide	***Pseudomicrophyllium* Cumming, 2017**
3	Prescutum stout, ca. 2× as wide as long	***Chitoniscus* Stål, 1875**
–	Prescutum as long as wide or notably longer than wide	**4**
4	Vomer with two apical hooks	***Cryptophyllium* gen. nov.**
–	Vomer with a single apical hook	**5**
5	Alae radial sector, media anterior, and media posterior veins fusing to the cubitus at different locations along the vein and running together to the wing margin	**Phyllium (Pulchriphyllium) Griffini, 1898**
–	Alae radial sector, media anterior, and media posterior not fusing with the cubitus	**6**
6	Tegmina media vein splits into the anterior media vein (MA) and posterior media vein (MP) very early on, immediately or at most ¼ of the way through the wing length and they run unbranched and subparallel through the wing length; protibial interior lobe not reaching from end to end of the shaft, only restricted to the proximal ½ to ⅔ but never more; a head capsule with clearly defined nodes arranged in evenly spaced patterns	**Phyllium (Comptaphyllium) Cumming et al. 2019**
–	Tegmina media vein running unbranched for the first ⅓ to ⅖ of the wing length, and then branching with either a single short media posterior running to the wing margin or two short media posteriors branching from the notably longer media anterior and running to the margin; protibial interior lobe variable, either fully spanning the full length or only ½ of the length; head capsule at most with random granulation but frequently bare	**7**
7	Abdominal shape rectangular, with segments V and VI with fully parallel-sided margins (segments IV and VII with only half parallel-sided and the remainder curved)	**Phyllium (Walaphyllium) Cumming et al. 2020**
–	Abdominal shape variable, either spade-shaped (with the margins of V parallel or strongly converging and segment VI strongly converging), ovular (with margins expanding and then contracting, no segments parallel-sided), thin and slender with converging margins, bell-shaped (with margins expanding until segment VI then strongly converging) or boxy with only segment V parallel-sided (segments IV and VI only partially parallel-sided, the remainder rounded	**Phyllium (Phyllium) Illiger, 1798**

### Key to eggs of the genera of the Phylliini Brunner von Wattenwyl, 1893

Unknown for *Microphyllium* Zompro, 2001 and *Pseudomicrophyllium* Cumming, 2017

**Table:** 

1	Surface lacking pinnae, instead porous and stiffly spongy	**2**
–	Surface with pinnae (moss-, rope- or feather-like)	**4**
2	Lateral margins fanned out into distinct fins with an operculum which is typically longer than wide (but not always), or if the capsule fins are reduced (not prominently protruding), the egg in cross-section is distinctly triangular (not pentagonal or rectangular), with the dorsal surface notable broader than the other surfaces	**Phyllium (Pulchriphyllium) Griffini, 1898**
–	Capsule boxy and rectangular without distinct fins and the operculum is always notable wider than long (length ca. ½ the greatest width)	**3**
3	Eggs medium to large, 5.0–7.0 mm long	**Phyllium (Walaphyllium) Cumming et al. 2020**
–	Eggs small, ca. 3.0 mm long	***Chitoniscus* Stål, 1875**
4	Pinnae short and moss-like over the entire capsule	***Cryptophyllium* gen. nov.**
–	Pinnae long and feather- or rope-like	**5**
5	Operculum with a row of pinnae along the sagittal plane, not pinnae encircling the margin of the operculum	**Phyllium (Comptaphyllium) Cumming et al. 2019**
–	Operculum with pinnae encircling the margin, not along the sagittal plane	**Phyllium (Phyllium) Illiger, 1798**

#### Generic characteristics

**Figure 6.**
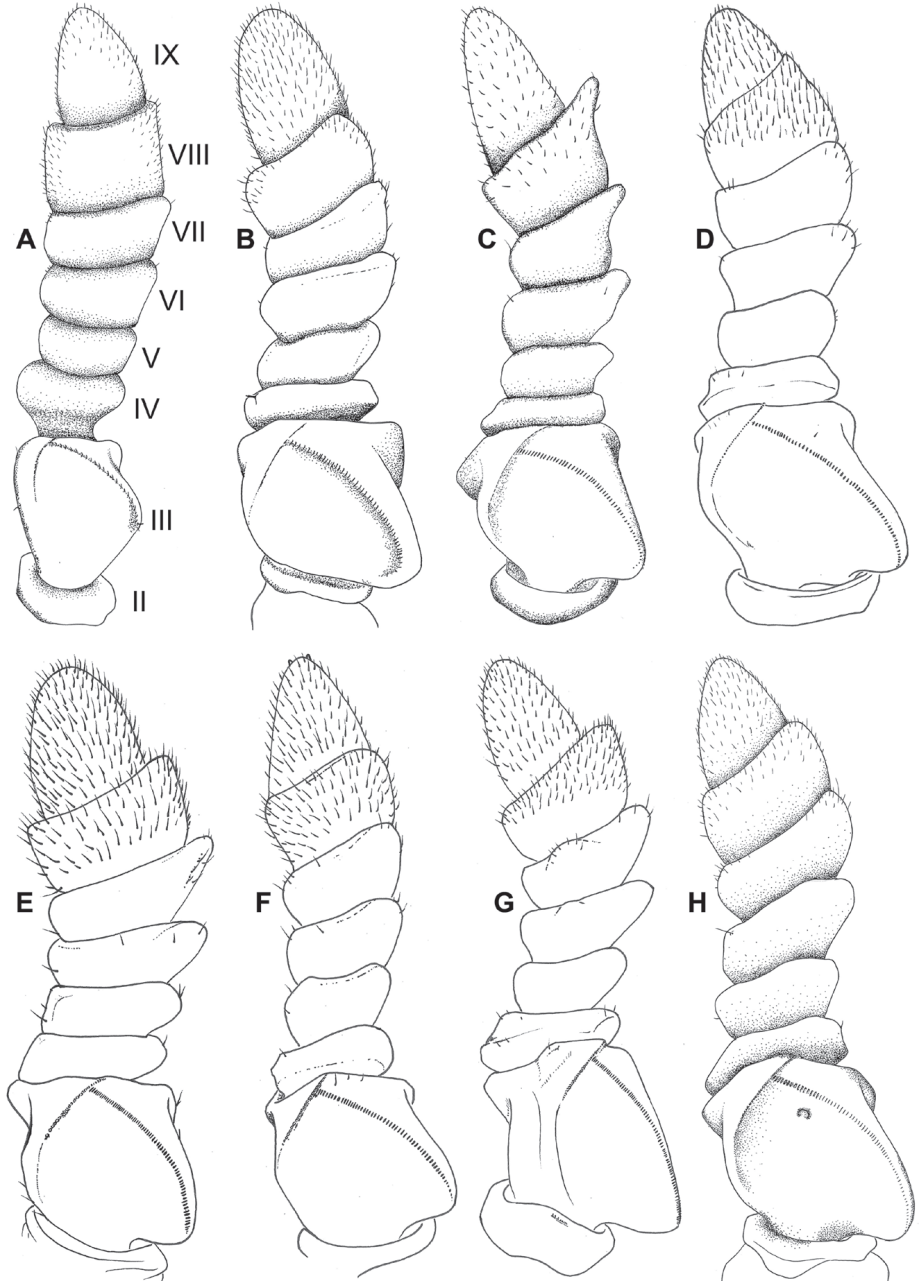
Details of female phylliid antennae illustrated from specimens within the RBINS collection, illustrations by Julien Caudron (RBINS). Note that the figures start with the scapus, therefore the diagnostic fourth antennomere is the third one shown; to ensure clarity **A** has the segment number listed to the right of the image. Note that the fourth antennomere of *Phyllium* (**A**) is longer than the fifth antennomere in comparison to the *Cryptophyllium* gen. nov. representatives (**B–H**), which have the fourth antennomere short and disk-like **A***Phylliumericoriai*, Philippines **B***Cryptophylliumbollensi* gen. et sp. nov., Vietnam, Phuoc Binh **C***Cryptophylliumcelebicum* comb. nov., Indonesia, Sulawesi **D***Cryptophylliumkhmer* gen. et sp. nov., Cambodia, Siem Reap Province **E***Cryptophylliumnuichuaense* gen. et sp. nov., Vietnam, Nui Chua **F***Cryptophylliumphami* gen. et sp. nov., Vietnam, Cat Tien **G***Cryptophylliumwestwoodii* comb. nov., Laos, Bokeo Province **H***Cryptophylliumwestwoodii* comb. nov., Thailand, Chiang Mai. Drawn to relative scale.

**Figure 7.**
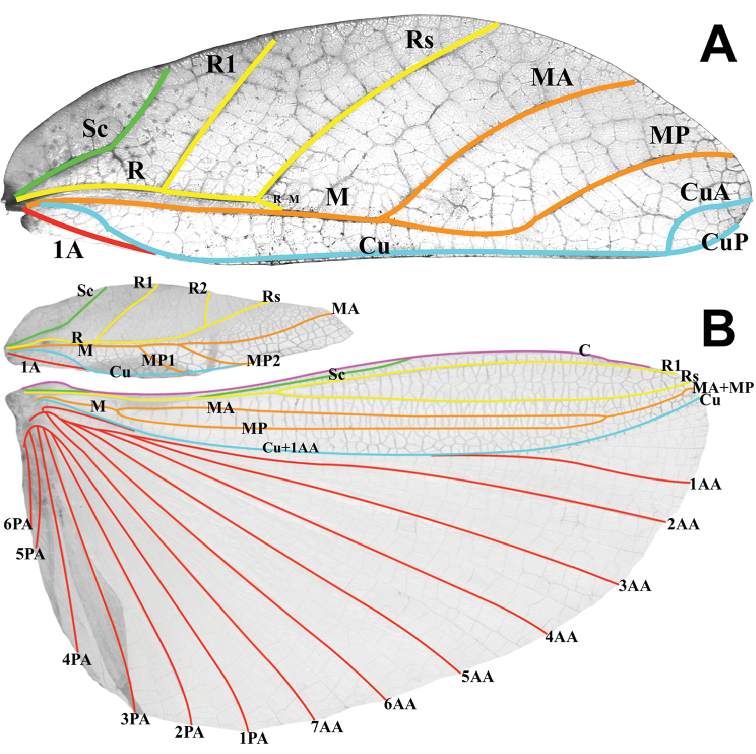
Typical tegmina and alae venation present in the *Cryptophyllium* gen. nov., photographed by RTC **A** female: *Cryptophylliumcelebicum* comb. nov. (Coll RC 16-075) **B** male: *Cryptophylliumyunnanense* comb. nov. (Coll RC 16-120). Abbreviations: C (costa); Sc (subcosta); R (radius); R1 (radius 1); R2 (radius 2); Rs (radial sector); M (media); MA (media anterior); MP (media posterior); MP1 (first media posterior); MP2 (second media posterior); MA+MP (media anterior fused with media posterior); Cu (cubitus); CuA (cubitus anterior); CuP (cubitus posterior); Cu+1AA (cubitus and first anterior anal); 1A (first anal); 1AA–7AA (first–seventh anterior anal); 1PA–7PA (first–seventh posterior anal).

In general, the *Cryptophyllium* gen. nov. are medium to large, with females ranging from 77.0 mm (in the smallest recorded *Cryptophylliumathanysus* comb. nov.; Hennemann et al. 2009) to 107.0 mm long (in the only recorded *Cryptophylliumdaparo* sp. nov.), and males from 55.4 mm (in the smallest recorded *Cryptophylliumbollensi* sp. nov.) to 89.4 mm (in the only recorded *Cryptophylliumanimatum* sp. nov.). Both sexes only have interior tibial lobes on the protibiae and some species either have full exterior meso-, and metatibial exterior lobes; lobes on the pro-, meso-, and metatibial exterior, which are small and reduced to the distal end only; or have meso-, and metatibiae, which are completely devoid of exterior lobes. Females have antennae with nine segments of which the fourth antennal segment is short and disk-like (Fig. 6) and males have antennae which range between 24 and 29 segments. Exterior profemoral lobes on both sexes can be rounded and obtuse, roundly angled, distinctly right angled, or even acutely angled with recurved exterior angles. Interior profemoral lobes can have uniformly sized and spaced teeth or doubly serrate and triangular teeth in a variety of arrangements, but generally there are five teeth arranged in a two-one-two pattern. The thorax in both sexes is variable with mesopleura that can be narrow on the anterior and only fanning out halfway through the length, or on the other extreme, broad and uniformly diverging throughout the entire length, with some species exhibiting a somewhat intermediate range of shapes. Female tegmina are always long and have a subcoastal vein, radial vein split into the first radial and radial sector and ending in a small radial to medial crossvein, a bifurcate medial vein, a bifurcate cubitus vein, and a first anal which fuses with the cubitus early on (Fig. 7A). Females can have highly reduced alae (rare) or more commonly decently developed alae which can be half to almost the same length as the tegmina. Male tegmina range in length slightly with most species having tegmina, which reach onto abdominal segment III or less commonly longer onto abdominal segment IV. Male tegminal venation has two media posterior veins and the cubitus is unbranched. Male alae are always long and fully developed with a set of coastal and subcoastal veins on the anterior margin, a radial split into a first radius and radial sector which do not reconnect before terminating on the wing margin, a media which splits into the media anterior and media posterior but then reconnects and runs fused to the wing margin, and a typical set of a cubitus and anal veins filling the remainder of the alae as a fused cubitus and first anterior anal which splits before the wing margin, seven total anterior anals and typically five to seven posterior anals (Fig. 7B). Abdominal shape is variable in both sexes and can be boxy, spade-shaped, ovoid, narrow and long, or strongly tapered. Female subgenital plates can be short and stout or very long and thin with the apex reaching the tip of the abdomen. Males have a unique vomer which is two-pronged, with the hooks either the same size and shape or with one more prominent hook and a second smaller hook adjacent to the first (Fig. 5).

**Figure 8.**
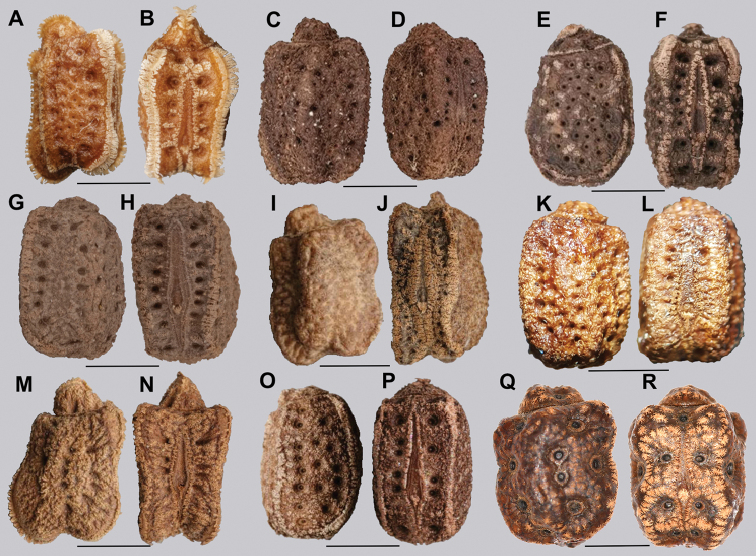
Known eggs for the *Cryptophyllium* gen. nov. set in couplets of lateral and then dorsal views. All images except for K and L were taken by Bruno Kneubühler (Switzerland), K and L images taken by the first author, Q and R images taken by René Limoges (IMQC) **A, B***Cryptophylliumwestwoodii* comb. nov. **C, D***Cryptophylliumtibetense* comb. nov. **E, F***Cryptophylliumbollensi* gen. et sp. nov. **G, H***Cryptophylliumoyae* comb. nov. **I, J***Cryptophylliumicarus* gen. et sp. nov. **K, L***Cryptophylliumliyananae* gen. et sp. nov. **M, N***Cryptophylliumchrisangi* comb. nov. **O, P***Cryptophylliumcelebicum* comb. nov. **Q, R***Cryptophylliumlimogesi* gen. et sp. nov. Scale bars 2.0 mm long.

Egg morphology is variable in regard to color and pitting size/arrangement, but the general features are relatively uniform. All known eggs are relatively small without strongly formed lateral fins. Instead, the eggs are rather boxy in appearance with relatively straight margins and short moss-like pinnae on most surfaces. The egg operculum is conically raised and shares a similar surface texture to the overall egg with short moss-like pinnae throughout, but typically with slightly longer pinnae along the margins of the operculum (Fig. 8).

**Figure 9.**
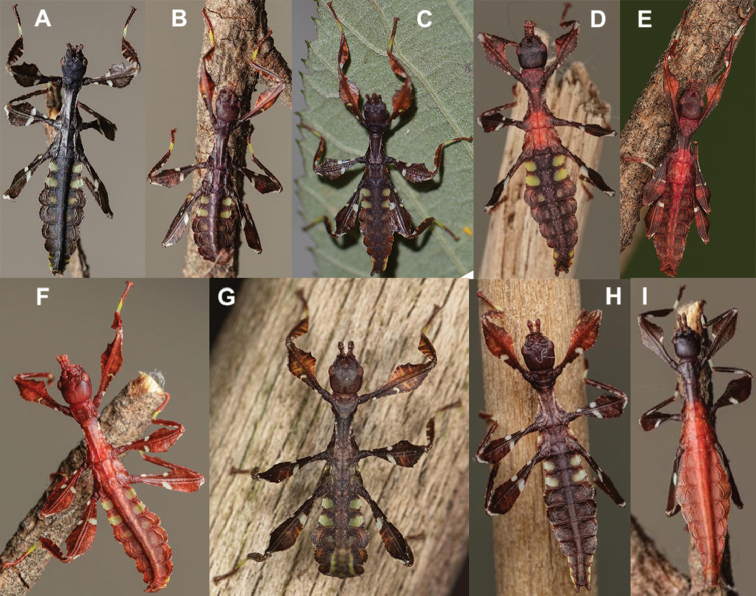
Freshly hatched nymphs known for the *Cryptophyllium* gen. nov., all except for C bred and photographed by Bruno Kneubühler (Switzerland) **A***Cryptophylliumcelebicum* comb. nov. **B***Cryptophylliumchrisangi* comb. nov. **C***Cryptophylliumkhmer* gen. et sp. nov. bred and photographed by Tim Bollens (Belgium) **D***Cryptophylliumtibetense* comb. nov. **E***Cryptophylliumoyae* comb. nov. **F***Cryptophylliumwestwoodii* comb. nov. **G***Cryptophylliumphami* gen. et sp. nov. **H***Cryptophylliumbollensi* gen. et sp. nov. **I***Cryptophylliumicarus* gen. et sp. nov.

Freshly hatched nymphs have a base coloration of brown to red, and many of the known species have abdominal segments II and III with distinct green patches which stand out from the rest of the general nymph coloration. All freshly hatched nymphs have partial white bands on their meso- and metafemoral lobes, and some have these white bands weakly to fully formed on their profemoral lobes as well (Fig. 9). The nymphs with bright red coloration such as *Cryptophylliumicarus* sp. nov. (Fig. 9I) bear a striking resemblance to nymphs of *Hymenopuscoronatus* Olivier, 1792 (Mantodea: Hymenopodidae) and *Paradasynus* spp. China, 1934 (Hemiptera: Coreidae) with their two-toned aposematic coloration (Hawkeswood and Sommung 2019). It appears as though *Hymenopuscoronatus* and *Cryptophylliumicarus* sp. nov. freshly emerged nymphs are benefitting in this aposematic mimicry which is common in the Pentatomorpha true bugs warning of their distastefulness (Burdfield-Steel and Shuker 2014).

**Distribution.** Sri Lanka to the west, Tibet to the north, Micronesia to the east, and Wangi-wangi Island, Indonesia to the south (Fig. 2). To date we have recorded *Cryptophyllium* gen. nov. from 14 countries with China and Vietnam the current hotspots of diversity with nine and eleven species presently known from each, respectively (Fig. 2).

**Etymology.** Adjectival, meaning the hidden leaves. From Greek *kryptós* = hidden + *phyllium* = leaf. This genus is neuter in gender like *Phyllium*. We chose this name with a double meaning in mind. First, this clade of leaf insects has been “hidden” within the true *Phyllium*, and only recently have they been noted as unique and placed within the *celebicum* species group by Hennemann et al. (2009) of *Phyllium*, but still not completely recognized as separate. Secondly, these insects are masters of disguise and well-hidden within their canopy habitat (Fig. 1) which means only rarely are individuals found.

**Species included within *Cryptophyllium* gen. nov.** (listed and treated below alphabetically).

*Cryptophylliumanimatum* gen. et sp. nov.

*Cryptophylliumathanysus* (Westwood, 1859), comb. nov.

*Cryptophylliumbankoi* gen. et sp. nov.

*Cryptophylliumbollensi* 1gen. et sp. nov.

*Cryptophylliumcelebicum* (de Haan, 1842), comb. nov.

*Cryptophylliumchrisangi* (Seow-Choen, 2017), comb. nov.

*Cryptophylliumdaparo* gen. et sp. nov.

*Cryptophylliumdrunganum* (Yang, 1995), comb. nov.

*Cryptophylliumechidna* gen. et sp. nov.

*Cryptophylliumfaulkneri* gen. et sp. nov.

*Cryptophylliumicarus* gen. et sp. nov.

*Cryptophylliumkhmer* gen. et sp. nov.

*Cryptophylliumlimogesi* gen. et sp. nov.

*Cryptophylliumliyananae* gen. et sp. nov.

*Cryptophylliumnuichuaense* gen. et sp. nov.

*Cryptophylliumoyae* (Cumming & Le Tirant, 2020), comb. nov.

*Cryptophylliumparum* (Liu, 1993), comb. nov.

*Cryptophylliumphami* gen. et sp. nov.

*Cryptophylliumrarum* (Liu, 1993), comb. nov.

*Cryptophylliumtibetense* (Liu, 1993), comb. nov.

*Cryptophylliumwennae* gen. et sp. nov.

*Cryptophylliumwestwoodii* (Wood-Mason, 1875), comb. nov.

*Cryptophylliumyapicum* (Cumming & Teemsma, 2018), comb. nov.

*Cryptophylliumyunnanense* (Liu, 1993), comb. nov.

#### Species recorded per country

Nepal

*Cryptophylliumtibetense*: Gandaki Pradesh (Tanahun District); Province No. 1 (Ilam)

India

*Cryptophylliumtibetense*: West Bengal (Kalimpong); Sikkim; Assam (Digboi)

*Cryptophyllium* sp.: Andaman Islands [Wood-Mason 1875]

Bhutan

None yet known, but biogeographically assumed *Cryptophyllium* gen. nov. should be present.

Bangladesh

None yet known, at this time we are only aware of Phyllium (Pulchriphyllium) bioculatum scythe Gray, 1843 from Bangladesh, but biogeographically assumed *Cryptophyllium* gen. nov. should be present as well.

China

*Cryptophylliumtibetense*: Tibet (Xizang Autonomous Region), Mêdog County (Motuo); Southeast Tibet (Tenga Valley, Anjaw District, Lohit District, East Siang district);

*Cryptophylliumdrunganum*: Yunnan Province (Nùjiāng Lisu Autonomous Prefecture)

*Cryptophylliumyunnanense*: Yunnan Province (Xishuangbanna Dai Autonomous Prefecture, Pu’er prefecture-level city, Honghe Hani and Yi Autonomous Prefecture, Yuxi prefecture-level city)

*Cryptophylliumwennae*: Yunnan Province (Xishuangbanna Dai Autonomous Prefecture, Pu’er prefecture-level city)

*Cryptophylliumdaparo*: Yunnan Province (Xishuangbanna Dai Autonomous Prefecture)

*Cryptophylliumrarum*: Guangxi Zhuang Autonomous Region (Wuzhou prefecture-level city, Baise prefecture-level city, Nanning City)

*Cryptophylliumparum*: Hainan Island (Baisha Li Autonomous County, Ledong Li Autonomous County)

*Cryptophylliumliyananae*: Guangxi Zhuang Autonomous Region (Wuzhou prefecture-level city)

*Cryptophylliumoyae*: Yunnan Province (Maguan County)

Myanmar

*Cryptophylliumwestwoodii*: Kayin Province, Tanintharyi Province (Karathuri)

*Cryptophyllium* sp.: Mandalay Region (Myingyan District)

Vietnam

*Cryptophylliumoyae*: Ha Giang Province (Tung Ba Commune)

*Cryptophylliumyunnanense*: Lai Chau Province (Mount Fansipan), Yen Bai Province (Nghia Lo)

*Cryptophylliumrarum*: Vinh Phuc Province (Tam Dao), Quang-Ninh Province (Tay Yen Tu), Ninh Binh Province (Cuc Phuong), Da Nang Province (Ba Na), Kon Tum Province (Ngoc Linh), Hoa Binh Province

*Cryptophylliumanimatum*: Quang Nam Province (Tay Giang)

*Cryptophylliumbankoi*: Thua Thien Hue Province (Bach Ma), Da Nang Province (Ba Na Nui Chua), Quang Nam Province (Axan)

*Cryptophylliumbollensi*: Ninh Thuan Province (Phuoc Binh N.P.)

*Cryptophylliumphami*: Dong Nai Province (Cat Tien N.P.), Binh Thuan Province (Dong Tien)

*Cryptophylliumnuichuaense*: Ninh Thuan Province (Nui Chua N.P.)

*Cryptophylliumicarus*: Lâm Dông Province (Bidoup Nui Ba N.P., Bao Loc), Dak Lak Province

*Cryptophylliumlimogesi*: Dak Lak Province (Chu Yang Sin), Dak Nong Province (Ta Dung), Lam Dong Province (Dambri)

*Cryptophylliumfaulkneri*: Quang Ngai Province (Bato), Lam Dong Province (Bao Lam)

Laos

*Cryptophylliumoyae*: Houaphan Province (Xamnuen District)

*Cryptophylliumwestwoodii*: Bokeo Province (Ban Muang Kan), Louang Prabang Province, Vientiane Province (Muang Feuang District)

Thailand

*Cryptophylliumwestwoodii*: Chiang Mai Province (Fand District), Lamphun Province (Mae Tha District), Loei Province (Na Haeo)

*Cryptophylliumchrisangi*: Nakhon Si Thammarat Province (Thung Song District), Satun Province (Tarutao Island)

*Cryptophylliumoyae*: Nan Province (Bo Kluea Tai District), Phetchabun Province, Chiang Mai Province

Cambodia

*Cryptophylliumkhmer*: Koh Kong Province (Tatai), Siem Reap Province (Kbal Spean)

Sri Lanka

*Cryptophylliumathanysus*: Southern Province (Sinharaja Forest Reserve)

Philippines

*Cryptophyllium* sp. “Cebu”: Cebu Island

*Cryptophyllium* sp. “Zamboanga”: Mindanao (Zamboanga Peninsula)

Malaysia

*Cryptophylliumchrisangi*: Perak (Tapah), Sarawak State (Kuching)

Singapore

*Cryptophylliumchrisangi*: mainland Singapore and St. John’s Island

Indonesia

*Cryptophylliumchrisangi*: Pulau Weh Island (north coast of Sumatra)

*Cryptophyllium* sp. “Bangka”: Bangka Island (off the southeastern Sumatra coast)

*Cryptophylliumcelebicum*: Sulawesi; Peleng Island; and Buton Island

*Cryptophylliumechidna*: Wangi-wangi Island

Micronesia

*Cryptophylliumyapicum*: Yap Island

##### Cryptophyllium animatum

Taxon classificationAnimalia

gen. et sp. nov.

53CC67C8-7017-5165-968A-D28480EFC5C1

http://zoobank.org/0EA5C7BF-DD05-4889-AEBA-8203220B57CE

Figure 10

###### Material examined.

***Holotype*** ♂: “VIETNAM: Quang Nam, Tay Giang, Axan Mt, 1,300 meters: July 2017 (Coll RC 17-261)”. Deposited in the Montreal Insectarium (IMQC).

###### Remarks.

This large species is presently only known from the singular holotype male (89.4 mm in length) from Central Vietnam. With such a large male we look forward to when the female is located as the female must also be significantly large and possibly larger than any of the presently known *Cryptophyllium* gen. nov. females.

###### Differentiation.

**Figure 10.**
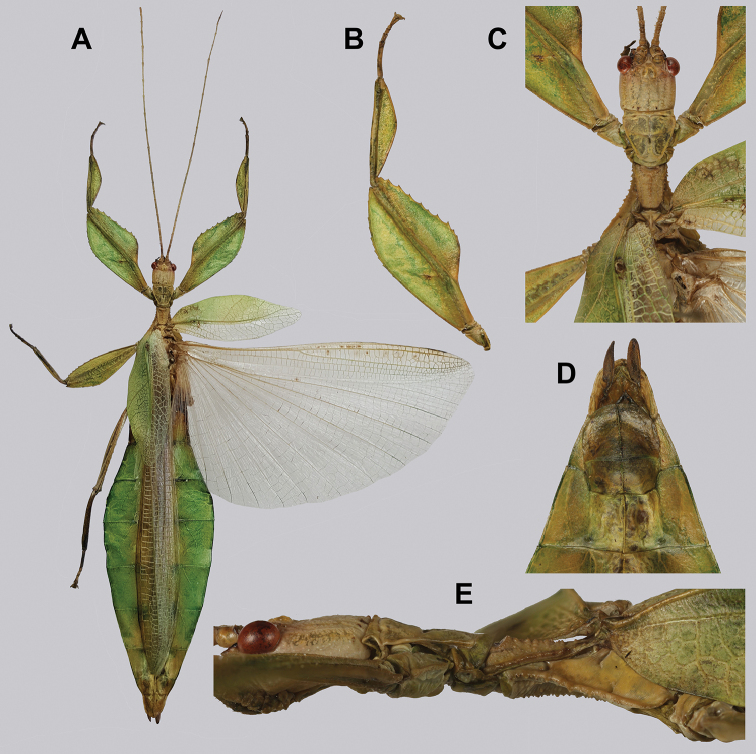
Holotype *Cryptophylliumanimatum* gen. et sp. nov., photographs by RTC **A** habitus, dorsal **B** details of the protibial and profemoral lobes **C** details of the head and thorax, dorsal **D** genitalia, ventral **E** head–thorax, lateral.

Females unknown. Males can be differentiated by the profemoral interior lobe which is marked by teeth which are uniform in size and spacing, a feature which no other known congenerics possess (Fig. 10B). Molecularly *Cryptophylliumanimatum* sp. nov. is most closely related to *Cryptophylliumrarum* comb. nov. and shares several morphological similarities such as the large size, serrated profemoral exterior lobe, similar tegmina lengths, and a similar shape of the mesothorax and similar arrangement/sizing of the teeth (Fig. 10C). These species can be differentiated, as *Cryptophylliumrarum* comb. nov. have profemoral interior lobe teeth, which are unevenly sized and spaced (Fig. 61E), and a broad rounded abdomen and not a thin spade-like abdomen as in *Cryptophylliumanimatum* sp. nov.

An additional species morphologically similar to *Cryptophylliumanimatum* sp. nov. is *Cryptophylliumfaulkneri* sp. nov., which can also be found in Central Vietnam. Morphological similarities between these species are the thin spade-shaped abdomens, large sizes, and similar tegmina lengths. Although superficially they look similar with their large thin appearance, it is easy to differentiate the species when the details of the thorax are observed, as *Cryptophylliumanimatum* sp. nov. has small numerous teeth throughout the mesopleura length (Fig. 10C) vs. *Cryptophylliumfaulkneri* sp. nov., which has four or five large tubercle-like teeth and smaller interspersed teeth between (Fig. 31A). Additionally, the exterior profemoral lobes differ in their serration as *Cryptophylliumanimatum* sp. nov. has eight or nine sharply serrate teeth (Fig. 10B) vs. *Cryptophylliumfaulkneri* sp. nov. which at most has four rounded, less prominent teeth (Fig. 31B).

###### Distribution.

Currently only known from the type locality in Central Vietnam: Quang Nam Province, Axan Mountain.

###### Male.

***Coloration.*** Coloration description based on the single dried holotype specimen, not on living individuals which are likely a more vibrant green. Overall coloration is pale green with variable patches of tan throughout due to the drying of the specimen (Fig. 10A). Compound eyes are cherry red (Fig. 10C). The antennae, head, and thorax are tan (Fig. 10C). Abdominal segment V has a set of eye spots which are clearer and situated closer to the midline than the margin.

***Morphology.****Head.* Head capsule slightly longer than wide, with a vertex that is weakly granular in a slightly longitudinal arrangement; posteromedial tubercle small but notable and slightly raised above the head capsule (Fig. 10C). Frontal convexity stout and finely pointed with two or three short setae near the apex. Compound eyes large but not overly bulbous, occupying > ⅓ of the head capsule lateral margins (Fig. 10C). Three ocelli are well-developed and located between and slightly posterior to the compound eyes. Antennal fields are slightly wider than, and approximately as long as the scapus. *Antennae.* Antennae (including the scapus and pedicellus) consists of 27 segments. The scapus and pedicellus are bare, all other segments are covered in dense, thin, pale setae that are as long as or longer than the antennae segment is wide, and the terminal three segments have shorter setae which are denser than the other segments. *Thorax.* Pronotum with anterior margin gently concave and lateral margins that are nearly straight and converge to a straight posterior margin that is approximately half the width of the anterior rim (Fig. 10C). Anterior and lateral margins of the pronotum with distinct rims and the posterior margin lacks a rim (Fig. 10C). Face of the pronotum is marked by a smooth surface, distinct sagittal furrow, pit just posterior to the center, a moderate perpendicular furrow just anterior to the central pit, and two distinct pits along the anterior margin (Fig. 10C). Prosternum is nearly smooth, with only a slightly wrinkled surface. Mesosternum surface marked densely with prominent nodes, with the largest along the sagittal plane and more strongly on the anterior margin, posterior margin with less prominent and slightly smaller nodes. Prescutum longer than wide, with lateral margins slightly converging to the posterior which is slightly narrower than the anterior rim width (Fig. 10C). Lateral rims with ten or eleven small tubercles of varying sizes, none large or prominent, most approximately even in size and one or two not much more prominent than nodes (Fig. 10C). The surface of the prescutum is heavily granulose and rises slightly up to meet the prescutum crest (Fig. 10E). Prescutum crest along the sagittal plane with seven or eight nodes which are denser on the anterior and slightly wider spaced on the posterior (Fig. 10E). In addition to the granular surface there is shallow pitting along the lateral margins, but none very prominent (Fig. 10C). Prescutum anterior rim with a heavily granulose surface and no distinct central tubercle (Fig. 10E). Mesopleura narrow, gradually diverging from the anterior to the posterior (Fig. 10C). Lateral margin with four or five moderately sized tubercles throughout the length, and between six or seven smaller minor tubercles interspersed throughout (Fig. 10C). Face of the mesopleura slightly wrinkled and with two distinct pits, one on the anterior ⅓ and one on the posterior ⅓. *Wings.* Tegmina moderate length, extending slightly > ½ through abdominal segment III. Tegmina wing venation (see Fig. 7B for general venation found in the *Cryptophyllium* gen. nov.): the subcosta (Sc) is the first vein and terminates the earliest, slightly > ca. ⅓ of the way through the overall tegmina length. The radius (R) spans nearly the entire length of the tegmina with the first radius (R1) branching just anterior to the middle and terminating just posterior to the middle of the wing, the second radius (R2) branches near the distal ⅓ of the wing, and then the radial sector (Rs) runs parallel and side by side with the media anterior until the posterior ⅕ of the tegmina, at which point the radial sector bends away from the media anterior and then terminates just shy of the tegmina apex. The media (M) spans the entire length of the tegmina, terminating at the wing apex as the media anterior (MA). The first media posterior (MP1) begins and terminates near the tegmina mid length followed by the second media posterior (MP2) which begins ca. ⅔ of the way through the tegmina length and terminates near the posterior quarter of the wing. The cubitus (Cu) runs through the wing surface angled towards the margin which it meets ca. ⅓ of the way through the tegmina length and then runs along the margin as the two media posterior veins then meet it and the cubitus eventually dissipates ca. ⅔ of the way through the tegmina length. The first anal (1A) vein runs subparallel to the cubitus until it meets it slightly > ⅓ of the way through the tegmina length. Alae well developed in an oval fan configuration, reaching to the anterior margin of abdominal segment IX. Alae wing venation (see Fig. 7B for general venation found in the species of this genus): the costa (C) is present along the entire foremargin giving stability to the wing. The subcosta (Sc) spans ca. ⅔ of the wing length and is mostly fused with the radius in the beginning but terminates when it meets the costa. The radius (R) spans the entire wing and branches slightly > ⅖ of the way through into the radius 1 (R1) and radial sector (Rs) which run nearly parallel through most of their length until they terminate at the wing apex near each other but not touching. The media (M) branches early, ca. ⅐ of the way through the wing into the media anterior (MA) and the media posterior (MP) which run parallel with each other until the distal ⅐ of the wing where the media posterior fuses with the media anterior which then run fused together to the wing margin. The cubitus (Cu) runs unbranched and terminates at the wing apex. Of the anterior anal veins, the first anterior anal (1AA) fuses with the cubitus near the point where the media branches into the media anterior and media posterior and then the first anterior anal branches from the cubitus ⅔ of the way through the wing length where it uniformly diverges from the cubitus until it terminates at the wing margin. The anterior anal veins two–seven (2AA–7AA) have a common origin and run unbranched in a folding fan pattern of relatively uniform spacing to the wing margin. The posterior anal veins (1PA–7PA) share a common origin separate from the anterior anal veins and run unbranched to the wing margin with slightly thinner spacing than the anterior anal veins. *Abdomen.* Abdomen general shape is a narrow spade. Abdominal segments II through the anterior half of segment IV slightly diverging to the widest point with the remainder of segment IV parallel sided. Segments V–IX are uniformly converging slowly with straight margins to the terminal abdominal segment X which at first also converges slowly but then quickly tapers off to a blunt rounded apex. *Genitalia.* Poculum broad and rounded, ending in a straight margined apex that passes beyond the anterior margin of segment X (Fig. 10D). Cerci long and slender, with ca. ½ of their length extending out from under the anal abdominal segment. The cerci are slightly cupped, covered in a granulose surface and numerous short setae throughout (Fig. 10D). Vomer broad and stout with straight sides evenly converging to the apex, which is armed with two upwards turning hooks, one ca. 2× as wide and ⅓ longer than the other smaller hook adjacent to it. *Legs.* Profemoral exterior lobe a rounded arc, only slightly wider than the interior lobe (ca. 3× the width of the profemoral shaft at its widest), and with the anterior half marked by eight or nine small but sharp anteriorly pointing teeth (Fig. 10B). Profemoral interior lobe rounded near the midline and then straight to the anterior margin, ca. 2½× as wide as the profemoral shaft at its widest. The profemoral interior lobe is marked with eight or nine small, serrate, anteriorly pointing teeth which are nearly uniform in size and spacing (Fig. 10B). Mesofemoral exterior lobe arcs end to end but with the widest portion distal to the midline, and the widest point about as wide as the interior lobe or the mesofemoral shaft at its widest. The mesofemoral exterior lobe is marked with three or four small serrate teeth distal to the widest point, with the proximal portion of the lobe smooth. The mesofemoral interior lobe is approximately the same width as the exterior lobe, but more evenly weighted from end to end with the widest portion near the midline. The distal half is marked with nine small serrate teeth with those most distal slightly closer together than those near the middle. Metafemoral exterior lobe lacks dentation, and has a straight margin along the metafemoral shaft. Metafemoral interior lobe smoothly arcs end to end with ten or eleven small serrate teeth on the distal half only. Protibiae lacking exterior lobe, interior lobe reaching end to end in a smooth triangle slightly > 2× as wide as the protibial shaft, with the widest point just distal to the midline (Fig. 10B). Mesotibiae and metatibiae simple, lacking lobes completely.

***Measurements of holotype male* [mm].** Length of body (including cerci and head, excluding antennae) 89.4, length/width of head 5.5/4.5, antennae 55.8, pronotum 4.5, mesonotum 5.8, length of tegmina 27.1, length of alae 63.7, greatest width of abdomen 21.6, profemora 19.6, mesofemora 15.6, metafemora 17.6, protibiae 12.4, mesotibiae 9.7, metatibiae 13.4.

###### Etymology.

Noun, Latin for “endowed with spirit”. We felt that these incredible insects are so leaf-like in texture, color, and movement, they simply appear to be a leaf on a tree come to life and walking right out of a fantasy novel and therefore the full binomial translating to “hidden leaf endowed with spirit” was fitting.

##### Cryptophyllium athanysus

Taxon classificationAnimalia

(Westwood, 1859) comb. nov.

FD7965C1-0ABE-5CC2-9D3C-500CF043194D

Figures 11 , 12

###### Material examined.

**Figure 11.**
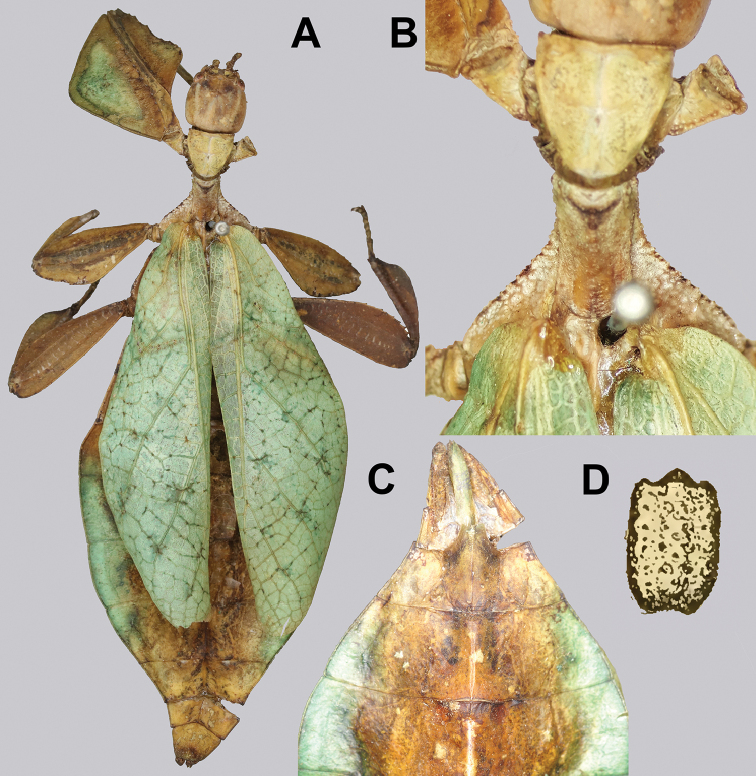
*Cryptophylliumathanysus* comb. nov. holotype female, photographs by Paul Brock (NHMUK) **A** habitus, dorsal view **B** details of the thorax, dorsal **C** genitalia details, ventral **D** illustration of a *Cryptophylliumathanysus* comb. nov. egg from Greene (1906).

**Figure 12.**
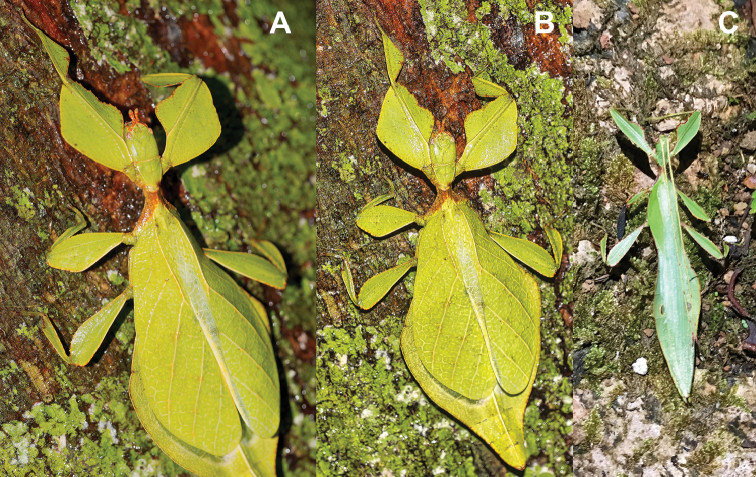
Live *Cryptophylliumathanysus* comb. nov. observed in the Sinharaja Forest Reserve, Sri Lanka **A, B** female photographed by Alberto Carrera (**A** photograph purchased from Alamy photo stock website **B** photograph purchased from Adobe Stock) **C** first record of a live male observed and photographed by Angela Christine Chua (Singapore) in January 2020.

Very few specimens are known. We examined the holotype female in the NHMUK “Holo-type. Ceylon. *Athanysus*, Westw. *PhylliumAthanysus* Westw, Ceylon. BMNH(E) #845230” (Fig. 11A) and a male in the OUMNH collection “P. athanysus, Westw. Ceylon. E Coll. (1830–73), W. W. Saunders., Purchased and, pres. ‘73 by Mrs. F. W. Hope.” Greene (1906: fig. 1b, plate between page 220 and 221) illustrated a single female specimen as well as an egg (fig. 2b, page 221). We were unable to trace these specimens for proper examination. Besides these few specimens, we reviewed images of one live female (Fig. 12A, B) and one live male (Fig. 12C) from the Sinharaja Forest Reserve, but no other records> are known to us.

###### Remarks.

This rare species has only had a number of instances over the years where preserved specimens were illustrated. Here we present the first known photographs of live male and female individuals observed on two different occasions, both within the Sinharaja Forest Reserve, Sri Lanka (Fig. 12).

###### Differentiation.

Females can be differentiated by the smaller size (the smallest known species of *Cryptophyllium* gen. nov., ca. 77.0 mm) and the fully developed exterior metatibial lobes, which span the entire metatibial shaft, and the weak but notable full mesotibial exterior lobes, which also span the entire length of the shaft (when there are exterior lobes present in the other *Cryptophyllium* gen. nov. species, they are small and restricted to the distal end of the shaft only). *Cryptophylliumathanysus* comb. nov. females are most morphologically similar to *Cryptophylliumbollensi* sp. nov. and to *Cryptophylliumchrisangi* comb. nov. due to the anteriorly narrow mesopleura and the broad rounded profemoral exterior lobes. Additionally, *Cryptophylliumchrisangi* comb. nov. also has a tapered abdomen like *Cryptophylliumathanysus* comb. nov. without distinct abdominal lobes on segment VII, which many *Cryptophyllium* species have. Both of these morphologically similar species can immediately be differentiated however by the lack of metatibial exterior lobes.

Males can also be differentiated from all other species by the presence of fully developed exterior metatibial lobes which span the entire metatibial shaft, as no other *Cryptophyllium* gen. nov. species are known to have such a feature. Male *Cryptophylliumathanysus* comb. nov. are morphologically very similar to *Cryptophylliumwestwoodii* comb. nov. as they both have similar narrow abdominal shapes, similar profemoral morphology, and similar thorax shape and serration. *Cryptophylliumathanysus* comb. nov. can be differentiated easily however by the presence of metatibial exterior lobes, which *Cryptophylliumwestwoodii* comb. nov. lacks.

###### Distribution.

Sri Lanka. All old records> simply state “Ceylon’’ as the location, but the two modern records> we have come across were both from within the Sinharaja Forest Reserve on the southern end of the island (Fig. 12). Even Greene (1906) regarded this species as rarely encountered with most records> belonging to the other phylliid species present on the island.

##### Cryptophyllium bankoi

Taxon classificationAnimalia

gen. et sp. nov.

803CD1D0-8BC6-5EBB-869D-05E0B758D8B4

http://zoobank.org/A99E5AA5-D9A3-405B-92F7-DE8B83A57384

**Figure 13.**
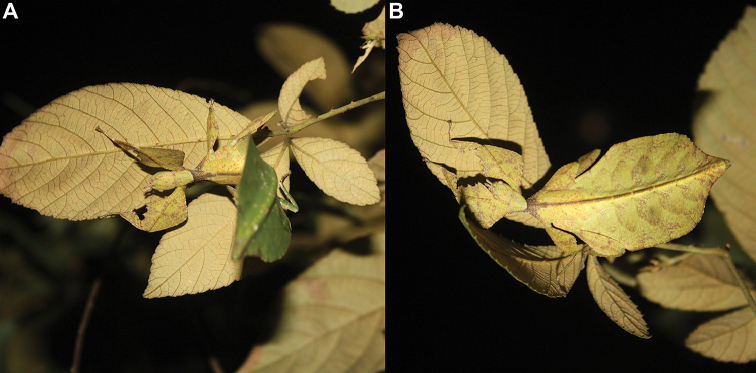
*Cryptophylliumbankoi* gen. et sp. nov. paratype female nymph collected in Bach Ma N.P. on *Rubus* sp. (Rosaceae), Vietnam, photographed by Jérôme Constant (RBINS) on 11^th^ July 2011. Molecular sample RBINS06 within this study **A** dorsal view with abdomen curled **B** dorsal view with abdomen held flat.

**Figure 14.**
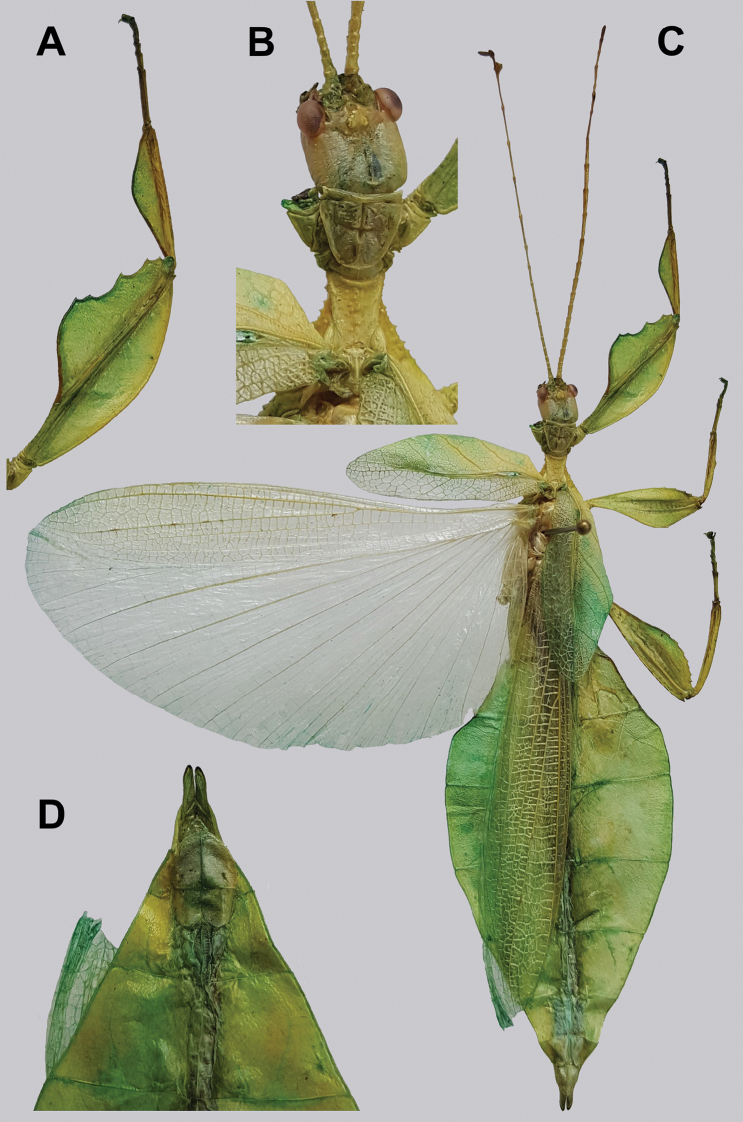
*Cryptophylliumbankoi* gen. et sp. nov. holotype male, photographs by RTC **A** front leg details, dorsal **B** details of the base of antennae, head, thorax **C** habitus, dorsal **D** details of genitalia, ventral.

**Figure 15.**
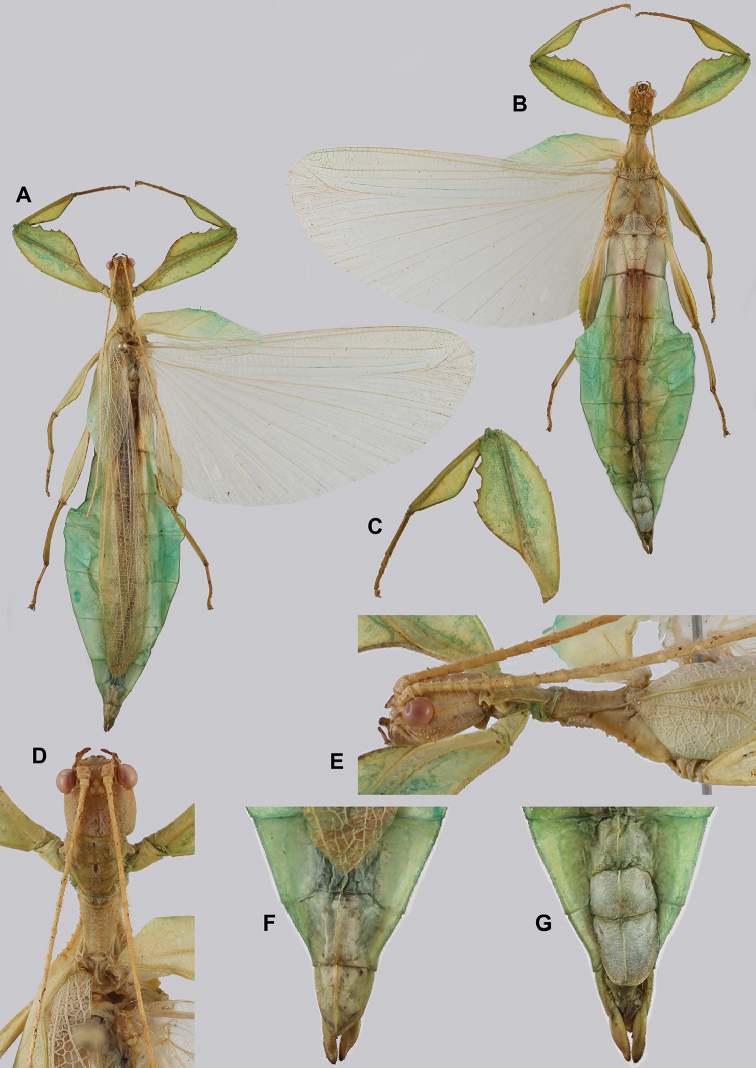
*Cryptophylliumbankoi* gen. et sp. nov. paratype male from the Ba Na-Nui Chua Nature Reserve (RBINS), photographs by Jérôme Constant (RBINS) **A** habitus, dorsal **B** habitus, ventral **C** pro- femoral and tibial, dorsal **D** details of the base of antennae, head, and thorax, dorsal **E** details of the base of antennae, head, and thorax, lateral **F** terminalia, dorsal **G** genitalia, ventral.

Figures 5G , 13 , 14 , 15

###### Material examined.

***Holotype*** ♂: “VIETNAM: Quang Nam, Tay Giang, Axan Mt, 1,300 meters: June 2017, Coll RC 17-228”. Deposited in the Montreal Insectarium (IMQC).

***Paratypes***: (4 ♂♂, 1 ♂ nymph, 1 ♀ nymph) • 1 ♂; “Vietnam; Daknong, June, 2017, Coll RC 17-338” (Coll RC) • 1 ♂; “Vietnam; Quang Ngai Province, Bato Mt. 900 m. elv: May 2015, Coll RC 16-141” (Coll RC) • 1 ♂: “Coll. I.R.Sc.N.B., Da Nang prov., Ba Na-Nui Chua Nat. Res., 18°09’N 105°55’E, 16-19.vii.2017, GTI Project, Leg. J. Constant and J. Bresseel, I.G.: 33.498” [vomer dissected] (RBINS) • 1 ♂: “Ngoc Linh, Kon Tum prov., Vietnam, 1700 m, VI.2016, leg. Luong coll. TB-05-134” (Coll TB) • 1 subadult ♀: “C. Vietnam, Bach Ma N.P., 16°12’N 107°52’E, 12-17.vii.2011, Leg J. Constant and J. Bresseel, I.G.: 31.933” [RBINS- Phyllium-DNA sample 0006] (RBINS) • 1 subadult ♂: “Vietnam, Gia Lai prov. Kon Chu Rang N.R., 600-1200 m, 13-20.vii.2018, GTI project, 14°28’28”N 108°32’27”E, Leg. J. Constant, J. Bresseel and X. Vermeersch, I.G.:33.769” [RBINS- Phyllium-DNA sample 0008] (RBINS).

###### Remarks.

This species, in true “cryptic leaf” fashion, was only recognized as unique when the molecular results were reviewed as morphologically it was hidden within the *Cryptophylliumrarum* comb. nov. males. Their morphological resemblance is uncanny and even with a series of males it is difficult to adequately differentiate these two species based on male morphology. Only the male *Cryptophylliumbankoi* sp. nov. is known at present and therefore it is unknown if the female also morphologically resembles *Cryptophylliumrarum* comb. nov. or is morphologically more unique. Despite several expeditions to southern Vietnam by the RBINS expedition members, only one nymph female *Cryptophylliumbankoi* sp. nov. has been collected to date, unfortunately she did not survive to adulthood (Fig. 13). With this species recorded from three Vietnamese provinces, we hope that as a widely ranging species the female can one day be identified and morphologically described.

###### Differentiation.

Females unknown. Males are morphologically very similar to *Cryptophylliumrarum* comb. nov. in their antennae length, profemoral interior lobe serration, protibial interior lobe shape, tegmina length, and their thorax shape and spination. The molecular results revealed however that this species was in fact not identical to *Cryptophylliumrarum* comb. nov. with which it shares a sympatric geographic range in central Vietnam and a striking morphological resemblance. Thankfully with the molecular analysis we were able to separate out these two species to allow us to see the subtle differences between them, which we originally thought to be simple morphological variation. The only consistent morphological differences we were able to locate between these species is that *Cryptophylliumrarum* comb. nov. tends to have an abdomen which is slightly more rounded (Fig. 59A), vs. *Cryptophylliumbankoi* sp. nov. which has the abdomen ever so slightly spade-shaped (Fig. 14). Additionally, it appears as though *Cryptophylliumrarum* comb. nov. tend to be larger (79.8–89.0 mm) vs. *Cryptophylliumbankoi* sp. nov. (61.9–69.5 mm), and the profemoral exterior lobes have slightly differing serration, with *Cryptophylliumbankoi* sp. nov. having no teeth or up to five small teeth, vs. *Cryptophylliumrarum* comb. nov., which tend to have seven or eight teeth.

###### Distribution.

Central and Southern Vietnam. Known from Quang Ngai, Thua Thien Hue, Da Nang, Gia Lai, Quang Nam, and Dak Nong Provinces.

###### Male.

***Coloration.*** Living individuals are a more vibrant green, and our descriptions are only based on preserved specimens. Overall coloration pale green throughout with variable patches of yellow and tan coloration (Figs 14, 15). Compound eyes are slightly reddish to pink (Figs 14B, 15D). The antennae are yellow to orange in color. In all specimens examined there were no variable brown patches on the lobes of the legs as is sometimes common in male phylliids.

***Morphology.****Head.* Head capsule approximately as long as wide, with a vertex that is irregularly lumpy and with small granulation throughout, and a posteromedial tubercle which is larger than any of the nodes on the head (Fig. 14B). Frontal convexity stout with sparse thin setae throughout the surface. Compound eyes large and bulbous, occupying ca. ⅖ of the head capsule lateral margins (Fig. 14B). Between the compound eyes are three well-developed ocelli (Fig. 14B). Antennal fields approximately as wide and as long as the scapus. *Antennae.* Antennae (including the scapus and pedicellus) consists of 25 or 26 segments, all segments except the scapus and pedicellus and terminal four segments are covered in dense setae that are as long as or longer than the antennae segment is wide. The terminal four segments are covered in dense short setae and the scapus and pedicellus are nearly bare, lacking setae. *Thorax.* Pronotum with anterior margin slightly concave and lateral margins that are straight and converging to a straight posterior margin that is ca. ½ as wide as the anterior margin (Fig. 14B). Anterior and lateral margins of the pronotum have distinct rims, but the posterior margin has a weakly formed rim (Fig. 14B). Face of the pronotum is marked by a distinct pit in the center with prominent anterior and lateral furrows from this central pit, and the pronotum surface is marked with wrinkles throughout, but not prominent nodes (Fig. 14B). Prosternum surface is moderately granulose throughout, the mesosternum surface is mostly smooth with only small granulation along the sagittal plane which then continues onto the metasternum surface which has moderate granulation throughout (Fig. 15B). Prescutum ca. ⅓ longer than the anterior margin width, with lateral margins slightly converging to the posterior (Fig. 14B). Lateral margins lacking distinct tubercles, instead with granulation throughout giving them a rough appearance (Fig. 14B). Prescutum surface throughout with granulation, with smaller and tighter packed granulation along the lateral margins, and slightly larger nodes along the sagittal plane (Fig. 14B). Prescutum anterior margin distinct but not significantly raised, with a surface that is marked with minimal granulation. Mesopleura not notably wide, narrow near the anterior margin and then only gradually diverge for the remainder of their length (Fig. 14B). Lateral margin with five or six small tubercles throughout the length and several smaller minor tubercles interspersed throughout. Face of the mesopleura slightly wrinkled and with two faint divots, one near the anterior margin and one on the posterior ⅓ (Fig. 15E). *Wings.* Tegmina moderate length, extending ⅓ to ½ through abdominal segment III. Tegmina wing venation: the subcosta (Sc) is the first vein, is simple, and terminates the earliest ca. ⅓ of the way through the overall tegmina length. The radius (R) spans the entire length of the tegmina with the first radius (R1) branching ca. ⅖ of the way through the wing length and terminating near the midline, followed by the branching and termination of the second radius (R2) near the distal ⅓ of the wing, and the radial sector runs to the wing apex. The media (M) also spans the entire length of the tegmina with the first media posterior (MP1) branching off ca. ⅓ of the way through the wing length, the second media posterior (MP2) branching near the midline, and the media anterior (MA) running to the wing apex. The cubitus (Cu) runs along the edge of the wing as the two media posterior veins fuse with it and as the cubitus reaches the apex it fades. The first anal (1A) vein terminates upon reaching the cubitus ca. ⅓ of the way through the wing length. Alae well developed in an oval fan configuration, long, reaching to the middle of abdominal segments VIII. Alae wing venation: the costa (C) is present along the entire foremargin giving stability to the wing. The subcosta (Sc) is long, spanning ca. ⅔ of the wing length and is mostly fused with the radius in the beginning but terminates when it meets the costa. The radius (R) spans the entire wing and branches ca. ⅓ of the way through into the first radius (R1) and radial sector (Rs) which run gently diverging for most of their length and then converge at the apex of the wing where they terminate near each other but not touching. The media (M) branches early, ca. ⅙ of the way through the wing into the media anterior (MA) and the media posterior (MP) which run parallel with each other throughout the wing until the distal ⅕ of the wing when the media posterior fuses with the media anterior which then run fused together to the wing apex where they terminate near the radial sector. The cubitus (Cu) runs unbranched and terminates at the wing apex. Of the anterior anal veins, the first anterior anal (1AA) fuses with the cubitus near the point where the media branches into the media anterior and media posterior and then the first anterior anal branches from the cubitus ⅔ of the way through the wing length where it uniformly diverges from the cubitus until it terminates at the wing margin. The anterior anal veins two–seven (2AA–7AA) have a common origin and run unbranched in a folding fan pattern of relatively uniform spacing to the wing margin. The posterior anal veins (1PA–6PA) share a common origin separate from the anterior anal veins and run unbranched to the wing margin with slightly thinner spacing than the anterior anal veins. *Abdomen.* Abdominal segment II with parallel margins, III through the anterior ⅓ of segment IV gradually diverging to the widest portion. The posterior of IV–V are parallel or slightly subparallel. Segments VI–X uniformly converging, giving the abdomen a spade-shaped appearance (Fig. 14C). Abdominal segment X distinctly longer than wide (Fig. 15F). *Genitalia.* Poculum broad, and ending in a rounded apex that slightly passes the anterior margin of segment X (Fig. 14D). Cerci long and slender, extending from under the anal abdominal segment by slightly > half of their length, surface slightly cupped and with a granulose surface with numerous short setae throughout (Fig. 14D). Vomer broad and stout with straight sides evenly converging to the apex, which is marked by two apical hooks, one slightly larger than the other (Fig. 5G). *Legs.* Profemoral exterior lobe slightly thinner than the interior lobe, arcing end to end in a smoothly bending lobe which has a granular margin throughout and can range from lacking dentation to having four or five small serrate teeth on the distal half of the lobe (Figs 14A, 15C). Profemoral interior lobe roundly triangular and marked with five teeth arranged in a two-one-two pattern with large looping gaps between these groups (Fig. 15A). Mesofemoral exterior lobe arcs end to end, but is more heavily weighted toward the distal half which is slightly broader than the mesofemoral shaft width, and which is marked with 1–3 serrate teeth on the distal half only, and the proximal half is rather thin and lacks dentation. Mesofemoral interior lobe is about the same width as the mesofemoral shaft and arcs end to end, is slightly broader on the distal end and is marked with four or five small serrate teeth. Metafemoral exterior lobe lacks dentation, and has a straight margin hugging the metafemoral shaft. Metafemoral interior lobe smoothly arcs end to end with seven or eight serrate teeth on the distal half only. Protibiae lacking exterior lobe, interior lobe reaching end to end in a smoothly rounded triangle which is slightly weighted to the distal half and at the widest point slightly < 2× the protibial shaft width (Fig. 15A). Meso- and metatibiae simple, lacking lobes completely.

***Measurements of holotype male* [mm].** Length of body (including cerci and head, excluding antennae) 64.3, length/width of head 3.8/3.6, antennae 34.5, pronotum 2.9, mesonotum 3.7, length of tegmina 17.4, length of alae 47.0, greatest width of abdomen 20.6, profemora 13.7, mesofemora 10.6, metafemora 12.5, protibiae 8.2, mesotibiae 7.0, metatibiae 9.4.

***Measurements of paratype males* [mm].** Length of body (including cerci and head, excluding antennae) 61.9–69.5, length/width of head 3.2–3.6/3.1–3.7, antennae 32.8–41.2, pronotum 2.7–3.6, mesonotum 4.0–5.1, length of tegmina 19.7–19.9, length of alae 48.3–50.1, greatest width of abdomen 18.4–20.6, profemora 12.5–15.7, mesofemora 9.9–12.1, metafemora 10.8–14.2, protibiae 7.6–9.2, mesotibiae 7.8–8.2, metatibiae 8.0–10.4.

###### Etymology.

Patronym. This species is dedicated to our friend and colleague Alexandre Banko for his extensive efforts to discover new species and his long collaboration with Team Phyllies to present us with fresh material to sequence and study.

##### Cryptophyllium bollensi

Taxon classificationAnimalia

gen. et sp. nov.

74B70D7C-2163-5BE1-9FE3-D1049C04008F

http://zoobank.org/8023142D-4BAA-4FBF-A26F-B280DC7C5E02

Figures 5E , 6B , 8E , 8F , 9H , 16 , 17 , 18 , 19 , 20 , 21

###### Material examined.

***Holotype*** ♂: “Coll. I.R.Sc.N.B., Ex breeding Tim Bollens, 2018, Coll. I.R.Sc.N.B., Vietnam, Ninh Thuan prov., Phuoc Binh N.P., 12°04’N, 108°45’E, 26.vii.2014, night coll., Leg.: J. Constant & J. Bresseel, GTI project, IG: 32.779”. Deposited in the Royal Belgian Institute of Natural Sciences (RBINS).

***Paratypes***: (16 ♀♀, 25 ♂♂, 46 eggs) • 1 ♀: “Coll. I.R.Sc.N.B., Vietnam, Ninh Thuan prov., Phuoc Binh N.P., 12°04’N, 108°45’E, 26.vii.2014, night coll., Leg.: J. Constant and J. Bresseel, GTI project, IG: 32.779” (RBINS) • 6 ♂♂: same data as HT [3 with vomer dissected] (RBINS) • 2 ♂♂, 3 ♀♀, 1 subadult ♂: “Coll. I.R.Sc.N.B., Ex breeding Tim Bollens, 2019, Vietnam, Ninh Thuan prov., Phuoc Binh N.P., 12°04’N, 108°45’E, 26.vii.2014, night coll., Leg.: J. Constant and J. Bresseel, GTI project, IG: 32.779” (RBINS) • 1 ♂: “Coll. I.R.Sc.N.B., Ex breeding B. Kneubuhler, Vietnam, Ninh Thuan prov., Phuoc Binh N.P., 12°04’N, 108°45’E, 26.vii.2014, night coll., Leg.: J. Constant and J. Bresseel, GTI project, IG: 32.779” (RBINS) • 1 ♂, 1 ♀: “Coll. I.R.Sc.N.B., Ex breeding Bruno Kneubühler, 2017” “Coll. I.R.Sc.N.B., Vietnam, Ninh Thuan prov., Phuoc Binh N.P., 12°04’N, 108°45’E, 26.vii.2014, night coll., Leg.: J. Constant and J. Bresseel, GTI project, IG: 32.779” (VNMN) • 1 ♀; “VIETNAM: Ninh Thuan prov. Phuoc Binh N.P., 12°04’N 108°45’E, Bred from eggs supplied by Bruno Kneubühler (Switzerland), May, 2018, Coll RC 18-414” (Coll RC) • 2 subadult ♀♀; “VIETNAM: Ninh Thuan prov. Phuoc Binh N.P., 12°04’N 108°45’E, Bred from eggs supplied by Bruno Kneubühler (Switzerland), May, 2018” Coll RC 18-412 and 18-413 (Coll RC) • 1 ♂; “VIETNAM: Ninh Thuan prov. Phuoc Binh N.P., 12°04’N 108°45’E, Bred from eggs supplied by Bruno Kneubühler (Switzerland), April, 2018, Coll RC 18-2017” (Coll RC) • 19 eggs; “VIETNAM: Ninh Thuan prov. Phuoc Binh N.P., 12°04’N 108°45’E, eggs supplied by Bruno Kneubühler (Switzerland), 2018”, Coll RC 17-375, 17-376, 18-282–18-298 (Coll RC) • 1 ♀, 3 ♂♂, 27 eggs; “ex Zucht T. Bollens 2018, Herkunft: Vietnam, Prov. Nin Thuan, Bác Ái Distr., Phuoc Binh N.P., leg. Bresseel and Constant 2014” [coll. FH, No’s 1061-1 to 4, E], (Coll FH) • 1 ♀, 1 ♂ (nymph n3); “ex Zucht F. Hennemann 2019, Herkunft: Vietnam, Prov. Nin Thuan, Bác Ái Distr., Phuoc Binh NP, leg. Bresseel and Constant 2014” [coll. FH, No’s 1061-5 and 6], (Coll FH) • 2 ♀♀, 3 ♂♂; “VIETNAM: Ninh Thuan prov. Phuoc Binh N.P., 12°04’N 108°45’E, Bred by Maxime Ortiz, France, circa 2020” (Coll MO) • 4 ♀♀, 6 ♂♂; “VIETNAM: Ninh Thuan prov. Phuoc Binh N.P., bred by Bruno Kneubühler (Switzerland), circa 2017-2018” (Coll OC).

###### Remarks.

**Figure 16.**
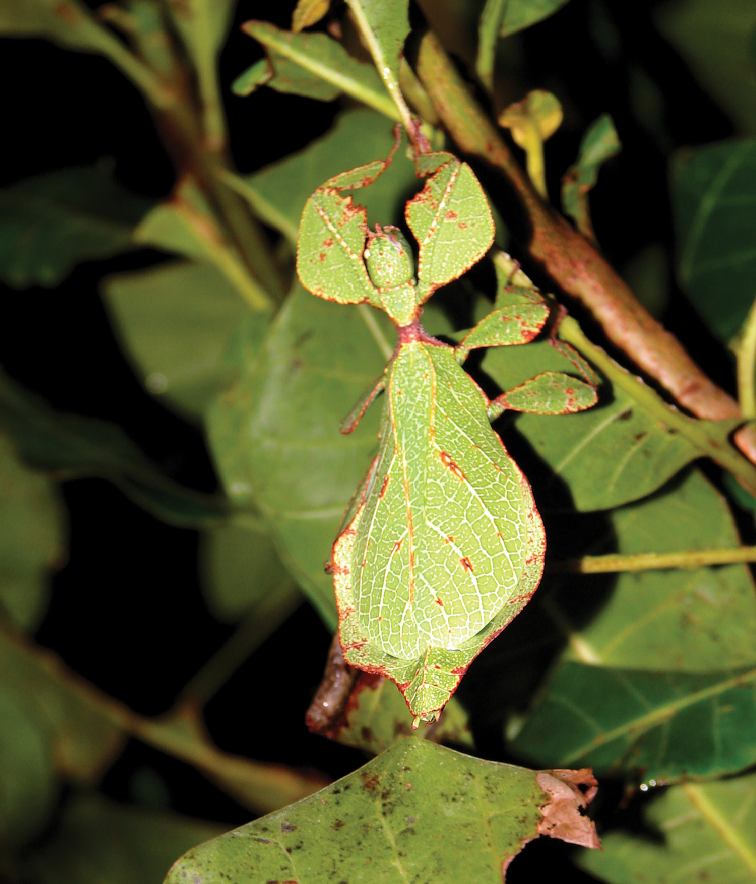
Live *Cryptophylliumbollensi* gen. et sp. nov. paratype where she was found in Phuoc Binh N.P., Vietnam in July 2014 by Joachim Bresseel (RBINS) and Jérôme Constant (RBINS). Photograph by Jérôme Constant (RBINS).

**Figure 17.**
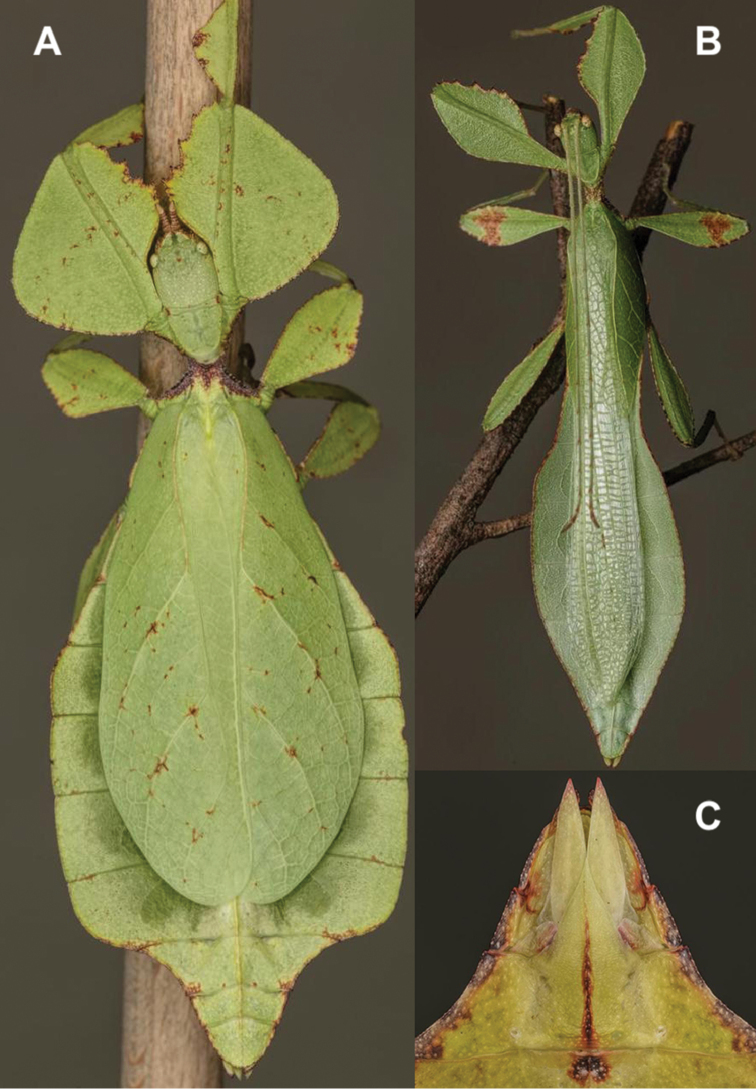
Live *Cryptophylliumbollensi* gen. et sp. nov. bred and photographed by Bruno Kneubühler (Switzerland) **A** adult female, dorsal **B** adult male, dorsal **C** genitalia, female, ventral.

This species was first collected by Jérôme Constant (RBINS) and Joachim Bresseel (RBINS) during a GTI research expedition. Only a single adult female was found (Fig. 16) while collecting at night in Phuoc Binh N.P.. The female was found on a shrub in a field that had been cleared of trees and planted with crops. The clearing of the forest had been done fairly recently as tree stumps were still present. In this area there was no adjacent old growth forest, only secondary forest nearby. The female was kept alive long enough to lay a series of fertilized eggs which were shared with expert phylliid breeder Tim Bollens (Belgium) who was able to rear a nice series of specimens and eventually share this species with other leaf insect breeder enthusiasts (Fig. 17).

###### Differentiation.

Females are morphologically most similar to *Cryptophylliumphami* sp. nov., *Cryptophylliumchrisangi* comb. nov., and *Cryptophylliumnuichuaense* sp. nov. based on the general femoral lobe shapes, the broad rounded exterior profemoral lobe, and the thorax shape and spination. *Cryptophylliumbollensi* sp. nov. have moderately long alae reaching onto abdominal segment IV that can be differentiated from *Cryptophylliumphami* sp. nov., which have shorter alae only reaching the anterior margin of abdominal segment III. The ventral surface of the antennae differentiates *Cryptophylliumbollensi* sp. nov. from the other similar species as *Cryptophylliumbollensi* sp. nov. have the ventral surface of segments VI, VII, and VIII flush (Fig. 6B) vs. *Cryptophylliumchrisangi* comb. nov. and *Cryptophylliumnuichuaense* sp. nov. which have the ventral surface of antennal segments VI and VII projecting beyond segment VIII, giving the antennae a slight lamellate appearance (Fig. 6E).

Males are morphologically most similar to *Cryptophylliumwestwoodii* comb. nov., *Cryptophylliumchrisangi* comb. nov., *Cryptophylliumphami* sp. nov., and *Cryptophylliumkhmer* sp. nov. due to the femoral shape and spination, the length of antennae and alae, and thorax shape and spination. *Cryptophylliumwestwoodii* comb. nov. and *Cryptophylliumchrisangi* comb. nov. can be differentiated by their narrower abdominal shape with a maximum width only 30–34% of the abdominal length, vs. the others which have an abdominal shape that is broadly elliptical or broadly spade-shaped with a maximum width ca. 38–45% of the abdominal length. Due to similarities in morphology and the intraspecific variation within *Cryptophylliumbollensi* sp. nov., *Cryptophylliumphami* sp. nov., and *Cryptophylliumkhmer* sp. nov., we could not identify a reliable morphological feature for differentiation within the males. The female morphology does allow differentiation of these species, and of course molecular analysis (Fig. 4) allows reliable differentiation even between these variable and difficult to distinguish species.

###### Distribution.

At present only known from the type locality of Phuoc Binh N.P., Ninh Thuan Province, southern Vietnam.

###### Description.

**Female. *Coloration.*** Coloration description is based upon living individuals (Figs 16, 17A). Overall coloration is pale green throughout, with variable areas highlighted with burnt red or brown coloration. These areas tend to be the margins on the lobes of the legs, some striping on the lobes of the legs, the thorax, abdominal margins, and the venation in the tegmina (Fig. 16).

**Figure 18.**
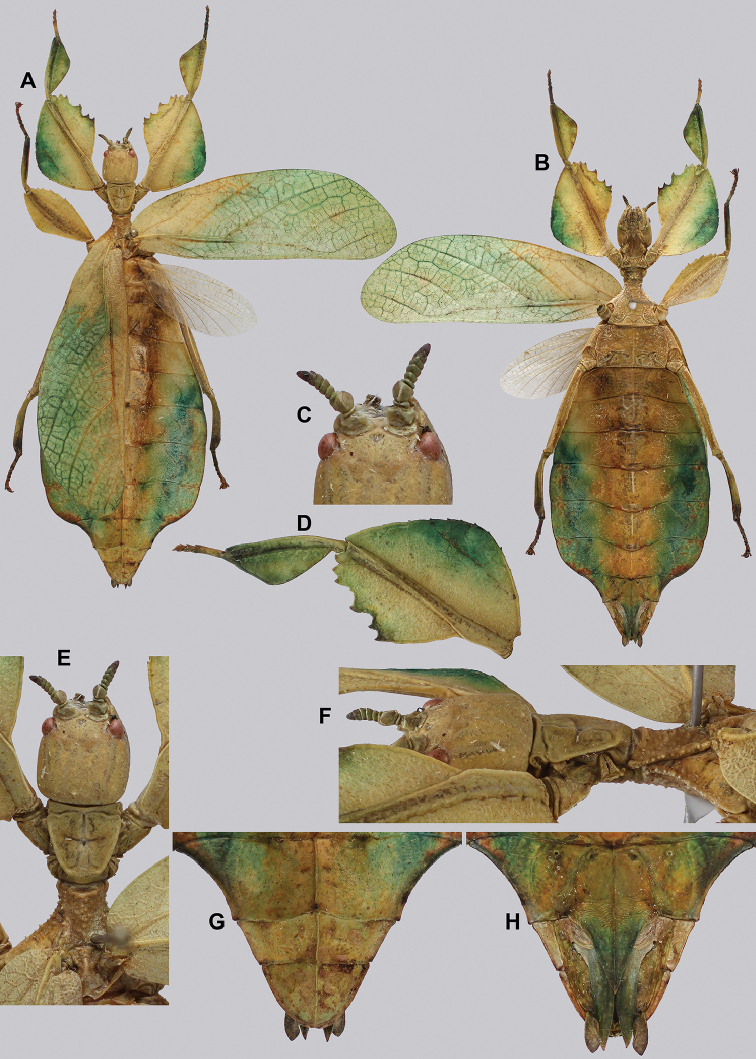
*Cryptophylliumbollensi* gen. et sp. nov. paratype female, photographs by Jérôme Constant (RBINS) **A** habitus, dorsal **B** habitus, ventral **C** details of the antennae, dorsal **D** front leg details, dorsal **E** details of the antennae, head, and thorax, dorsal **F** details of the antennae, head, and thorax, lateral **G** terminalia, dorsal **H** genitalia, ventral.

***Morphology.****Head.* Head capsule about as long as wide, vertex relatively smooth with the only notable feature being the posteromedial tubercle which is finely pointed (Fig. 18E). Frontal convexity broad and blunt, with a slightly granular surface. Compound eyes slightly protruding from the head capsule, and are not particularly large, taking up ca. ¼ of the head capsule lateral margins (Fig. 18E). Ocelli absent. Antennal fields slightly wider than the width of the first antennomere. *Antennae.* Antennae consist of nine segments, with the terminal segment about the same length as the preceding 2½ segments’ lengths combined (Fig. 6B). Antennomeres I–VIII sparsely marked with small transparent setae, the terminal antennomere and the anterior margin of antennomere VIII are covered in stout, brown setae (Fig. 18C). *Thorax.* Pronotum with gently concave anterior margin and slightly convex lateral margins, which converge to a straight posterior margin that is half the width of the anterior margin (Fig. 18E). The pronotum surface is smooth, with only a prominent pit in the center, and slight furrows anterior and lateral to the pit (Fig. 18E). The pronotum has moderately formed anterior and lateral rims and a weakly formed posterior rim, all of which are relatively smooth (Fig. 18E). Prosternum and the mesosternum are covered with numerous broad nodes, but the metasternum has a somewhat wrinkled surface. Prescutum longer than wide, lateral rims with 9–11 small to medium tubercles, similar in size giving the margin a rough appearance (Fig. 18E). Prescutum anterior rim prominent but not strongly protruding, rim surface is granular, lacking a large sagittal spine (Fig. 18F). Prescutum surface heavily granular, with those along the sagittal plane slightly larger than the rest (Fig. 18E). Mesopleura begin ca. ¼ of the way through the prescutum length and evenly diverge; lateral margin with nine or ten small tubercles with about half of those slightly larger than the rest, with the smaller ones interspersed throughout (Fig. 18E). Face of the mesopleura smooth or slightly wrinkled, with two notable divots, one on the anterior margin and one near the middle (Fig. 18F). *Wings.* Tegmina long, reaching ½ through abdominal segment VII. Tegmina venation; the subcosta (Sc) is the first vein in the forewing, running parallel with the margin for the first half, and then bending and running towards the margin. The radius (R) spans the central portion of the forewing with two subparallel branched veins; the first radius (R1) branches ca. ¼ of the way through the wing length and terminates slightly proximal to the midline, and the radial sector (Rs) branches ca. ⅖ of the way through the wing length and terminates near the distal ⅓ of the wing length. There is a weak continuation of the radius following the prominent Rs branching which continues on as a short and thin R–M crossvein that weakly connects the two veins. The media (M) is simply bifurcate with both the media anterior (MA) and media posterior (MP) terminating near to the posterior ¼ of the wing. The cubitus (Cu) is also bifurcate, branching near the posterior ⅕ of the wing into the cubitus anterior (CuA) and cubitus posterior (CuP) which both terminate at or very near the wing posterior apex. The first anal vein (1A) is simple and fuses with the cubitus early on, at the length about midway between the splitting of the R1 and Rs. Alae short, with their apex only just passing the anterior margin of abdominal segment IV. *Abdomen.* Abdominal segments II through the anterior half of IV uniformly diverging. The posterior half of segment IV through the anterior of segment VII are parallel, giving the abdomen a boxy appearance. The posterior half of segment VII ends in a slightly rounded lobe. Segments VIII–X are notably narrower than the previous segments, and have converging margins to the broad rounded apex (Fig. 18G). *Genitalia.* Subgenital plate starts at the anterior margin of tergum VIII, is moderately broad, and extends halfway onto tergum X with straight margins ending in a fine point (Fig. 18H). Gonapophyses VIII are long and moderately broad, slightly exceeding the apex of abdominal tergum X; gonapophyses IX are shorter and narrower, hidden below (Figs 17C, 18H). Cerci flat, not strongly cupped, with a granular surface and few detectable setae (Fig. 18H). *Legs.* Profemoral exterior lobe broad, rounded, and obtusely angled, smoothly arcing from end to end, ca. ⅓ wider than the width of the interior lobe (Fig. 18D). Edge of the profemoral exterior lobe granular, or with a slightly serrate surface of eight or nine small teeth (Fig. 18D). Profemoral interior lobe ca. 2× as wide as the greatest width of the profemoral shaft, obtusely angled, and marked with five teeth arranged in a two-one-two pattern with looping gaps between them (Fig. 18D). Mesofemoral exterior lobe arcs from end to end but is slightly bent in the center, weighted towards the distal half, and marked with three or four small serrate teeth distributed on the distal half only. Interior lobe is about the same width as the mesofemoral shaft, and the exterior lobe is slightly wider. Mesofemoral interior lobe arcs smoothly end to end with 6–8 small serrate teeth only on the distal half of the arc which is slightly wider than the proximal half of the arc. Metafemoral interior lobe arcs end to end, with the distal half slightly wider than the proximal half and marked with 7–10 serrate teeth on the distal half of the lobe. Metafemoral exterior lobe is thin and smooth, hugging the metafemoral shaft and lacks dentation. Protibiae lacking an exterior lobe (Fig. 18D). Protibiae interior lobe spans the entire length of the protibiae and is ca. 2× the width of the protibiae shaft itself. The lobe is roundly triangular with the widest portion on the distal half. Mesotibiae and metatibiae lacking exterior and interior lobes.

***Measurements of paratype female* [mm] (wild caught).** Length of body (including cerci and head, excluding antennae) 75.6, length/width of head 8.0/6.1, antennae 4.3, pronotum 4.8, mesonotum 7.2, length of tegmina 45.3, length of alae 23.1, greatest width of abdomen 28.6, profemora 16.7, mesofemora 13.6, metafemora 16.3, protibiae 10.8, mesotibiae 9.4, metatibiae 12.6.

***Measurements of paratype females* [mm] (ex culture).** Length of body (including cerci and head, excluding antennae) 74.6–80.1, length/width of head 7.6–8.5/5.9–6.2, antennae 3.9–4.2, pronotum 4.5–5.4, mesonotum 6.7–6.8, length of tegmina 43.2–45.1, length of alae (not measurable as they were hidden by the tegmina), greatest width of abdomen 29.7, profemora 17.2–18.2, mesofemora 13.7–14.4, metafemora 16.5–16.6, protibiae 10.9–11.0, mesotibiae 9.1–9.6, metatibiae 12.5–12.8.

**Male. *Coloration.*** Coloration based upon live bred specimens in captivity (Fig. 17B). Overall coloration pale green throughout with variable patches of tan to reddish coloration (Fig. 17B). These tan to reddish areas are primarily around the margins of the lobes of the legs, the margins of the thorax, the tips of the antennae, and the margins of the abdomen. In darker colored specimens the mesofemoral lobes can also have coloration, not just along the margins. Abdominal segment V has a pair of slightly transparent eye spots.

**Figure 19.**
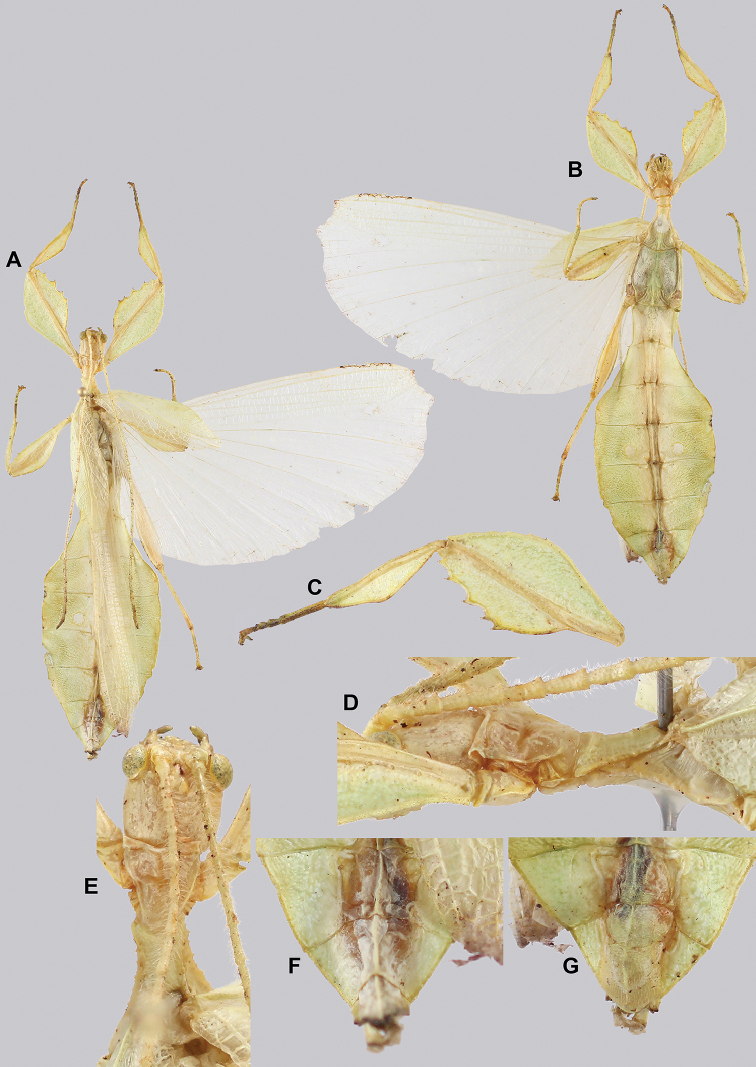
*Cryptophylliumbollensi* gen. et sp. nov. holotype male, photographs by Jérôme Constant (RBINS) **A** habitus, dorsal **B** habitus, ventral **C** pro- tibial and femoral lobes, dorsal **D** details of the base of the antennae, head, and thorax, lateral **E** details of the antennae, head, and thorax, dorsal **F** terminalia, dorsal **G** genitalia, ventral.

**Figure 20.**
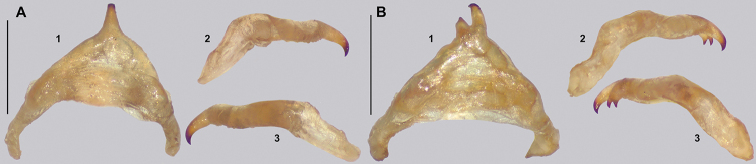
Abnormal male vomer found in *Cryptophylliumbollensi* gen. et sp. nov. from Vietnam, Phuoc Binh, observed in the RBINS collection bred from the original limited stock. Prepared and photographed by Jérôme Constant (RBINS). Scale bars, 1.0 mm. Views **1** ventral **2** right lateral of the ventrally oriented vomer **3** left lateral of the ventrally oriented vomer **A** abnormal singularly hooked vomer **B** abnormal three hooked vomer.

***Morphology.****Head.* Head capsule about as long as wide, with a vertex that is relatively smooth with only light granulation throughout. Frontal convexity stout with sparse thin setae. The posteromedial tubercle is not broad but is distinctly raised from the head capsule. Compound eyes large and bulbous, taking up ca. ⅖ of the head capsule lateral margins (Fig. 19E). There are three well-developed ocelli located between and slightly posterior to the compound eyes. *Antennae.* Antennae (including the scapus and pedicellus) consist of 23–26 segments, all segments except the scapus and pedicellus and terminal three segments are covered in dense setae that are as long as or longer than the antennae segment is wide. The terminal three segments are covered in dense short setae and the scapus and pedicellus are nearly completely bare. *Thorax.* Pronotum with anterior margin slightly concave and lateral margins that are slightly convex and converging to a straight posterior margin that is ca. ½ the width of the anterior rim (Fig. 19E). Anterior and lateral margins of the pronotum have moderately formed rims and the posterior margin lacks a rim (Fig. 19D). Face of the pronotum is marked by a distinct furrow and pit in the center and a relatively smooth lumpy surface with weak granulation (Fig. 19D). Prosternum surface is weakly granular with small nodes of even size and spacing. Mesosternum surface marked with slightly more prominent nodes, with the largest along the sagittal plane and denser on the anterior margin, posterior margin with less prominent and smaller nodes. Prescutum slightly longer than wide, with lateral margins that are only slightly converging to the posterior (Fig. 19E). Lateral rims with eight or nine node-like tubercles, giving the lateral margins a rough textured appearance. Prescutum surface with minimal nodes throughout, with those along the sagittal plane slightly larger than the others. Prescutum anterior rim moderately formed but not strongly raised, with a granular surface and lacking a prominent sagittal tubercle. Mesopleura begin on the anterior prescutum margin but are narrow throughout the anterior ⅓ of their length, only diverging gently for the posterior ⅔ (Fig. 19E). Lateral margin with eight or nine minor tubercles throughout the length except for the posterior ⅓ which is relatively smooth. Face of the mesopleura mostly smooth, with slight wrinkling throughout. *Wings.* Tegmina moderate length, extending ⅓ of the way onto abdominal segment III. Tegmina wing venation: the subcosta (Sc) is the first vein, is simple, and terminates the earliest ca. ⅓ of the way through the overall tegmina length. The radius (R) spans the entire length of the tegmina with the first radius (R1) branching just proximal to the midline and terminating just distal to the midline, followed by the branching and termination of the second radius (R2) near the distal ⅓ of the wing, and then the radial sector runs to the wing apex. The media (M) also spans the entire length of the tegmina with the first media posterior (MP1) branching off slightly more than ⅓ of the way through the wing length, and then the second media posterior (MP2) branches just distal to the midline, and the media anterior (MA) runs to the wing apex. The cubitus (Cu) runs along the edge of the wing as the two media posterior veins fuse with it and as the cubitus reaches the apex it fades. The first anal (1A) vein terminates upon reaching the cubitus ca. ⅓ of the way through the wing length. Alae well developed in an oval fan configuration, long, reaching to the middle or posterior of abdominal segments IX. Alae wing venation: the costa (C) is present along the entire foremargin giving stability to the wing. The subcosta (Sc) is long, spanning ca. ⅔ of the wing length and is mostly fused with the radius in the beginning but terminates when it meets the costa. The radius (R) spans the entire wing and branches slightly proximal to the midline into the first radius (R1) and radial sector (Rs) which run gently diverging for most of their length and then converge at the apex of the wing where they terminate near each other but not touching. The media (M) branches early, ca. ⅙ of the way through the wing into the media anterior (MA) and the media posterior (MP) which run parallel with each other throughout the wing until the distal ⅕ of the wing where the media posterior fuses with the media anterior which then run fused to the wing apex where they terminate near the radial sector. The cubitus (Cu) runs unbranched and terminates at the wing apex. Of the anterior anal veins, the first anterior anal (1AA) fuses with the cubitus near the point where the media branches into the media anterior and media posterior and then the first anterior anal branches from the cubitus ⅔ of the way through the wing length where it uniformly diverges from the cubitus until it terminates at the wing margin. The anterior anal veins two–seven (2AA–7AA) have a common origin and run unbranched in a folding fan pattern of relatively uniform spacing to the wing margin. The posterior anal veins (1PA–6PA) share a common origin separate from the anterior anal veins and run unbranched to the wing margin with slightly thinner spacing than the anterior anal veins. *Abdomen.* Lateral margins of abdominal segment II parallel, III through the anterior ⅔ of segment IV gradually diverging, the remainder of IV and segment V are parallel-sided, segment VI starts parallel-sided but then gently starts to converge and the remaining segments converge uniformly to the rounded apex of the abdomen. *Genitalia.* Poculum broad and ends in a rounded apex that slightly passes the anterior margin of segment X (Fig. 19G). Cerci long and slender, extending from under the anal abdominal segment, nearly flat, not strongly cupped, covered in a granulose surface and numerous short setae (Fig. 19G). Vomer broad and stout with straight sides evenly converging and ending in a thick apical hook with a smaller second hook adjacent to it (Fig. 5E). Interestingly while examining the vomers of type material we found several aberrant vomers with some bearing only a singular hook (Fig. 20A) or even three hooks (Fig. 20B), based on other specimens examined and the trend within the *Cryptophyllium* gen. nov. males we expect that a typical male of this species has a two hooked vomer. *Legs.* Profemoral exterior lobe slightly broader than the interior lobe, ca. 2½× the greatest width of the profemoral shaft, roundly arcing end to end in a broad obtuse angle that is not distinctly bent, with the proximal margin slightly granulose, and the distal margin with four or five small serrate teeth (Fig. 20C). Profemoral interior lobe roundly triangular and marked with five sharp teeth arranged in a two-one-two pattern with looping gaps between them, and the central tooth slightly larger than the others (Fig. 20C). Mesofemoral exterior lobe arcs end to end but is slightly wider on the distal ⅓ which is marked with three or four serrate teeth, and a proximal half that is rather thin. Mesofemoral interior lobe is about the same width as the exterior, is broader on the distal end and is marked with 6–8 small serrate teeth. Metafemoral exterior lobe lacks dentation and has a straight margin along the metafemoral shaft. Metafemoral interior lobe smoothly arcs end to end with eight or nine small serrate teeth on the distal ⅔, which is slightly wider than the proximal ⅓. Protibiae lacking exterior lobe, interior lobe reaching end to end in a smooth triangle which is slightly weighted to the distal half and at its widest is ca. 2½ as wide as the protibial shaft (Fig. 20C). Meso- and metatibiae simple, lacking lobes completely.

***Measurements of holotype male* [mm].** Length of body (including cerci and head, excluding antennae) 57.6, length/width of head 4.⅓.4, antennae 41.4, pronotum 3.0, mesonotum 4.3, length of tegmina 18.3, length of alae 43.4, greatest width of abdomen 16.0, profemora 12.8, mesofemora 11.2, metafemora 13.0, protibiae 8.9, mesotibiae 7.7, metatibiae 9.5.

***Measurements of paratype males* [mm] (ex culture).** Length of body (including cerci and head, excluding antennae) 55.8–65.5, length/width of head 4.1–4.9/3.2–3.5, antennae 39.9–41.5, pronotum 3.0–3.4, mesonotum 4.0–4.8, length of tegmina 18.2–20.1, length of alae 41.5–47.2, greatest width of abdomen 13.6–16.3, profemora 12.4–13.6, mesofemora 10.4–12.3, metafemora 12.7–14.4, protibiae 8.7–10.2, mesotibiae 7.5–8.4, metatibiae 9.0–10.2.

**Figure 21.**
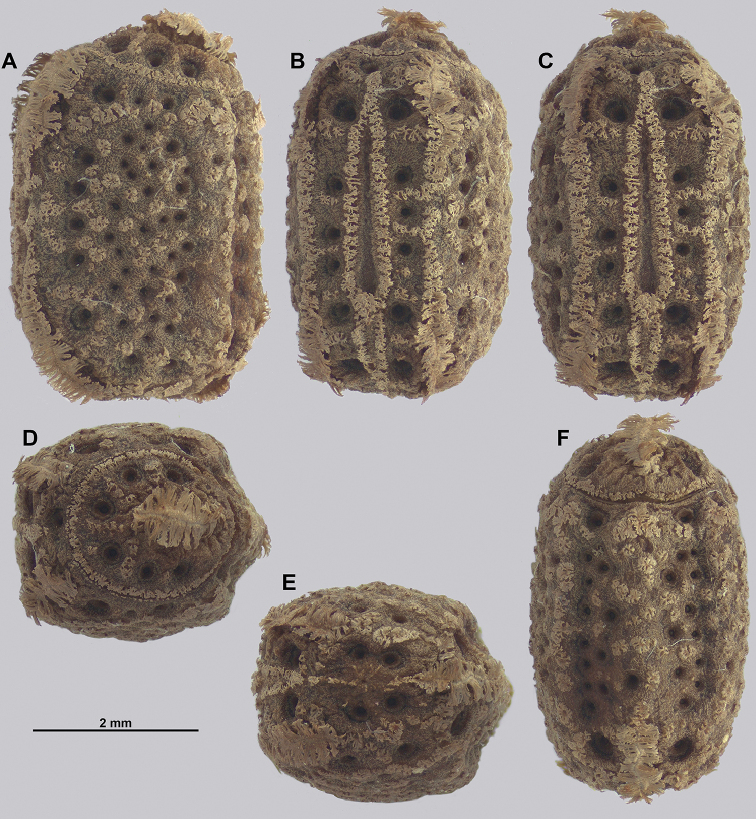
*Cryptophylliumbollensi* gen. et sp. nov. egg (RBINS), photographs by Jérôme Constant **A** lateral view **B** dorso-lateral view **C** dorsal view **D** opercular (anterior) view **E** posterior view **F** ventral view.

**Eggs.** (Fig. 21). The overall color is muted dark brown, with the moss-like pinnae lighter in color, generally tan or light brown. The lateral surfaces are flat or slightly convex, with eggs either the same width anterior to posterior or with the posterior of the egg slightly wider. The lateral surfaces are marked with 40–50 small to medium sized pits, unevenly spaced in no detectable pattern, with sparse tufts of moss-like pinnae between these pits (Fig. 21A). The dorsal surface has the micropylar plate spanning a majority of the length but not quite reaching end to end. On either side of the micropylar plate is variable pitting (generally eight or so pits) with those one the anterior and posterior ends slightly larger than the central pits (Fig. 21C). The micropylar plate is symmetrical with the anterior and posterior thin and the middle the widest point. The micropylar cup is not located at this widest midpoint but is instead located on the posterior ⅓ of the micropylar plate. The micropylar plate margin is lined with short moss-like pinnae. Operculum slightly ovular, outer margin with a distinct row of short moss-like pinnae and in from the outer margin is a singular semi-circle of small to medium pits on the dorsal and lateral aspects (not fully surrounding the apex of the operculum as the ventral portion lacks these pits; Fig. 21D). Operculum is roundly raised with the height ca. ½ the operculum width and the apex of the raised operculum has a tuft of moss-like pinnae. The ventral surface of the egg capsule has a slightly raised sagittal crest marked sparsely with short moss-like pinnae on the anterior ⅔, and the posterior ⅓ has longer moss-like pinnae. On either side of this raised sagittal crest is pitting, near the posterior ⅓ on each side of the longer moss-like pinnae of the sagittal crest is a large circular pit, and anterior to the lowest large pit are around ten small to medium pits arranged in no detectable pattern (Fig. 21F).

**Measurements including the extended pinnae [mm].** Length (including operculum): 4.1–4.4; maximum width of capsule when viewed from lateral aspect 2.6–2.8; length of micropylar plate 3.3–3.4

**Newly hatched nymphs.** (Fig. 9H). The general color throughout the body is dark brown with slightly lighter brown on the legs. The basitarsi are yellow and remaining tarsal segments are dark brown. All tibiae lack exterior lobes but do have extremely thin smoothly arcing interior lobes which have several tan to brown stripes throughout their length. All femoral lobes are similar in width and have distinct serration on their distal halves. The interior profemoral lobe lacks a white spot, but the exterior lobe has a distinct white patch on the proximal ⅓ with an additional small white patch at the proximal most margin. The meso- and metafemoral interior lobes have two white patches, one on the proximal most edge, and a larger white patch ⅓ of the way through the length. The meso- and metafemoral exterior lobes also have a large white patch on the proximal ⅓, but lack a smaller white patch on the proximal most margin. The distal ends of the meso- and metafemora also have minimal white edges. The abdomen is mostly brown, but abdominal segments II and III have distinct green patches on their lateral surfaces (the centerline of the abdomen is uniform brown throughout). The terminal three abdominal segments also have a little bit of green on their margins. The widest point of the abdomen is abdominal segment IV.

###### Etymology.

Patronym. Named after Tim Bollens (Belgium) who has been instrumental in bringing many new phylliid species into the phasmid breeding community over the years. With his expertise in breeding these difficult phasmids he has allowed us to compare the informative sets of male, female, freshly hatched nymph, and egg morphology instead of only comparing singular dead specimens collected in the wild.

##### Cryptophyllium celebicum

Taxon classificationAnimalia

(de Haan, 1842) comb. nov.

9E1ECF64-5E4D-5FD6-AAC3-28B2E0E41250

**Figure 22.**
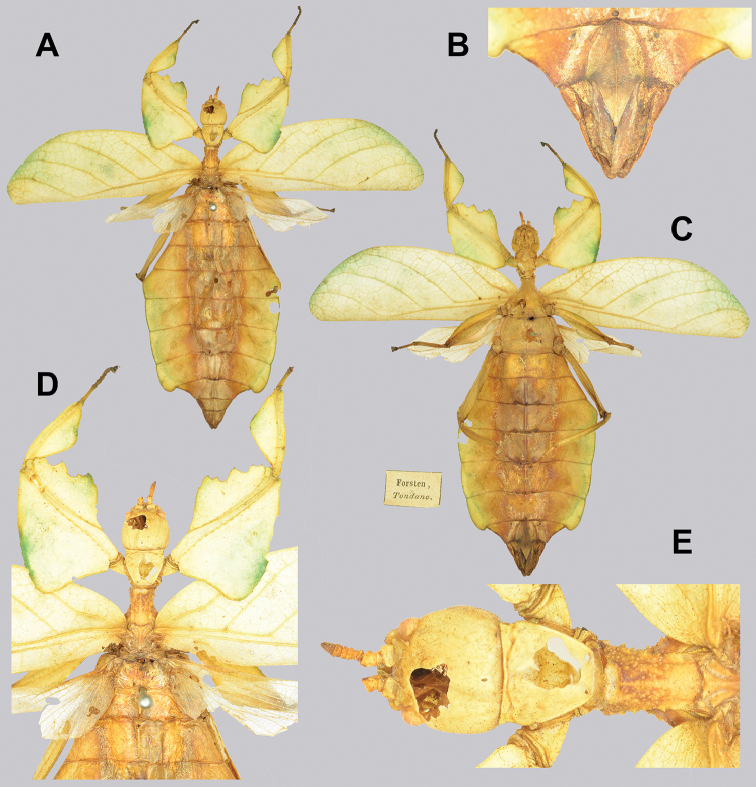
Holotype, *Cryptophylliumcelebicum* (de Haan, 1842), comb. nov. the type species for the *Cryptophyllium* gen. nov. Photographs by Luc Willemse, Naturalis Biodiversity Center (RMNH) **A** dorsal, habitus **B** genitalia, ventral **C** ventral, habitus, and original collection label inset to left **D** details of the front legs, head, and thorax, dorsal **E** details of the antennae and thorax, dorsal.

**Figure 23.**
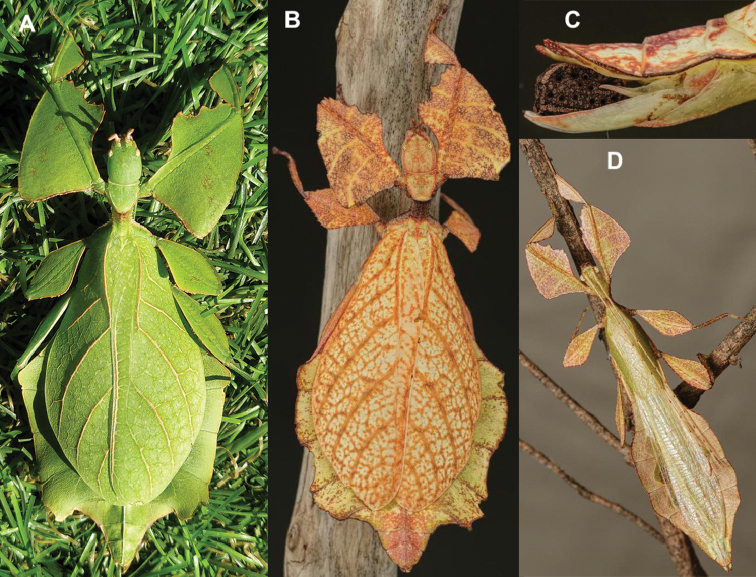
Live *Cryptophylliumcelebicum* comb. nov. **A** green form female, dorsal, bred and photographed by Thomas Stijnts (Belgium; Flanders) **B–D** bred and photographed by Bruno Kneubühler (Switzerland) **B** orange form adult female, dorsal **C** lateral view of the female genitalia holding an egg ready to be flicked away. Note the large gonapophyses VIII and the smaller gonapophyses IX holding the egg. **D** orange form adult male, dorsal.

Figures 6C , 8P , 8P , 9A , 22 , 23

###### Material examined.

(11 ♀♀, 3 ♂♂, 5 eggs): 6 ♀♀: “Indonesia: Sulawesi” (Coll RC 16-069, 16-070, 16-075, 16-238, (nymph) 16-074, (nymph) 16-072); 2 ♀♀: “Indonesia: Sulawesi, Palolo, Palu, 2.2008” (Coll RC 16-071, (nymph) 16-073); 1 ♀: “Indonesia: Peleng, Tattendeng, Sept. 2019” (Coll RC 19-181); 2 ♂♂: “Indonesia: Sulawesi” (Coll RC 16-146, 16-076); 1 ♂: “Sulawesi, Central Sulawesi Province, Palu Palolo: February, 2008” (Coll RC 16-145); 1 ♀: “Coll. I.R.Sc.N.B., Indonesia, Sulawesi, Puncak BEI, Palopo, VI.2001” (RBINS); 1 ♀: “Indonesia: Bugadidi, ex culture T. Bollens” (RBINS); 2 eggs: “Indonesia: S-Sulawesi, Tiulapolu leg. Jasmin III.2008, F-1 Generation, Cultured F.Hennemann 2009 Ex. Coll. Frank Hennemann (Germany)” (Coll RC 18-250, 18-251); 3 eggs: “Indonesia: Sulawesi; removed from specimen Coll RC 16-075” (Coll RC 17-345, 17-346, 17-347).

###### Remarks.

This was the first species described within the newly erected *Cryptophyllium* gen. nov. and we herein designate it as the type species for this new genus. This species is now well-known and little confusion surrounds this species’ true identity. This has not always been the case however as for years it was the subject of repeated misidentifications by many authors (see Hennemann et al. (2009) for a thorough list of misidentifications which instead represented species such as *Phylliumericoriai*Hennemann et al., 2009 from the Philippines and the closely related *Cryptophylliumwestwoodii* comb. nov. from mainland Asia). Gray (1843) appears to be the first to erroneously state that ‘*Phylliumcelebicum*’ occurs in the Philippines and Wood-Mason (1875) disrupted the mainland Asia identifications when he claimed that ‘*Phylliumcelebicum*’ could be found in Myanmar. These two works snowballed for decades as nearly all specimens from Northern Thailand (a major commercial breeding site for *Cryptophylliumwestwoodii* comb. nov.) were sold as ‘*Phylliumcelebicum*’ therefore confusing collectors and researchers. Additional confusion likely occurred due to the fact that the last publication explicitly recording the holotype ‘*Phylliumcelebicum*’ appears to have been by Willemse (1947 [1945]) when he illustrated it and then it subsequently went missing despite several attempts to locate it by other authors (for example by Hennemann et al. in April of 2006 in their review of the RMNH collection). Thankfully the holotype specimen was located by the authors of this work while reviewing photographs of the RMNH collection and appears to have been overlooked as it was misplaced and labeled with “Type, Phylliumcrurifolium Serv., 1938 (sic!)”, a simple mistake but one that shows how important proper labeling can be. Now the holotype is properly labelled, and we here present the first photographs of this important specimen (Fig. 22).

Interestingly, this species is commonly collected and sold from the forest of Sulawesi solely as green color form specimens, but nearly all captive bred individuals are yellow to orange in coloration (Fig. 23B, D) with green individuals captive reared quite rare (Fig. 23A). Molecularly, we unfortunately do not have a wide sampling from throughout Sulawesi or the surrounding islands so we do not yet know the average intraspecific variation on Sulawesi. We were able to obtain a molecular sample from Peleng Island off the northeast Sulawesi coast which shows a notable molecular distance from our single mainland sample (Fig. 4). This Peleng specimen did not have significant morphological differences to differentiate it from the mainland series we examined, and due to our lack of sampling from throughout Sulawesi within this review we treat this offshore population as identical. Perhaps additional molecular sampling will reveal the true intraspecific variation of this species and warrant the Peleng population to be described as a sister species one day.

###### Differentiation.

Females can be differentiated by the following combination of features: mesopleura which are narrow on the anterior half, alae which are ca. ½ the length of the tegmina, and profemoral exterior lobes which are broad and slightly recurved which gives them an acute angle at the bend. Two species which are morphologically very similar are *Cryptophylliumechidna* sp. nov. and *Cryptophylliumlimogesi* sp. nov. due to the abdominal and femoral lobe shapes. *Cryptophylliumechidna* sp. nov. is the molecular sister species to *Cryptophylliumcelebicum* comb. nov. and morphologically very similar with the only easy to differentiate feature being the profemoral exterior lobe which in *Cryptophylliumechidna* sp. nov. is nearly right angled, not slightly recurved with an acute angle. The male and egg morphology are not known for *Cryptophylliumechidna* sp. nov. but hopefully once that is observed, additional features can be identified. *Cryptophylliumlimogesi* sp. nov. has a very similarly shaped abdomen and exterior profemoral lobes, but can immediately be differentiated by the mesopleura, which are prominent and reach nearly to the anterior rim (Fig. 42E) vs. *Cryptophylliumcelebicum* comb. nov. which has the mesopleura narrowed on the anterior rim (Fig. 22E).

**Figure 24.**
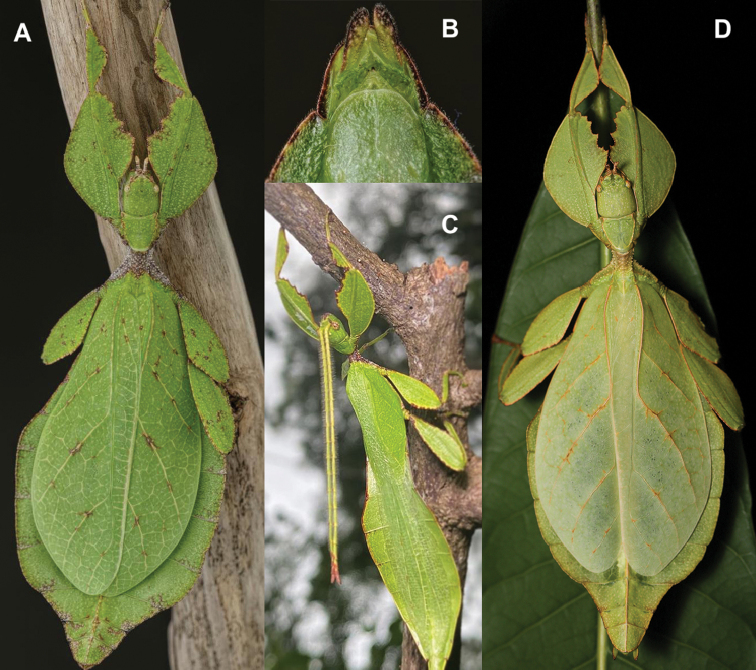
Live *Cryptophylliumchrisangi* comb. nov. **A** female bred and photographed by Bruno Kneubühler (Switzerland) **B** male genitalia, ventral view, bred by Bruno Kneubühler (Switzerland) **C** male observed and photographed by Mathieu MJP Van Goethem (South Africa) on Weh Island off the north shore of Sumatra **D** female observed and photographed in Thailand: Nakhon Si Thammarat Province, Thung Song District, by Tatsatorn Dharithai (Thailand) in August 2020.

Males are rather morphologically unique as they have profemoral exterior lobes, which are broad and strongly angled almost to a right angle. No other known males of *Cryptophyllium* gen. nov. have such a prominent profemoral exterior lobe as they either have a narrow rounded lobe (like in *Cryptophylliumyunnanense* comb. nov.; Fig. 75B) or an exterior profemoral lobe, which at most is broad with a distinct bend, but still clearly obtuse (like in *Cryptophylliumlimogesi* sp. nov. or *Cryptophylliumoyae* comb. nov.; Fig. 50B).

###### Distribution.

Known from throughout the island of Sulawesi and from the nearby offshore islands of Peleng to the east and Buton to the south.

##### Cryptophyllium chrisangi

Taxon classificationAnimalia

(Seow-Choen, 2017) comb. nov.

A16BAF51-41C0-5232-A409-75226EEEBA20

**Figure 25.**
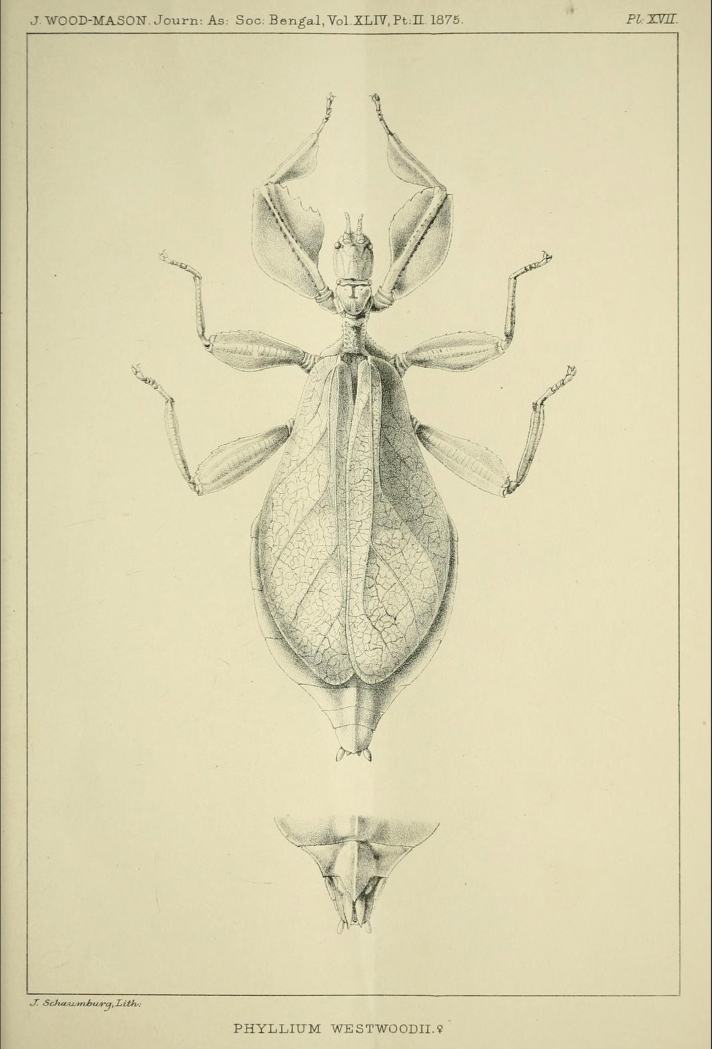
Wood-Mason’s 1875 plate XVII with the beautifully illustrated ‘*Phylliumwestwoodii*’ female syntype from South Andaman. Note the morphological similarity to *Cryptophylliumchrisangi* comb. nov. and given the wide range of *Cryptophylliumchrisangi* comb. nov. outside of the type locality of Singapore, this record may represent a range expansion. Public domain work downloaded from the Biodiversity Heritage Library (https://www.biodiversitylibrary.org/item/114410#page/227/mode/1up)

**Figure 26.**
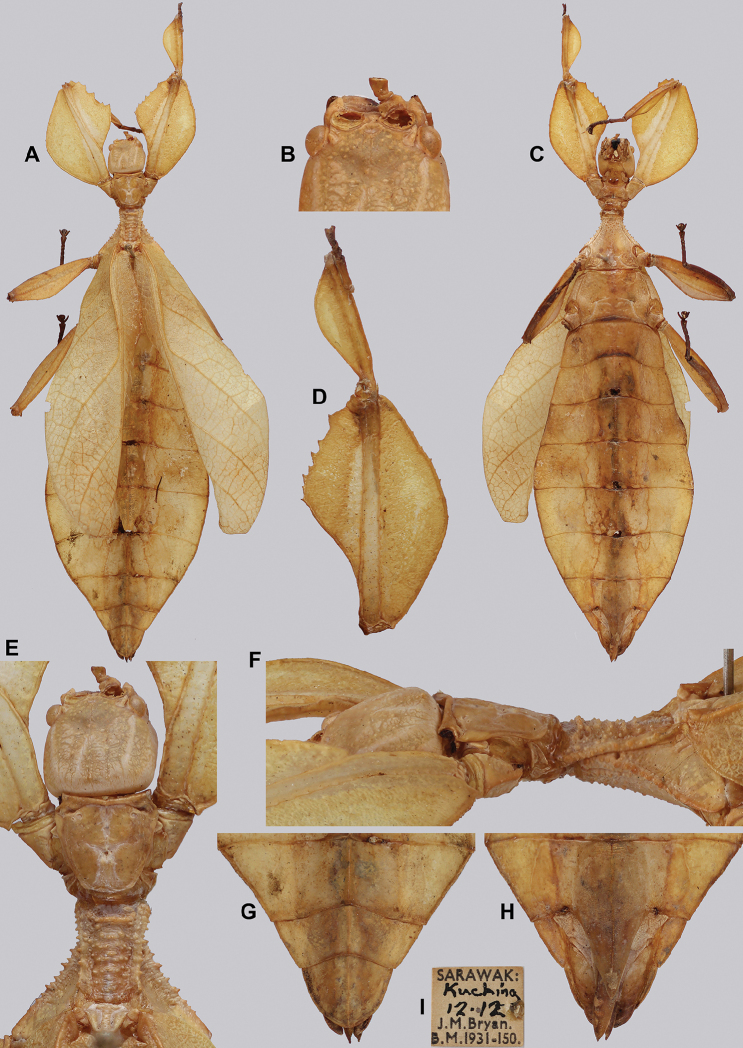
Tentative female *Cryptophylliumchrisangi* comb. nov. from Kuching, Sarawak, Malaysia which represents a range expansion for this species, photographs by Jérôme Constant (RBINS) **A** habitus, dorsal **B** details of the anterior of the head, dorsal **C** habitus, ventral **D** front leg details, dorsal **E** details of the head and thorax, dorsal **F** details of the head and thorax, lateral **G** genitalia details, dorsal **H** genitalia details, ventral **I** data label.

Figures 8M , 8N , 9B , 24 , 25 , 26

###### Material examined.

(7 ♀♀, 5 ♂♂, 10 eggs): 2 ♂♂, 2 ♀♀: “Singapore, ex breeding” (RBINS); 1 ♂: “Singapore (Pulau Ubin), Collected at light, by Francis Seow-Choen, January 5^th^, 2018 (0-75 m elv)” (Coll RC 18-279); 1 ♂: “Thailand: Satun Province, Tarutao Island” (UCR); 1 ♀: “Sarawak: Kuching, 12.12, J.M. Bryan., B.M.1931-150.” (NHMUK); 1 ♀: “P.siccifolium, 4.93 Tapah, Dr. Yeh” (LKCNHM); 10 eggs: “Singapore” (Coll RC 18-332–18-341); 1 ♀: “Presented by Dr. Brooke, St. John’s I. 26.4.09, Coll Freie Universitat Berlin Sammlung Exotische Insekten, DEI Hemimetabola #100117”.

***Photographic records***: 2 ♀♀: Thailand: Nakhon Si Thammarat Province, Thung Song District, August 2020 (photographed by Tatsatorn Dharithai, Thailand);

1 ♂: Indonesia, Pulau Weh Island off the coast of Sumatra (photographed by Mathieu MJP Van Goethem, South Africa).

###### Remarks.

This species is one which was brought into the breeding community (Fig. 24A) and has therefore allowed thorough observation of the adults, nymph, and egg morphology. Molecularly, *Cryptophylliumchrisangi* comb. nov. is sister species to *Cryptophylliumwestwoodii* comb. nov. with these two species biogeographically separated by the Isthmus of Kra. The Isthmus of Kra is a notable line of biogeography for several organisms (e.g., Li and Li 2018; Parnell 2013) which separates the Ranong and Chumphon Provinces of Thailand (Fig. 2). This isthmus appears to be significant for the Phyllium (Phyllium) as no species are presently known north of this line, but insignificant for the Phyllium (Pulchriphyllium) as this group is found on both sides of this line.

With our herein designation of a neotype for *Cryptophylliumwestwoodii* comb. nov. we can help to clear up possible significant confusion which surrounds this species. When Wood-Mason described this species in 1875 he did so with a male and female pair of syntypes from two different localities. The female was from “South Andaman” and the male was from “near Pahpoon, ca. 150 miles north of Moulmein, in the Salween country” (Wood-Mason 1875). It is almost certain biogeographically that these syntypes represent two different phylliid species, not a singular *Cryptophylliumwestwoodii* comb. nov. as he intended. Morphologically, Wood-Mason’s description and wonderful illustration of the female syntype interestingly appears very similar morphologically to *Cryptophylliumchrisangi* comb. nov. with the correct femoral and abdominal shapes, and alae length (Fig. 25). With our designation of a neotype *Cryptophylliumwestwoodii* comb. nov. from a male from the mainland, this leaves the Andaman female somewhat mysterious as few records> of phylliids have been noted from the Andaman Islands, all of which are Phyllium (Pulchriphyllium) bioculatum-like species, none representing a *Cryptophyllium* gen. nov. species. Hopefully one day a fresh specimen of this *Cryptophyllium* gen. nov. species from the Andamans can be located and molecularly compared with congenerics to identify if it is an additional *Cryptophylliumchrisangi* comb. nov. range expansion or an undescribed species.

###### Differentiation.

Females can be differentiated by the following combination of features: spade-shaped abdomen (as segment VII lacks lobes), mesopleura which are distinctly narrower on the anterior half, and alae which are only ca. ½ as long as the tegmina. Morphologically, this species is similar to *Cryptophylliumwestwoodii* comb. nov. due to the femoral and mesopleura shape, but can be differentiated by the shorter alae (only half of the tegmina length) as *Cryptophylliumwestwoodii* comb. nov. has alae which are nearly the same length as the tegmina (Fig. 68D). Additionally, *Cryptophylliumathanysus* comb. nov. is morphologically similar due to the femoral, mesopleura, and abdominal shape, but can immediately be differentiated by the metatibial exterior lobes which are lacking in *Cryptophylliumchrisangi* comb. nov.

Males are morphologically very similar to *Cryptophylliumwestwoodii* comb. nov. and we have yet to find a reliable morphological feature to differentiate these two species. Both are morphologically variable and can even have a wide range of sizes which does not allow for confident differentiation when molecular markers and locality are unknown. One of the more consistent features however is the abdominal shape as *Cryptophylliumwestwoodii* comb. nov. tends to have a slightly more spade-shaped abdomen with segments V–IX converging, and *Cryptophylliumchrisangi* comb. nov. having a slightly more ovoid abdomen with segments V–VI parallel or subparallel, but we have seen morphological intermediates which do not allow this as a diagnostic feature.

###### Distribution.

The type locality for *Cryptophylliumchrisangi* comb. nov. is mainland Singapore and it has additionally been recorded from St. John’s Island from a record in the SDEI collections. Additionally, we have observed specimens and photographs from several areas, including several from Thailand: Nakhon Si Thammarat Province, Thung Song District (Fig. 24D) and Satun Province, Tarutao Island (UCR coll.); one tentative old record of a large female from Malaysia, Sarawak, Kuching, from the NHMUK collection (Fig. 26); an adult female from Tapah, Malaysia (LKCNHM); and a record from Indonesia, Pulau Weh Island off the coast of Sumatra (Fig. 24C). Hopefully molecular samples from these far-reaching areas can one day be obtained to confirm the identification as *Cryptophylliumchrisangi* comb. nov. but for now morphologically this is what these records> appear to represent.

##### Cryptophyllium daparo

Taxon classificationAnimalia

gen. et sp. nov.

DB5E7671-6257-5058-8592-1E4B977E7CF1

http://zoobank.org/92D078EF-D482-4F03-AB57-E63EEFE742A5

Figure 27

###### Material examined.

***Holotype*** ♀: “CHINA: Yunnan, Wangtianshu, Mengla County, Xishuangbanna Prefecture, VII-2016, Legit: Xiao-Yu Zhu”. Deposited in the Kunming Institute of Zoology (KIZ), Yunnan, China.

###### Remarks.

This large species is at present only known from the single holotype female, which has a unique set of morphological features which do not link it to the molecularly recovered closely related species of *Cryptophylliumdrunganum* comb. nov. and *Cryptophylliumtibetense* comb. nov. Geographically, these species do represent some of the highest latitude species and interestingly *Cryptophylliumdaparo* sp. nov. and *Cryptophylliumtibetense* comb. nov. are the largest species in this genus. Hopefully further collection efforts in this region will reveal the unknown male and the presently unknown egg morphology.

###### Differentiation.

Females are morphologically most similar to *Cryptophylliumathanysus* comb. nov. and *Cryptophylliumrarum* comb. nov. due to the tapered, spade-like abdomen and the anteriorly narrower mesopleura. From both species *Cryptophylliumdaparo* sp. nov. can be differentiated by the exterior profemoral lobe shape which is distinctly obtuse and nearly rounded in its shape, not smoothly right-angled like in the other species. *Cryptophylliumdaparo* sp. nov. is also notably larger than *Cryptophylliumathanysus* comb. nov. and *Cryptophylliumrarum* comb. nov. which are only 77 and 88 mm long respectively (Hennemann et al. 2009; Liu 1993).

Males are presently unknown, and the unique female morphology means we cannot predict much about the male morphology. The only feature which can be guessed is that the male must also be rather large due to the female size.

###### Distribution.

At present only known from the unique holotype collected in China, Yunnan, Wangtianshu, Mengla County, Xishuangbanna Prefecture.

###### Description.

**Figure 27.**
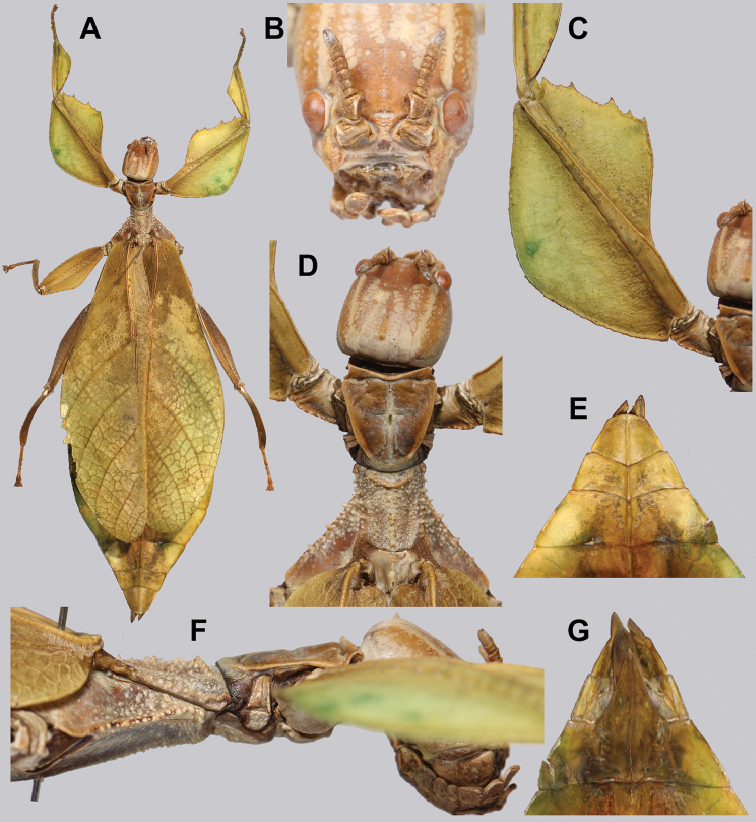
Holotype female *Cryptophylliumdaparo* gen. et sp. nov., photographs by Zhiwei Dong (KIZ) **A** habitus, dorsal **B** details of the face and antennae, rostral view **C** profemoral lobes, dorsal **D** details of the head and thorax, dorsal **E** terminalia, dorsal **F** details of the head and thorax, lateral **G** genitalia, ventral.

**Female. *Coloration.*** Coloration description is based upon the dried holotype which is somewhat discolored (Fig. 27). Leaf insects are a more vibrant lime green in life and we expect that this specimen was likely originally this color. The holotype female is mostly tan to pale green throughout and appears to not have any natural brown patches of color as are sometimes present on the lobes or thorax.

***Morphology.****Head.* Head capsule about as long as wide, vertex with moderately spaced small granulation, and two notable tubercles, one on each side of the sagittal plane near the midline of the head capsule which are larger than the rest, but not as prominent as the posteromedial tubercle (Fig. 27D). The posteromedial tubercle is about as broad as the two tubercles on the capsule, but the posteromedial tubercle is notably taller and pointed (easily seen from the lateral aspect; Fig. 27F). Frontal convexity broad but not very large, with an irregularly lumpy surface and sparse transparent setae throughout. Compound eyes only slightly protruding from the head capsule and only occupying ca. ⅕ of the head capsule length (Fig. 27D). Ocelli absent. *Antennae.* Antennae consisting of nine segments, with the terminal segment slightly shorter than the length of the preceding two segments’ lengths combined (Fig. 27B). Antennomeres I–VIII sparsely marked with small transparent setae, the terminal antennomere is covered in stout, brown setae. *Thorax.* Pronotum with a distinctly concave anterior margin and nearly straight lateral margins, which converge to a straight posterior margin that is half the width of the anterior margin (Fig. 27D). The pronotum surface is marked with slight granulation, with a prominent pit in the center, and distinct furrows anterior and posterior to the pit and slight furrows lateral to the central pit (Fig. 27D). The pronotum has a prominent anterior rim which is marked throughout by minute setae and moderately formed lateral rims, and a posterior rim which is weakly formed (Fig. 27D). Prosternum with moderate nodes, irregularly sized and spaced. Mesosternum with similar nodes as on the pronotum, but only along the margins and on the anterior half of the sagittal plane, the remainder of the surface is rather smooth. Metasternum with an irregularly lumpy surface, no strong nodes. Prescutum slightly longer than wide and with nearly parallel lateral margins (Fig. 27D). Lateral rims with eight or nine irregularly shaped but short tubercles with various small nodes mixed throughout, giving the margins a rough textured appearance (Fig. 27D). Prescutum anterior rim distinct, but not strongly raised above the prescutum surface, the rim has a granular surface, no distinct sagittal tubercle larger than the rest (Fig. 27F). Prescutum surface with irregular nodes throughout, with those along the sagittal plane slightly more prominent (Fig. 27D). Mesopleura beginning slightly posterior to the anterior margin of the prescutum and evenly diverging; lateral margin with three or four larger tubercles throughout the length, and ten or eleven smaller node-like tubercles interspersed (Fig. 27D). Face of the mesopleura with granulation throughout and slight wrinkling on areas where the nodes are less prominent (Fig. 27F). *Wings.* Tegmina long, extending three quarters of the way through abdominal segment VII. The subcosta (Sc) is the first vein in the forewing and runs parallel with the wing for the first half and then distinctly bends towards the distal margin where it terminates ca. ¼ of the way through the wing length. The radius (R) fills approximately the anterior ⅓ of the wing as two subparallel branched veins; radius 1 (R1) terminates ca. ⅓ of the way through the wing length, and the radial sector (Rs) terminates posterior to the widest portion of the tegmina, just past the midline. There is a thinner continuation of the radius following the prominent Rs branching which continues on as a short R–M crossvein that does not appear to solidly connect the two veins. The media (M) is bifurcate with both the media anterior (MA) and media posterior (MP) terminating close to the posterior ⅓ of the wing. The cubitus (Cu) is also bifurcate, branching near the posterior ¼ of the wing into the cubitus anterior (CuA) and cubitus posterior (CuP) which both terminate at or very near the wing posterior apex. The first anal vein (1A) is simple and fuses with the cubitus early on, only slightly past the branching distance of the R1 from the R. Alae well developed, 58.5 mm long, only a little shorter than the tegmina. *Abdomen.* Segments II through the anterior ⅓ of IV gradually diverging, with the posterior ⅔ of segments IV and V parallel-sided. Segments VI–X are gradually converging to the broadly rounded apex, giving the abdomen a smooth spade-shaped appearance (Fig. 27A). *Genitalia.* Subgenital plate starts at the anterior margin of segment VIII, is broad, and extends ca. ⅔ of the way onto segment X, ending in a fine point (Fig. 27G). Gonapophyses VIII are long and moderately broad, slightly exceeding the apex of abdominal segment X (Fig. 27G); gonapophyses IX are thinner and shorter and are concealed below the larger gonapophyses VIII. Cerci flat, not strongly cupped, with a granular surface and rough granular lateral margins (Fig. 27E). *Legs.* Profemoral exterior lobes slightly broader than the interior lobe, roundly arcing from end to end in a broad obtuse angle (Fig. 27C). Edge of the profemoral exterior lobe with a highly granular surface on the proximal margin, and the distal margin has less granulation, but does have five or six small but notable teeth (Fig. 27C). Profemoral interior lobe ca. 3× as wide as the greatest width of the profemoral shaft, and with a distinct obtuse angle and a distal margin marked by four or five prominent serrate teeth, with a larger gap between the middle teeth (Fig. 27C). Mesofemoral exterior lobe arcs from end to end with a distinct bend near the center slightly weighted towards the distal half and marked with two or three dulled serrate teeth on the distal half only. Interior mesofemoral lobe is slightly narrower than the exterior lobe. Mesofemoral interior lobe arcs end to end with five or six serrate teeth on the distal half of the arc only. Metafemoral interior lobe narrow, arcing end to end, and marked with five or six serrate teeth and slight granulation on the distal half of the lobe only. Metafemoral exterior lobe is thin and smooth, hugging the metafemoral shaft and lacks teeth. Pro-, meso-, and meta- tibiae lacking exterior lobes. Protibial interior lobe spans the entire length, is ca. 2× the width of the shaft, and is roundly triangular with the widest portion on the distal half (Fig. 27A). Meso- and metatibiae lacking interior lobes.

***Measurements of holotype female* [mm].** Length of body (including cerci and head, excluding antennae) 107.0, length/width of head 8.5/7.0, antennae 5.0, pronotum 6.3, mesonotum 7.0, length of tegmina 67.5, length of alae 58.5, greatest width of abdomen 32.0, profemora 27.0, mesofemora 19.2, metafemora 20.0, protibiae 17.4, mesotibiae 18.0, metatibiae 18.5.

###### Etymology.

Noun, named for the artistic company “DAPARO”, owned by Daparo-Yeung which is well-known for their beautiful natural history themed brooches. Several years ago, DAPARO even produced a beautifully crafted leaf insect themed brooch which helped to shed light on these beautiful creatures and bring them into the public eye.

##### Cryptophyllium drunganum

Taxon classificationAnimalia

(Yang, 1995) comb. nov.

37ECB48A-D860-50F7-AB81-4CAADB108F15

Figures 28 , 29

###### Material examined.

We examined the holotype female from within the Beijing Agricultural University from detailed photographs taken by Yu-Chen Zheng (China Agricultural University, China). Additionally, we examined a tentatively identified male from “Yunnan China: Qinglangdang, Dulongjiang Township, Gongshan County, Nujiang Prefecture, II-2016, Local” (Coll ZD).A tentative male specimen collected very near the type locality was used in our molecular analysis, which cannot be confidently confirmed as *Cryptophylliumdrunganum* comb. nov. due to extreme sexual dimorphism of the phylliids and lack of a fresh tissue sample from a true *Cryptophylliumdrunganum* comb. nov. female (Fig. 29).

###### Remarks.

**Figure 28.**
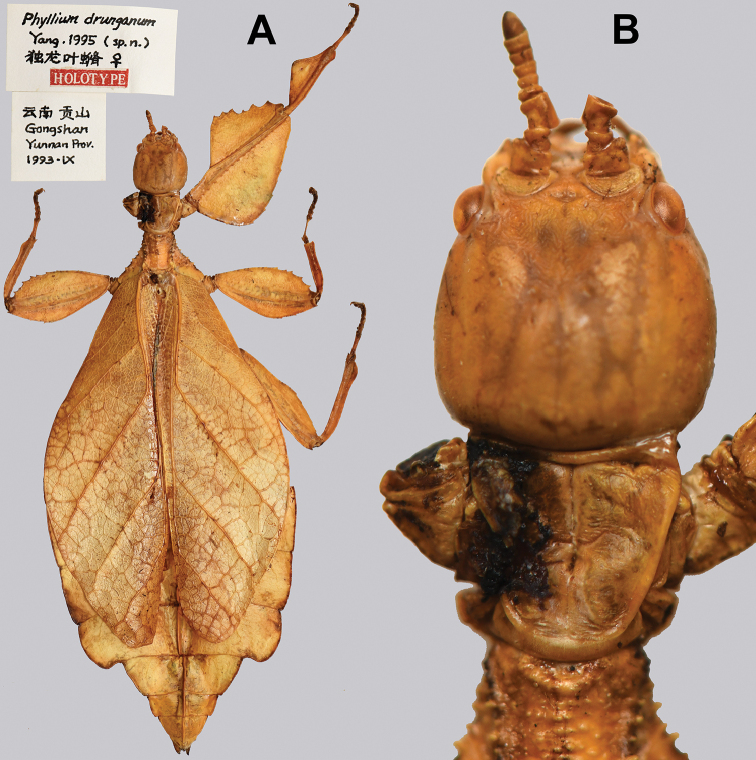
*Cryptophylliumdrunganum* comb. nov. holotype female within the Beijing Agricultural University, photographed by Yu-Chen Zheng (China Agricultural University, China) **A** habitus, dorsal, inset specimen data labels **B** details of antennae, head, and anterior of the thorax.

This species is only known at present from the morphologically unique holotype female from northern Yunnan Province (Fig. 28). However, this area is not known for a high diversity of species, so we are fairly confident that this male represents the undescribed *Cryptophylliumdrunganum* comb. nov. male. Also, this male specimen was molecularly recovered as distinct to the other species described from China as we have successfully sampled almost all species of *Cryptophyllium* gen. nov. and included them in our molecular phylogeny (Fig. 4).

**Figure 29.**
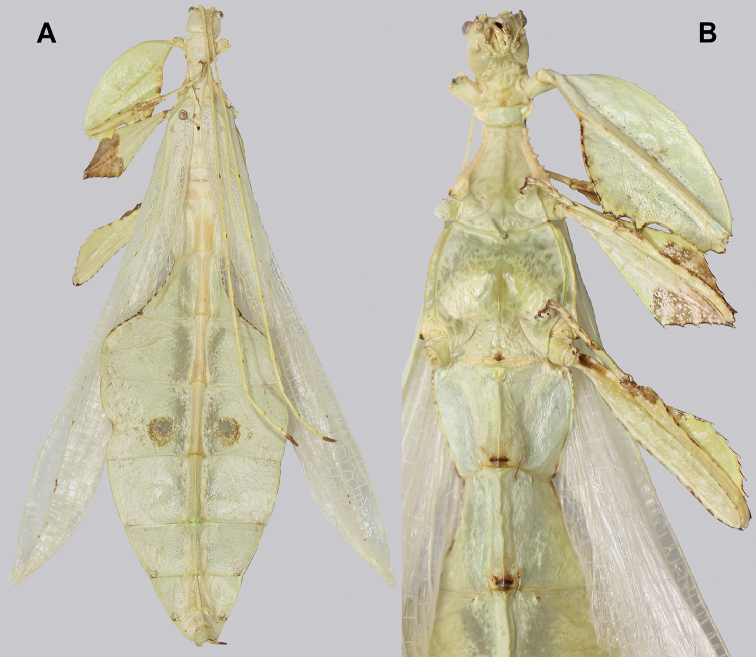
Presumed male *Cryptophylliumdrunganum* comb. nov. male used within our molecular analysis (sample DZW05), from Du Longjiang Township, Nujian Lisu Autonomous Prefecture, photographs by Zhiwei Dong (KIZ) **A** habitus, dorsal **B** details of the legs and thorax, ventral.

###### Differentiation.

Females are morphologically similar to *Cryptophylliumtibetense* comb. nov. and *Cryptophylliumliyananae* sp. nov. due to the long alae, rounded exterior profemoral lobe, mesopleura which are distinctly reaching the anterior margin but slightly curved on the anterior end (not perfectly straight margined), boxy abdomen with a notable bend on abdominal segment VII, and the presence of small exterior lobes on all tibiae. Both of these species can be differentiated by the length of the subgenital plate as in *Cryptophylliumdrunganum* comb. nov. it is short, just passing the anterior margin on the tenth abdominal segment, and in the other species it is at least three quarters of the length of the tenth abdominal segment (in *Cryptophylliumliyananae* sp. nov.) or even longer and exceeding the tip of the abdomen (in *Cryptophylliumtibetense* comb. nov.).

Our male specimen is morphologically similar to *Cryptophylliumtibetense* comb. nov. and *Cryptophylliumyunnanense* comb. nov. due to the shape of the profemoral exterior lobe which smoothly arcs end to end without a distinct bend, the exterior profemoral lobe that is the same width or slightly thinner than the interior lobe (not wider as is common in many of the *Cryptophyllium* gen. nov. species), tegmina which are long reaching the anterior margin of abdominal segment IV or slightly passing it, a similar spade-shaped abdomen, and prominent tubercles on the mesopleura.

*Cryptophylliumtibetense* comb. nov. males additionally have small exterior tibial lobes on the distal ends like are present in our *Cryptophylliumdrunganum* comb. nov. male. Our male *Cryptophylliumdrunganum* comb. nov. can however be differentiated from *Cryptophylliumtibetense* comb. nov. by the presence of eight or nine small serrate teeth present throughout the full length of the profemoral exterior lobe vs. *Cryptophylliumtibetense* comb. nov. males which only have two or three small teeth on the distal end only. Additionally, the mesofemoral exterior lobe also can differentiate these species as it is distinctly angled in our *Cryptophylliumdrunganum* comb. nov. male and smoothly arcing without a distinct bend in *Cryptophylliumtibetense* comb. nov. males.

*Cryptophylliumyunnanense* comb. nov. can be differentiated by the absence of exterior tibial lobes and the lack of a distinctly serrate exterior profemoral lobe margin, with *Cryptophylliumyunnanense* comb. nov. only having two or three small teeth vs. our male *Cryptophylliumdrunganum* comb. nov. which has eight or nine small serrate teeth present throughout the full length of the profemoral exterior lobe.

###### Distribution.

At present only known from northern Yunnan Province, from the type locality of Nujian Lisu Autonomous Prefecture, Gongshan County (Drung-Nu), and our tentative male *Cryptophylliumdrunganum* comb. nov. from Du Longjiang Township, Qing Lang Dang in the same prefecture.

##### Cryptophyllium echidna

Taxon classificationAnimalia

gen. et sp. nov.

B7FA223E-2335-5527-9DF4-863A17B08329

http://zoobank.org/076CABC1-2772-4E56-9918-F3804B62356E

Figure 30

###### Material examined.

***Holotype*** ♀: INDONESIA: Wangi-wangi Island. Collected prior to 2020, but no exact date given. Deposited in the Montreal Insectarium (IMQC).

###### Remarks.

This is the first phylliid record we have seen from the small island of Wangi-wangi in the Wakatobi Regency in Southeast Sulawesi Province. This small island appears to be rather unique biogeographically and unexplored as a yet to be described bird species presently known as the “Wangi-wangi White-eye” (*Zosterops* sp. nov.) has recently been identified as well, suggesting this island may hold many endemic undescribed species (O’Connell et al. 2019).

###### Differentiation.

Presently we only know of a single female specimen of this new species which we here designate as the holotype. Morphologically and molecularly this species is closely related to *Cryptophylliumcelebicum* comb. nov. which has a much wider distribution to the north on the islands of Buton, Sulawesi, and Peleng (Fig. 2).

**Figure 30.**
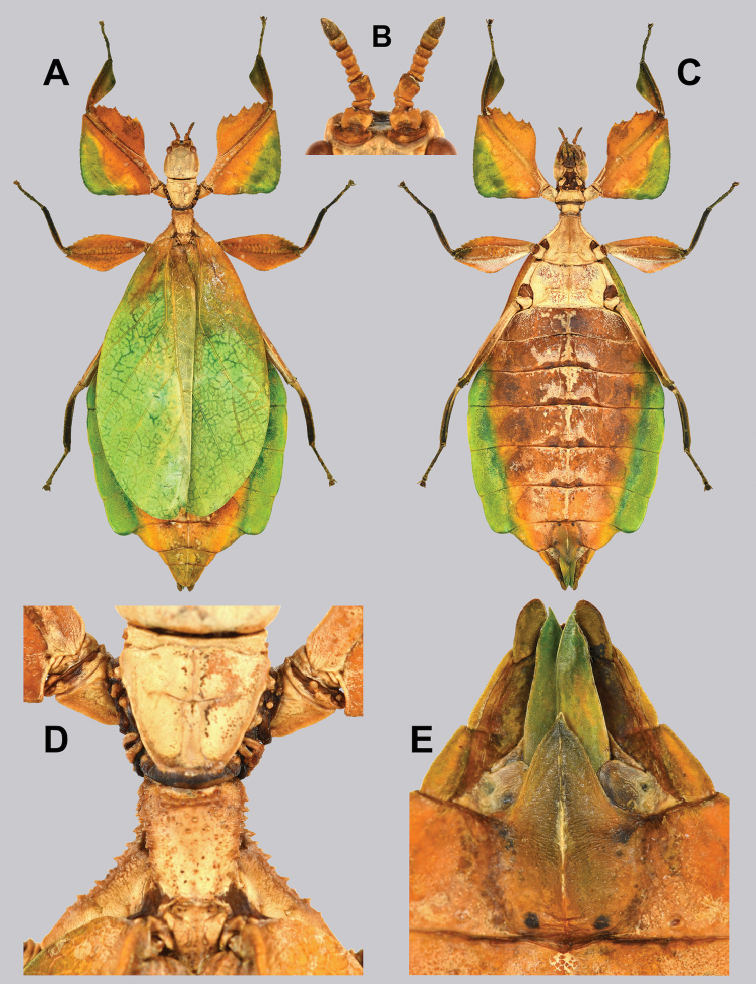
*Cryptophylliumechidna* gen. et sp. nov. holotype female, photographs by René Limoges (IMQC) **A** habitus, dorsal **B** dorsal details of the antennae **C** habitus, ventral **D** dorsal details of the thorax **E** ventral details of the genitalia.

Female *Cryptophylliumechidna* sp. nov. can be differentiated from *Cryptophylliumcelebicum* comb. nov. by only subtle differences in the thorax and profemoral exterior lobes. In *Cryptophylliumechidna* sp. nov. the prescutum is slightly broader and with a weaker sagittal crest (Fig. 30D) than in *Cryptophylliumcelebicum* comb. nov. (Fig. 22E). Additionally the profemoral exterior lobe of *Cryptophylliumechidna* sp. nov. has a right exterior angle (Fig. 30A), not acute like in *Cryptophylliumcelebicum* comb. nov. (Fig. 22D).

Males are presently unknown, but as the sister species to *Cryptophylliumcelebicum* comb. nov. the males likely have a similar morphology.

###### Distribution.

At present only known from the small Indonesian island of Wangi-wangi off the east coast of Buton Island.

###### Description.

**Female. *Coloration*.** At present we only have the dried holotype female to describe the color from which has a bit of rot through the legs, head, thorax, and the central area of the abdomen. The rotten areas are brown but are assumed to have been green in life. The remainder of the female is lime-green in color throughout, with no indication of natural brown patches (which even on somewhat rotten specimens can generally be identified) but this female appears to have been uniform green in life.

***Morphology.****Head.* Head capsule slightly longer than wide, vertex with minimal granulation throughout the surface, all relatively well-spaced with no areas on the head tightly packed. The posteromedial tubercle is the most prominent feature on the vertex of the head capsule. Frontal convexity broad and stout, shorter than the length of the first antennomere, and with a lumpy surface marked by few short transparent setae. Compound eyes not particularly large, only slightly protruding from the head capsule, taking up ca. ¼ of the length of the lateral head capsule margins (Fig. 30A). Ocelli absent. Antennal fields slightly wider than and about as long as the length of the first antennomere. *Antennae.* Antennae consist of nine segments, with the terminal segment approximately the same length as the preceding two segments’ lengths combined (Fig. 30B). Antennomeres I–III sparsely marked with small transparent setae (with the longest on the first segment), the terminal two antennomeres are densely covered in stout, brown setae. *Thorax.* Pronotum with a gently concave anterior margin and slightly convex lateral margins, which converge to a straight posterior margin that is half the width of the anterior margin (Fig. 30D). The pronotum surface lacks granulation but is slightly lumpy, with only a prominent pit in the center, and slight furrows anterior and lateral to the pit (Fig. 30D). The pronotum has a prominent anterior rim and moderate lateral and posterior rims (Fig. 30D). Prosternum and mesosternum with numerous nodes throughout the surface, all about the same size and spacing throughout. Metasternum with slightly less granulation but they are slightly larger than those on the pro- and mesosternum. Prescutum longer than wide, with a slightly broader anterior margin (Fig. 30D). Lateral rims with 6–8 prominent tubercles with various small, lumpy granules interspersed throughout the margins (Fig. 30D). Prescutum anterior rim prominent but not strongly protruding, surface is granular and lacks a prominent sagittal spine (Fig. 30D). Prescutum surface without a strongly raised sagittal crest, instead the surface is only slightly raised along the sagittal plane. The prescutum surface has moderate granulation throughout ranging in size from small to medium with irregular spacing (Fig. 30D). Mesopleura not spanning the entire length, instead with the anterior ⅓ narrow and only starting to fan out near the midline of the prescutum length. Mesopleura lateral margins with five or six larger, sharp tipped tubercles with an additional five or six smaller nodes interspersed throughout (Fig. 30D). Face of the mesopleura lacking granulation, but instead highly wrinkled and with two notable pits, one on the anterior ⅓ and one nearer the posterior ⅓ (Fig. 30D). *Wings.* Tegmina long, reaching nearly to the posterior margin of abdominal segment VII. The subcosta (Sc) is the first vein in the forewing and runs subparallel with the wing for the first half of its length, and then bends towards the wing margin for the second half. The radius (R) spans the central portion of the tegmina with two subparallel branched veins. The first radius (R1) branches ca. ⅗ of the way through the radius length and terminates ca. ⅓ of the way through the wing length. The radial sector (Rs) branches from the end of the radius and runs angled to the wing margin where it terminates just posterior to the wing midline length. There is a weak continuation of the radius following the prominent radial sector branching which continues on as a short and thin radius to media crossvein (R–M). The media (M) is simply bifurcate with both the media anterior (MA) and media posterior (MP) terminating close to the posterior ¼ of the wing. The cubitus (Cu) runs throughout the entire wing length simply, and then near the posterior ⅕ of the wing becomes bifurcate into the cubitus anterior (CuA) and cubitus posterior (CuP) which both terminate at or very near the wing posterior apex. The first anal vein (1A) is simple and fuses with the cubitus early on, near where the radial sector branches from the radial. Alae of moderate length, reaching abdominal segment IV. *Abdomen.* Abdominal segments II through the anterior ½ of IV diverging, with the middle of segment IV the widest segment. Segments V–VII gently converging, with segment VII ending with a distinct lobe which bends inward to a notably narrower segment VIII. Segments VIII–X converging to a broad apex. *Genitalia.* Subgenital plate starts at the anterior margin of segment VIII, is broad, and only extends ⅓ of the way under segment X, ending in a fine point (Fig. 30E). Gonapophyses VIII are long (exceeding the tip of the abdomen but not as long as the tips of the cerci) and moderately broad (together side by side are about as broad as the subgenital plate; Fig. 30E). Gonapophyses IX are smaller and shorter, and mostly covered from view by the notably larger gonapophyses VIII. Cerci flat, with a granular surface throughout and few setae on the ventral surface, the dorsal surface has thin, transparent setae throughout the surface (Fig. 30E). *Legs.* Profemoral exterior lobes broad (ca. 2× width of the interior lobe) and approximately right angled (Fig. 30A). Proximal edge of the exterior profemoral lobe gently undulates giving this margin a slightly wavy appearance, whereas the distal margin is nearly straight, both margins have notable serration throughout their lengths (Fig. 30A). Profemoral interior lobe with a slightly obtuse angle and marked with five large, serrate teeth with looping gaps between them. These teeth are arranged somewhat into a two-one-two pattern with the gaps between these sets larger. Mesofemoral exterior lobe arcs from end to end and is almost evenly weighted on both sides, but with the broadest point just off center to the distal side of the midline, and on the distal half only marked with three small serrate teeth. Interior mesofemoral lobe arcs end to end, is ca. ¼ narrower than the exterior lobe, not as strongly angled, and marked with six or seven teeth on the distal half. Metafemoral interior lobe arcs end to end but is notably wider on the distal ⅔ of the lobe and this wider portion is marked by 11 or 12 serrate teeth. Metafemoral exterior lobe is thin and smooth, hugging the metafemoral shaft and marked with one or two small, rounded teeth on the distal edge. Protibiae lacking an exterior lobe. Protibiae interior lobe spans the entire length of the protibiae as a rounded scalene triangle with the widest portion on the distal ⅓. Mesotibiae and metatibiae simple, lacking exterior and interior lobes.

***Measurements of holotype female* [mm].** Length of body (including cerci and head, excluding antennae) 94.5, length/width of head 7.8/7.5, antennae 5.0, pronotum 6.6, mesonotum 7.9, length of tegmina 59.1, length of alae 31.6, greatest width of abdomen 41.3, profemora 20.7, mesofemora 16.5, metafemora 21.2, protibiae 14.0, mesotibiae 12.1, metatibiae 16.4.

###### Etymology.

Noun, Greek in origin. Relating to the tenth labor of Heracles (apparently a favorite story of Gray (1843) as four of his therein described species came from this myth) in which Heracles was tasked with capturing the red cattle from the monster Geryon (for which Gray named *Phylliumgeryon* Gray, 1843). Before Heracles sailed out to the island Erythia where Geryon and his red cattle lived, he rested on the mainland and forgot to tie up his horses. When he eventually found them, they were in a cave with an Echidna (a monster which is described as women from the waist up and snake on her lower half). She refused to give back his horses unless he lay with her, which being a classic Greek hero, he did. Before he left the Echidna, she told him that she was pregnant with three of his sons and asked him which of the three should rule her lands one day. Heracles then left her with his bow and a girdle, and told her that whichever of his three sons could draw the bow and wear the girdle best would inherit her land and the other two should be banished. Those three sons were Agathyrsus, Gelonus, and Scythes (for which Gray named *Phylliumagathyrsus* Gray, 1843; *Phylliumgelonus* Gray, 1843; and *Phylliumscythe* Gray, 1843).

We felt that this species could help to finish telling the story which Gray was so fond of. With *Phylliumgeryon* being a species from the Philippines, one biogeographical bridge for species to the Philippines is from Sulawesi through the Sangihe Islands (Evans et al. 2003), therefore as a steppingstone to the Philippines we felt there should be an Echidna along the route to the Philippines where *Phylliumgeryon* can be found.

##### Cryptophyllium faulkneri

Taxon classificationAnimalia

gen. et sp. nov.

C8E17D58-FF8F-562D-AD93-1973898C355E

http://zoobank.org/30DBFBC7-7AF0-46ED-9A3F-E50D0F17E457

Figure 31

###### Material examined.

***Holotype*** ♂: “VIETNAM: Quang Ngai Province, Bato Mt. 900 m. elv: May 2015 (Coll RC 16-114)”. Molecularly sampled within our analysis. Deposited in the Montreal Insectarium (IMQC).

***Paratypes*** (2♂): 1 ♂: “Ngoc Linh, Kon Tum Prov. Vietnam, 1700 m, VI.2016, leg. Luong coll. TB-05-134’ (Coll TB) • ♂ nymph: “VIETNAM: Lam Dong, Bao Lam, Da Tom: March 2016 (Coll RC 16-236)”, molecularly sampled within our analysis (Coll RC).

###### Remarks.

This large species was immediately identified as distinct by the size and additionally by the large, prominent tubercles of the mesopleura, which are not as distinct in males of other *Cryptophyllium* gen. nov. species. Despite several expeditions to southern Vietnam by the RBINS expedition members, no possible female for this species has been located to date, despite the fact that the female is likely a very large phylliid. Hopefully future expeditions reveal the female so the morphology to congenerics can be compared.

###### Differentiation.

**Figure 31.**
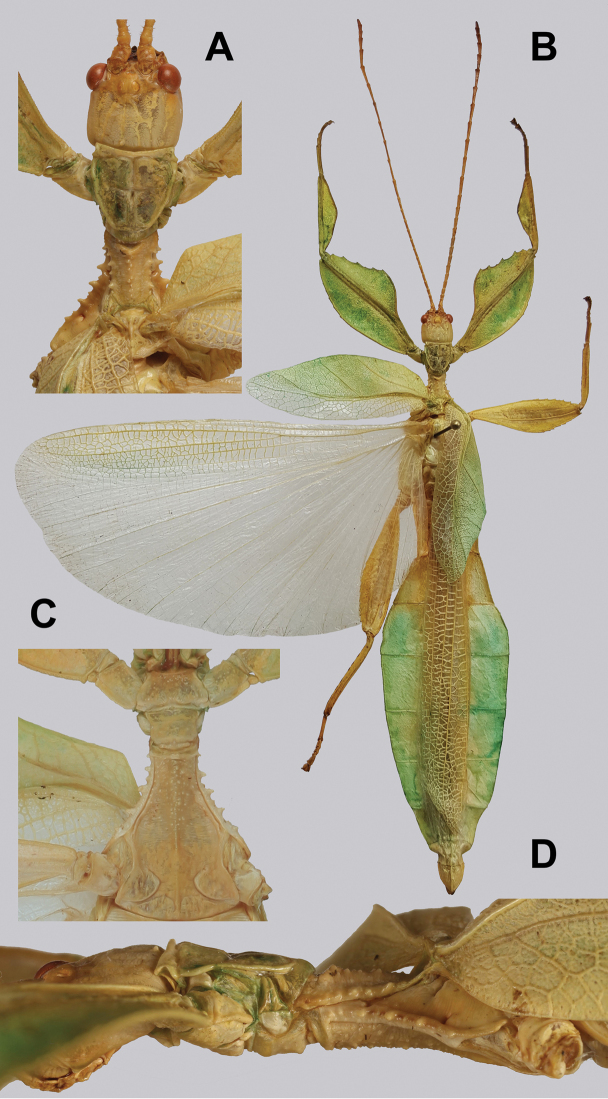
Holotype *Cryptophylliumfaulkneri* gen. et sp. nov., photographs by RTC **A** details of the base of antennae, head, and thorax, dorsal **B** habitus, dorsal **C** details of thorax, ventral **D** details of head and thorax, lateral.

Females are presently unknown. Male *Cryptophylliumfaulkneri* sp. nov. are most morphologically similar to *Cryptophylliumlimogesi* sp. nov. due to the large size and thorax spination and *Cryptophylliumanimatum* sp. nov. due to the superficially similar appearance of a slender abdomen and large overall size. *Cryptophylliumfaulkneri* sp. nov. can be differentiated from *Cryptophylliumanimatum* sp. nov. by the mesopleura spination (as *Cryptophylliumfaulkneri* sp. nov. has meseopleurae with four large protruding tubercles and five smaller interspersed nodes; Fig. 31A) vs. *Cryptophylliumanimatum* sp. nov. which has six small sized tubercles and six or seven small nodes interspersed (Fig. 10C). Additionally, *Cryptophylliumfaulkneri* sp. nov. has a rather average interior profemoral lobe serration pattern with irregular sized and spaced teeth (Fig. 31B), vs. *Cryptophylliumanimatum* sp. nov. which has teeth all evenly spaced and sized (Fig. 10B). *Cryptophylliumfaulkneri* sp. nov. can be differentiated from *Cryptophylliumlimogesi* sp. nov. by the narrower abdomen (rather broad and spade-shaped in *Cryptophylliumlimogesi* sp. nov.), a lack of small exterior tibial lobes (as *Cryptophylliumlimogesi* sp. nov. has distinct small exterior tibial lobes), and the interior protibial lobe shape which in *Cryptophylliumlimogesi* sp. nov. is weighted towards the anterior ⅓ in a scalene triangle, vs. *Cryptophylliumfaulkneri* sp. nov. which has the lobe evenly weighted with the widest portion in the middle as an isosceles triangle.

###### Distribution.

Presently only known from central and southern Vietnam, no other specimens are presently known. The two Vietnamese provinces that this species is known from are Quang Ngai and Lam Dong Provinces.

###### Male.

***Coloration.*** Coloration description based on the dried holotype specimen, not on living individuals which are likely a more vibrant green. Overall coloration is pale green with variable patches of yellow throughout due to the drying of the specimen (Fig. 31B). Compound eyes are rust red (Fig. 31A). There are no hints of natural brown coloration on the holotype and there are no eye spots present on the abdomen.

***Morphology.****Head.* Head capsule slightly longer than wide, with a vertex that lacks granulation; posteromedial tubercle notable as the only feature on the posterior half of the head capsule (Fig. 31A). Frontal convexity stout, with a somewhat broad point, and with numerous short setae throughout the surface. Compound eyes large but not overly bulbous, occupying slightly > ⅓ of the head capsule lateral margins (Fig. 31A). Between the compound eyes are three well-developed ocelli. Antennal fields are slightly wider than, and approximately as long as the scapus. *Antennae.* Antennae (including the scapus and pedicellus) consists of 28 segments. The scapus and pedicellus are mostly bare but with few very short setae throughout, segments III–XXV are covered in dense, thin, pale setae that are as long as or longer than the antennae segment is wide. The terminal three segments have dense short setae throughout that are notably shorter than the segments are wide. *Thorax.* Pronotum with anterior margin gently concave and lateral margins that are slightly convex and converge to a straight posterior margin that is ca. ½ the width of the anterior rim (Fig. 31A). Anterior and lateral margins of the pronotum have distinct rims and the posterior margin lacks a rim (Fig. 31A). Face of the pronotum is marked by a smooth surface, distinct sagittal furrow on the anterior half, a pit just posterior to the center, and a moderate perpendicular furrow just anterior to the central pit (Fig. 31A). Prosternum only has minimal small granulation on a mostly smooth surface, the mesosternum surface on the anterior half is heavily granulose and the posterior half is wrinkled to smooth (Fig. 31C). The metasternum lacks granulation but has a smooth wrinkled surface throughout. Prescutum narrow, longer than wide, with lateral margins that are straight and marked with four or five large smooth tubercles of almost equal size with few smaller nodes interspersed between them, spaces between the prominent tubercles relatively smooth (Fig. 31A).The surface of the prescutum is slightly raised along the sagittal plane which is marked with nine or ten, smooth nodes of slightly varying size, with the remainder of the prescutum surface lacking nodes, but with a slightly wrinkled surface instead (Fig. 31A). Prescutum anterior rim with a slightly lumpy surface and a moderately formed central tubercle that rises above the prescutum slightly (Fig. 31A). Mesopleura narrow on their anterior ⅓, then gradually diverging with straight margins on the posterior ⅔ (Fig. 31A). Lateral margin with five or six large protruding tubercles and five or six smaller nodes interspersed throughout (Fig. 31A). Face of the mesopleura smooth but slightly wrinkled and with two distinct pits, one near the anterior and one on the posterior ⅓. *Wings.* Tegmina moderate length, extending ½ through abdominal segment III. Tegmina venation: the subcosta (Sc) is the first vein, is simple, and terminates the earliest ca. ⅖ of the way through the overall tegmina length. The radius (R) spans the entire length of the tegmina with the first radius (R1) branching just proximal to the midline and terminating just distal to the midline, followed by the branching and termination of the second radius (R2) near the distal ⅓ of the wing, and then the radial sector runs to the wing apex. The media (M) also spans the entire length of the tegmina with the first media posterior (MP1) branching off just proximal to the midline, and then the second media posterior (MP2) branches just posterior to the midline, and the media anterior (MA) runs to the wing apex. The cubitus (Cu) runs along the edge of the wing as the two media posterior veins fuse with it and as the cubitus reaches the apex it fades. The first anal (1A) vein terminates upon reaching the cubitus ca. ⅓ of the way through the wing length. Alae well-developed in an oval fan configuration, long, reaching ⅔ of the way through abdominal segment VIII. Alae venation: the costa (C) is present along the entire foremargin giving stability to the wing. The subcosta (Sc) is long, spanning slightly < ⅔ of the wing length and is fused with the radius in the beginning but terminates when it meets the costa. The radius (R) spans the entire wing and branches ca. ⅖ of the way through into the first radius (R1) and radial sector (Rs) which run gently diverging for most of their length and then converge at the apex of the wing where they terminate near each other but not touching. The media (M) branches early, ca. ⅙ of the way through the wing into the media anterior (MA) and the media posterior (MP) which run parallel with each other throughout the wing until the distal quarter of the wing where the media posterior fuses with the media anterior which then run fused together to the wing apex where they terminate near the radial sector. The cubitus (Cu) runs unbranched and terminates at the wing apex. Of the anterior anal veins, the first anterior anal (1AA) fuses with the cubitus near the point where the media branches into the media anterior and media posterior and then the first anterior anal branches from the cubitus ⅔ of the way through the wing length where it uniformly diverges from the cubitus until it terminates at the wing margin. The anterior anal veins two–seven (2AA–7AA) have a common origin and run unbranched in a folding fan pattern of relatively uniform spacing to the wing margin. The posterior anal veins (1PA–6PA) share a common origin separate from the anterior anal veins and run unbranched to the wing margin with slightly thinner spacing than the anterior anal veins. *Abdomen.* Abdomen is a narrow dagger-like shape. Abdominal segment II with parallel-sided margins, III with gently diverging margins, the anterior half of segment IV gently diverging to the widest point of the abdomen. The remainder of segment IV through the apex are converging uniformly with straight margins to the apex. *Genitalia.* Poculum broad and rounded, ending in a straight margined apex that passes onto segment X. Cerci not exceptionally long, with only ca. ½ of their length protruding from under the terminal abdominal segment. The cerci are relatively flat, not strongly cupped, covered in a granulose surface and numerous short setae throughout. Vomer broad and stout with straight sides evenly converging to the apex which is armed with two upwards turning hooks. *Legs.* Profemoral exterior lobe a rounded arc, about as wide as the interior lobe (ca. 3× the width of the profemoral shaft at its widest), and with the anterior half marked by five small, rounded teeth (Fig. 31B). Profemoral interior lobe an obtusely rounded triangle with six, serrate, anteriorly pointing teeth arranged in pairs with looping gaps between them (in the holotype the middle pair of teeth are uneven in size, one notably larger than the other; Fig. 31B). Mesofemoral exterior lobe arcs end to end but with the widest portion slightly distal to the midline, and the widest point slightly wider than the interior lobe. The mesofemoral exterior lobe is marked with six or seven small serrate teeth distal to the widest point, with the proximal portion of the lobe smooth. The mesofemoral interior lobe is approximately the same width as the mesofemoral shaft with five or six small serrate teeth on the distal half only. Metafemoral exterior lobe thin, lacks dentation, and has a straight margin along the metafemoral shaft. Metafemoral interior lobe smoothly arcs end to end with nine or ten small serrate teeth on the distal half only. Protibiae lacking exterior lobe, interior lobe reaching end to end in a smooth evenly weighted triangle two and a half times as wide as the protibial shaft (Fig. 31B). Meso- and metatibiae simple, lacking lobes.

***Measurements of holotype male* [mm].** Length of body (including cerci and head, excluding antennae) 87.0, length/width of head 5.4/4.9, antennae 49.4, pronotum 4.4, mesonotum 5.9, length of tegmina 27.3, length of alae 60.8, greatest width of abdomen 19.7, profemora 19.5, mesofemora 17.4, metafemora 20.0, protibiae 12.9, mesotibiae 10.7, metatibiae 14.0.

###### Etymology.

Patronym. Dedicated to David Faulkner, California, United States. Forensic entomology mentor to RTC and dear friend.

##### Cryptophyllium icarus

Taxon classificationAnimalia

gen. et sp. nov.

875D19DB-F600-5EC7-8679-3DEA37E816C1

http://zoobank.org/BFC89435-7C71-4751-B404-CACE95B79616

Figures 5H , 8I , 8J , 9I , 32 , 33 , 34 , 35 , 36

###### Material examined.

***Holotype*** ♂: “Coll. I.R.Sc.N.B., Ex breeding Tim Bollens, 2018, Coll. I.R.Sc.N.B., Vietnam, Lam Dong prov., Bidoup-Nui Ba N.P., 12°26’N, 108°30’E, 21-25.vii.2014, Leg. J. Constant and J. Bresseel, GTI Project, I.G.: 32.779”. Deposited in the Royal Belgian Institute of Natural Sciences (RBINS).

***Paratypes***: (26 ♀♀, 49 ♂♂, 78 eggs) • 1 ♂: “Coll. I.R.Sc.N.B., Ex breeding Tim Bollens, 2018, Coll. I.R.Sc.N.B., Vietnam, Lam Dong prov., Bidoup-Nui Ba N.P., 12°26’N, 108°30’E, 21-25.vii.2014, Leg. J. Constant and J. Bresseel, GTI Project, I.G.: 32.779” [vomer dissected] (RBINS) • 5 ♀♀, 4 ♂♂: “Coll. I.R.Sc.N.B., Ex breeding Tim Bollens, 2018, Coll. I.R.Sc.N.B., Vietnam, Lam Dong prov., Bidoup-Nui Ba N.P., 12°26’N, 108°30’E, 21-25.vii.2014, Leg. J. Constant and J. Bresseel, GTI Project, I.G.: 32.779” (RBINS) • 1 ♀, 1 ♂: “Coll. I.R.Sc.N.B., Ex breeding Tim Bollens, 2018, Coll. I.R.Sc.N.B., Vietnam, Lam Dong prov., Bidoup-Nui Ba N.P., 12°26’N, 108°30’E, 21-25.vii.2014, Leg. J. Constant and J. Bresseel, GTI Project, I.G.: 32.779” (VNMN) • 1 ♀, 5 ♂♂: “Coll. I.R.Sc.N.B., Ex breeding Tim Bollens, 2019, Coll. I.R.Sc.N.B., Vietnam, Lam Dong prov., Bidoup-Nui Ba N.P., 12°26’N, 108°30’E, 21-25.vii.2014, Leg. J. Constant and J. Bresseel, GTI Project, I.G.: 32.779” (RBINS) • 1 ♀, 1 ♂: “Coll. I.R.Sc.N.B., Ex breeding Bruno Kneubühler, 2017, Coll. I.R.Sc.N.B., Vietnam, Lam Dong prov., Bidoup-Nui Ba N.P., 12°26’N, 108°30’E, 21-25.vii.2014, Leg. J. Constant and J. Bresseel, GTI Project, I.G.: 32.779” (RBINS) • 6 ♀♀, subadult ♀, ♂: “Coll. I.R.Sc.N.B., Vietnam, Lam Dong prov., Bidoup-Nui Ba N.P., 12°26’N, 108°30’E, 21-25.vii.2014, Leg. J. Constant and J. Bresseel, GTI Project, I.G.: 32.779” (RBINS) • 1 ♂; “Vietnam, Lam Dong, Bao Loc” (molecular sample SLT005 within our analysis) (Coll SLT) • 3 ♂♂; “ex Zucht F. Hennemann 2019, Herkunft: Z-Vietnam, Lâm Dông Prov., Dam Rông, Bidoup Nui Ba NP, leg. Bresseel and Constant VII.2014 [ex coll. FH]” (Coll SLT) • 1 ♀; “VIETNAM, Lam Dong Prov., Dam Rong, Bidoup-Nui Ba NP, Leg. Bresseel and Constant VII.2014. Ex breeding F. Hennemann 2015-2017. Ex coll. F. H. Hennemann (Germany); Coll RC 18-226” (Coll RC) • 4 ♂♂; “ex Zucht F. Hennemann 2019, Herkunft: Z-Vietnam, Lâm Dông Prov., Dam Rông, Bidoup Nui Ba NP, leg. Bresseel and Constant VII.2014 [ex coll. FH]”, Coll RC 20-099–20-102 (Coll RC) • 1 ♂; “VIETNAM, Dak Lak Prov., May 2018. Coll RC 18-408” (Coll RC) • 1 ♂; “VIETNAM, Lam Dong Prov., Dam Rong, Bidoup-Nui Ba NP, Leg. Bresseel and Constant VII.2014. Ex breeding F. Hennemann 2015-2017. Ex coll. F. H. Hennemann, Germany; Coll RC 18-225” (Coll RC) • 1 ♂; “VIETNAM, Lam Dong Prov., Bao Loc, October 2016, Coll RC 16-248” (Coll RC) • 1 ♂; “VIETNAM, Lam Dong Prov., Bao Loc, May 2016, Coll RC 17-264” (Coll RC) • 3 ♂♂; “VIETNAM, Lam Dong Prov., Bao Loc, July 2017”, Coll RC 17-266, 17-267, 17-268 (Coll RC) • 1 ♂; “VIETNAM, Lam Dong Prov., Bao Loc, June 2017. Coll RC 17-265” (Coll RC) • 2 eggs: “VIETNAM, Lam Dong Prov., Dam Rong, Bidoup-Nui Ba NP, Leg. Bresseel and Constant VII.2014. Ex breeding F. Hennemann 2015-2017. Ex coll. F. H. Hennemann, Germany”; Coll RC 18-238 and 18-239 (Coll RC) • 20 eggs: “VIETNAM: Lam Dong Prov., Bidoup Nui Ba N.P., culture from Maxime Ortiz (France), 2018”. Coll RC 18-343–18-362 (Coll RC) • 7 ♀♀, 1 ♂; “VIETNAM: Lam Dong Prov., Bidoup Nui Ba N.P., bred by Bruno Kneubühler (Switzerland), circa 2016” (Coll OC) • 3 ♀♀, 7 ♂♂, 28 eggs: “ex Zucht F. Hennemann 2018, Herkunft: Z-Vietnam, Lâm Dông Prov., Dam Rông, Bidoup Nui Ba NP, leg. Bresseel and Constant VII.2014” [coll. FH, No’s 0896-1 to 10, E1] (Coll FH) • 1 ♀, 7 ♂♂, 1 ♂ nymph n5, 28 eggs; “ex Zucht F. Hennemann 2019, Herkunft: Z-Vietnam, Lâm Dông Prov., Dam Rông, Bidoup Nui Ba NP, leg. Bresseel and Constant VII.2014” [coll. FH, No’s 0896-11 to 19, E2], (Coll FH) • 1 ♀: “ex Zucht F. Hennemann 2020, Herkunft: Z-Vietnam, Lâm Dông Prov., Dam Rông, Bidoup Nui Ba NP, leg. Bresseel and Constant VII.2014” [coll. FH, No’s 0896-20], (Coll FH) • 3 ♀♀, 4 ♂♂; “Vietnam, Lâm Dông Prov., Dam Rông, Bidoup Nui Ba, bred by Maxime Ortiz, France, circa 2020” (Coll MO).

###### Remarks.

**Figure 32.**
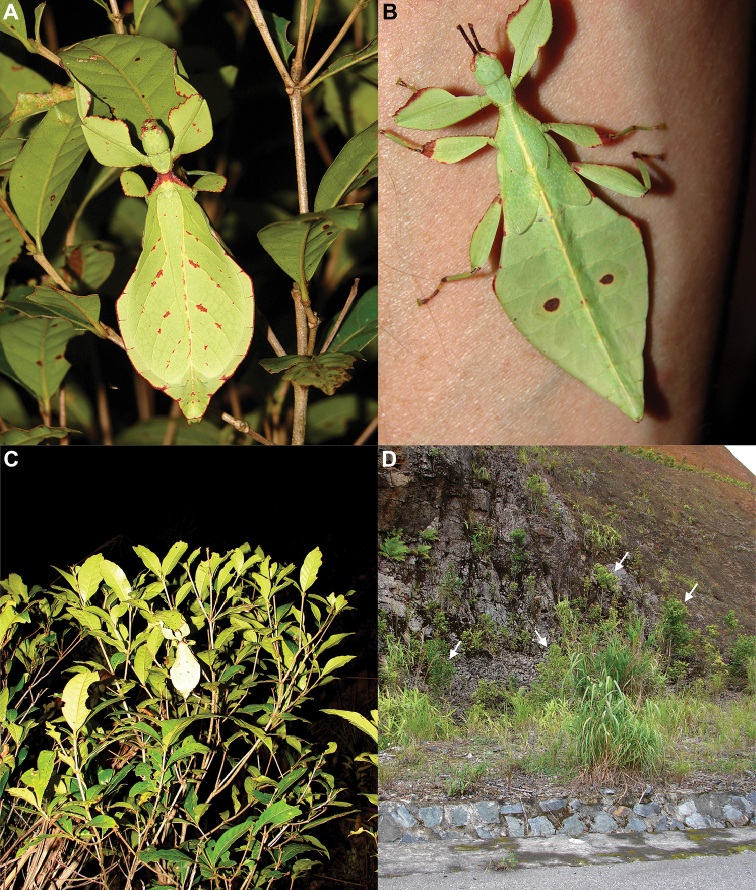
Live *Cryptophylliumicarus* found in July 2014 by Joachim Bresseel (RBINS) and Jérome Constant (RBINS) in Bidoup Nui Ba N.P., Vietnam. Photographs by Jérome Constant (RBINS) **A** paratype female, dorsal closeup **B** male nymph, paratype **C** same female as in A but showing the host tree she was found on **D** disturbed area along the road where the original stock was found with arrows pointing out host trees.

**Figure 33.**
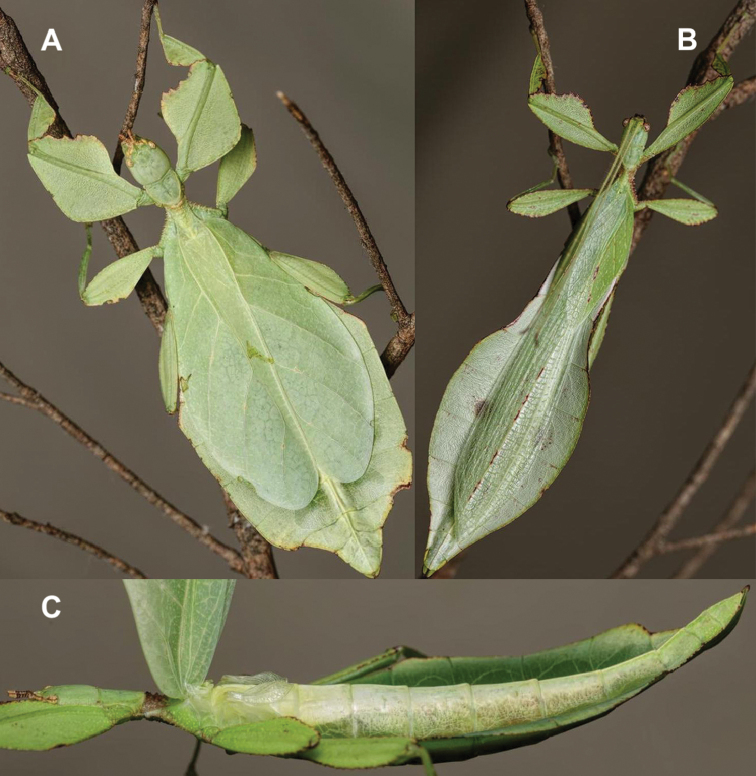
Live adult *Cryptophylliumicarus* bred and photographed by Bruno Kneubühler (Switzerland) **A** female **B** male **C** lateral view of female with tegmina held up to expose the dwarfed alae underneath.

*Cryptophylliumicarus* sp. nov. was first collected in 2014 by Joachim Bresseel (RBINS) and Jérome Constant (RBINS) from Bidoup Nui Ba N.P., Vietnam. Several adult females and a subadult male nymph were found on small trees between the side of the road and next to a high cliff with primary forest on top (Fig. 32C, D). This species was successfully brought into the phasmid breeding community which allowed observation of morphological variation in the females and description of the adult male, egg, and freshly hatched nymph morphology (Fig. 33). Interestingly, a significant change in abdominal shape occurred when this species was reared in captivity vs. the wild collected females. The wild collected paratype females (Fig. 32A) has a slenderer abdomen, with segments VI and VII notably converging towards the posterior with only a slight bend near the posterior of segment VII, vs. individuals reared in captivity (Fig. 33A) which instead develop a rather boxy abdominal shape, with segments VI and the majority of VII parallel-sided and ending in a distinct lobe on the posterior of VII. Interestingly all wild caught females were slender and all bred females have been boxy; at this time we are unsure what mechanism is promoting this polymorphism within this species. Hopefully additional field work in the region will reveal additional observations to review.

An additional unique feature for this species is the coloration of the freshly hatched nymphs, which do not match well with the coloration of other known congenerics. This could be due to our lack of knowledge as freshly hatched nymph coloration is known only for nine species (Fig. 9). Molecularly, we found *Cryptophylliumicarus* sp. nov. to be sister species to *Cryptophylliumlimogesi* sp. nov. whose freshly hatched nymph morphology we do not know. It is possible that these may share similar coloration, but at the present *Cryptophylliumicarus* sp. nov. with their solid red abdomen and dark extremities (Fig. 9I) appear quite unique within the *Cryptophyllium* gen. nov. as presently known.

###### Differentiation.

Female morphology is superficially similar to *Cryptophylliumdaparo* sp. nov. and *Cryptophylliumchrisangi* comb. nov. due to the tapered abdominal shape, obtusely rounded profemoral exterior lobes, and similarly shaped and textured thorax. *Cryptophylliumicarus* sp. nov. can be differentiated from both species by the highly reduced alae not reaching abdominal segment II (Fig. 33C), vs. the other species which both have well-developed alae.

Males with their thorax shape and spination, the spade-shaped abdomen, shorter tegmina, and exterior profemoral lobe which is thinner than the interior, are morphologically similar to *Cryptophylliumbankoi* sp. nov. and *Cryptophylliumrarum* comb. nov. males. From both species, *Cryptophylliumicarus* sp. nov. can be differentiated by the width of the profemoral lobe which in *Cryptophylliumicarus* sp. nov. is only ca. 2× as wide as the profemoral shaft, vs. the other species which are at least 2½ or 3× as wide as the profemoral shaft. Additionally, males of *Cryptophylliumrarum* comb. nov. are notably larger than the largest *Cryptophylliumicarus* sp. nov. males.

###### Distribution.

Only presently known from southern Vietnam, from the provinces of Lam Dong and Dak Lak.

###### Description.

**Female. *Coloration.*** Coloration is variable, as when it was bred in captivity females were almost uniformly pale green, with only slightly orange/tan areas along the profemoral lobes, antennae, eyes, thorax, and terminal abdominal margins (Fig. 33A). In contrast, the paratype female when she was found in the wild was quite colorful with a vibrant green body with highlights of red throughout the antennae, legs, thorax, tegmina, and abdomen (Fig. 32A), much darker and more plentiful than on captive bred specimens.

**Figure 34.**
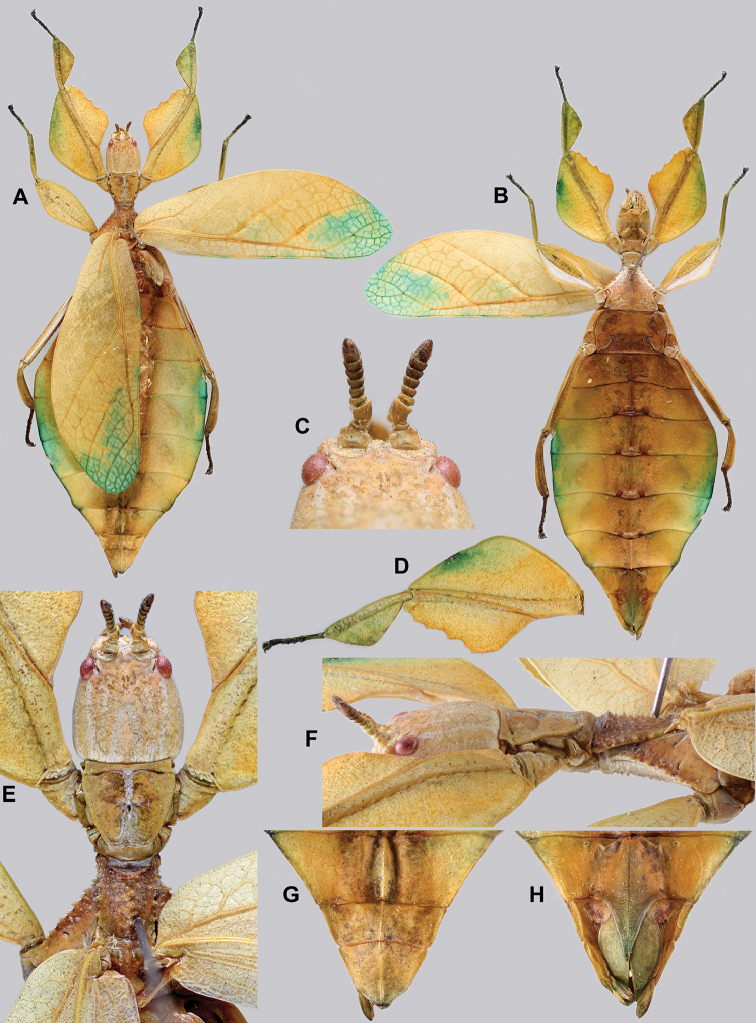
*Cryptophylliumicarus* gen. et sp. nov. female paratype, photographs by Jérôme Constant (RBINS) **A** habitus, dorsal **B** habitus, ventral **C** antennae and anterior of head capsule **D** profemoral and protibial lobes **E** head and thorax details dorsal **F** head and thorax details lateral **G** genitalia dorsal **H** genitalia ventral.

***Morphology.****Head.* Head capsule longer than wide, vertex relatively smooth, with the only notable feature the posteromedial tubercle which is not notably broad but is significantly raised above the head capsule (Fig. 34E). Frontal convexity broad and stout, with a lumpy surface, and with several setae throughout (Fig. 34E). Compound eyes slightly protruding from the head capsule, not notably large, only taking up slightly < 1/4 of the length of the lateral head capsule margins (Fig. 34E). Ocelli absent. Antennal fields slightly wider than the first antennomere (Fig. 34C). *Antennae.* Antennae consisting of nine segments, with the terminal segment about as long as 2½× the preceding segments lengths (Fig. 34C). Antennomeres I–VIII sparsely marked with small transparent setae, the terminal antennomere and the distal margin of antennomere VIII has darker, shorter, and denser setae than the other segments (Fig. 34C). *Thorax.* Pronotum with a slightly concave anterior margin and slightly convex lateral margins, which converge to a straight posterior margin that is half the width of the anterior margin (Fig. 34E). The pronotum surface and moderately formed pronotum rims are only slightly lumpy, lacking significant granulation, with only a prominent pit in the center, and slight furrows anterior, posterior, and lateral to the pit (Fig. 34E). Prosternum with moderate granulation, mesosternum anterior half and lateral margins with moderate granulation (Fig. 34B). Metasternum relatively smooth, lacking notable nodes. Prescutum slightly longer than wide, lateral rims with four larger nodes and four or five nodes interspersed throughout (Fig. 34E). Prescutum anterior rim prominent but not strongly protruding, surface marked throughout with irregular granulation, no prominent singular sagittal spine present (Fig. 34F). Prescutum surface covered densely by small tubercles and numerous nodes, with those along the sagittal plane the largest (Fig. 34E). Mesopleura beginning slightly posterior to the anterior margin of the prescutum and evenly diverging; lateral margin with five larger tubercles, and five or six smaller tubercles interspersed unevenly throughout (Fig. 34E). Face of the mesopleura slightly wrinkled, with a distinct pit near the anterior margin and one near the center (Fig. 34E). *Wings.* Tegmina long, reaching ½ way through abdominal segment VII. The subcosta (Sc) is the first vein in the forewing and runs parallel with the tegmina lateral margin for the first half of the vein, then bends gently and runs to the to the lateral margin of the wing where it terminates ca. ⅓ through the length. The radius (R) spans the central portion of the forewing with two subparallel branched veins; radius 1 (R1) terminates slightly proximal to the midline, and the radial sector (Rs) terminates ca. ⅔ of the way through the wing length. There is a weak continuation of the radius following the prominent Rs branching which continues on as a short and thinner R–M crossvein that does not solidly connect the two veins as it reaches the media. The media (M) is simply bifurcate with both the media anterior (MA) and media posterior (MP) terminating on the posterior ¼ of the wing. The cubitus (Cu) is also bifurcate, branching near the posterior ⅕ of the wing into the cubitus anterior (CuA) and cubitus posterior (CuP) which both terminate at or very near the wing posterior apex. The first anal vein (1A) is simple and fuses with the cubitus early on, halfway between the branching of the R1 and the Rs. Alae are highly reduced, not reaching the anterior margin of abdominal segment II. *Abdomen.* Abdominal segments II through the anterior half of IV diverge uniformly towards the posterior. The posterior half of IV and segment V are parallel-sided and are the widest portion of the abdomen. Segment VI and VII are variable depending on the environmental conditions, with wild collected females (such as in the paratype; Fig. 32A) having segments VI and VII strongly converging and VII ending with a small bulge on the posterior margin with a final width about the same as the anterior margin of segment VIII, vs. captive bred individuals generally having VI and VII subparallel and ending with VII having a distinct lobe which is notably wider than the anterior margin of segment VIII (Fig. 33A). Segments VIII–X converge uniformly to the rounded apex. *Genitalia.* Subgenital plate starts at the anterior margin of segment VIII, is long and narrow reaching ca. ½ onto segment X (Fig. 34H). Gonapophyses VIII are long and significantly broad, each about as wide as the subgenital plate projection, with their tips reaching the apex of segment X, gonapophyses IX are smaller and slender, hidden below the gonapophyses VIII (Fig. 34H). Cerci flat, not strongly cupped, with a heavily granular surface and a setae throughout (Fig. 34H). *Legs.* Profemoral exterior lobes notably wider than the interior lobe with a rounded obtuse angle (Fig. 34D). Exterior lobe margin is not marked by teeth and is instead rather smooth or at most slightly granular (Fig. 34D). Profemoral interior lobe ca. 2× as wide as the greatest width of the profemoral shaft, with an obtuse angle, and marked with five prominent teeth arranged in a two-one-two pattern with large looping gaps between the teeth (Fig. 34D). Mesofemoral exterior lobe arcs from end to end in a slightly bent lobe slightly weighted to the distal ½ and marked with two or three small serrate teeth on the distal ½ only. Interior and exterior lobes are of a similar width, or the exterior is slightly wider. Mesofemoral interior lobe arcs smoothly end to end, is marked with six or seven serrate teeth only on the distal half of the arc, and is about as wide as the mesofemoral shaft. Metafemoral interior lobe arcs end to end, but is slightly wider on the distal half, and has six or seven serrate teeth on the distal half of the lobe only. Metafemoral exterior lobe is thin and smooth, hugging the metafemoral shaft and lacks dentation. Protibiae interior lobe spans the entire length of the protibiae and is at ca. 2× as wide as the protibial shaft. The lobe is distinctly triangular with the broadest point distal to the midline (Fig. 34D). Pro-, meso-, and meta- tibiae lacking exterior lobes; meso-, and meta- tibiae lack interior lobes as well.

***Measurements of paratype females* [mm].** Length of body (including cerci and head, excluding antennae) 81.8–94.1, length/width of head 8.0–9.7/6.4–7.4, antennae 3.3–4.2, pronotum 4.6–5.7, mesonotum 6.7–9.1, length of tegmina 45.4–57.5, length of alae 6.0–7.2, greatest width of abdomen 30.7–34.4, profemora 17.9–21.6, mesofemora 13.8–15.9, metafemora 16.2–18.6, protibiae 11.1–11.8, mesotibiae 10.1–10.7, metatibiae 13.6–13.7.

**Male. *Coloration.*** Coloration description based on live captive reared males (Fig. 33B) and the male nymphs found in the wild (Fig. 32B). Captive males are generally mostly pale green throughout with the margins of the legs, thorax, and abdomen a reddish brown, and slightly transparent eye spots on abdominal segment V. The wild caught nymphs had these areas typically darker, more dark red than reddish brown and dark eyespots on abdominal segment V.

**Figure 35.**
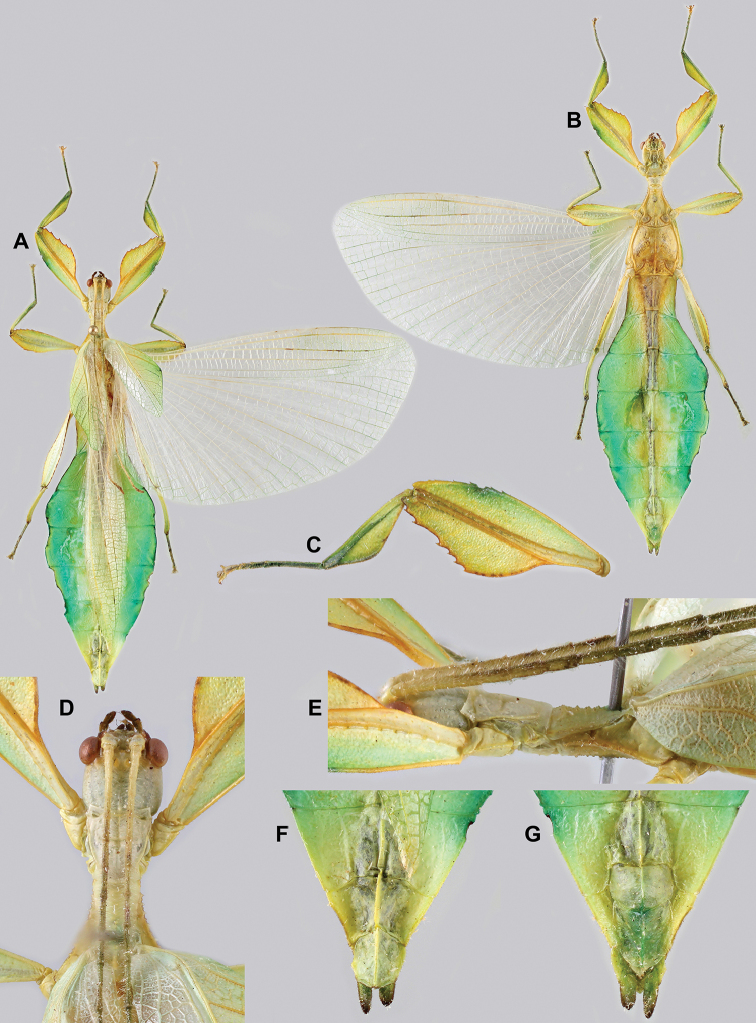
*Cryptophylliumicarus* gen. et sp. nov. male holotype, photographs by Jérôme Constant (RBINS) **A** habitus, dorsal **B** habitus, ventral **C** profemoral and protibial lobes **D** head and thorax details dorsal **E** head and thorax details lateral **F** genitalia dorsal **G** genitalia ventral.

***Morphology.****Head.* Head capsule about as long as wide, with a vertex that is weakly granular; posteromedial tubercle small but notable and slightly raised above the head capsule (Fig. 35D). Frontal convexity stout with a few short setae near the apex. Compound eyes large and bulbous, occupying ca. ⅖ of the head capsule lateral margins and significantly protruding from the head capsule (Fig. 35D). There are three well-developed ocelli between and slightly posterior to the compound eyes. Antennae (including the scapus and pedicellus) consists of 23 segments. The scapus and pedicellus are bare, all other segments are covered in dense, thin, pale setae that are as long as or longer than the antennae segment is wide. The terminal three segments have shorter darker setae. *Thorax.* Pronotum anterior margin is distinctly concave and lateral margins are slightly convex and converge to a straight posterior margin that is slightly > ½ width of the anterior rim (Fig. 35D). Anterior and lateral margins of the pronotum have moderate rims and the posterior margin lacks a rim (Fig. 35D). Face of the pronotum is slightly lumpy, has a distinct sagittal furrow, a pit just posterior to the center, a moderate perpendicular furrow just anterior to the central pit, and has a distinct pit on each side near the anterior margin (Fig. 35D). The prosternum surface is slightly granular. The mesosternum surface is marked densely with prominent nodes, with the largest along the sagittal plane and more prominent on the anterior margin, posterior margin with less prominent and slightly smaller nodes. Prescutum slightly longer than wide, with lateral margins slightly converging to the posterior which is ca. ¾ the width of the anterior rim (Fig. 35D). Lateral rims with nine or ten nodes of slightly varying size, none very large or prominent, but each marked with a single stiff seta protruding from the tip. The surface of the prescutum is notably granulose along the sagittal plane with lateral surfaces rather smooth (Fig. 35D). Prescutum anterior rim slightly granulose with no distinct central tubercle (Fig. 35E). Mesopleura narrow, only gradually diverging from the anterior to the posterior (Fig. 35D). Lateral margin granulose throughout, with only four or five slightly larger than the rest, but not significantly larger. The largest nodes along the mesopleura have a singular seta protruding from them like those on the prescutum margins. Face of the mesopleura smooth but slightly wrinkled and with two faint pits, one on the anterior margin and one near the middle of the mesopleura. *Wings.* Tegmina short, only reaching the anterior margin of abdominal segment III. Tegmina wing venation: the subcosta (Sc) is the first vein and terminates the earliest, ca. ⅖ of the way through the overall tegmina length. The radius (R) spans the entire length of the tegmina with the first radius (R1) branching ca. ⅓ of the way through the wing length and terminating just posterior to the middle of the wing, the second radius (R2) branches near the distal ⅓ of the wing, and then the radial sector (Rs) runs straight to the tegmina apex and terminates. The media (M) spans the entire length of the tegmina, terminating at the wing apex as the media anterior (MA) with the first media posterior (MP1) beginning and terminating near the tegmina mid length followed by the second media posterior (MP2) which begins ca. ⅔ of the way through the tegmina length and terminates near the posterior quarter of the wing. The cubitus (Cu) runs through the wing surface angled until it meets the margin ca. ⅓ of the way through the tegmina length and then runs along the margin as the two media posterior veins then meet it and fuse and the cubitus continues to run nearly to the wing apex. The first anal (1A) vein runs subparallel to the cubitus until it meets it slightly > ⅓ of the way through the tegmina length and fuses with it. Alae well-developed in an oval fan configuration, reaching to the anterior margin of abdominal segment IX or halfway through it. Alae wing venation: the costa (C) is present along the entire foremargin giving stability to the wing. The subcosta (Sc) spans ca. ⅔ of the wing length and is mostly fused with the radius in the beginning but terminates when it meets the costa. The radius (R) spans the entire wing and branches ca. ⅖ of the way through into the radius 1 (R1) and radial sector (Rs) which run slightly diverging for the first ⅓ of their length, parallel for the central portion until the terminal quarter where they converge and terminate on the wing margin near each other but not touching. The media (M) branches early, ca. ⅙ of the way through the wing into the media anterior (MA) and the media posterior (MP) which run parallel with each other until the distal ⅙ of the wing where the media posterior fuses with the media anterior which then run fused together to the wing margin. The cubitus (Cu) runs unbranched and terminates at the wing apex. Of the anterior anal veins, the first anterior anal (1AA) fuses with the cubitus near the point where the media branches into the media anterior and media posterior and then the first anterior anal branches from the cubitus ⅔ of the way through the wing length where it uniformly diverges from the cubitus until it terminates at the wing margin. The anterior anal veins two–seven (2AA–7AA) have a common origin and run unbranched in a folding fan pattern of relatively uniform spacing to the wing margin. The posterior anal veins (1PA–7PA) share a common origin separate from the anterior anal veins and run unbranched to the wing margin with slightly thinner spacing than the anterior anal veins. *Abdomen.* Segment II, parallel-sided, segment III and the anterior half of IV diverging, the posterior ½ of IV through segment V either parallel-sided or slightly diverging, VI through the apex converging to a blunt rounded apex. The margins of segments VIII–X have a line of setae along them (Fig. 35F). *Genitalia.* Poculum broad and rounded, ending in a rounded apex that passes beyond the anterior margin of segment X (Fig. 35G). Cerci long and slender, with ca. ½ of their length extending out from under the anal abdominal segment. The cerci are slightly cupped, with a granulose surface and numerous short setae throughout (Fig. 35F). Vomer broad and stout with rounded sides converging to the apex which is armed with two upwards turning hooks, one at the apex which is larger and one lateral to it which is slightly smaller (Fig. 5H). *Legs.* Profemoral exterior lobe a rounded arc without a distinct angle, slightly thinner than the interior lobe (at its widest slightly > 2× the greatest width of the profemoral shaft), and with the distal half marked by three or four small but sharp anteriorly pointing teeth (Fig. 35). Profemoral interior lobe roundly triangular, at its widest ca. 2½× as wide as the profemoral shaft at its widest. The profemoral interior lobe is generally marked with five, serrate, anteriorly pointing teeth arranged in a two-one-two pattern, with shallow looping gaps between them, and occasionally marked with an extra tooth within the set (like as can be seen in Fig. 35C). Mesofemoral exterior lobe arcs end to end but with a more prominent bend near the distal ⅓ of the lobe which is marked with two or three small serrate teeth on the distal ⅓ only, with the proximal portion of the lobe smooth. The mesofemoral interior lobe at its widest is approximately the same width as the exterior lobe, but the proximal half is slightly wider than the proximal half of the exterior lobe. The distal half of the mesofemoral interior lobe is marked with six or seven small serrate teeth and the proximal half is rather smooth. Metafemoral exterior lobe lacks dentation and has a straight margin along the metafemoral shaft. Metafemoral interior lobe smoothly arcs end to end with six or seven small serrate teeth on the distal ½ only. Protibiae lacking exterior lobe, interior lobe reaching end to end in a smooth triangle ca. 3× as wide as the protibial shaft, with the widest point just distal to the midline, and all margins notably marked with short setae throughout (Fig. 35C). Mesotibiae and metatibiae simple, lacking lobes completely.

***Measurements of holotype male* [mm].** Length of body (including cerci and head, excluding antennae) 58.6, length/width of head 3.7/3.3, antennae 32.5, pronotum 2.8, mesonotum 4.5, length of tegmina 16.1, length of alae 42.0, greatest width of abdomen 15.8, profemora 12.0, mesofemora 9.5, metafemora 11.4, protibiae 7.5, mesotibiae 6.3, metatibiae 8.2.

***Measurements of paratype males* [mm].** Length of body (including cerci and head, excluding antennae) 56.0–69.4, length/width of head 2.9–4.6/2.9–4.0, antennae 34.5–43.9, pronotum 2.5–3.0, mesonotum 3.8–4.8, length of tegmina 16.4–19.8, length of alae 40.5–49.9, greatest width of abdomen 14.4–18.3, profemora 11.7–14.8, mesofemora 9.5–12.3, metafemora 11.0–13.6, protibiae 7.7–9.9, mesotibiae 6.5–8.0, metatibiae 8.5–10.4.

**Figure 36.**
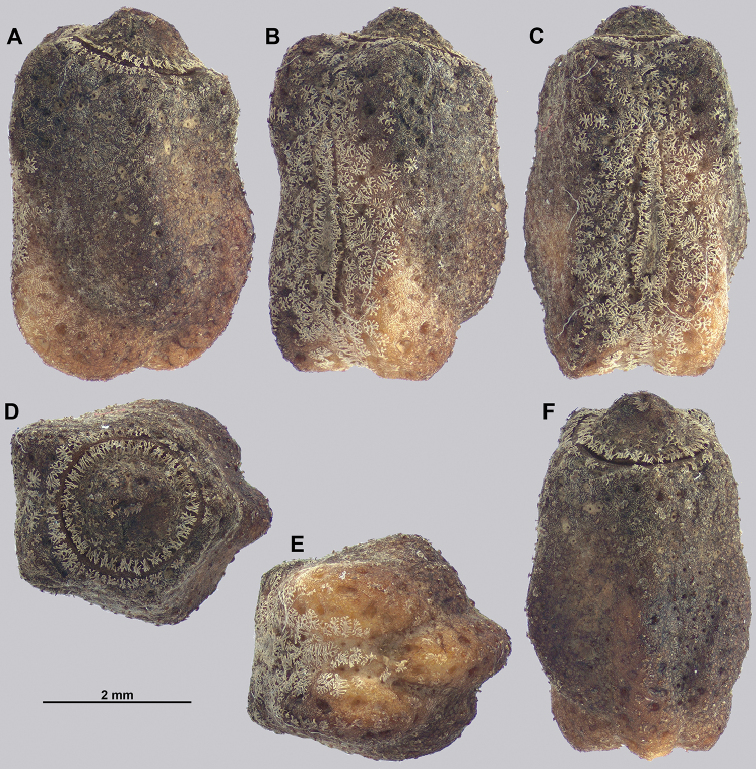
*Cryptophylliumicarus* gen. et sp. nov. egg, RBINS collection, photographs by Jérôme Constant **A** lateral view **B** dorso-lateral view **C** dorsal view **D** opercular (anterior) view **E** posterior view **F** ventral view.

**Description of egg** (Fig. 36). The lateral surfaces are flat but with the posterior half slightly wider than the anterior half. The dorsal surface is slightly convex, which gives the margin a slight undulating appearance when viewed from the lateral aspect as the middle is thinner than either end of the egg. When viewed from the lateral aspect; the ventral margin is also not straight but is instead with the posterior slightly protruding more than the anterior. All surfaces have numerous small sized pits throughout with short moss-like pinnae interspersed throughout the capsules surfaces with those on the margins and those on the dorsal surface slightly more prominent. Dorsal surface with irregular medium sized pitting and moss-like pinnae around the micropylar plate. Micropylar plate long, ca. 7/8 of the overall dorsal surface length, with the widest portion around the micropylar cup. Micropylar plate nearly symmetrical with the anterior and posterior thin and the area around the micropylar cup the widest point. Micropylar cup of moderate size and placed just slightly posterior to the micropylar plate midline. Operculum slightly ovular, with the outer margin with a distinct row of moss-like pinnae surrounding the operculum. Operculum is roundly raised with the height ca. ½ the operculum width. The overall color is light brown, with the moss-like pinnae sometimes slightly lighter in color.

###### Measurements including the extended pinnae

[**mm].** Length (including operculum) 2.2, maximum width of capsule when viewed from lateral aspect 1.3 mm, length of micropylar plate 1.1 mm.

**Newly hatched nymphs.** (Fig. 9I). The body is made up of two general blocks of color, the legs, head, pronotum, and mesonotum are primarily chocolate brown and the mesonotum and abdomen are burnt red in color. Basitarsi are white and the remaining tarsal segments are burnt reddish. The tibiae only have interior lobes which smoothly span the full length and lack exterior lobes. The tibiae interior lobes are brown with two white patches on the proximal half only, the proximal most is notably larger. Interior and exterior femoral lobes are all about the same width, are smoothly arcing, and all have minimal serration. On the profemoral interior lobe there is a notable whitish patch, the exterior lobe and profemoral shaft itself are devoid of prominent white markings. The meso- and metafemoral interior lobes are similar in that they have a small white patch right at the proximal end, and then another white marking ca. ⅖ of the way through the length. The exterior meso- and metafemoral lobes only have one white patch located on the proximal ⅓, but notably wider than the interior lobe white patch. The meso- and metafemoral shafts lack white coloration. The abdomen is slender with segment II through the anterior ½ of IV diverging and the posterior ½ of IV–X converging.

###### Etymology.

Noun, from Greek mythology. Named for the tragic story of Icarus, son of Daedalus. During their escape from the island of Crete, Icarus flew too close to the sun and melted the wax wings his father built. We felt it was fitting that this mythological name is shared with this species that lacks the hindwings within the former *celebicum* species group (characterized by females with well-developed alae).

##### Cryptophyllium khmer

Taxon classificationAnimalia

gen. et sp. nov.

7F2F01FA-3059-59E3-9E1E-C7B005EBDF4C

http://zoobank.org/118A6B83-0408-4373-8C02-3705528BB026

Figures 5B , 5D , 6D , 9C , 37 , 38 , 39 , 40 , 41

###### Material examined.

***Holotype*** ♂: “Coll. I.R.Sc.N.B., collected as nymph, Coll. I.R.Sc.N.B., Cambodia, Koh Kong prov., Tatai, 11°35’13”N 103°05’50”E, 9-19.x.2016, day collecting, GTI Project, Leg. J. Constant and J. Bresseel, I.G.: 33.345 (RBINS-PHYLLIUM DNA sample 0002)” [vomer dissected], deposited in RBINS.

***Paratypes*** (9 ♀♀, 2 ♂♂): • “Coll. I.R.Sc.N.B., collected as nymph, Coll. I.R.Sc.N.B., Cambodia, Koh Kong prov., Tatai, 11°35’13”N 103°05’50”E, 9-19.x.2016, day collecting, GTI Project, Leg. J. Constant and J. Bresseel, I.G.: 33.345, RBINS-PHYLLIUM DNA sample 0001” (RBINS) • 3 ♀♀, 1 ♂: “Coll. I.R.Sc.N.B., Ex breeding Tim Bollens, 2018, Coll. I.R.Sc.N.B., Cambodia, Koh Kong prov., Tatai, 11°35’13”N 103°05’50”E, 9-19.x.2016, day collecting, GTI Project, Leg. J. Constant and J. Bresseel, I.G.: 33.345” • 2 ♂♂: “Coll. I.R.Sc.N.B., Ex breeding Tim Bollens, 2018, Coll. I.R.Sc.N.B., Cambodia, Koh Kong prov., Tatai, 11°35’13”N 103°05’50”E, 9-19.x.2016, day collecting, GTI Project, Leg. J. Constant and J. Bresseel, I.G.: 33.345; ex breeding Tim Bollens, 2018” • 2 ♀♀: “Coll. I.R.Sc.N.B., CAMBODIA, Siem Reap Prov., Phnom Kulen N.P., Forest near Preah Thom, 26-27-VII-2006, Leg K. Smets, Y. Oul and D. Jump.” (1 ♀: RBINS; 1 ♀: RUPP) • 1 ♂: “Cambodia, Siem Reap; Kbal Spean, 13°40.858’N 104°01.111’E, 122 m, 6-jul-2015, Hap, Sour, Phauk, Khearn, Chhum, Ly, Lom, Heang, Hok, CA0028, Lighttrap in the forest with canopy cover.” (RUPP) • 3 ♀, 1 ♂: “Coll. I.R.Sc.N.B., Ex breeding Tim Bollens, 2019, Coll. I.R.Sc.N.B., Cambodia, Koh Kong prov., Tatai, 11°35’13”N 103°05’50”E, 9-19.x.2016, day collecting, GTI Project, Leg. J. Constant and J. Bresseel, I.G.: 33.345; ex breeding Tim Bollens, 2019” (RBINS) • 1 ♀, 1 ♂: “Coll. I.R.Sc.N.B., Ex breeding Tim Bollens, 2019, Coll. I.R.Sc.N.B., Cambodia, Koh Kong prov., Tatai, 11°35’13”N 103°05’50”E, 9-19.x.2016, day collecting, GTI Project, Leg. J. Constant and J. Bresseel, I.G.: 33.345; ex breeding Tim Bollens, 2018” (Coll RC).

###### Remarks.

When this species was first reviewed morphologically, it was assumed to be an additional distribution record of *Cryptophylliumwestwoodii* comb. nov., which is known from a relatively expansive range (Fig. 2). However, our molecular analysis revealed that the Tatai and Siem Reap (Cambodia) populations instead represented an undescribed species distinct from *Cryptophylliumwestwoodii* comb. nov. The recognition of this cryptic species from Cambodia, leaves many observational records> from Laos, Thailand, and Cambodia without confirmed identification (represented by the bi-colored circles in our distribution map noting a record which, due to the lack of molecular confirmation, could represent either species; Fig. 2).

**Figure 37.**
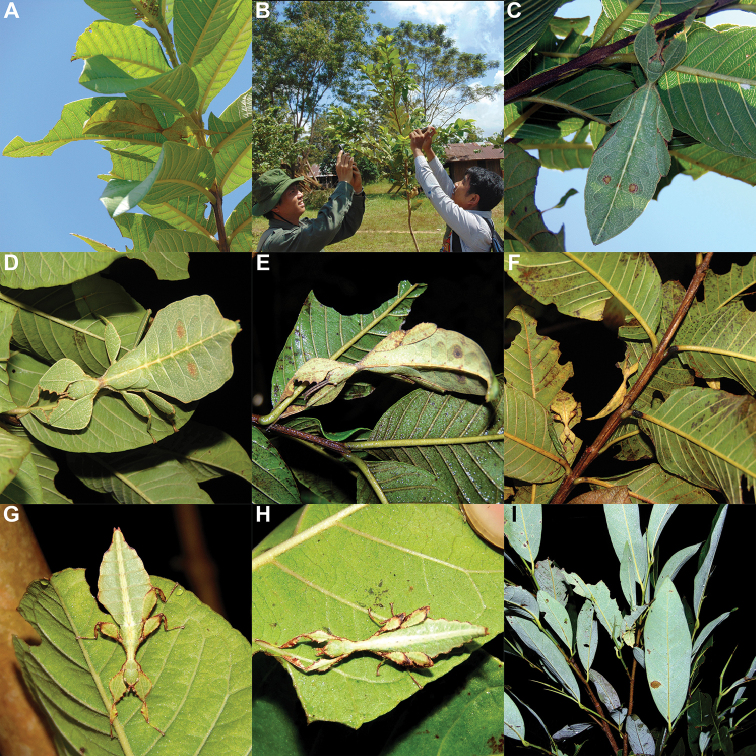
Live *Cryptophylliumkhmer* gen. et sp. nov. type material found by the joint effort of the RBINS, RUPP, and VNMN teams in Tatai, Cambodia in October 2016, all found as nymphs. Individuals in photographs **A–H** were found feeding on Guava trees (*Psidiumguajava*) and the specimen in photograph **I** was found feeding on an unidentified species. Photographs by Jérôme Constant (RBINS) **A** female **B** Hong Thai Pham (left) and Sisonila Kangsun (right), participants of the GTI project, photographing the leaf insects, which are very popular in Asia **C** male nymph **D** female nymph **E** male nymph **F** several nymphs **G** nymph **H** nymph **I** female nymph on unidentified host species.

During GTI expeditions several nymphs ranging from L1 to subadult were collected on multiple closely situated guava trees behind a house near the start of the trail leading to Tatai falls. Nymphs were successfully reared to adulthood by Tim Bollens (Belgium). Strangely locals had never noticed the insects before due to their excellent camouflage (Fig. 37).

Interestingly, in 2006, an attempt was made to describe a *Cryptophylliumwestwoodii* comb. nov. like species from Rayong, Thailand as ‘*Phylliumrayongii*’ (Sorpongpaisal and Thanasinchayakul 2006). Cumming and Le Tirant (2020) note however that this name is a nomen nudum and therefore unavailable according to ICZN Article 16.4.1. (ICZN 1999). With this population rather geographically close to the type locality of *Cryptophylliumkhmer* sp. nov. it is entirely possible that ‘*Phylliumrayongii*’ may have been intended to represent a valid population, but with the lack of a holotype specimen to define this species it was never confirmed and is now a moot point.

Due to the cryptic nature of this new species, we hope that efforts will be undertaken in the future to molecularly sample from throughout Thailand, Myanmar, Laos, and Cambodia to determine with more clarity the geographic distributions where *Cryptophylliumkhmer* sp. nov. and *Cryptophylliumwestwoodii* comb. nov. occur.

###### Differentiation.

Morphological differentiation of this species has proven to be difficult, with the only clear and consistent differences being ascertained through molecular analysis (Fig. 4).

Females are most morphologically similar to *Cryptophylliumwestwoodii* comb. nov., *Cryptophylliumbollensi* sp. nov., *Cryptophylliumphami* sp. nov., and *Cryptophylliumnuichuaense* sp. nov. females based on the general shape of the abdomen, lobes of the legs, and the thorax. The later three species can be differentiated by their shorter alae reaching to abdominal segments II or III vs. *Cryptophylliumkhmer* sp. nov. which has long alae reaching onto abdominal segment VI. We have yet to identify a reliable morphological feature between *Cryptophylliumkhmer* sp. nov. and *Cryptophylliumwestwoodii* comb. nov. as both species have long alae and at least for *Cryptophylliumwestwoodii* comb. nov. there is significant intraspecific variation which often encompasses the range of *Cryptophylliumkhmer* sp. nov. female variation.

Males are most morphologically similar to *Cryptophylliumwestwoodii* comb. nov., *Cryptophylliumchrisangi* comb. nov., *Cryptophylliumbollensi* sp. nov., and *Cryptophylliumphami* sp. nov. based on features of the thorax, tegmina, and lobes of the legs. *Cryptophylliumkhmer* sp. nov. males can be differentiated from the first two species by the general shape of the abdomen as it is thinly elliptical with a maximum width only 30–34% the abdominal length in *Cryptophylliumwestwoodii* comb. nov. and *Cryptophylliumchrisangi* comb. nov. vs. broadly elliptical or broadly spade-shaped with a maximum width ca. 38–45% the abdominal length in *Cryptophylliumkhmer* sp. nov., *Cryptophylliumbollensi* sp. nov., and *Cryptophylliumphami* sp. nov. males. Unfortunately, due to intraspecific variation within *Cryptophylliumkhmer* sp. nov., *Cryptophylliumbollensi* sp. nov., and *Cryptophylliumphami* sp. nov. we could not identify a reliable morphological feature for differentiation males based on morphology alone.

This difficulty for differentiating a single sex alone emphasizes the importance of captive rearing of specimens to reveal the informative set of female, male, and egg morphology, and of course the importance of molecular comparison.

###### Distribution.

At present only confirmed from two Cambodian provinces, Koh Kong Province (Tatai) and Siem Reap Province (Kbai Spean and Phnom Kulen N.P., Forest Near Prean Thom). It is likely that other nearby localities may also represent this species, but due to a lack of molecular data we cannot at this time confirm them.

###### Description.

**Figure 38.**
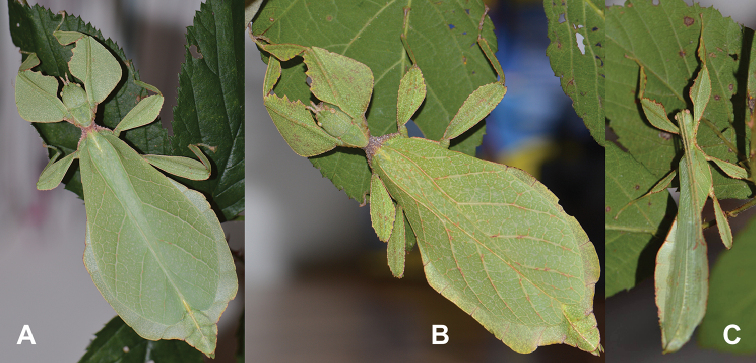
Live *Cryptophylliumkhmer* gen. et sp. nov. type material bred and photographed by Tim Bolllens (Belgium), dorsal views **A** paler form female (paratype) **B** darker form female (paratype) **C** male (paratype).

**Female. *Coloration.*** Coloration description is based upon photographs of living individuals (Fig. 38A, B) reared by Tim Bollens (Belgium). Overall coloration pale mint green with variable slight highlighting of orange or tan coloration throughout. Compound eyes are slightly more yellow with tan highlights. Antennae are tan. The prescutum and mesopleura are reddish tan with pale cream granulation throughout. Throughout the head, legs, and body there is slight speckling as granulation is slightly paler in color than the surface it is found on. In lighter individuals, the venation of the tegmina is pale yellow to pale mint green (Fig. 38A) and in darker individuals the venation is yellow with highlights of orange interspersed throughout (Fig. 38B). Darker individuals also have variable reddish patches throughout the lobes of the legs and slightly darker coloration on the abdomen.

**Figure 39.**
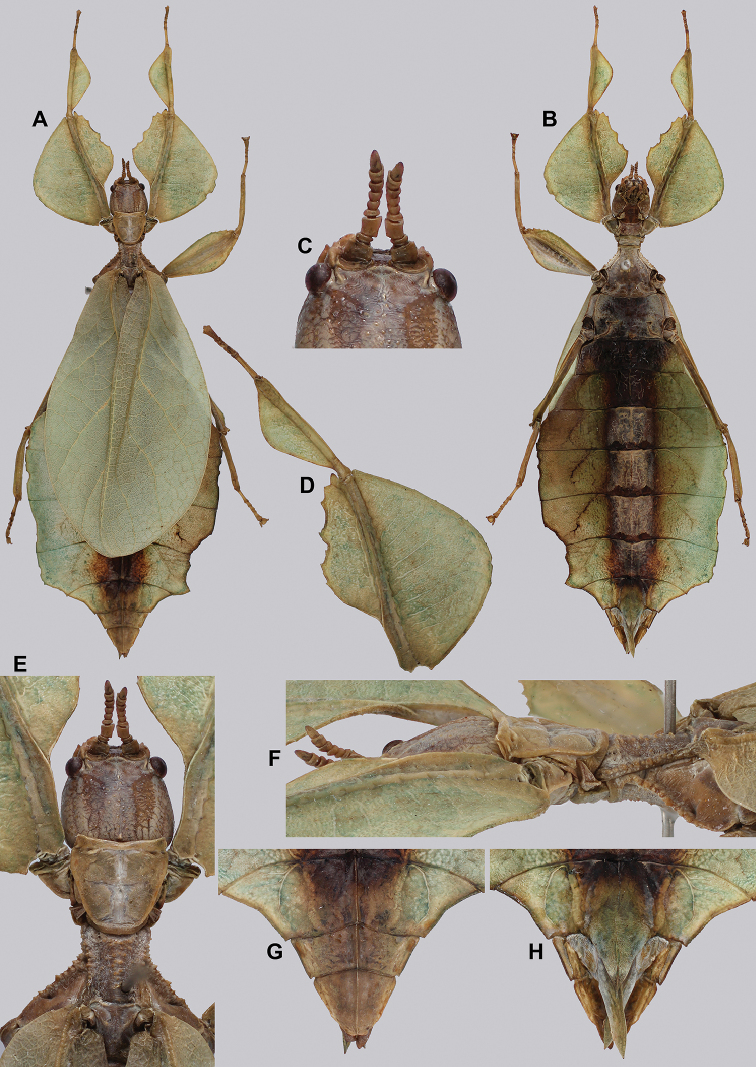
*Cryptophylliumkhmer* gen. et sp. nov. paratype female, molecular sample RBINS01 in our analysis, from Tatai, Cambodia, photographs by Jérôme Constant (RBINS) **A** habitus, dorsal **B** habitus, ventral **C** details of the antennae, dorsal **D** pro- tibial and femoral lobes, dorsal **E** details of the antennae, head, and thorax, dorsal **F** details of the antennae, head, and thorax, lateral **G** terminalia, dorsal **H** genitalia, ventral.

***Morphology.****Head.* Head capsule slightly longer than wide, vertex with granulation throughout the surface, none as prominent as the posteromedial tubercle which is not notably wide but is distinctly taller than any other nodes on the head (Fig. 39E). Frontal convexity stout, marked throughout with slight granulation and several short setae. Compound eyes slightly protruding from the head capsule, but are significantly large, taking up slightly < ⅓ of the head capsule margins (Fig. 39E). Ocelli absent. Antennal fields slightly wider than the first antennomere. *Antennae.* Antennae consisting of nine segments, with the terminal segment slightly longer than the preceding two segments’ lengths combined (Fig. 39C). Antennomeres I–VIII sparsely marked with small transparent setae, the terminal antennomere is covered densely in slightly shorter setae. *Thorax.* Pronotum slightly wider than long, with gently concave anterior margin and slightly convex lateral margins, which converge to a slightly convex posterior margin that is half the width of the anterior margin (Fig. 39E). The pronotum surface is marked with granulation throughout, a prominent pit in the center, and slight furrows anterior and lateral to the pit (Fig. 39E). The pronotum has a prominent anterior rim and weakly formed lateral and posterior rims (Fig. 39E). Prosternum and the anterior half of the mesosternum are marked with stout and numerous nodes, with the remainder of the mesosternum and the metasternum lacking prominent nodes (Fig. 39B). Prescutum about as long as wide with lateral rims with 11 or 12 lumpy tubercles ranging in size from small to medium with granulation present throughout the length giving the margins a tough textured appearance (Fig. 39E). Prescutum anterior rim not strongly protruding and marked with a granular surface (Fig. 39F). Prescutum surface with 14 or 15 distinct nodes predominantly along the sagittal plane, with those on the anterior half slightly larger than the rest (Fig. 39E). Mesopleura are narrow and parallel on the anterior ⅓, and then bend distinctly and diverge uniformly throughout their length; lateral margin with 13–16 small to medium lumpy tubercles, of which three or four are slightly larger than the rest, but most are small and variable in shape, giving the margin a rough textured appearance (Fig. 39E). Face of the mesopleura with granulation along the margin, with the remainder of the surface relatively smooth or with slight wrinkles. The surface of the mesopleura also has two distinct pits, one near the anterior ⅓ where the mesopleura bend, and one near the posterior ⅓ (Fig. 39E). *Wings.* Tegmina long, reaching onto abdominal segment VIII. The subcosta (Sc) is the first vein in the forewing and runs parallel with the wing for the first half of its length, and then bends towards the wing margin for the second half, terminating ca. ⅓ of the way through the wing length. The radius (R) spans the central portion of the tegmina with two subparallel branched veins. The first radius (R1) branches ca. ½ through the radius length and terminates ca. ⅖ of the way through the wing length. The radial sector (Rs) branches from the end of the radius and runs angled to the wing margin where it terminates near the posterior ⅓ of the wing length. There is a weak continuation of the radius following the prominent radial sector branching which continues on as a short and thin radius to media crossvein (R–M). The media (M) is simply bifurcate with both the media anterior (MA) and media posterior (MP) terminating close to the posterior ⅕ of the wing. The cubitus (Cu) runs throughout the entire wing length simply, and then near the posterior ⅕ of the wing splits into the cubitus anterior (CuA) and cubitus posterior (CuP) which both terminate at or very near the wing posterior apex. The first anal vein (1A) is simple and fuses with the cubitus early on, at around the midline between the first radial branching and the radial sector branching. Alae well-developed, reaching abdominal segment VI. *Abdomen.* Abdominal segments II through the anterior half of IV diverging, the posterior half of IV through the anterior half of VII parallel-sided (giving the abdomen a boxy appearance), the remainder of VII smoothly rounded and converging to the apex with segments VIII–X. *Genitalia.* Subgenital plate starts at the anterior margin of segment VIII, is moderately broad, and extends ½ to ¾ of the way onto segment X, ending in a fine point (Fig. 39H). Gonapophyses VIII are long and moderately broad, exceeding the apex of the abdomen with the tips slightly longer than the cerci, gonapophyses IX are thinner and shorter, hidden below gonapophyses VIII (Fig. 39H). Cerci flat, not strongly cupped, with a finely granular surface and moderately marked with a few short setae. *Legs.* Profemoral exterior lobe broad and smoothly rounded, ca. 1½ to ca. 2× wider than the interior lobe (Fig. 39D). Margin of the profemoral exterior lobe with 10–12 small weakly formed teeth throughout the length (Fig. 39D). Profemoral interior lobe obtusely angled and typically marked with five teeth arranged in a two-one-two pattern with looping gaps between them, but occasionally individuals can have doubly serrate teeth or an extra small tooth between sets (Fig. 39D). Mesofemoral exterior lobe arcs from end to end but is weighted towards the distal half with a detectable bend and marked with four or five rounded teeth distributed on the distal half only. Interior and exterior lobes of a similar width. Mesofemoral interior lobe arcs end to end smoothly with five or six small serrate teeth only on the distal half of the arc which is slightly wider than the proximal half of the arc. Metafemoral interior lobe arcs end to end and has five or six serrate teeth on the distal half of the lobe which is slightly wider than the proximal half. Metafemoral exterior lobe is thin and smooth, hugging the metafemoral shaft and lacks notable teeth but the distal ⅓ can be slightly granular. Protibial interior lobe spans the entire length of the protibiae and is ca. 2½ the width of the protibiae shaft itself. The lobe is roundly triangular and is slightly wider on the distal half. Protibiae lacking a distinct exterior lobe. Mesotibiae and metatibiae lacking exterior and interior lobes.

***Measurements of paratype females* [mm] (from Tatai, Cambodia).** Length of body (including cerci and head, excluding antennae) 83.3–90.0, length/width of head 8.4–8.7/6.6–7.1, antennae 4.1–4.6, pronotum 5.6–6.0, mesonotum 7.6–7.8, length of tegmina 52.8-53.6, length of alae 42.6 (only measured on one specimen, the others have the alae covered by the tegmina), greatest width of abdomen 31.3–36.2, profemora 19.1–21.4, mesofemora 15.1–15.4, metafemora 18.7–19.6, protibiae 12.5–12.6, mesotibiae 11.4–11.6, metatibiae 14.7–15.0.

**Male. *Coloration.*** Coloration description based on images of live males bred by Tim Bollens (Belgium). Overall coloration pale mint green throughout with highlighting of tan to orange (Fig. 39C). The areas most often with the orange highlighting are the tips of the antennae, margins of the lobes of the legs, the thorax, and the margins of the abdomen. Additionally, on more prominently colored individuals the base of the antennae and the posteromedial tubercle of the head capsule can also be colored. Compound eyes are a muddled tan to reddish.

**Figure 40.**
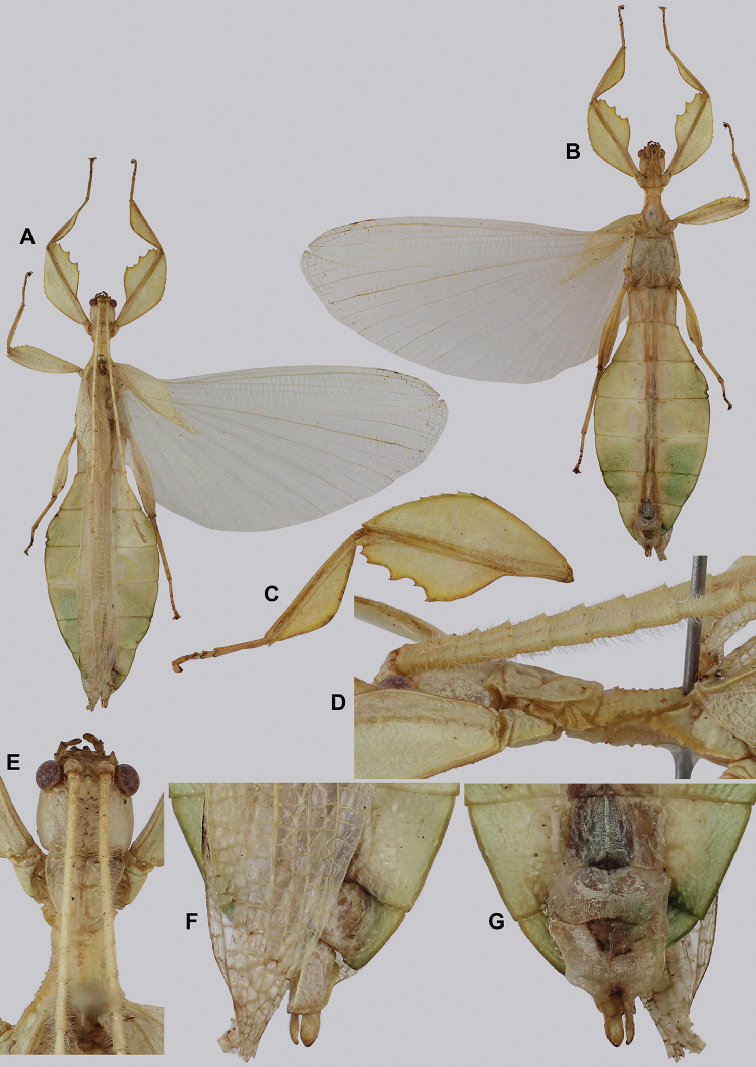
*Cryptophylliumkhmer* gen. et sp. nov. holotype male, from Tatai, Cambodia, photographs by Jérôme Constant (RBINS) **A** habitus, dorsal **B** habitus, ventral **C** pro- tibial and femoral lobes, dorsal **D** details of the antennae, head, and thorax, lateral **E** details of the antennae, head, and thorax, dorsal **F** terminalia, dorsal **G** genitalia, ventral.

***Morphology.****Head.* Head capsule about as long as wide, with a vertex that has moderate granulation throughout and a prominent but not broad posteromedial tubercle which is larger than any of the granules on the head capsule (Fig. 40E). Frontal convexity not particularly long but ending in a fine point and covered with sparse thin setae. Compound eyes large and bulbous, taking up ca. ⅖ of the head capsule lateral margins (Fig. 40E). There are three moderately developed ocelli located between and slightly posterior to the compound eyes. Antennal fields about as wide as the scapus. *Antennae.* Antennae (including the scapus and pedicellus) consists of 25 segments, all segments except the scapus and pedicellus and terminal three segments are covered in dense setae that are as long as or longer than the antennae segment is wide. The terminal three segments are covered in dense short setae and the scapus and pedicellus are nearly completely bare. *Thorax.* Pronotum with anterior margin slightly concave and lateral margins that are straight or slightly convex and converging to a straight posterior margin that is half the width of the anterior rim (Fig. 40E). Anterior margin of the pronotum has a distinct rim, lateral margins have moderate rims, and the posterior margin lacks a rim (Fig. 40E). Face of the pronotum is marked by a distinct sagittal furrow and pit in the center, a granular surface, and a slight perpendicular furrow from the central pit. Prosternum is granulose throughout with small nodes of nearly even size. Mesosternum anterior half with nodes of a similar size to the prosternum and those on the posterior half slightly less prominent. The metasternum has a slightly wrinkled surface and sparse granulation. Prescutum longer than wide, with lateral margins slightly converging to the posterior (Fig. 40E). Lateral rims with small granulation throughout giving them a rough textured appearance, only three or four are slightly larger than the rest. Prescutum surface with granulation throughout with those along the sagittal plane slightly larger than the others. Prescutum anterior rim weakly formed but marked with a surface which is granular. Mesopleura narrow, almost parallel for the anterior quarter, and then only gradually diverge for the remainder of the length (Fig. 40E). Lateral margin lacking prominent tubercles, instead marked with sharp granulation throughout with only two or three slightly larger than the rest, giving the margins a rough textured appearance. Face of the mesopleura slightly wrinkled and with two faint divots, one near the anterior margin and one half-way through the length (Fig. 40D). *Wings.* Tegmina moderate length, reaching ⅓ to ½ onto abdominal segment III. Tegmina wing venation: the subcosta (Sc) is the first vein, is simple, and terminates ca. ½ through the overall tegmina length. The radius (R) spans the entire length of the tegmina with the first radius (R1) branching < ca. ½ through the wing length and terminating just distal to the midline, followed by the branching and termination of the second radius (R2) near the distal ⅓ of the wing, and then the radial sector runs to the wing apex. The media (M) also spans the entire length of the tegmina with the first media posterior (MP1) branching off ca. ⅖ of the way through the wing length, and then the second media posterior (MP2) branches near the midline, and the media anterior (MA) runs to the wing apex. The cubitus (Cu) runs along the edge of the wing as the two media posterior veins fuse with it and as the cubitus reaches the apex it fades. The first anal (1A) vein terminates upon reaching the cubitus slightly < ⅓ of the way through the wing length. Alae well-developed in an oval fan configuration, long, reaching onto abdominal segments IX or X. Alae wing venation: the costa (C) is present along the entire foremargin giving stability to the wing. The subcosta (Sc) is long, spanning slightly > ⅔ of the wing length and is mostly fused with the radius in the beginning but terminates when it meets the costa. The radius (R) spans the entire wing and branches ca. ⅓ of the way through into the first radius (R1) and radial sector (Rs) which run gently diverging for most of their length and then converge at the apex of the wing where they terminate near each other but not touching. The media (M) branches early, ca. ⅙ of the way through the wing into the media anterior (MA) and the media posterior (MP) which run parallel with each other throughout the wing until the distal ⅙ of the wing where the media posterior fuses with the media anterior which then run fused together to the wing apex where they terminate near the radial sector. The cubitus (Cu) runs unbranched and terminates at the wing apex. Of the anterior anal veins, the first anterior anal (1AA) fuses with the cubitus near the point where the media branches into the media anterior and media posterior and then the first anterior anal branches from the cubitus ⅔ of the way through the wing length where it uniformly diverges from the cubitus until it terminates at the wing margin. The anterior anal veins two–seven (2AA–7AA) have a common origin and run unbranched in a folding fan pattern of relatively uniform spacing to the wing margin. The posterior anal veins (1PA–6PA) share a common origin separate from the anterior anal veins and run unbranched to the wing margin with slightly thinner spacing than the anterior anal veins. *Abdomen.* Margins of abdominal segment II either slightly converging or parallel-sided. Abdominal segments III through the anterior ⅔ of IV diverging. Segment V with parallel margins and VI–X converging slowly at first then more prominently for the terminal three segments, giving the abdomen a spade-shaped appearance. *Genitalia.* Poculum broad, posteriorly rounded and with a shallow notch medioapically; slightly passes the anterior margin of segment X (Fig. 40G). Cerci long and slender, extending from under the anal abdominal segment, slightly cupped with a granular surface and numerous short setae throughout (Fig. 40F). Vomer broad and stout with sides evenly converging and terminating in an upward hooking apical spine with a smaller hook next to the base of the primary spine (Fig. 5D). *Legs.* Profemoral exterior lobe about the same width as the interior lobe or slightly wider, smoothly arcing from end to end and marked with a granular margin and five or six small serrate teeth on the distal half only (Fig. 40C). Profemoral interior lobe roundly triangular and marked with five teeth arranged in a two-one-two pattern with prominent looping gaps between the sets and the middle tooth larger than the others (Fig. 40C). Mesofemoral exterior lobe arcs end to end, but is slightly more bent than the interior lobe and is broader on the distal half which can either be lacking dentation or have three or four dulled teeth, and the proximal half that is rather thin and lacking teeth. Mesofemoral interior lobe of a similar width to the exterior lobe, is broader on the distal end and is marked with five or six serrate teeth mostly situated on the distal ⅓ to ½ of the lobe. Metafemoral exterior lobe lacks dentation, and has a straight margin along the metafemoral shaft. Metafemoral interior lobe smoothly arcs end to end with eight or nine serrate teeth on the slightly wider distal half. Protibiae lacking exterior lobe, interior lobe reaching end to end in a smoothly rounded triangle with the widest portion ca. 3–3½× as wide as the protibial shaft and situated just distal to the midline. Meso- and metatibiae simple, lacking lobes completely.

***Measurements of holotype male* [mm].** Length of body (including cerci and head, excluding antennae) 61.9, length/width of head 4.5/3.9, antennae 37.3^1^

, pronotum 3.6, mesonotum 4.3, length of tegmina 19.0, length of alae 49.3, greatest width of abdomen 17.3, profemora 13.5, mesofemora 11.3, metafemora 13.4, protibiae 9.7, mesotibiae 7.8, metatibiae 10.1.

***Measurements of paratype males* [mm].** Length of body (including cerci and head, excluding antennae) 63.8–70.2, length/width of head 5.0–5.5/4.1–4.3, antennae 38.8–39.5, pronotum 3.6–4.1, mesonotum 5.0–6.2, length of tegmina 20.0–20.4, length of alae 50.0–52.1, greatest width of abdomen 17.1–17.9, profemora 15.7^2^

, mesofemora 12.1, metafemora 13.7–14.1, protibiae 9.8**, mesotibiae 8.7–8.9, metatibiae 11.2–11.4.

**Figure 41.**
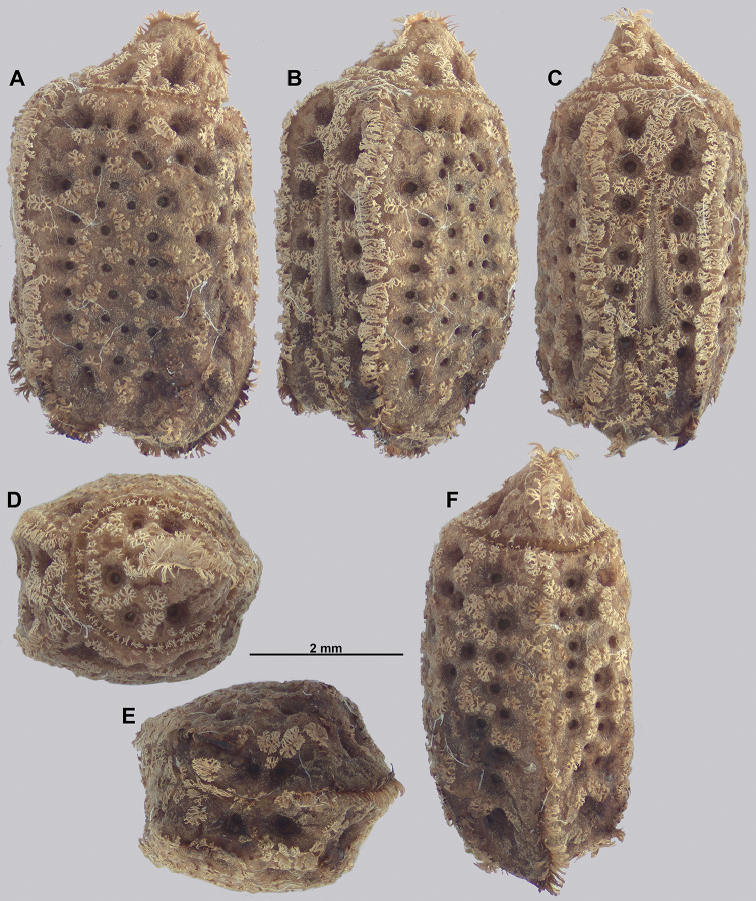
*Cryptophylliumkhmer* gen. et sp. nov. egg, RBINS collection, photographs by Jérôme Constant (RBINS) **A** lateral view **B** dorso-lateral view **C** dorsal view **D** opercular (anterior) view **E** posterior view **F** ventral view.

**Eggs.** (Fig. 41). The lateral surfaces are flat with a length ca. 1½× the width with parallel margins, giving the capsule a rectangular appearance. All surfaces have numerous small to medium sized pits throughout, the lateral surface has around 35 pits (mostly on the smaller end of the spectrum) arranged in no detectable order, some more closely spaced than others. In addition, between the pits the surfaces are covered with short moss-like pinnae with the pinnae along the margins slightly longer than the pinnae on the other surfaces. The dorsal surface is marked with six or seven slightly irregular medium sized pits on each half running the length of the capsule with short moss-like pinnae around the micropylar plate and between the pits. The micropylar plate is not overly long, occupying ca. ½ of the dorsal surface length but not perfectly centered, with ca. ⅓ of the unoccupied space below and ⅔ above the micropylar plate. The micropylar cup is the widest portion of the micropylar plate and is located ca. ⅓ of the dorsal surface length from the posterior. The micropylar plate is approximately teardrop-shaped with the anterior portion longer and thinner than the posterior after the micropylar cup. Operculum slightly ovular, with the outer margin encircled with short moss-like pinnae surrounding the operculum and four or five medium pits surrounding the dorsal and lateral margins. The operculum is roundly raised with a height slightly > ½ operculum width. This rounded raised cap is marked with a sagittal raised row of pinnae similar in length to those along the capsule margins. The rounded raised cap is not perfectly centered and instead the rounded projection is shifted slightly towards the ventral surface. The overall egg color is tan to light brown, with the moss-like pinnae sometimes slightly lighter in color.

**Measurements including the extended pinnae [mm].** Length (including operculum): 5.6; maximum width of capsule when viewed from lateral aspect 3.2; length of micropylar plate 3.0.

###### Etymology.

Noun. The species epithet is the Hindi word *khmer*, meaning Cambodia, referring to the country of origin for this species.

##### Cryptophyllium limogesi

Taxon classificationAnimalia

gen. et sp. nov.

3634D0DD-9872-5A75-9E2C-550D8B673343

http://zoobank.org/411639C9-07AD-4BE6-AFAD-F8D5D4EA1152

**Figure 42.**
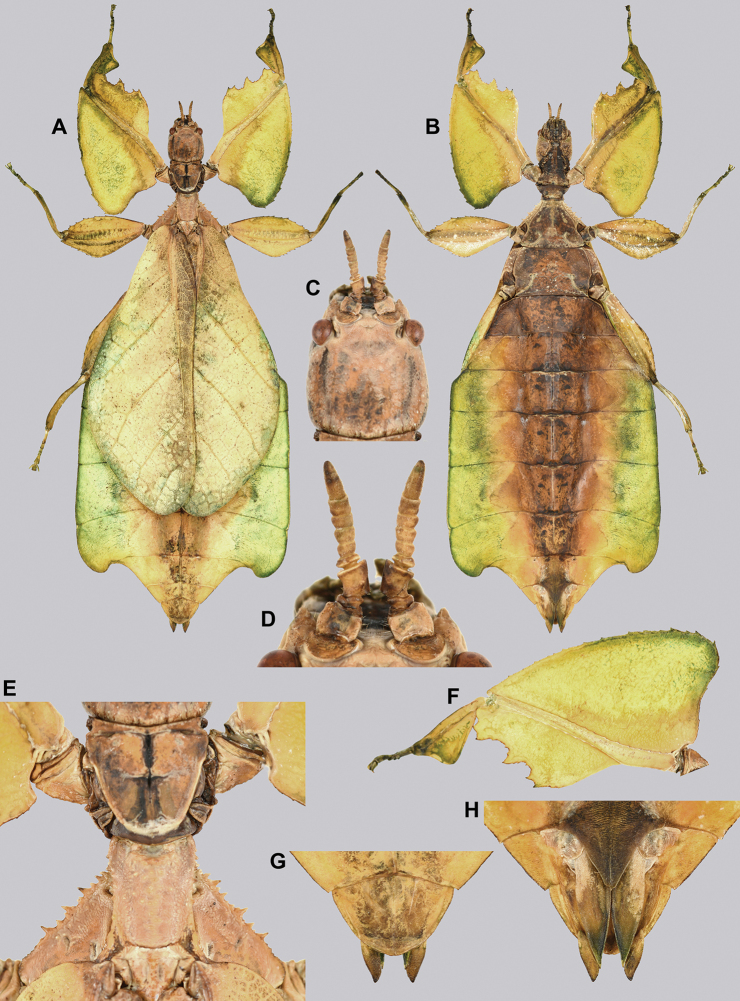
Holotype female *Cryptophylliumlimogesi*, photographs by René Limoges (IMQC) **A** habitus, dorsal **B** habitus, ventral **C** head and antennae dorsal **D** antennae detail dorsal **E** thorax dorsal **F** front leg, dorsal **G** terminalia, dorsal **H** genitalia, ventral.

**Figure 43.**
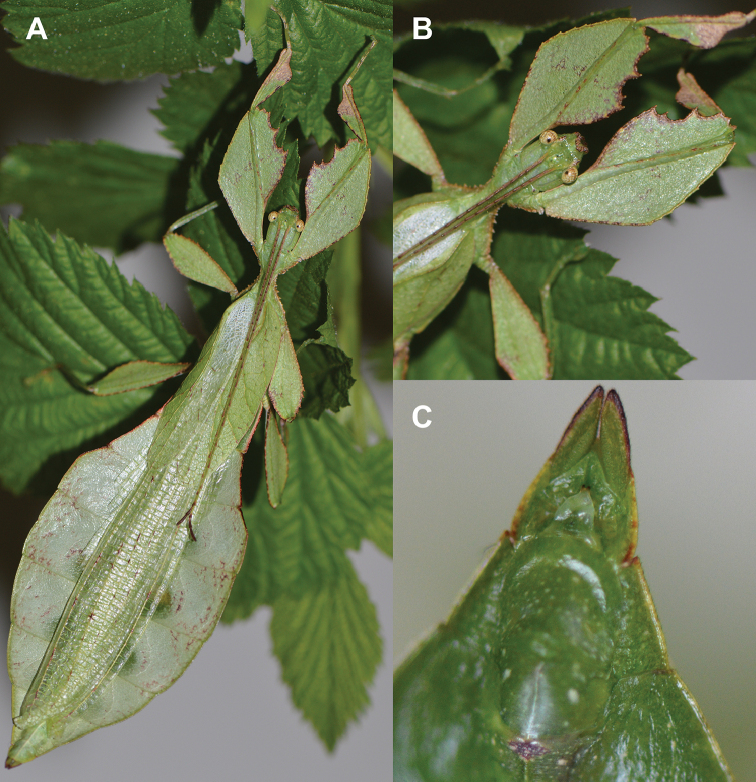
Paratype male *Cryptophylliumlimogesi* gen. et sp. nov. from Ta Dung N.P. reared to adulthood and photographed by Tim Bollens (Belgium) **A** habitus, dorsal **B** detail of the front legs and head **C** genitalia details, ventral.

**Figure 44.**
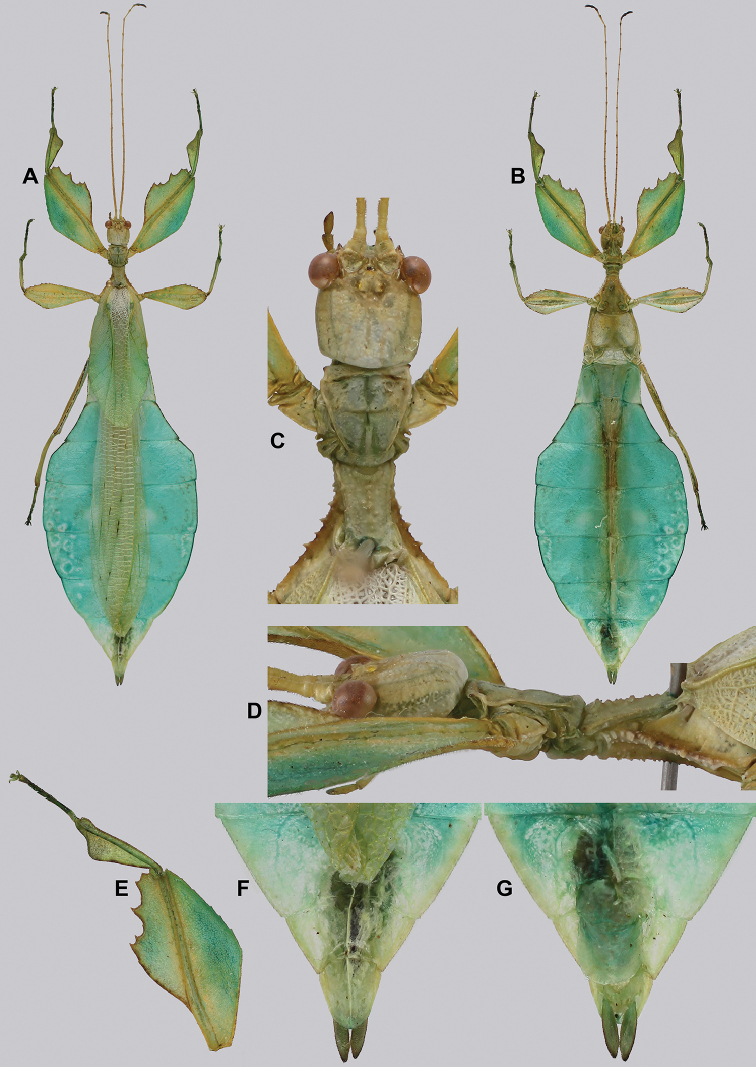
Paratype male *Cryptophylliumlimogesi* gen. et sp. nov. same individual as in Figure 43 photographs by Jérôme Constant (RBINS) **A** habitus, dorsal **B** habitus, ventral **C** details of the base of antennae, head, and thorax, dorsal **D** details of the base of antennae, head, and thorax, lateral **E** front leg details, dorsal **F** genitalia details, dorsal **G** genitalia details, ventral.

**Figure 45.**
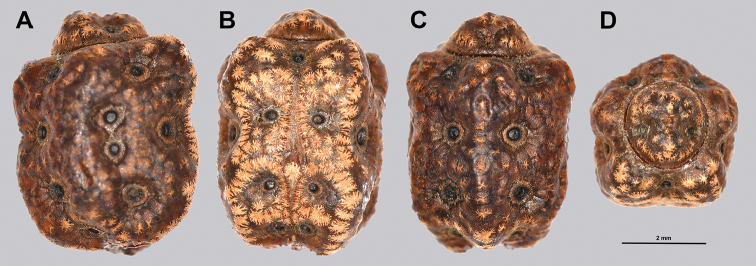
Paratype *Cryptophylliumlimogesi* gen. et sp. nov. egg, laid by paratype females from Dak Lak Province, Vietnam. Photographs by René Limoges (IMQC) **A** lateral **B** dorsal **C** ventral **D** opercular (anterior) view.

Figures 5J , 8Q , 8R , 42 , 43 , 44 , 45

###### Material examined.

***Holotype*** ♀: “VIETNAM, Lam Dong Province, Bao Lam, Dambri, V.2018”. Deposited within the Montreal Insectarium (IMQC).

***Paratypes***: (13 ♀♀, 2 ♂, 7 eggs) • 1 ♂: “Coll. I.R.Sc.N.B., VIETNAM, Dak Nong prov., Ta Dung N.P., 11°52’22”N 107°58’40”E, 5-8.viii.2019, GTI Project, Leg. J. Constant and J. Bresseel, I.G.: 34.048 (Coll. I.R.Sc.N.B.)”, vomer dissected (RBINS) (SB0531 molecular sample within our analysis) • 1 ♂: “Vietnam: Dak Lak Province, Local collector, September 2020” (IMQC) • 5 ♀♀; “Vietnam: Dak Lak Province, Local collector, September 2020” (Coll RC 20-127, 20-128, 20-129) • 5 ♀♀; “Vietnam: Dak Lak Province, Local collector, September 2020” (Coll SLT) • 3 ♀♀; “Vietnam: Dak Lak Province, Local collector, September 2020” (IMQC) • 1 egg: “Vietnam: Dak Lak Province, Local collector, September 2020” (Coll SLT) • 1 egg: “Vietnam: Dak Lak Province, Local collector, September 2020” (Coll FH) • 3 egg: “Vietnam: Dak Lak Province, Local collector, September 2020” (IMQC) • 2 egg: “Vietnam: Dak Lak Province, Local collector, September 2020” (Coll RC).

###### Remarks.

This large and morphologically unique species has been located in several provinces of southern Vietnam over the last few years and molecularly we found it to be sister species to *Cryptophylliumicarus* sp. nov. (Fig. 4) a species also only at the present known from southern Vietnam (Fig. 2). Interestingly, these molecular sister species are morphologically drastically different, perhaps due to their geographic sympatry. During a GTI joint expeditions two small nymphs were collected in Ta Dung N.P. on the forest edge adjacent to a coffee plantation. Tim Bollens (Belgium) reared one nymph to adulthood, thus revealing the male morphology (Fig. 42).

###### Differentiation.

Female *Cryptophylliumlimogesi* sp. nov. are most morphologically similar to *Cryptophylliumcelebicum* comb. nov. and *Cryptophylliumtibetense* comb. nov. based on the wide profemoral exterior lobe with an acute angle and broad boxy abdomen. From *Cryptophylliumcelebicum* comb. nov., the easiest observed difference is the structure of the thorax, with the mesopleura of *Cryptophylliumcelebicum* comb. nov. notably narrower on the anterior half vs. the mesopleura of *Cryptophylliumlimogesi* sp. nov. reaching nearly to the anterior margin of the prescutum with straight margins (Fig. 42E). From *Cryptophylliumtibetense* comb. nov. the female subgenital plates easily differentiate these species as *Cryptophylliumtibetense* comb. nov. has a long subgenital plate with a point which exceeds the apex of the terminal abdominal segment (Fig. 63B) vs. *Cryptophylliumlimogesi* sp. nov. where the subgenital plate is short, reaching no more than ½ through the terminal abdominal segment (Fig. 42H).

Male *Cryptophylliumlimogesi* sp. nov. are similar morphologically to *Cryptophylliumoyae* comb. nov. due to the broad spade-shaped abdomen and the profemoral lobe shapes. These species can be differentiated by the length of their tegmina (only reaching onto abdominal segment III in *Cryptophylliumlimogesi* sp. nov. but reaching halfway onto segment IV in *Cryptophylliumoyae* comb. nov.); and they can be differentiated by the shape of the mesopleura as they are broad and nearly straight margined in *Cryptophylliumoyae* comb. nov. but slightly narrower on the anterior in *Cryptophylliumlimogesi* sp. nov. males (Fig. 44C).

###### Distribution.

Southern Vietnam: presently known from three provinces: the type locality of Lam Dong Province, Bao Lam, Dambri; the male paratype record from Dak Nong Province, Ta Dung N.P.; an observational record from Dak Lak Province, Chu Yang Sin N.P.; and paratype records> from Dak Lak Province.

###### Description.

**Female. *Coloration*.** Coloration description is based on photos of the holotype female shortly after being preserved. Nearly the entire body was of a uniform lime-green without differing colored markings (all legs and even wing venation a similar color to the rest of the body). Only the compound eyes were slightly yellow and not the same shade of green as the rest of the body.

***Morphology*.***Head*. Head capsule longer than wide, vertex with a moderately granular surface, and the posteromedial tubercle which is three or four times larger than the most prominent granules on the capsule. Frontal convexity broad and stout, notably shorter than the length of the first antennomere, and with several long, thin, clear setae across the surface. Compound eyes are not large, only slightly protruding from the head capsule and with a width of ca. ¼ the head capsule length (Fig. 42C). Ocelli absent. Antennal fields wider than the first antennomere but not protruding back farther than the frontal suture. *Antennae*. Antennae consist of nine segments, with the terminal segment approximately the same length as the previously three segments combined (Fig. 42D). The eighth antennal segment has a distinct furrow around the middle which makes the segment appear to be two separate segments (giving the antennae a ten segmented appearance), but this furrow appears to only be superficial (Fig. 42D). Antennomeres I-III sparsely marked with thin transparent setae, similar to those found on the frontal convexity, but slightly shorter in length. Antennomere IV is short, disk-like, and wider than the following segments, and interestingly has a base which is narrow and somewhat longer than other congeneric antennal segments IV, giving it a raised appearance (Fig. 42D). The terminal antennomere and the distal half of segment VIII (distal to the midline furrow) are covered in dense, stout, setae. *Thorax*. Pronotum with anterior margin slightly concave and lateral margins that are relatively straight, converging to a narrow, straight posterior margin that is ca. ½ the width of the anterior rim (Fig. 42E). The pronotum surface is smooth, with only a prominent pit in the center, and slight furrows anterior and lateral to the pit, no prominent wrinkles or granulation. The pronotum has a prominent anterior rim and moderate lateral rims, the posterior is lacking a rim. Prosternum with notable nodes throughout the surface, relatively evenly spaced. Mesosternum with prominent nodes on the anterior margin, followed by moderate nodes on the anterior ⅓ of the surface, with the remainder with dispersed, weak granulation which continues onto the metasternum. Prescutum notably longer than wide, with lateral margins running parallel to the posterior margin giving it a distinctly rectangular appearance. Lateral rims with five or six medium sized tubercles situated on the anterior ⅔, with only small granulation on the remainder. Prescutum anterior rim prominent but not strongly protruding, with the surface granular and lacking a prominent sagittal tubercle. Prescutum surface with granulation throughout with those along the sagittal plane slightly larger (Fig. 42E). Mesopleura start ca. ⅓ down the prescutum and evenly diverge with straight lateral margins. Lateral margin with six or seven major and distinctly pointed tubercles and six or seven smaller tubercles intermixed amongst them. This mix of tubercles is only prominent along the anterior ⅔ of the length with the remaining ⅓ lacking notable tubercles and instead marked with consistent granulation (Fig. 42E). Face of the mesopleura with slight wrinkles throughout most of the surface and a few irregular nodes along the lateral margin, as well as two faint divots, one on the anterior margin and one closer to the center. *Wings*. Tegmina reaching slightly past the anterior margin of abdominal segment VII. Tegmina venation is rather typical for the *Cryptophyllium* gen. nov. The subcosta (Sc) is the first vein in the forewing and runs parallel with the wing for the first half of its length, and then bends towards the wing margin for the second half. The radius (R) spans the central portion of the tegmina with two subparallel branched veins. The first radius (R1) branches ca. ½ through the radius length and terminates ca. ⅓ of the way through the wing length. The radial sector (Rs) branches from the end of the radius and runs angled to the wing margin where it terminates near the wing midline length. There is a weak continuation of the radius following the prominent radial sector branching which continues on as a short and thin radius to media crossvein (R–M). The media (M) is simply bifurcate with both the media anterior (MA) and media posterior (MP) terminating close to the posterior ¼ of the wing. The cubitus (Cu) runs throughout the entire wing length simply, and then near the posterior ⅕ of the wing becomes bifurcate into the cubitus anterior (CuA) and cubitus posterior (CuP) which both terminate at or very near the wing posterior apex. The first anal vein (1A) is simple and fuses with the cubitus early on, at around the midline between the first radial branching and the radial sector branching. Alae short, only 21.8 mm long. *Abdomen*. Abdominal segments II through the anterior half of IV diverging, posterior half of IV through the anterior ⅓ of VII parallel. Abdominal segment VII with a distinct looping lobe which meets abdominal segment VIII which is notably narrower. Segments VIII–X uniformly converge to a broad rounded apex. *Genitalia*. Projecting portion of the subgenital plate stout, beginning at the anterior margin of abdominal segment IX and projecting with nearly straight sides to just under the anterior margin of the terminal abdominal segment (Fig. 42H). Gonapophyses VIII are long and broad with a dagger-like shape (parallel-sided at first and then after ca. ½ of the length uniformly converging to the point) with the points just projecting from under the terminal abdominal segment (Fig. 42H). Gonapophyses IX are smaller and not visible from under the large gonapophyses VIII. Cerci strongly pointed and relatively flat (not strongly cupped) with weakly crenate margins, and the dorsal surface heavily granular and marked by thin transparent setae throughout (Fig. 42G, H). *Legs*. Profemoral exterior lobes broad, at its broadest ca. 2× wider than the interior lobe, and distinctly serrate throughout the entire length (with 13–17 small, pointed teeth). The proximal edge is slightly concave and the distal edge is smoothly convex, therefore giving the lobe a distinct recurved appearance and an acute exterior angle (Fig. 42F). Interior profemoral lobe ca. 2½× the width of the profemoral shaft at its widest and with doubly serrate, large, triangular teeth. The largest teeth are grouped into a two-one-two pattern with large looping gaps between these groupings, with these large gaps also finely serrate, not smooth (Fig. 42F). Interior mesofemoral lobe arcs evenly weighted from end to end, and at its widest is approximately the same width as the mesofemoral shaft itself. The interior mesofemoral lobe is finely serrate for ca. ¾ of its distal length with seven or eight teeth. Mesofemoral exterior lobe is also approximately at its widest as wide as the mesofemoral shaft, but the exterior lobe is distinctly bent in the center with straight margins, not smoothly arcing from end to end. On the distal half of the lobe only there are six or seven small serrate teeth. Metafemoral interior lobe arcs end to end and has five or six dull teeth pointing distally. Metafemoral exterior lobe is thin and smooth, hugging the metafemoral shaft and lacks dentation. Protibiae with a thin but notable exterior lobe on the distal ⅕ only. Protibial interior lobe spans the entire length as a broad scalene triangle with the broad end on the distal half of the protibiae. Mesotibiae simple, completely lacking lobes. Metatibiae lacking interior lobes, exterior is marked by a very slender lobe which only occupies the distal ¼ of the shaft.

***Measurements of holotype* female [mm].** Length of body (including cerci and head, excluding antennae) 99.0, length/width of head 8.9/6.8, antennae 5.1, pronotum 5.9, mesonotum 6.5, length of tegmina 55.7, length of alae 21.8, greatest width of abdomen 41.5, profemora 22.8, mesofemora 16.0, metafemora 19.1, protibiae 11.2, mesotibiae 11.8, metatibiae 15.0.

**Male. *Coloration*.** Coloration description based on the captive reared paratype male when it was alive (Fig. 43). Overall coloration mint-green throughout with highlights of tan coloration on the distal half of the protibiae, along all femoral lobe margins, the frontal convexity, the margins of the thorax, the base of the antennae and the distal tips of each longer antennomere, and intermittently along the tegmina and alae sclerotized veins. Abdominal segment V has a slightly darker patch and a transparent eye spot on each side of the midline. Compound eyes are pale yellow with slight orange marbling throughout.

***Morphology*.***Head.* Head capsule approximately as long as wide, with a vertex that is only slightly granular with no discernable pattern (Fig. 44C). The posteromedial tubercle is not broad and is only weakly raised from the head capsule. Compound eyes are large and bulbous, taking up ca. ⅖ of the head capsule lateral margins (Fig. 44C). There are three well-developed ocelli between and slightly posterior to the compound eyes (Fig. 44C). Antennal fields are about as wide as the scapus. *Antennae.* Antennae (including the scapus and pedicellus) consist of 29 segments. The scapus and pedicellus are nearly completely bare, lacking long setae. The following segments except the terminal three are covered in dense setae that are as long as or longer than the antennae segment is wide, and the terminal three segments are covered in dense short setae. *Thorax.* Pronotum with anterior margin slightly concave and lateral margins that are straight and converging to a slightly curved posterior margin that is ca. ½ the width of the anterior rim (Fig. 44C). Anterior and lateral margins have moderate rims and the posterior margin lacks a rim (Fig. 44C). Face of the pronotum is marked by a distinct pit in the center with a furrow anterior to the pit along the sagittal plane, weakly formed furrows lateral to the central pit, and a smooth surface with only slight granulation in no detectable pattern (Fig. 44C). The prosternum is slightly granulose throughout and the anterior ⅓ of the mesosternum surface is marked with more prominent nodes, with the remainder of the surface with fewer and smaller nodes (Fig. 44B). Prescutum longer than wide, with lateral margins slightly converging to the posterior (Fig. 44C). Lateral rims with eight or nine small tubercles (Fig. 44C). Prescutum surface slightly raised along the sagittal plane with six or seven nodes of varying size, with the remainder of the surface with only slight granulation throughout. Prescutum anterior margin weakly formed and with a granular surface, lacking a prominent central tubercle. Mesopleura narrow on the anterior quarter of the length but then gently diverging with nearly straight margins to the posterior (Fig. 44C). Mesopleura lateral margin with three large conical tubercles, three or five small tubercles, and four or five nodes throughout the length (Fig. 44C). Face of the mesopleura slightly wrinkled, with slight granulation throughout, and with two faint divots, one on the anterior margin and one near the midline. *Wings.* Tegmina moderate length, extending halfway through abdominal segment III. Tegmina wing venation: the subcosta (Sc) is the first vein, is simple, and terminates the earliest slightly < ½ through the overall tegmina length. The radius (R) spans the entire length of the tegmina with the first radius (R1) branching ca. ⅓ of the way through the wing length and terminating near the midline, followed by the branching and termination of the second radius (R2) slightly distal to the midline, and then the radial sector runs to the wing apex. The media (M) also spans the entire length of the tegmina with the first media posterior (MP1) branching off ca. ⅓ of the way through the wing length, then the second media posterior (MP2) branching near the midline, and the media anterior (MA) runs to the wing apex. The cubitus (Cu) runs along the edge of the wing as the two media posterior veins fuse with it and as the cubitus reaches the apex it fades. The first anal (1A) vein terminates upon reaching the cubitus ca. ⅓ of the way through the wing length. Alae well-developed in an oval fan configuration, long, reaching the middle of abdominal segment VIII. *Abdomen.* Abdominal segment II gently diverging, III through the anterior half of segment IV diverging to the widest portion of the abdomen. The posterior of IV–V parallel-sided giving the abdomen a broad spade-shaped appearance. Segments VI–X uniformly converging with slightly undulating margins (Fig. 44B). *Genitalia.* Poculum broad, and ends in a broadly rounded apex that slightly passes the anterior margin of segment X (Fig. 44G). Cerci long and slender, with > ½ their length extending from underneath the terminal abdominal segment (Fig. 44F), relatively flat, covered in a heavily granulose surface and with equally granular margins, and surface with numerous short setae (Fig. 44G). Vomer stout with slightly rounded sides converging to an apex with two side by side thick apical hooks which are the same size and hook upwards into the paraproct (Fig. 43C). *Legs.* Profemoral exterior lobe broader than the interior lobe, arcing end to end with a distinct rounded bend in the center, with the proximal half margin with a distinctly granular surface, and the distal half with six small serrate teeth (Fig. 44E). Profemoral interior lobe roundly triangular, at least 3× wider than the profemoral shaft, and marked with five large, serrate teeth arranged in a two-one-two pattern with looping gaps between them (Fig. 44E). Mesofemoral exterior lobe arcs end to end, is slightly wider than the mesofemoral shaft, but with the widest portion on the distal ⅓ which is marked with granulation or with one or two weakly formed teeth. Mesofemoral interior lobe is slightly thinner than the exterior lobe and is slightly broader on the distal end which is marked with six serrate teeth. Metafemoral exterior lobe lacks dentation, and has a straight margin hugging the metafemoral shaft. Metafemoral interior lobe smoothly arcs end to end, with the distal half wider than the proximal half, and the distal half is marked with seven or eight serrate teeth on the distal half. Protibiae with a small but notable exterior lobe on the anterior ⅕ which is no wider than the width of the protibial shaft (Fig. 44E). Protibial interior lobe reaching end to end in a smoothly rounded triangle with the widest portion on the distal third ca. 3× as wide as the protibial shaft (Fig. 44E). Meso- and metatibiae simple, lacking lobes.

***Measurements of reared paratype male* [mm].** Length of body (including cerci and head, excluding antennae) 79.7, length/width of head 4.9/3.9, antennae 41.8, pronotum 3.7, mesonotum 5.7, length of tegmina 24.4, length of alae 57.5, greatest width of abdomen 26.5, profemora 16.4, mesofemora 11.4, metafemora 13.7, protibiae 9.1, mesotibiae 7.7, metatibiae 10.9.

**Eggs.** (Fig. 45). When viewed from the anterior the egg capsule cross section is rounded pentagonal, therefore the lateral surfaces are raised into dorsolateral and ventrolateral surfaces. All surfaces and margins slightly undulate giving the egg an overall lumpy appearance. The ventrolateral surface is marked by four large, evenly spaced pits from the anterior to the posterior in a singular line. The dorsolateral surface has five large pits, arranged with one on the anterior, one on the posterior, and three in the middle spaced out in a two-one pattern. Both lateral surfaces are primarily bare but do have sparse and small moss-like pinnae between the pits. The dorsal surface is marked with seven medium sized pits total (one on the anterior end along the sagittal plane at the apex of the micropylar plate, followed by three on each slide of the plate with broad spacing between them ending with the posterior most near the base of the capsule). The dorsal surface is also marked throughout with short moss-like pinnae around the micropylar plate, with the area immediately around each pit bare. The micropylar plate is long, ca. 5/7 of the overall dorsal surface length with the micropylar cup situated on the posterior ⅓ of the length. The micropylar plate is thin with the widest portion the area around the micropylar cup. The operculum is slightly ovular with a surface that is roundly raised and a height slightly < ½ the operculum width. The operculum is marked intermittently with moss-like pinnae similar in shape but slightly smaller than those found on the rest of the capsule, as well as marked with two medium sized pits (one on each side of the sagittal plane). The overall egg color is dark brown, with the moss-like pinnae light brown in color so they stand out clearly on the surface.

**Measurements including the extended pinnae [mm].** Length (including operculum): 5.2; maximum width of capsule when viewed from lateral aspect 3.8; length of micropylar plate 3.0.

###### Etymology.

Patronym. Named after René Limoges (Canada) from the Montreal Insectarium to thank him for his many years of assisting Team Phyllies with countless publication worthy photographs.

##### Cryptophyllium liyananae

Taxon classificationAnimalia

gen. et sp. nov.

DC10D792-8340-5E2C-8DC0-92214AC2E7B9

http://zoobank.org/87CFC18E-3479-47CD-B052-37AEB4A07BDB

Figures 46 , 47

###### Material examined.

***Holotype*** ♀: “CHINA: Guangxi Prov., Jinxiu County, Liuzhou City, Dayaoshan Mountain, 875-1,500 m., 18-19.IX.2019. (Coll RC 20-002)”. Deposited in the Montreal Insectarium (IMQC).

***Paratypes***: (6 ♀♀, 4 eggs) • 1 ♀: “CHINA: Guangxi Prov., Jinxiu County, Liuzhou City, Dayaoshan Mountain, 875-1,500 m., 19.IX.2019. Coll RC 19-182” (Coll RC) • 1 ♀: “CHINA: Guangxi Prov., Jinxiu County, Liuzhou City, Dayaoshan Mountain, 875-1,500 m., 18-19.IX.2019. Coll RC 20-003” (Coll RC) • 3 ♀♀: Same data as the holotype (Coll SLT) • 1 ♀: Same data as the holotype (Coll FH)• 4 eggs: Removed from the abdomen of paratype female 19-182. “CHINA: Guangxi Prov., Jinxiu County, Liuzhou City, Dayaoshan Mountain, 875-1,500 m., 19.IX.2019”; Coll RC 20-069–20-072: 20-069, 20-072 (Coll RC); 20-070, 20-071 (IMQC).

**Figure 46.**
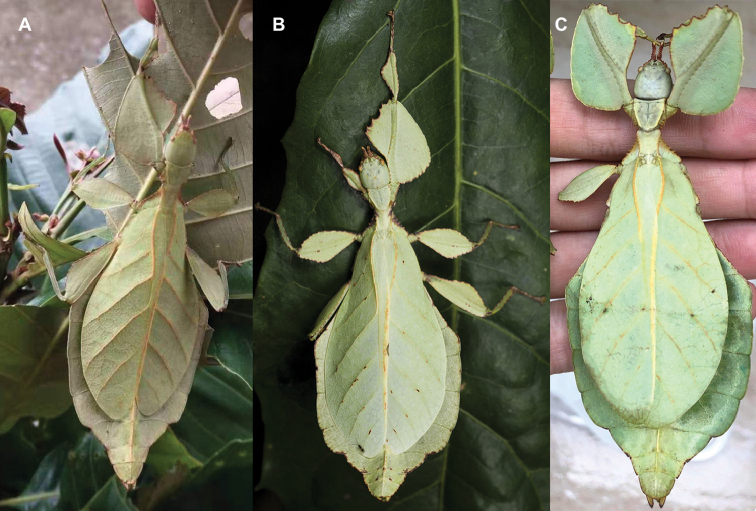
Female *Cryptophylliumliyananae* gen. et sp. nov. **A** live holotype female, photograph by Chengzhi Bian (China) **B** live female observed and photographed by Dr. Lu Qiu in Guangxi, Huaping Nature Reserve, 900–1000 m, August 2020 **C** paratype female Coll RC 19-182 shortly after being collected before the color faded, photograph by Chengzhi Bian.

***Photographic record***: (1 ♀) In addition to the type material examined, images of a live female observed by Dr. Lu Qiu in Guangxi, Huaping Nature Reserve, 900–1000m, in August 2020 (Fig. 46B) were shared with us and compared to type material.

###### Remarks.

**Figure 47.**
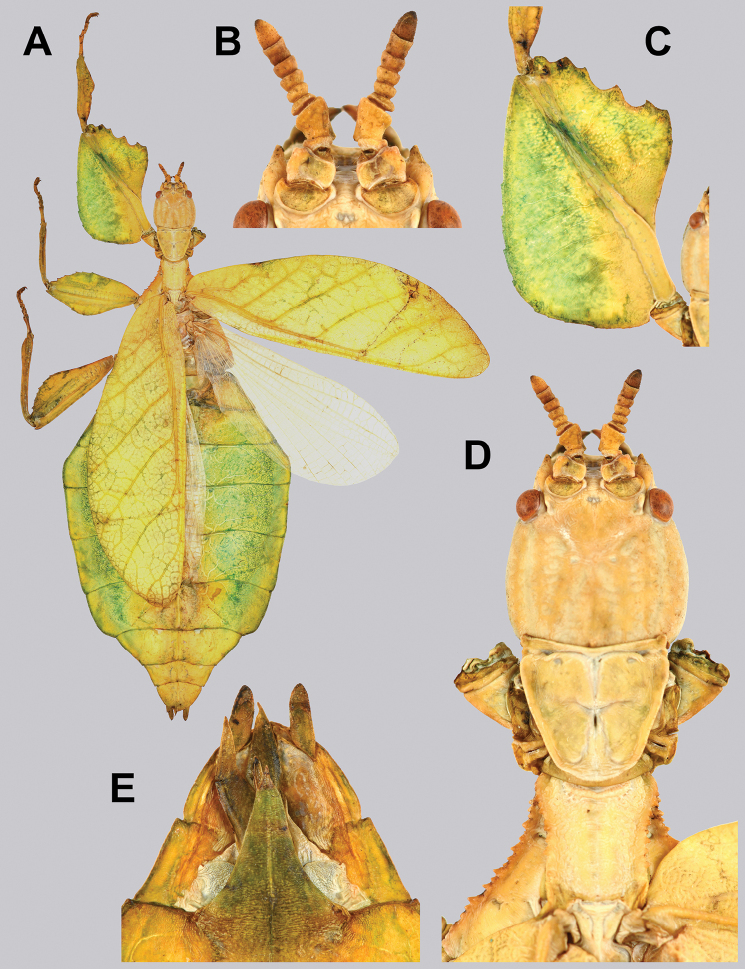
Holotype *Cryptophylliumliyananae* gen. et sp. nov., photographs by René Limoges (IMQC) **A** habitus, dorsal **B** details of the antennae, dorsal **C** profemoral lobes, dorsal **D** details of the antennae, head, and thorax, dorsal **E** genitalia, ventral.

This species appears to be the same which was illustrated by Liu (1993) and called Phyllium (Phyllium) celebicum within the work. Not only do the illustrations clearly show similar morphology, but one of the specimens is also from the locality of Dayao Mountain, Jinxiu, Guangxi. The illustrated female (figures 10–12; Liu 1993) clearly shows gently rounded lobes on abdominal segment VII, the same tegmina length, a small but notable metatibial exterior lobe on the distal end, the same shape/length features of the genitalia (Fig. 47E), mesothorax shape (Fig. 47D), similar rounded profemoral exterior lobes (Fig. 47C), and the VIII antennal segment which is notably longer than the other preceding individual segments (Fig. 47B).

###### Differentiation.

Females are morphologically similar to *Cryptophylliumdrunganum* comb. nov. due to the tibial and femoral shapes (including small exterior pro- and metatibial anteriorly situated lobes), alae long (reaching abdominal segment VI), a seventh abdominal segment which is slightly lobed, and similar genitalia shapes and lengths of features. These species can be differentiated by the mesopleura as they are broader on the anterior end in *Cryptophylliumliyananae* sp. nov. and slightly narrower in *Cryptophylliumdrunganum* comb. nov. and the overall size as *Cryptophylliumliyananae* sp. nov. are 88.0–92.0 mm long and the holotype *Cryptophylliumdrunganum* comb. nov. is 75.0 mm long. Males are presently unknown.

###### Distribution.

Presently only confirmed from two localities in Guangxi Province, Liuzhou prefecture-level city (Fig. 46A, C) and Guilin prefecture-level city (Fig. 46B). The other specimens within Liu (1993) with varying locality data which may represent additional localities have not been reviewed so it is possible that these may represent other species or range expansions.

###### Description.

**Female. *Coloration.*** Coloration descriptions are based upon photos of the live individuals which became the type material herein described (Fig. 46A, C). The overall coloration is pale mint green, with highlights of yellow, orange, and red. The antennae, interior profemoral lobe margins, and the margins of the terminal abdominal segments are red. The mesopleura margins, veins of the tegmina, and exterior profemoral lobe margins are orange to yellow.

***Morphology.****Head.* Head capsule about as long as wide, vertex smooth (Fig. 47D). The posteromedial tubercle is the only notable feature on the head capsule. Frontal convexity broad for the posterior half then narrowing on the anterior half, about as long as the first antennomere, and with slight granulation on the dorsal surface and several setae present which are longer than any setae on the antennae. Compound eyes slightly protruding from the head capsule, taking up ca. ¼ of the length of the lateral head capsule margins (Fig. 47D). Ocelli absent. Antennal fields slightly wider than the first antennomere and slightly shallower than the first antennomere is tall (Fig. 47B). *Antennae.* Antennae consisting of nine segments, with the terminal segment slightly shorter than the preceding two segment lengths combined (Fig. 47B). Antennomeres I–VIII sparsely marked with small transparent setae, the terminal antennomere is more densely covered in stout, brown setae. The *pars stridens* of antennomere III has 46 or 47 teeth. *Thorax.* Pronotum with gently concave anterior margin and straight lateral margins, which converge to a convex posterior margin that is ½ the width of the anterior margin (Fig. 47D). The pronotum surface is smooth, with only a prominent pit in the center, and slight furrows anterior, posterior, and lateral to the pit (Fig. 47D). The pronotum has a prominent anterior rim and weakly formed lateral and posterior rims, all of which are relatively smooth (Fig. 47D). Prosternum and the mesosternum with stout and numerous nodes, with the central area of the mesosternum with less nodes and relatively smooth. Metasternum with granulation reduced and only minimal. Prescutum anterior margin as wide as the presuctum is long, with margins slightly narrowing on the anterior ⅓ and then running parallel to the posterior margin which is slightly narrower than the anterior margin (Fig. 47D). Lateral rims with 7–9 short tubercles which are all about the same size (Fig. 47D). Prescutum anterior rim prominent but not strongly protruding, with a surface that is granular, lacking a singular prominent sagittal tubercle (Fig. 47D). Prescutum surface with minimal granulation throughout, with those along the sagittal plane only slightly larger (Fig. 47D). Mesopleura beginning near the anterior margin of the prescutum and evenly diverging; lateral margin with 9–13 tubercles which are variable in size, with three larger than the rest (Fig. 47D). Face of the mesopleura relatively smooth, and with two notable divots, one near the anterior margin and another ca. ⅗ of the way through the length (Fig. 47D). *Wings.* Tegmina reaching ca. ½ through abdominal segment VII. The subcosta (Sc) is the first vein in the forewing, is distinctly bent in the center, and terminates ca. ¼ of the way through the wing length. The radius (R) spans the anterior half of the forewing with two subparallel branched veins; radius 1 (R1) terminates ca. ⅓ of the way through the wing length, and the radial sector (Rs) terminates in the center of the wing at the widest portion. There is a weak continuation of the radius following the prominent Rs branching which continues on as a short and thinner R–M crossvein that does not appear to solidly connect the two veins fading as it reaches the media. The media (M) is bifurcate with both the media anterior (MA) and media posterior (MP) terminating on the posterior ⅓ of the wing. In some individuals there is a weak continuation of the media as a thin crossvein to the cubitus, but this was not present in all individuals observed. The cubitus (Cu) is also bifurcate, branching near the posterior ⅓ to ¼ of the wing into the cubitus anterior (CuA) and cubitus posterior (CuP) which both terminate at or very near the wing posterior apex. The first anal vein (1A) is simple and fuses with the cubitus early on, only slightly past the branching distance of the first radius from the radius. Alae well-developed, reaching abdominal segment VI (41.0–43.0 mm long). *Abdomen.* Abdominal shape relatively stable between all females observed. Segments II through the anterior ⅓ of IV evenly diverging, with the posterior ⅔ of segment IV the widest segment. Segments V–VI are slightly subparallel, converging gently to the posterior, giving the abdomen a slight narrowing appearance. Segment VII is distinctly rounded to the terminal three segments which are notably narrower than the previous segments (segment VIII on the anterior is slightly > ½ width of the widest portion of the abdomen). Segments VIII–X converge to the apex which is broad and rounded. *Genitalia.* Subgenital plate starts at the anterior margin of segment VIII, is moderately broad, and extends ca. ⅔ of the way onto segment X, ending in a fine point (Fig. 47E). Gonapophyses VIII are long and moderately broad, with their tips notably exceeding the apex of the abdomen, and slightly shorter than the tips of the cerci; gonapophyses IX are slender and long, extending ca. ¾ of the way onto segment X (Fig. 47E). Cerci flat, not strongly cupped, with a heavily granular surface (Fig. 47E). *Legs.* Profemoral exterior lobe broad (broader than the interior lobe), approximately right angled, and with a rounded exterior angle. The margin is marked by 7–9 small serrate teeth throughout the length, none prominent (Fig. 47C). Profemoral interior lobe narrower than the exterior lobe (only ca. 3× the greatest width of the profemoral shaft) and with a slightly obtuse angle giving the interior lobe a triangular appearance marked by five teeth on the distal margin (Fig. 47C). These teeth are arranged in a two-one-two pattern with the exterior pairs closer together and with a shallow gap between them, and the gap to the center tooth is deeper and wider than these exterior pairs (Fig. 47C). Mesofemoral exterior lobe arcs from end to end as a rounded triangle, but is slightly weighted towards the distal ½ and marked with two or three small serrate teeth distributed on the distal half only. Mesofemoral exterior lobe is slightly wider than the interior lobe. Mesofemoral interior lobe arcs end to end more evenly than the exterior lobe, is marked with five or six small serrate teeth only on the distal half of the arc. Metafemoral interior lobe arcs end to end but is significantly more heavily weighted on the distal half and has six or seven serrate teeth on the broader distal half of the lobe. Metafemoral exterior lobe is thin and smooth, hugging the metafemoral shaft. Protibiae interior lobe spans the entire length of the protibiae and is at its widest ca. 2× the width of the protibiae shaft itself. The lobe is distinctly triangular and widest on the distal half. There is a small but notable exterior protibial lobe on the distal quarter of the length, but this is only ca. ½ as wide as the protibial shaft itself. Mesotibiae simple, lacking lobes completely. Metatibiae lacks an interior lobe, but does have a small but notable anteriorly situated exterior lobe.

***Measurements of holotype female* [mm].** Length of body (including cerci and head, excluding antennae) 88.7, length/width of head 7.2/7.1, antennae 4.6, pronotum 5.3, mesonotum 7.5, length of tegmina 52.3, length of alae 41.5, greatest width of abdomen 37.9, profemora 19.7, mesofemora 15.0, metafemora 17.2, protibiae 12.6, mesotibiae 11.4, metatibiae 15.2.

***Measurements of paratype females* [mm].** Length of body (including cerci and head, excluding antennae) 90.8–92.0, length/width of head 7.5–7.7/6.6–7.3, antennae 4.9–5.3, pronotum 5.5–5.6, mesonotum 8.0–8.4, length of tegmina 50.5–53.8, length of alae 41.0–43.5, greatest width of abdomen 35.9–39.5, profemora 19.9–21.1, mesofemora 15.1–16.0, metafemora 17.6–18.0, protibiae 12.0–12.6, mesotibiae 10.8–11.0, metatibiae 15.6–15.9.

**Description of egg** (Fig. 8K, L). The entire capsule is covered in short moss-like pinnae and pitting of various size, depth, and spacing. The dorsal, ventral, and lateral surfaces are flattened, giving the egg a rectangular appearance. When viewed from the lateral aspect, the egg has an almost uniform width throughout. The lateral margins have slightly longer pinnae than the faces, but not drastically. Lateral surfaces slightly raised along the center of the length of the egg, and the entire surface has various shallow pitting in no detectable pattern. Micropylar plate spans the entire dorsal surface, with the thickest portion near the posterior ⅓ around the micropylar cup. The remainder of the micropylar plate is narrower, but not thin, at the thinnest still ca. ½ of the width of the widest portion. Operculum ovular and shallowly raised in the center and a singular circle of eight or nine shallow pits around the margin. Overall color light tan to brown.

**Measurements including the extended pinnae [mm].** Length (including operculum) 4.8–5.0, maximum width of capsule when viewed from lateral aspect 3.5–3.6 mm, length of micropylar plate 3.9–4.0 mm.

###### Etymology.

Patronym. The type specimens for this species were discovered by Chengzhi Bian (China) who recognized the scientific importance of the specimens and shared them with the authors to review. Chengzhi Bian has decided to name this species after his mother Liyanan to thank her for her amazing support of his passion for entomology.

##### Cryptophyllium nuichuaense

Taxon classificationAnimalia

gen. et sp. nov.

9DD319B2-D219-5D24-8366-348AE8D1C48F

http://zoobank.org/A801EC6F-5F97-4C17-A338-002FA9C9F112

**Figure 48.**
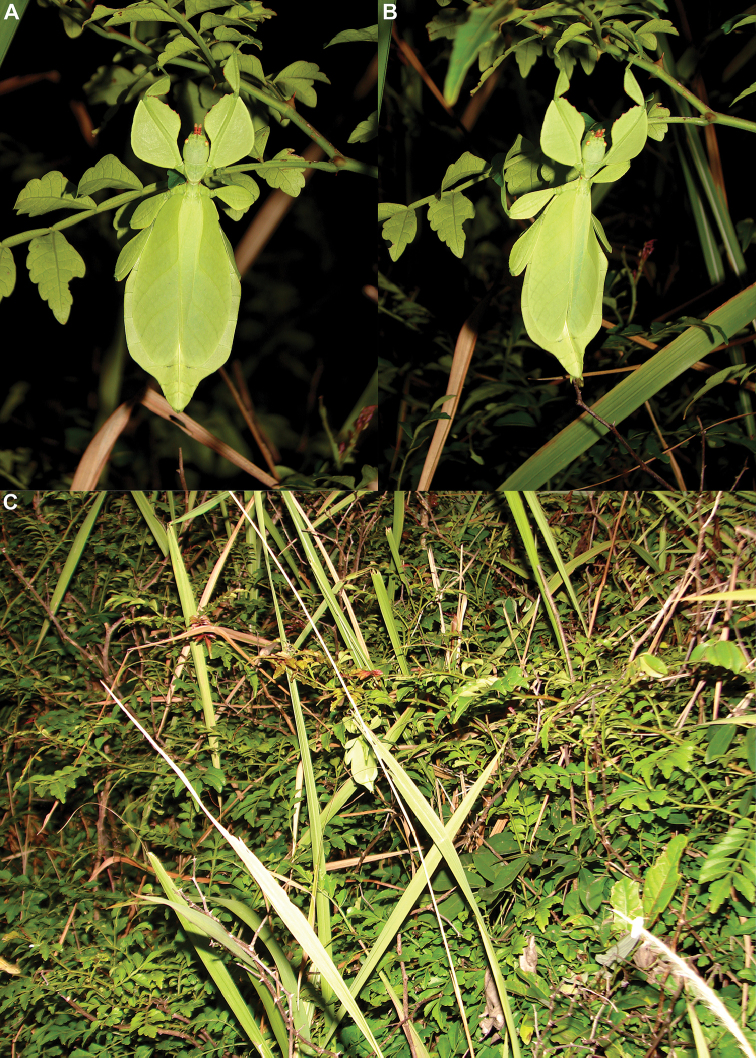
*Cryptophylliumnuichuaense* gen. et sp. nov. holotype female where she was found in August 2014 in Nui Chua N.P. by Jérôme Constant (RBINS) and Joachim Bresseel (RBINS). Photographs by Jérôme Constant (RBINS) **A** dorsal view **B** dorsolateral view **C** location where the female was found.

**Figure 49.**
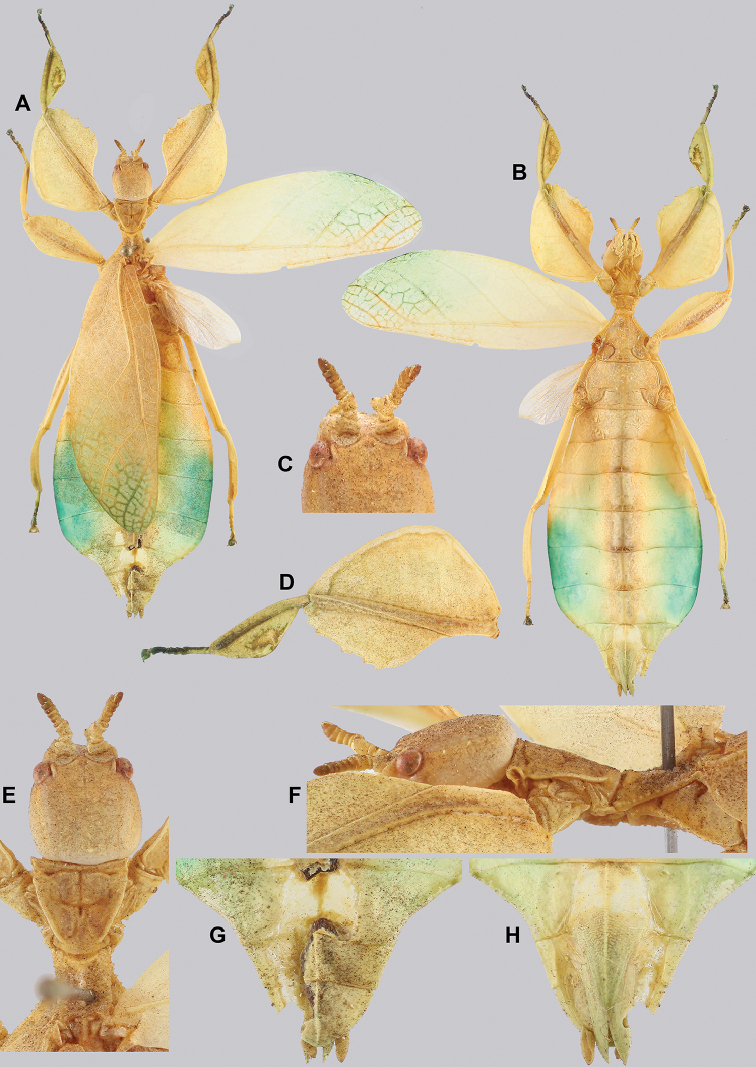
*Cryptophylliumnuichuaense* gen. et sp. nov. holotype female, photographs by Jérôme Constant (RBINS) **A** habitus, dorsal **B** habitus, ventral **C** details of the antennae and anterior half of the head capsule **D** pro- tibial and femoral lobes, dorsal **E** details of the antennae, head, and thorax, dorsal **F** details of the base of the antennae, head, and thorax, lateral **G** terminalia, dorsal **H** genitalia, ventral.

Figures 6E , 48 , 49

###### Material examined.

***Holotype*** ♀: “Coll. I.R.Sc.N.B., Vietnam, Ninh Thuan prov., Nui Chua N. P., 11°42’N 109°09’E, 3-9.VII.2014, night coll. Leg. J. Constant and J. Bresseel, GTI project I.G.:32.779, DNA PH006”. Deposited in the Royal Belgian Institute of Natural Sciences (RBINS).

***Paratype***: 1 ♀, “Coll. I.R.Sc.N.B., Vietnam, Ninh Thuan prov., Nui Chua N. P., 11°42’N 109°09’E, 3-9.VII.2014, night coll. Leg. J. Constant and J. Bresseel, GTI project I.G.:32.779” (VNMN).

###### Remarks.

This species was collected on a night walk in 2014 within Nui Chua National Park by Jérôme Constant (RBINS) and Joachim Bresseel (RBINS) (Fig. 48). Unfortunately, only two females were found, and this species could not be brought into breeding to reveal details about the egg, male, or freshly hatched nymph morphology. This is one of the many apparently highly endemic species within southern Vietnam, and we hope that future expeditions to this region reveal the male morphology and the range of this species with more clarity.

###### Differentiation.

Females are morphologically most similar to *Cryptophylliumphami* sp. nov. and *Cryptophylliumbollensi* sp. nov. due to their general femoral lobe shape, abdominal shape, femoral lobe spination, and thorax spination. When finer details are observed however these species can easily be differentiated. *Cryptophylliumnuichuaense* sp. nov. have slightly shorter alae which only reach onto the anterior of abdominal segment III, whereas the other species have longer alae reaching to the middle of segment III or even to the anterior margin of segment IV. Additionally, the antennae readily differentiate these three species as *Cryptophylliumnuichuaense* sp. nov. has antennal segments V, VI, and VII with ventral margins which project past the margin of segment VIII, giving the antennae a slightly lamellate appearance (Fig. 6E), vs. the other two species which have these segments ventral margin flush with the ventral margin of segment VIII (Fig. 6B, F).

Males are presently unknown, but due to the adult morphology we expect that they likely look similar to *Cryptophylliumphami* sp. nov. and *Cryptophylliumbollensi* sp. nov. males.

###### Distribution.

At present only known from Nui Chua N.P., in Ninh Thuan Province, Vietnam.

###### Description.

**Female. *Coloration.*** Coloration description is based upon the living type material (Fig. 48). Overall coloration is pale green throughout. The antennae, compound eyes, interior profemoral lobe margin, small patches along the protibial interior lobe, and the anterior of the prescutum margins are orange to red, but these areas are only sparsely marked.

***Morphology.****Head.* Head capsule about as long as wide, vertex relatively smooth with the only notable feature being the posteromedial tubercle which is finely pointed (Fig. 49E). Frontal convexity broad and blunt, with a slightly granular surface. Compound eyes slightly protruding from the head capsule, and are not particularly large, taking up slightly < ¼ of the head capsule lateral margins (Fig. 49E). Ocelli absent. Antennal fields slightly wider than the width of the first antennomere. *Antennae.* Antennae consisting of nine segments, with the terminal segment about the same length as the preceding two segments’ lengths combined (Fig. 49C). Antennomeres I–VII sparsely marked with small transparent setae, the terminal antennomere and antennomere VIII are covered in stout, brown setae (Fig. 49C). Antennomeres V–VII ventral margins project farther than the ventral margin of segment VIII, therefore giving the antennae a slight lamelatte appearance (Fig. 6E). *Thorax.* Pronotum with gently concave anterior margin and slightly convex lateral margins, which converge to a straight posterior margin that is slightly < half the width of the anterior margin (Fig. 49E). The pronotum surface is smooth, with only a prominent pit in the center, and slight furrows anterior and lateral to the pit (Fig. 49E). The pronotum has moderately formed anterior and lateral rims and a weakly formed posterior rim, all of which are relatively smooth (Fig. 49E). Prosternum and the anterior half of the mesosternum are covered with numerous nodes, the metasternum has lateral margins which are slightly granular, and the central area is relatively smooth. Prescutum longer than wide, lateral rims with four or five small tubercles on the anterior ⅓, the remainder only has nodes throughout, giving the margin a rough textured appearance (Fig. 49E). Prescutum anterior rim not strongly protruding, rim surface is granular, lacking a large sagittal spine (Fig. 49E). Prescutum surface granular, with those along the sagittal plane slightly larger than the rest (Fig. 49E). Mesopleura begin ca. ⅓ of the way through the prescutum length and evenly diverge; lateral margin with seven or eight small tubercles and several nodes interspersed (Fig. 49E). Face of the mesopleura smooth or slightly wrinkled, with two notable divots, one on the anterior margin and one near the middle (Fig. 49E). *Wings.* Tegmina long, reaching ½ through abdominal segment VII. Tegmina venation; the subcosta (Sc) is the first vein in the forewing, running parallel with the margin for the first half, and then bending and running towards the margin. The radius (R) spans the central portion of the forewing with two subparallel branched veins; the first radius (R1) branches ca. ¼ of the way through the wing length, terminates ca. ⅖ of the way through the wing length, and the radial sector (Rs) branches ca. ⅖ of the way through the wing length and terminates near the distal ⅓ of the wing length. There is a weak continuation of the radius following the prominent Rs branching which continues on as a short and thin R–M crossvein that weakly connects the two veins. The media (M) is simply bifurcate with both the media anterior (MA) and media posterior (MP) terminating near to the posterior ¼ of the wing. The cubitus (Cu) is also bifurcate, branching near the posterior ⅕ of the wing into the cubitus anterior (CuA) and cubitus posterior (CuP) which both terminate at or very near the wing posterior apex. The first anal vein (1A) is simple and fuses with the cubitus early on, at the length about midway between the splitting of the R1 and Rs. Alae short, with their apex only just passing the posterior margin of abdominal segment III or slightly passing onto the anterior margin of abdominal segment IV. *Abdomen.* Abdominal segments II through the anterior half of IV diverging. The posterior half of segment IV through segment VI are parallel, giving the abdomen a boxy appearance. Abdominal segment VII has a slightly rounded margin, no notable protruding lobe present. Segments VIII–X are notably narrower than the previous segments, and have converging margins to the broad rounded apex (Fig. 49G). *Genitalia.* Subgenital plate starts at the anterior margin of segment VIII, is moderately broad, and extends ca. ¾ of the way onto segment X with straight margins ending in a fine point (Fig. 49H). Gonapophyses VIII are long and moderately broad, exceeding the apex of abdominal segment X; gonapophyses IX are shorter and narrower, hidden below (Fig. 49H). Cerci only slightly cupped, with a granular surface and margins, and few detectable setae (Fig. 49G). *Legs.* Profemoral exterior lobe broad, rounded, and obtusely angled, smoothly arcing from end to end, ca. ⅓ again wider than the width of the interior lobe (Fig. 49D). Edge of the profemoral exterior lobe granular, or with a slightly serrate surface of four or five very small teeth (Fig. 49D). Profemoral interior lobe ca. 2½× as wide as the greatest width of the profemoral shaft, obtusely angled, and marked with five teeth arranged in a two-one-two pattern with shallow gaps between them (Fig. 49D). Mesofemoral exterior lobe arcs from end to end but is slightly bent in the center, weighted towards the distal ½, with a smooth proximal margin and a slightly lumpy distal half appearing to be weakly formed teeth. Interior and exterior mesofemoral lobes about the same width. Mesofemoral interior lobe arcs smoothly end to end with six or seven small serrate teeth only on the distal half of the arc which is slightly wider than the proximal half of the arc. Metafemoral interior lobe arcs end to end, with the distal half slightly wider than the proximal half and marked with seven or eight serrate teeth on the distal half of the lobe only. Metafemoral exterior lobe is thin and smooth, hugging the metafemoral shaft and lacks dentation. Protibiae lacking an exterior lobe (Fig. 49D). Protibiae interior lobe spans the entire length of the protibiae and is ca. 2½× the width of the protibiae shaft itself. The lobe is roundly triangular with the widest portion just slightly distal to the midline. Mesotibiae and metatibiae lacking exterior and interior lobes.

***Measurements of holotype female* [mm].** Length of body (including cerci and head, excluding antennae) 64.9, length/width of head 6.7/5.6, antennae 3.2, pronotum 4.5, mesonotum 5.9, length of tegmina 38.2, length of alae 15.2, greatest width of abdomen 22.0 (abdomen not perfectly flat), profemora 15.7, mesofemora 11.6, metafemora 14.2, protibiae 10.0, mesotibiae 8.2, metatibiae 11.4.

***Measurements of paratype female* [mm].** Length of body (including cerci and head, excluding antennae) 67.6, length/width of head 7.3/6.2, antennae 3.3, pronotum 4.9, mesonotum 6.2, length of tegmina 40.3, length of alae 16.4, greatest width of abdomen 24.6 (abdomen not perfectly flat), profemora 15.5, mesofemora 11.5, metafemora 15.9, protibiae 10.0, mesotibiae 8.6, metatibiae 11.8.

###### Etymology.

Toponym, named for the type locality, Nui Chua N.P. where this species was first discovered in Ninh Thuan Province, Vietnam.

##### Cryptophyllium oyae

Taxon classificationAnimalia

(Cumming & Le Tirant, 2020) comb. nov.

BD05CDBF-570B-51D9-8A88-77617AD023EB

**Figure 50.**
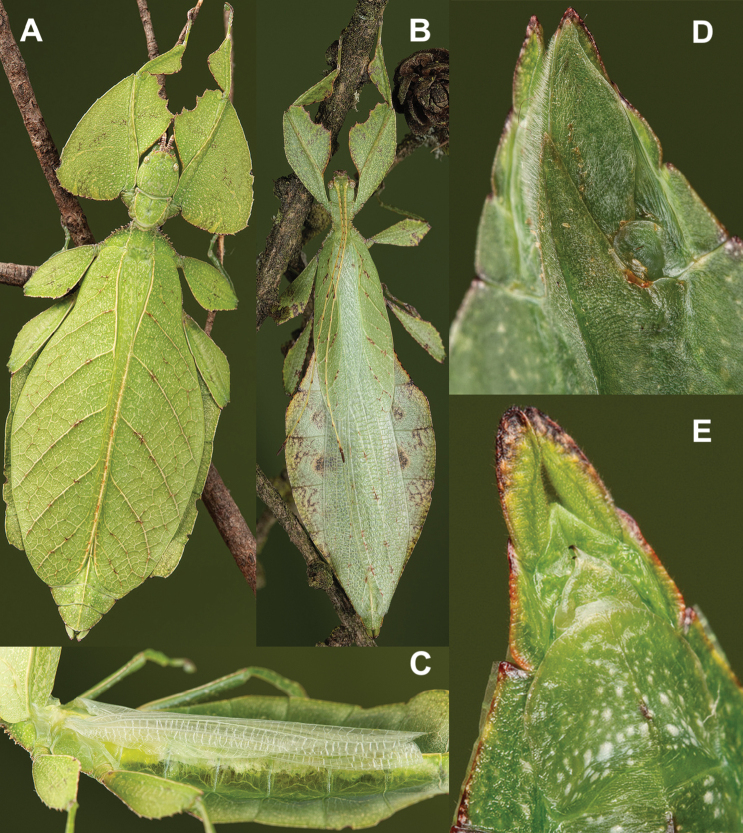
Live *Cryptophylliumoyae* comb. nov. bred and photographed by Bruno Kneubühler (Switzerland) **A** female **B** male **C** lateral view of female with tegmina held up to expose the alae **D** female genitalia, ventral **E** male genitalia, ventral.

**Figure 51.**
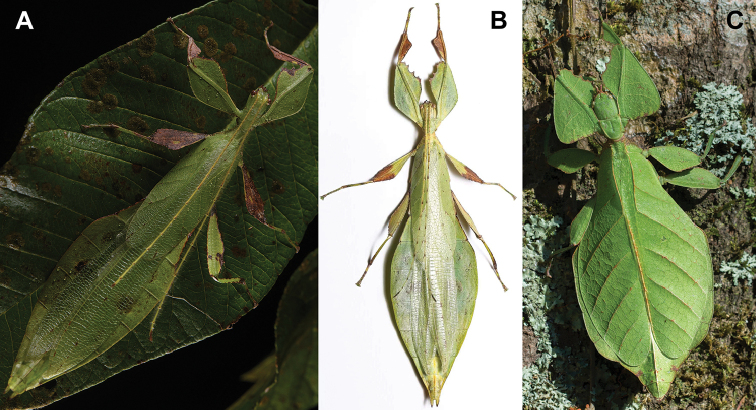
Live *Cryptophylliumoyae* males from additional, non-type localities **A** from Vietnam: Ha Giang, Dung Ba Commune, observed and photographed by Chien C. Lee (Malaysia) in January 2020 **B** from Thailand: Nan Province, Bo Kluea Tai District observed and photographed by Tatsatorn Dharithai (Thailand) in September 2019 **C** from Thailand: Chiang Mai Province, San Ku Ruins, observed and photographed by Rob Thacker (United Kingdom) in January 2021.

**Figure 52.**
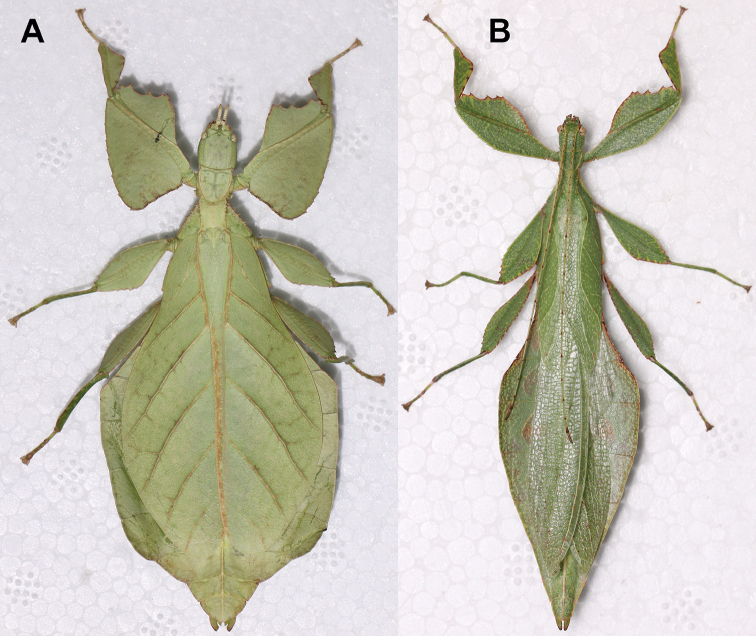
Live *Cryptophylliumoyae* pair from China, Yunnan Province, Maguan County, Gulinqing town (古林箐乡) collected by Xiang-Jing Liu (China) in October 2020, photographed by Zhiwei Dong (KIZ) **A** adult female dorsal **B** adult male dorsal.

Figures 5F , 8G , 8H , 9E , 50 , 51 , 52

###### Material examined.

The extensive paratype series within Coll RC and Coll SLT were examined to review the intraspecific variation within this species. See Cumming and Le Tirant (2020) for a detailed list of material examined. Additionally, 1 ♂: “Coll. I.R.Sc.N.B., Laos, NE, Mt Phu Phan, vi.2019, local collectors, I.G.: 34.159” [vomer dissected] (paratype male from original description) (RBINS).

***Photographic records***: 1 ♂, 1 ♀: “China, Yunnan Province, Maguan County, Gulinqing town (古林箐乡)” (observed by Xiang-Jing Liu, China); 1 ♂: “Vietnam, Ha Giang Province, Dung Ba Commune” (observed by Chien C. Lee, Malaysia); 1 ♂: “Thailand, Nan Province, Bo Kluea Tai District” (observed by Tatsatorn Dharithai, Thailand); 1 ♂: “Thailand: Phetchabun Province, Phetchabun Research Station” (Photograph shared by Tatsatorn Dharithai, Thailand); 1 ♀: “San Ku Ruins, Chiang Mai, Thailand” (observed by Rob Thacker, United Kingdom).

###### Remarks.

This species was only recently described and has entered the phylliid enthusiast breeding community in the last year or so (Fig. 50). Despite the relatively recent description, as a large prominent species it has been easy to identify from numerous observational records> from throughout mainland Asia, thus appearing to be a somewhat widespread species than was originally known (Fig. 2).

###### Differentiation.

Females are morphologically similar to *Cryptophylliumtibetense* comb. nov. and *Cryptophylliumyunnanense* comb. nov. due to the mesopleura which are prominent and reach uniformly from the anterior to the posterior of the prescutum and are marked with large prominent tubercles; the general femoral lobe shapes; the general abdominal shape with a boxy abdomen with a gently lobed abdominal segment VII; and due to the distal portion of the tibial exterior areas having small lobes. Both of these similar species can however be differentiated by the female subgenital plate as it is notably long in *Cryptophylliumtibetense* comb. nov. and *Cryptophylliumyunnanense* comb. nov. with the apex reaching the tip of the abdomen (Figs 63B, 77E), vs. *Cryptophylliumoyae* comb. nov. which has a subgenital plate which is shorter, only reaching ca. ½ through abdominal segment X (Fig. 50D).

Males are morphologically similar to *Cryptophylliumlimogesi* sp. nov. and *Cryptophylliumyunnanense* comb. nov. due to their large size and spade-shaped abdomens. *Cryptophylliumoyae* comb. nov. shares the following morphological similarities to *Cryptophylliumlimogesi* sp. nov.: similar femoral and tibial lobe shapes (particularly the profemoral exterior lobe which is broad, distinctly bent, and marked with prominent serration); and the occasional presence of small distal lobes of the tibial exteriors. *Cryptophylliumoyae* comb. nov. can be differentiated by the length of the tegmina as they reach significantly onto abdominal segment IV when folded, and in *Cryptophylliumlimogesi* sp. nov. the tegmina only reach ½ onto abdominal segment III, and the mesopleura of *Cryptophylliumlimogesi* sp. nov. are not as broad on the anterior as those of *Cryptophylliumoyae* comb. nov. which has mesopleura which are nearly straight margined, diverging evenly not gradually. Male *Cryptophylliumoyae* comb. nov. can be differentiated from *Cryptophylliumyunnanense* comb. nov. by the profemoral exterior lobe shape as it is slightly thinner than the interior lobe and smoothly arcing from end to end in *Cryptophylliumyunnanense* comb. nov. (Fig. 75B) but notably broad and clearly bent in the center like in *Cryptophylliumoyae* comb. nov.

###### Distribution.

Widely ranging through several countries with only inland records> to date, no coastal records> yet known. This species has been located in Vietnam, Ha Giang Province, Dung Ba Commune (observed by Chien C. Lee, Malaysia; Fig. 51A); the type locality of Laos, Houaphanh Province, Xam Neua District, Mount Phu Phan; China, Yunnan Province, Maguan County, Gulinqing town (古林箐乡) (observed by Xiang-Jing Liu, China; Fig. 52); and three localities from Thailand, one from Nan Province, Bo Kluea Tai District (observed by Tatsatorn Dharithai, Thailand, Fig. 51B), from San Ku Ruins, Chiang Mai (observed by Rob Thacker, United Kingdom; Fig. 51C), and from Phetchabun Province, Phetchabun Research Station.

##### Cryptophyllium parum

Taxon classificationAnimalia

(Liu, 1993) comb. nov.

553D7DA0-4C9C-5413-8356-2C995A6AEAD2

Figure 53

###### Material examined.

(1 ♀, 1 ♂): 1 ♂: “China: Hainan Island, Jianfengling Park: Tropical forest with saturated moisture at night, nymph found on 2m tall shrub: 13^th^ July 2019 leg. Yingtong Wang.” (Coll RC 19-156).

***Photographic records***: 1 ♀: “中国海南省乐东黎族自治县” (Ledong Li Autonomous County, Hainan Province, China) (iNaturalist user @chenhanlin) (https://www.inaturalist.org/observations/34767801)

###### Remarks.

**Figure 53.**
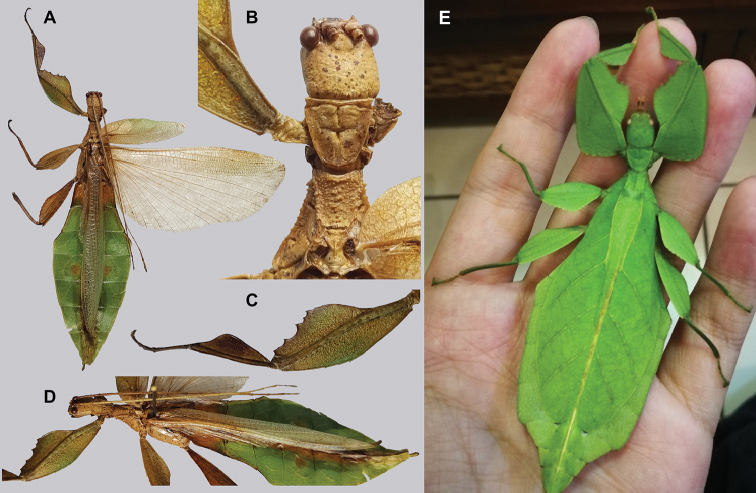
*Cryptophylliumparum* comb. nov. from Hainan **A–D** male which was used in our molecular analysis (Coll RC 19-156) from China: Hainan Island, leg. Yingtong Wang, photographs by RTC **A** habitus, dorsal **B** details of the head–thorax, dorsal **C** front leg details, dorsal **D** dorsolateral view **E** presumed female *Cryptophylliumparum* comb. nov. habitus, dorsal, from Ledong Li Autonomous County, Hainan Province, China, observed in December 2018 and uploaded to iNaturalist.com under the creative commons license (CC BY-NC 4.0) by user chenhanlin.

*Cryptophylliumparum* comb. nov. from Hainan Island, China is a little-known species with few records> to date (Fig. 53A). Originally described from a male holotype only, we have since seen a few additional male records> and a few presumed female records> from photographs (Fig. 53E). On Hainan island the only other presently known phylliid species is Phyllium (Pulchriphyllium) sinense Liu, 1990, therefore, the photographs of female *Cryptophyllium* gen. nov. we have seen from Hainan are likely the unknown *Cryptophylliumparum* comb. nov. female. We have not seen any additional records> of this species from the mainland which would represent a range expansion, instead we believe that this species is likely restricted to the island of Hainan. Interestingly, males of this small species with short to average length tegmina are molecularly sister species to *Cryptophylliumoyae* comb. nov. from the mainland, which is a rather large species with the longest tegmina presently known in the *Cryptophyllium* gen. nov. Females of both species also exhibit a striking size difference, with *Cryptophylliumoyae* comb. nov. rather large, and all records> we have seen of *Cryptophylliumparum* comb. nov. appearing to be rather small.

###### Differentiation.

The presumed females are morphologically similar to *Cryptophylliumoyae* comb. nov. and *Cryptophylliumtibetense* comb. nov. due to the prominent mesopleura which are broad and reach fully to the anterior margin of the prescutum and the overall boxy abdominal shape. *Cryptophylliumparum* comb. nov. can be differentiated from both by the serration of the mesopleura, as in *Cryptophylliumparum* comb. nov. the lateral margins only have granulation throughout the full length of even size, giving the lateral margins a rough textured appearance, vs. the other two species which have several prominent tubercles and smaller ones interspersed of differing sizes. In all the presumed female *Cryptophylliumparum* comb. nov. we have seen the profemoral exterior lobe shape was rather stable, nearly right angled, which is similar to certain forms of *Cryptophylliumoyae* comb. nov. which have rather variable exterior profemoral lobe shapes (see fig. 5 in Cumming and Le Tirant 2020 of the variable *Cryptophylliumoyae* comb. nov. lobes).

Males are most similar to *Cryptophylliumbollensi* sp. nov. due to the size, tegmina length, and wide profemoral exterior lobes with fine serration on the distal margin. These two species can be separated by the shape of the mesopleura as *Cryptophylliumbollensi* sp. nov. has mesopleura which are narrower on the anterior end and *Cryptophylliumparum* comb. nov. has broader mesopleura on the anterior end, much more similar to *Cryptophylliumoyae* comb. nov. mesopleura.

###### Distribution.

At present only known from a few localities on Hainan Island, China (Baisha Li Autonomous County and Ledong Li Autonomous County).

##### Cryptophyllium phami

Taxon classificationAnimalia

gen. et sp. nov.

19CD5EF7-61A9-57D8-9DA5-69CC8CCB7D94

http://zoobank.org/D4022B1A-0983-4D93-BB32-36FD96881F59

Figures 5C , 6F , 9G , 54 , 55 , 56 , 57 , 58

###### Material examined.

***Holotype*** ♂: “Coll. I.R.Sc.N.B., Vietnam, Cat Tien N.P., 11°26’N 107°26’E, 6-16.vii.2012, Leg. J. Constant and J. Bresseel, I.G.: 32.161”. Deposited in RBINS.

***Paratypes*** (5 ♀♀, 4 ♂♂): • 1 ♀: same data as HT (RBINS) • 1 ♂, 3♀♀: same data as HT, “Ex Breeding: Bruno Kneubühler” (2♀♀: RBINS♂; 1, • 1♀: VNMN) • 1 ♂: same data as HT, “Ex Breeding Tim Bollens” [damaged; vomer dissected] (RBINS) • 1 ♀: same data as HT, “Ex Breeding Tim Bollens” (RBINS) • 1 ♂: “Vietnam, Dong Nai Province, Cat Tien N.P., bred by Bruno Kneubühler (Switzerland), circa 2012” (Coll OC) • 1 ♂: “Vietnam, Binh Thuan Province, Dong Tien, IV.2019”, molecular sample SLT03 in our analysis (Coll SLT).

###### Remarks.

**Figure 54.**
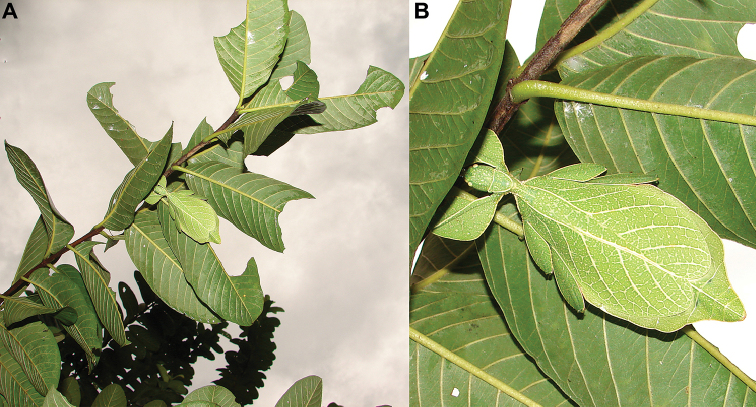
Paratype female *Cryptophylliumphami* gen. et sp. nov. where she was found in July 2012 by Joachim Bresseel (RBINS) and Jérôme Constant (RBINS) in the Cat Tien N.P., Vietnam. This is the same individual as in Figure 1, but these photos were taken with flash. Photographs by Jérôme Constant (RBINS) **A** branch the female was found on, note the leaves which show signs of the damage which led to her discovery **B** same individual, zoomed in to show details.

**Figure 55.**
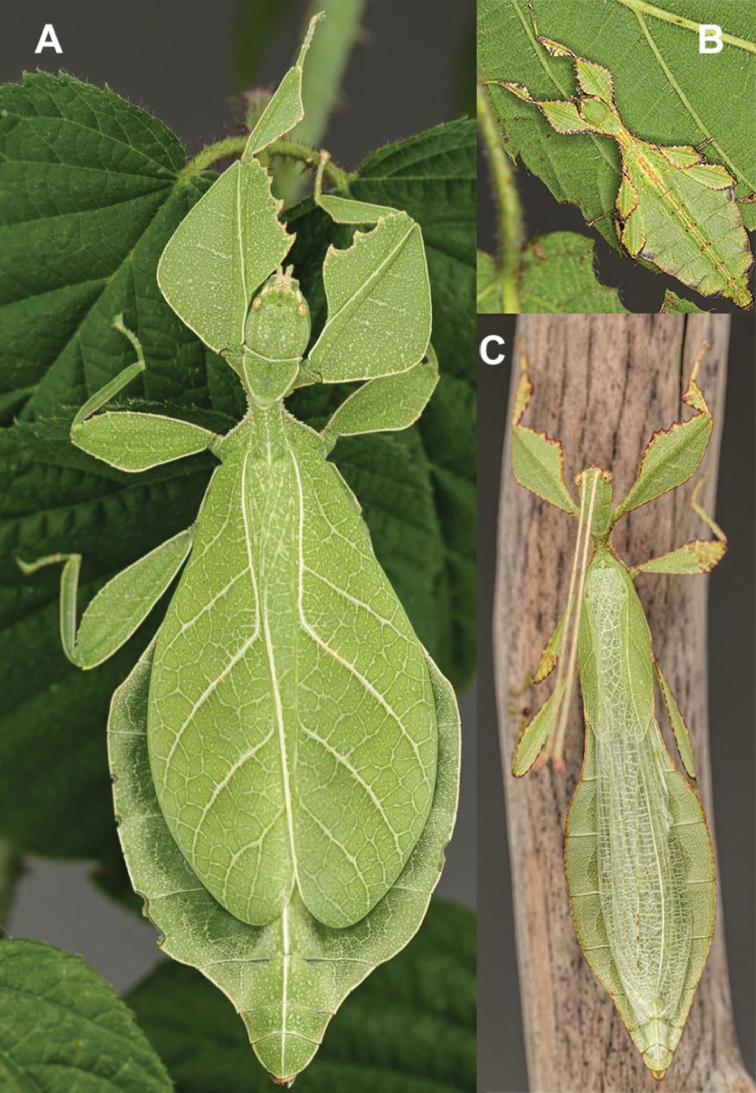
Live *Cryptophylliumphami* gen. et sp. nov. bred and photographed by Bruno Kneubühler (Switzerland) **A** adult female, dorsal **B** nymph, dorsal **C** adult male, doorsal.

This species was found in July 2012 by Joachim Bresseel (RBINS) and Jérôme Constant (RBINS) in Cat Tien N.P., Vietnam, during a GTI research expedition. A female was located due to the tell-tale sign of feeding damage on the guava tree on which she was found (Fig. 54) at crocodile lake between the ranger station and the lake shore. This female was kept alive long enough to lay fertilized eggs which were shared with expert phylliid breeder Bruno Kneubühler (Switzerland) who brought this species into culture (Fig. 55). Additionally, one male was collected while light trapping near the park headquarters.

###### Differentiation.

Females are most morphologically similar to *Cryptophylliumbollensi* sp. nov., *Cryptophylliumchrisangi* comb. nov., and *Cryptophylliumnuichuaense* sp. nov. based on the general abdominal shape, the broad obtuse exterior profemoral lobe, and the mesopleura shape and spination. *Cryptophylliumphami* sp. nov. can immediately be differentiated from these species by the length of the alae which are short, only reaching to the anterior margin of abdominal segment III vs. the other species which have moderate length alae, reaching at least onto abdominal segment IV.

Males are morphologically most similar to *Cryptophylliumwestwoodii* comb. nov., *Cryptophylliumchrisangi* comb. nov., *Cryptophylliumbollensi* sp. nov., and *Cryptophylliumkhmer* sp. nov. based on the tegminal length, femoral lobe shapes and spination, and the features of the thorax. The first two species can be differentiated by their abdominal shape which is thinly elliptical, with a maximum width only 30–34% the abdominal length vs. *Cryptophylliumphami* sp. nov. and the second two species which have an abdominal shape that is broadly elliptical or broadly spade-shaped with a maximum width ca. 38–45% the abdominal length. Due to their very similar morphology and intraspecific variation, we have not yet found a reliable morphological feature to differentiate *Cryptophylliumphami* sp. nov., *Cryptophylliumbollensi* sp. nov., and *Cryptophylliumkhmer* sp. nov. males. Thankfully females can help differentiate the species as noted above.

###### Distribution.

At present only known from two southern Vietnamese provinces, Dong Nai and Binh Thuan Provinces.

###### Description.

**Female. *Coloration.*** Coloration description is based upon both living wild caught (Fig. 54B) and captive reared individuals (Fig. 55A) as their coloration appears to be rather similar. Overall coloration is pale green, with many areas of the body highlighted with cream or very pale green. These areas tend to be the margins on the lobes of the legs, some striping on the lobes of the legs, the pronotum, abdominal margins, and the venation in the tegmina (Fig. 55A).

**Figure 56.**
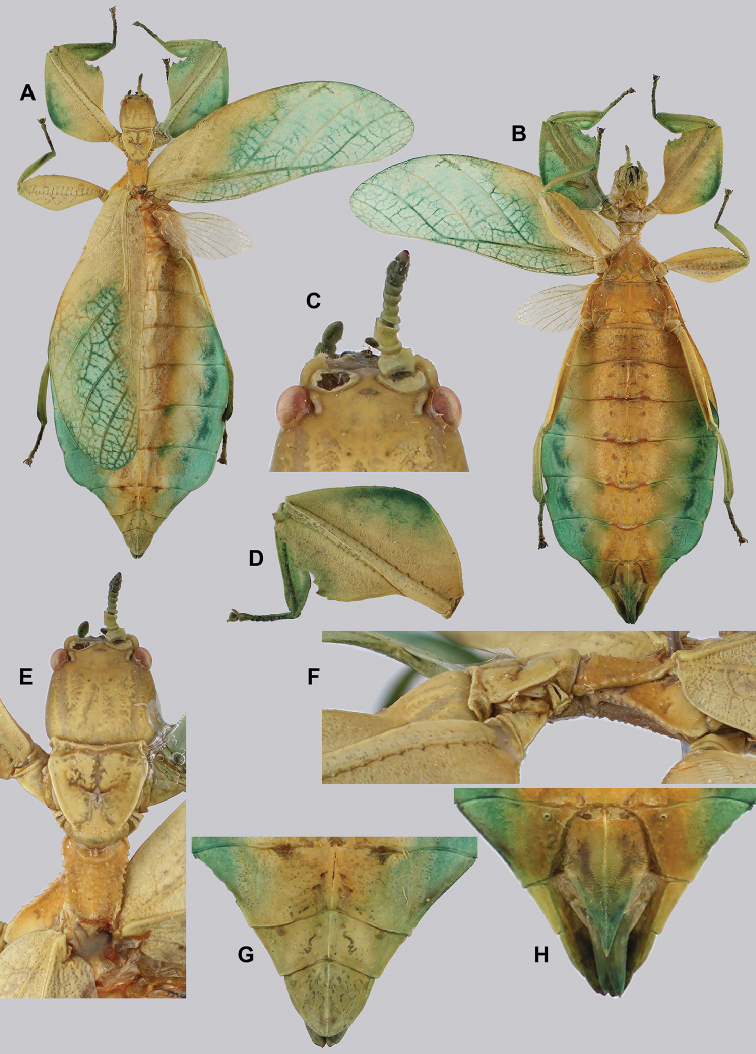
*Cryptophylliumphami* gen. et sp. nov. paratype female (RBINS), photographs by Jérôme Constant (RBINS) **A** habitus, dorsal **B** habitus, ventral **C** details of antennae and anterior of head capsule **D** profemoral and tibial lobes, dorsal **E** details of antennae, head, and thorax, dorsal **F** details of head and thorax, lateral **G** terminalia, dorsal **H** genitalia, ventral.

***Morphology.****Head.* Head capsule about as long as wide, vertex relatively smooth with the only notable feature being the posteromedial tubercle which is finely pointed (Fig. 56E). Frontal convexity broad and blunt, with a slightly granular surface. Compound eyes slightly protruding from the head capsule, and are not particularly large, taking up slightly < ⅓ of the head capsule lateral margins (Fig. 56E). Ocelli absent. Antennal fields slightly wider than the width of the first antennomere. *Antennae.* Antennae consist of nine segments, with the terminal segment about the same length as the preceding two and a half segments’ lengths combined (Fig. 56C). Antennomeres I–VII sparsely marked with small transparent setae, the terminal two antennomeres are covered in stout, brown setae. *Thorax.* Pronotum with gently concave anterior margin and slightly convex lateral margins, which converge to a straight posterior margin that is half the width of the anterior margin (Fig. 56E). The pronotum surface is smooth, with only a prominent pit in the center, and slight furrows anterior and lateral to the pit (Fig. 56E). The pronotum has moderately formed anterior and lateral rims and a weakly formed posterior rim, all of which are relatively smooth (Fig. 56E). Prosternum and the anterior of the mesosternum are covered with numerous nodes, but the remainder of the mesosternum and the metasternum are relatively smooth. Prescutum longer than wide, lateral rims with nine to eleven small to medium tubercles, not ranging dramatically in size giving the margin a rough textured appearance (Fig. 56E). Prescutum anterior rim prominent but not strongly protruding, rim surface is granular, lacking a large sagittal spine (Fig. 56F). Prescutum surface heavily granular, with all about the same size. Mesopleura begin ca. ⅓ of the way through the prescutum length and evenly diverge; lateral margin with seven or eight small tubercles with ca. ½ of those slightly larger than the rest, with the smaller ones interspersed throughout (Fig. 56E). Face of the mesopleura smooth, with two notable divots, one on the anterior margin and one near the middle (Fig. 56F). *Wings.* Tegmina long, reaching ½ through abdominal segment VII. Tegmina venation; the subcosta (Sc) is the first vein in the forewing, running parallel with the margin for the first half, and then bending and running towards the margin. The radius (R) spans the central portion of the forewing with two subparallel branched veins; the first radius (R1) branches ca. ¼ of the way through the wing length and terminates slightly proximal to the midline, the radial sector (Rs) branches ca. ⅖ of the way through the wing length and terminates near the distal ⅓ of the wing length. There is a weak continuation of the radius following the prominent Rs branching which continues on as a short and thin R–M crossvein that connects the two veins. The media (M) is simply bifurcate with both the media anterior (MA) and media posterior (MP) terminating near to the posterior ¼ of the wing. The cubitus (Cu) is also bifurcate, branching near the posterior ⅕ of the wing into the cubitus anterior (CuA) and cubitus posterior (CuP) which both terminate at or very near the wing posterior apex. The first anal vein (1A) is simple and fuses with the cubitus early on, at the length about midway between the splitting of the R1 and Rs. Alae reduced, with their apex only just passing the anterior margin of abdominal segment III. *Abdomen.* Abdominal segments II through the anterior half of IV uniformly diverging. The posterior half of segment IV through segment VII are subparallel, gradually converging, and segment VII is ending in a slightly rounded lobe. Segments VIII–X are notably narrower than the previous segments, and have converging margins to the broad rounded apex (Fig. 56G). *Genitalia.* Subgenital plate starts at the anterior margin of segment VIII, is moderately broad, and extends ½ onto segment X with straight margins ending in a fine point (Fig. 56H). Gonapophyses VIII are long and moderately broad, reaching the apex of abdominal segment X; gonapophyses IX are shorter and narrower, hidden below (Fig. 56H). Cerci flat, not strongly cupped, with a granular surface and few detectable setae (Fig. 56H). *Legs.* Profemoral exterior lobe broad, rounded, and obtusely angled, smoothly arcing from end to end, ca. ⅓ again wider than the width of the interior lobe (Fig. 56D). Edge of the profemoral exterior lobe granular, with a few slightly larger than the rest, but none very large to resemble teeth (Fig. 56D). Profemoral interior lobe ca. 2× as wide as the greatest width of the profemoral shaft, obtusely angled, and marked with five teeth arranged in a two-one-two pattern with looping gaps between them (Fig. 56D). Mesofemoral exterior lobe arcs from end to end but is slightly bent in the center, weighted towards the distal ½, and marked with three or four small serrate teeth distributed on the distal half only. Interior lobe is about the same width as the mesofemoral shaft, and the exterior lobe is slightly wider. Mesofemoral interior lobe arcs smoothly end to end with 6–8 small serrate teeth only on the distal half of the arc which is slightly wider than the proximal half of the arc. Metafemoral interior lobe arcs end to end, with the distal half slightly wider than the proximal half and marked with 7–10 serrate teeth on the distal half of the lobe. Metafemoral exterior lobe is thin and smooth, hugging the metafemoral shaft and lacks dentation. Protibiae lacking an exterior lobe. Protibiae interior lobe spans the entire length of the protibiae and is slightly > 2× the width of the protibiae shaft itself. The lobe is roundly triangular with the widest portion on the distal half. Mesotibiae and metatibiae lacking exterior and interior lobes.

***Measurements of paratype females* [mm] (wild caught).** Length of body (including cerci and head, excluding antennae) 76.7, length/width of head 7.9/6.1, antennae 3.8, pronotum 5.1, mesonotum 6.8, length of tegmina 45.8, length of alae 17.9, greatest width of abdomen 30.0, profemora 17.6, mesofemora 13.8, metafemora 17.1, protibiae 11.6, mesotibiae 9.8, metatibiae 12.7.

***Measurements of paratype females* [mm] (ex culture)** ^3^

. Length of body (including cerci and head, excluding antennae) 79.4–90.3, length/width of head 8.2–9.5/6.4–7.4, antennae 3.8–4.3, pronotum 5.1–5.8, mesonotum 7.0–7.5, length of tegmina 45.3–53.8, length of alae 17.2–22.9, greatest width of abdomen 28.7–36.3, profemora 17.8–20.7, mesofemora 14.7–15.6, metafemora 16.6–19.5, protibiae 11.5–12.0, mesotibiae 10.3–11.0, metatibiae 13.4–14.8.

**Male. *Coloration.*** Coloration based upon live bred specimens in captivity (Fig. 55C). Overall coloration pale green throughout with variable patches of tan to reddish coloration (Fig. 55C). These tan to reddish areas are primarily around the margins of the lobes of the legs, the margins of the thorax, the tips of the antennae, and the margins of the abdomen. In darker colored specimens the mesofemoral lobes can also have coloration, not just along the margins. Abdominal segment V has a pair of slightly transparent eye spots.

**Figure 57.**
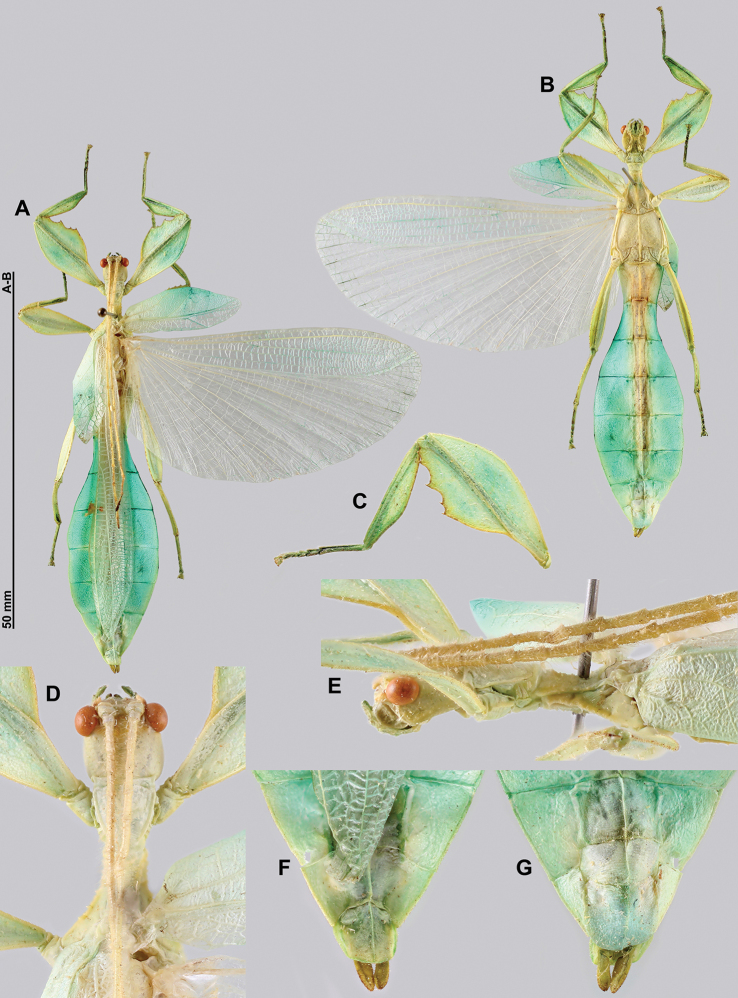
*Cryptophylliumphami* gen. et sp. nov. holotype male (RBINS), photographs by Jérôme Constant (RBINS) **A** habitus, dorsal **B** habitus, ventral **C** front leg, dorsal **D** details of base of antennae and head–thorax, dorsal **E** details of head and thorax, lateral **F** terminalia, dorsal **G** genitalia, ventral.

***Morphology.****Head.* Head capsule about as long as wide, with a vertex that is relatively smooth with only sparse granulation throughout. Frontal convexity stout with sparse thin setae. The posteromedial tubercle is not broad but is distinctly raised from the head capsule. Compound eyes large and bulbous, taking up slightly < ½ head capsule lateral margins (Fig. 57D). There are three well-developed ocelli located between and slightly posterior to the compound eyes. *Antennae.* Antennae (including the scapus and pedicellus) consists of 24 segments, all segments except the scapus and pedicellus and terminal three segments are covered in dense setae that are as long as or longer than the antennae segment is wide. The terminal three segments are covered in dense short setae and the scapus and pedicellus are nearly completely bare. *Thorax.* Pronotum with anterior margin distinctly concave and lateral margins that are slightly convex and converging to a straight posterior margin that is ca. ½ the width of the anterior rim. Anterior and lateral margins of the pronotum have moderately formed rims and the posterior margin lacks a rim. Face of the pronotum is marked by a distinct furrow and pit in the center and a relatively smooth surface with weak granulation. Prosternum surface is weakly granular with small nodes of even size and spacing. Mesosternum surface marked with slightly more prominent nodes, with the largest along the sagittal plane and more strongly on the anterior margin, posterior margin with less prominent and smaller nodes. Prescutum slightly longer than wide, with lateral margins that are only slightly converging to the posterior (Fig. 57D). Lateral rims with nine or ten node-like tubercles, giving the lateral margins a rough textured appearance. Prescutum surface with minimal nodes throughout, with those along the sagittal plane slightly larger than the others. Prescutum anterior rim prominent but not strongly raised, with a granular surface and lacking a prominent sagittal tubercle. Mesopleura begin on the anterior prescutum margin but are narrow throughout most of their length, only diverging gently for the posterior ⅔. Lateral margin with nine or ten minor tubercles throughout the length except for the posterior ⅓ which is relatively smooth. Face of the mesopleura mostly smooth, with slight wrinkling throughout. *Wings.* Tegmina moderate length, extending ⅓ of the way through abdominal segment III. Tegmina wing venation: the subcosta (Sc) is the first vein, is simple, and terminates the earliest ca. ⅓ of the way through the overall tegmina length. The radius (R) spans the entire length of the tegmina with the first radius (R1) branching just proximal to the midline and terminating just distal to the midline, followed by the branching and termination of the second radius (R2) near the distal ⅓ of the wing, and then the radial sector runs to the wing apex. The media (M) also spans the entire length of the tegmina with the first media posterior (MP1) branching off slightly > ⅓ of the way through the wing length, and then the second media posterior (MP2) branches just distal to the midline, and the media anterior (MA) runs to the wing apex. The cubitus (Cu) runs along the edge of the wing as the two media posterior veins fuse with it and as the cubitus reaches the apex it fades. The first anal (1A) vein terminates upon reaching the cubitus ca. ⅓ of the way through the wing length. Alae well-developed in an oval fan configuration, long, reaching to the middle or posterior of abdominal segments IX. Alae wing venation: the costa (C) is present along the entire foremargin giving stability to the wing. The subcosta (Sc) is long, spanning ca. ⅔ of the wing length and is mostly fused with the radius in the beginning but terminates when it meets the costa. The radius (R) spans the entire wing and branches slightly proximal to the midline into the first radius (R1) and radial sector (Rs) which run gently diverging for most of their length and then converge at the apex of the wing where they terminate near each other but not touching. The media (M) branches early, ca. ⅙ of the way through the wing into the media anterior (MA) and the media posterior (MP) which run parallel with each other throughout the wing until the distal ⅕ of the wing where the media posterior fuses with the media anterior which then run fused together to the wing apex where they terminate near the radial sector. The cubitus (Cu) runs unbranched and terminates at the wing apex. Of the anterior anal veins, the first anterior anal (1AA) fuses with the cubitus near the point where the media branches into the media anterior and media posterior and then the first anterior anal branches from the cubitus ⅔ of the way through the wing length where it uniformly diverges from the cubitus until it terminates at the wing margin. The anterior anal veins two–seven (2AA–7AA) have a common origin and run unbranched in a folding fan pattern of relatively uniform spacing to the wing margin. The posterior anal veins (1PA–6PA) share a common origin separate from the anterior anal veins and run unbranched to the wing margin with slightly thinner spacing than the anterior anal veins. *Abdomen.* Lateral margins of abdominal segment II are parallel, III through the anterior ⅔ of segment IV gradually diverging, the remainder of IV and segment V are parallel-sided, segment VI starts parallel-sided but almost immediately starts to converge and the remaining segments converge uniformly to the rounded apex of the abdomen. *Genitalia.* Poculum broad and ends in a rounded apex that slightly passes the anterior margin of segment X (Fig. 57G). Cerci long and slender, extending from under the anal abdominal segment, nearly flat, not strongly cupped, covered in a granulose surface and numerous short setae (Fig. 57F). Vomer broad and stout with straight sides evenly converging and ending in a thick apical hook with a smaller second hook adjacent to it (Fig. 5C). *Legs.* Profemoral exterior lobe about the same width as the interior lobe, ca. 2½× the greatest width of the profemoral shaft, roundly arcing end to end in a broad obtuse angle that is not distinctly bent, with the proximal margin slightly granulose, and the distal margin with four or five small serrate teeth (Fig. 57C). Profemoral interior lobe roundly triangular and marked with five sharp teeth arranged in a two-one-two pattern with looping gaps between them (Fig. 57C). Mesofemoral exterior lobe arcs end to end but is slightly wider on the distal ⅓ which is marked with four or five serrate teeth, and the proximal half that is rather thin. Mesofemoral interior lobe is about the same width as the exterior, is broader on the distal end and is marked with seven or eight small serrate teeth. Metafemoral exterior lobe lacks dentation and has a straight margin along the metafemoral shaft. Metafemoral interior lobe smoothly arcs end to end with eight or nine small serrate teeth on the distal ⅔, which is slightly wider than the proximal ⅓. Protibiae lacking exterior lobe, interior lobe reaching end to end in a smooth triangle which is slightly weighted to the distal ½ and at its widest is ca. 2½× as wide as the protibial shaft (Fig. 57C). Meso- and metatibiae simple, lacking lobes completely.

***Measurements of holotype male* [mm].** Length of body (including cerci and head, excluding antennae) 58.7, length/width of head 3.8/3.5, antennae 38.2, pronotum 2.9, mesonotum 3.8, length of tegmina 17.3, length of alae 42.9, greatest width of abdomen 13.4, profemora 12.8, mesofemora 10.6, metafemora 13.0, protibiae 10.1, mesotibiae 7.6, metatibiae 10.0.

***Measurements of paratype male* [mm] (ex culture).** ^4^

Length of body (including cerci and head, excluding antennae) 54.3, length/width of head 4.0/3.1, antennae 36.1, pronotum 2.7, mesonotum 4.3, length of tegmina 15.5, length of alae 41.1, greatest width of abdomen 13.7, profemora 11.6, mesofemora 10.1, metafemora 12.2, protibiae 8.0^5^, mesotibiae 7.4, metatibiae 8.9.

**Figure 58.**
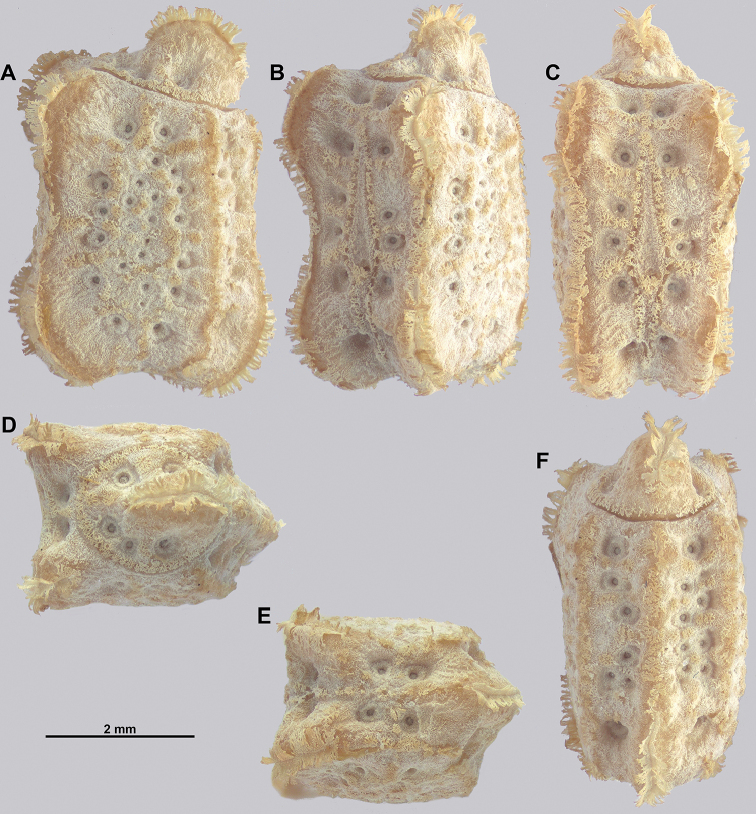
*Cryptophylliumphami* gen. et sp. nov. egg, RBINS collection, photographs by Jérôme Constant **A** lateral view **B** dorso-lateral view **C** dorsal view **D** opercular (anterior) view **E** posterior view **F** ventral view.

**Eggs.** (Fig. 58). The lateral surfaces are flat but with the posterior half slightly wider than the anterior half. The center of the dorsal surface is slightly convex, which gives the margin a slight undulating appearance when viewed from the lateral aspect as the middle is thinner than either end of the egg. When viewed from the lateral aspect; the ventral margin has the posterior slightly protruding more than the anterior, adding to the overall undulating shape of the egg. All surfaces have numerous small to medium sized pits throughout, the lateral surface has around 20 pits arranged in no particular order. The surfaces are also covered with short moss-like pinnae interspersed throughout the capsules with those along the margins slightly longer than the other surfaces. The dorsal surface is marked with six slightly irregular medium sized pits on each half and short moss-like pinnae around the micropylar plate. The micropylar plate is long, ca. 6/7 of the overall dorsal surface length and the shape is nearly symmetrical with the anterior and posterior thin and the area around the micropylar cup the widest point. Micropylar cup of moderate size and placed slightly posterior to the micropylar plate midline. Operculum slightly ovular, with the outer margin with a distinct row of moss-like pinnae surrounding the operculum and four or five medium pits surrounding the dorsal and lateral margins. The operculum is roundly raised with a height slightly > ½ operculum width, this rounded raised cap is marked with a sagittal raised row of pinnae similar in length to those along the capsule margins. The overall color is tan to light brown, with the moss-like pinnae sometimes slightly lighter in color.

**Measurements including the extended pinnae [mm].** Length (including operculum): 5.1; maximum width of capsule when viewed from lateral aspect 3.2–3.3; length of micropylar plate 2.9–3.2

**Newly hatched nymphs.** (Fig. 9G). The general color throughout the body is dark brown with slightly lighter brown on the legs. The basitarsi are yellow and remaining tarsal segments are dark brown. All tibiae lack exterior lobes, and all have smoothly arcing interior lobes which have several tan to brown stripes throughout their length. All femoral lobes are similar in width and have distinct serration on their distal halves. The interior profemoral lobe lacks a white spot, but the exterior lobe has a narrow white crescent on the proximal ⅓ with an additional small white patch at the proximal most margin. The meso- and metafemoral interior lobes have two white patches, one on the proximal most edge, and a larger white patch ⅓ of the way through the length. The meso- and metafemoral exterior lobes also have the large white patch on the proximal ⅓, but lack a smaller white patch on the proximal most margin. The distal ends of the meso- and metafemora also have minimal white edges. The abdomen is mostly brown, but abdominal segments II and III have distinct green patches on their lateral surfaces (the centerline of the abdomen is uniform brown throughout). The terminal three abdominal segments also have a little bit of green on their margins. The widest point of the abdomen is abdominal segment IV.

###### Etymology.

Patronym. This species is dedicated to Pham Hong Thai (VNMN), a good friend and colleague who co-organized the GTI entomological expeditions to Vietnam with the RBINS team since 2010.

##### Cryptophyllium rarum

Taxon classificationAnimalia

(Liu, 1993) comb. nov.

64EBAECA-F9BB-521E-AAB4-00E22E3CB59E

**Figure 59.**
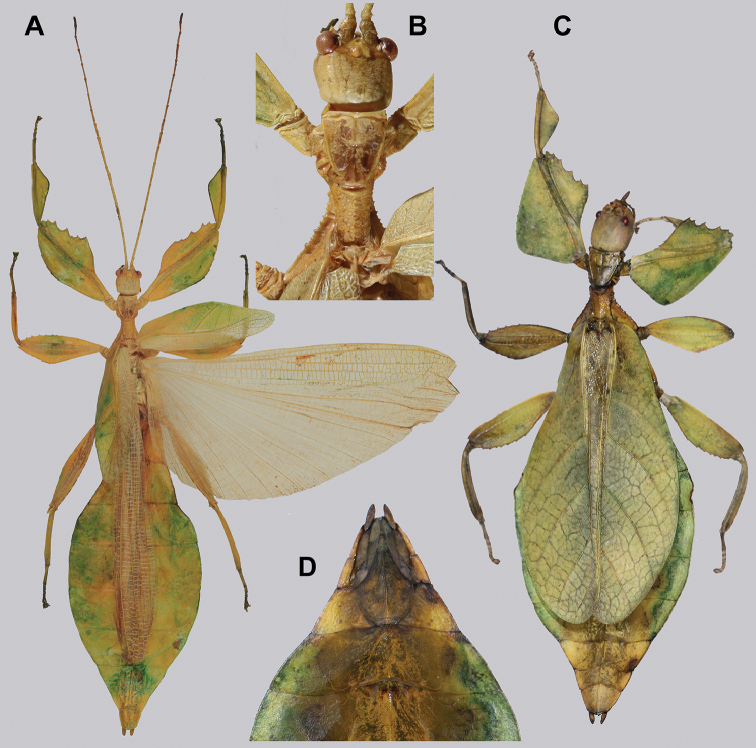
*Cryptophylliumrarum* comb. nov. male and female **A** male, habitus, dorsal, molecular sample within this study Coll RC 16-115 from Vietnam, Da Nang Province, photograph by RTC **B** details of the head and thorax, dorsal, Coll RC 16-115, photograph by RTC **C** female, habitus, dorsal, from China: Guangxi Province, Nanning city, Shuangding town, molecular sample DZW08 within this study, Photographs by Zhiwei Dong (KIZ) **D** female, genitalia, ventral, photographs by Zhiwei Dong (KIZ).

**Figure 60.**
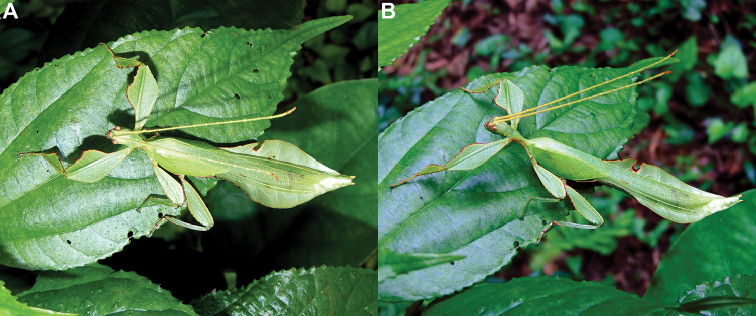
*Cryptophylliumrarum* comb. nov. male collected by Jérôme Constant (RBINS) and Joachim Bresseel (RBINS) in Cuc Phuong N.P., Vietnam in August 2016. Photographs by Jérôme Constant (RBINS). This male is molecular sample RBINS04 in our analysis.

**Figure 61.**
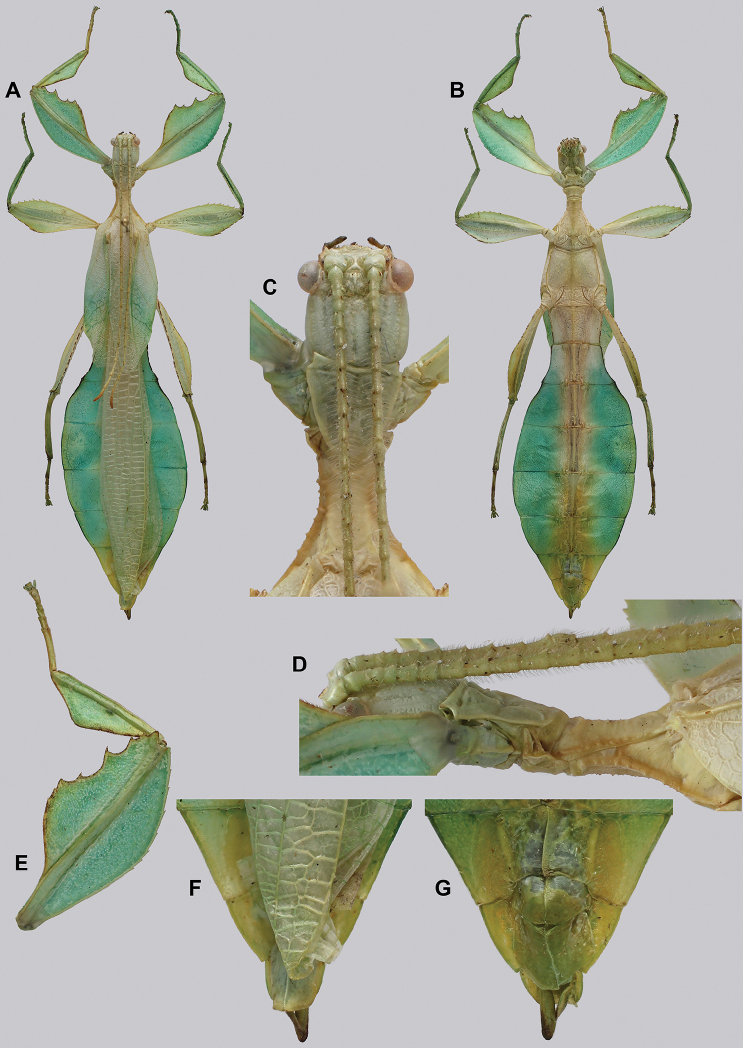
*Cryptophylliumrarum* comb. nov. male from Cuc Phuong N.P., Ninh Binh Province, Vietnam collected by Jérôme Constant and Joachim Bresseel (RBINS), photographs by Jérôme Constant (RBINS) **A** habitus, dorsal **B** habitus, ventral **C** details of the base of antennae, head, and thorax, dorsal **D** details of the base of antennae, head, and thorax, lateral **E** pro- femoral and tibial lobes, dorsal **F** terminalia, dorsal **G** genitalia, ventral.

**Figure 62.**
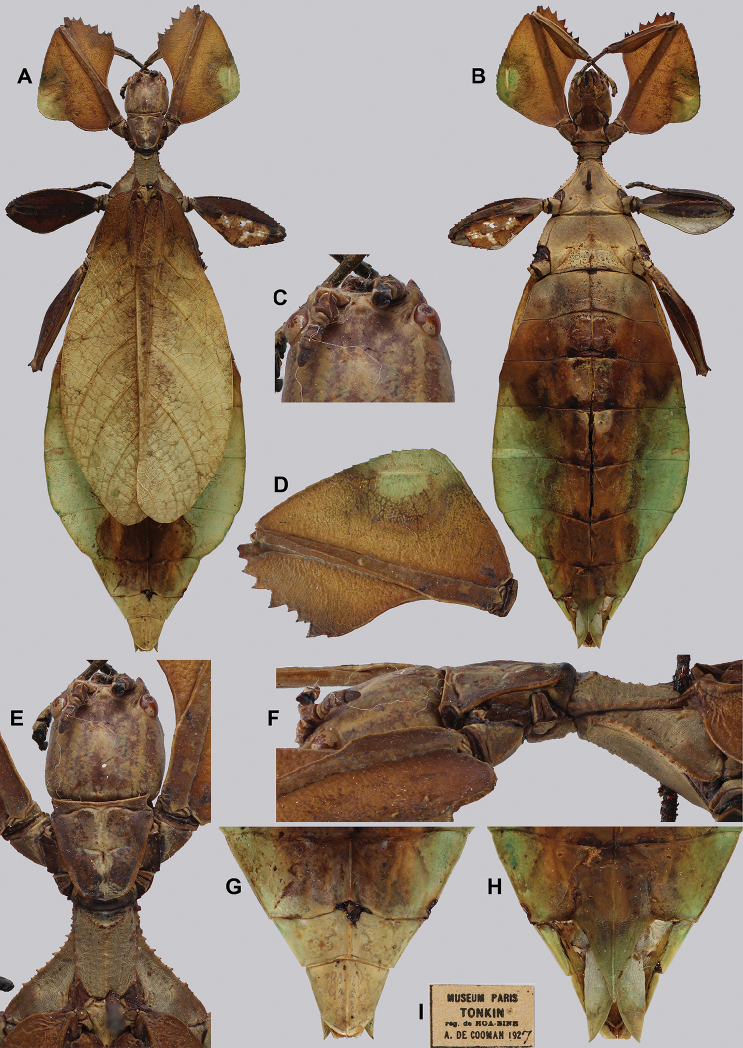
Presumed *Cryptophylliumrarum* comb. nov. a historic female from Hoa Binh, Vietnam, loaned to the RBINS from the MNHN collection, photographs by Jérôme Constant (RBINS) **A** habitus, dorsal **B** habitus, ventral **C** details of antennae and anterior of head capsule **D** profemoral lobe, dorsal **E** details of the head and thorax, dorsal **F** details of the head–thorax, lateral **G** terminalia, dorsal **H** genitalia, ventral **I** data label.

Figures 5I , 59 , 60 , 61 , 62

###### Material examined.

(5 ♀♀, 7 ♂♂): 1 ♀: “China: Guangxi Province, Nanning city, Shuangding town （双定镇）N22°59’41.39” E108°6’53.59”, VI-2016, Xiao-Yu Zhu. Molecular sample: DZW08” (Coll ZD); 1 ♀: “China, Guangxi, Liuzhou, Dayaoshan Mts, IX.2019” (IMQC); 1 ♀: “China, Guangxi, Jinxiu County, Dayao Mountain, July-Oct 2020” (Coll SLT); 1 ♀: “China, Guangxi, Jinxiu County, Dayao Mountain, Oct 2020” (Coll SLT); 1 ♀: “Museum Paris, Tonkin, reg. de Hoa-Binh, A. De Cooman 1927” (MNHN); 1 ♂: “Kon Tum Province, Ngoc Linh Mt. 1,700 m. elv. May 2015” (Coll RC 16-116); 1 ♂: “Vietnam: Da Nang Prov., Ba Na Mt.: May,2015 1,450 m.” (Coll RC 16-115); 1 ♂ “Da Nang Province, Ba Na Mt. 1,450 m. elv. May 2015” (Coll RC 16-119); 1 ♂: “Coll. I.R.Sc.N.B., Vietnam, Ninh Binh prov., Cuc Phuong Nat. Park, 20°20’53”N 105°35’52”E, 31.vii-3.viii.2016, GTI Project, Leg. J. Constant & J. Bresseel, I.G.: 33.282, RBINS-PHYLLIUM DNA sample 0004” [vomer dissected] (RBINS); 1 ♂: “Coll. I.R.Sc.N.B., Vietnam, Ninh Binh prov., Cuc Phuong Nat. Park, 20°20’53”N 105°35’52”E, 2-8.vii.2019, Leg. J. Constant, I.G.: 34.032” (RBINS); 1 ♂: “Coll. I.R.Sc.N.B., Vietnam, Tay Yen Tu Nat. Res., 21°11’10’’N 106°43’25”E, 7-11.vii.2013, night collecting, Leg J. Constant & J. Bresseel, I.G. 32.454” (VNMN); 1 ♂: “Coll. I.R.Sc.N.B., Vietnam, Tay Yen Tu Nat. Res., 21°11’10’’N 106°43’25”E, 7-11.vii.2013, night collecting, Leg J. Constant & J. Bresseel, I.G. 32.454, RBINS-PHYLLIUM DNA sample 0005” (RBINS).

###### Remarks.

The holotype female *Cryptophylliumrarum* comb. nov. is from Hexian, Baise prefecture level division, Guangxi Province, we were able to obtain a tissue sample from a female specimen from Guangxi Province from the adjoining division of Nanning City (Fig. 59C). With the species until now only known from a female, we were excited to uncover that large unknown males from Vietnam were molecularly in close relation to the female, allowing this species to be illustrated from both sexes (Fig. 60). During a joint GTI expedition, two males in Tay Yen Tu Nature Reserve and one male from Cuc Phuong N.P. were collected at night using a light trap. They were found while checking the surroundings of the light trap, having been attracted to the light but not landing on the white collection sheet. Additionally, a second male in Cuc Phuong N.P. was collected when it was attracted by the streetlights. Interestingly, inclusion of molecular data from six males from throughout north and central Vietnam did not reveal clearly delineated clades (which could represent distinct species) or a tightly formed clade with little genetic diversity (indicating a close kinship/homogenizing gene flow throughout this wide range), but instead somewhat revealed a nestedness of our samples from north to south with significant molecular distances between most samples (Fig. 4). Similar molecular distances were revealed between the geographically close *Cryptophylliumbollensi* sp. nov., *Cryptophylliumnuichuaense* sp. nov., and *Cryptophylliumphami* sp. nov. but our set of *Cryptophylliumrarum* comb. nov. samples come from a geographically wide range of several hundred kilometers. This trio of molecularly distinct and morphologically discernable species from within a limited geographic area contrasts greatly with our sampling of *Cryptophylliumrarum* comb. nov. males from a wide geographic area but no morphological feature was found to allow differentiation of these notably molecularly distinct samples. Due to the extreme geographic distance likely allowing a certain degree of intraspecific molecular variation and our lack of continuous samples of *Cryptophylliumrarum* comb. nov. from throughout the full range, we at this time treat this clade as a singular species but expect that future analysis may reveal it to be several once the larger geographic gaps in our sampling are filled.

###### Differentiation.

Females can be differentiated by the unique combination of exterior profemoral lobes which are right angled and distinctly serrate, alae which are long (reaching abdominal segment VI), and an abdomen which is distinctly tapering from the sixth segment to the tip of the abdomen (not parallel-sided or with a lobe). Species which are morphologically similar are *Cryptophylliumathanysus* comb. nov. (which has right angled exterior lobes and a spade-shaped abdomen) and *Cryptophylliumicarus* sp. nov. which as the slender form female can have a similar abdominal shape (Fig. 34A). *Cryptophylliumathanysus* comb. nov. can immediately be differentiated however as this species has prominent exterior lobes on the meso- and metatibiae whereas *Cryptophylliumrarum* comb. nov. has simple tibiae, lacking lobes. *Cryptophylliumicarus* sp. nov. can be differentiated by the profemoral exterior lobes which are notably more obtuse angled, not right angled, and the lack of well-developed alae which are small, only reaching the second abdominal segment (Fig. 33C).

Males are morphologically similar to *Cryptophylliumbankoi* sp. nov. due to their similarly shaped lobes on all legs, similar wing lengths, shape and spination of the thorax, and general abdominal shape. These two species occur sympatrically in central Vietnam and therefore we were only able to confidently differentiate them through molecular analyses which allowed us to observe the morphological variation in these species to more confidently separate them morphologically. The only two features which we consistently saw between these species were of the exterior profemoral lobe and the abdominal shape. *Cryptophylliumrarum* comb. nov. has a slightly more ovoid abdomen (*Cryptophylliumbankoi* sp. nov. is slightly more tapered on the abdominal segments VI and VII) and the profemoral exterior lobe on *Cryptophylliumrarum* comb. nov. has 6–8 distinct serrate teeth (Fig. 61E) vs. *Cryptophylliumbankoi* sp. nov. which at most have one to four small minor teeth (Figs 14A, 15C), never a full set of distinctly serrate margins.

###### Distribution.

China, Guangxi Province (recorded from Hexian, Shuangding town, and Dayaoshan Mountains) and distributed south through Vietnam. At present we have records> for five Vietnamese provinces: Vinh Phuc, Quang Ninh, Ninh Binh, Da Nang, and Kon Tum Provinces.

##### Cryptophyllium tibetense

Taxon classificationAnimalia

(Liu, 1993) comb. nov.

A26EBE1E-FDCC-5CDB-863F-9069FF5C0F01

**Figure 63.**
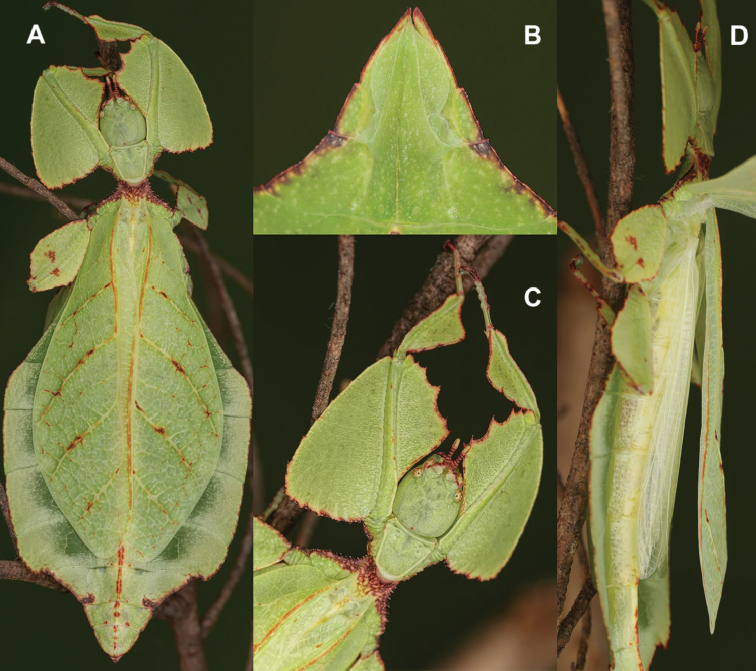
*Cryptophylliumtibetense* comb. nov. live female bred and photographed by Bruno Kneubühler (Switzerland) **A** dorsal, habitus **B** ventral genitalia, note the subgenital plate which reaches all the way to the apex of the abdomen **C** dorsal head, thorax, and front legs **D** lateral view with tegmina raised to expose the alae length.

**Figure 64.**
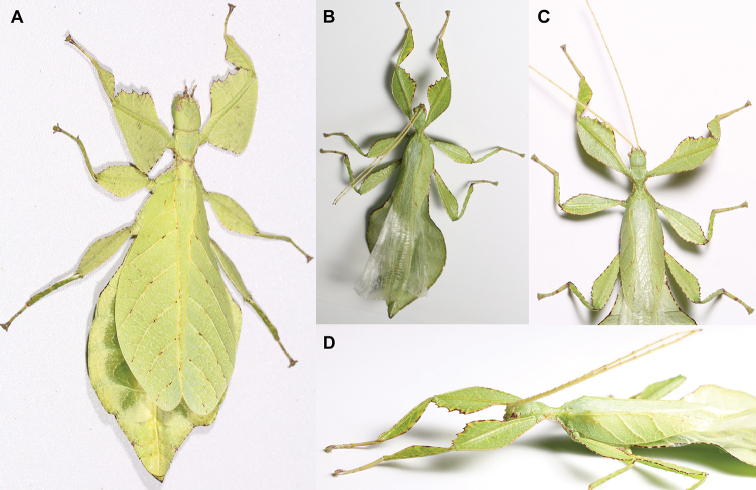
*Cryptophylliumtibetense* comb. nov. live pair from Tibet and bred and photographed by Zhiwei Dong **A** female, habitus, dorsal **B** male, habitus, dorsal **C** male, details of the antennae, front legs, head, thorax, and tegmina, dorsal **D** male, details of the antennae, front legs, head, thorax, and tegmina, lateral view.

**Figure 65.**
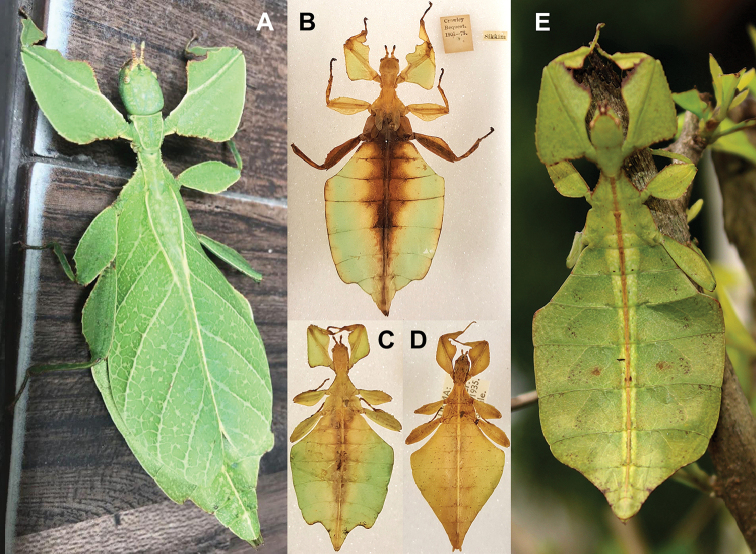
Additional *Cryptophylliumtibetense* comb. nov. distribution records> **A** live adult female from Kalimpong, West Bengal, India, observed and photographed by Vandana Wadwa Sood (West Bengal, India) in September 2019 **B** female nymph, NHMUK from Sikkim, India, photographs by RTC **C** female nymph, NHMUK: “Burma: Mishmi Hills. Delei River. 1,700ft. 28.i.1935, M.Steele.”, photographs by RTC **D** male nymph, NHMUK: “Burma: Mishmi Hills. Lohit River. 22.iii.1935, M.Steele.”, photographs by RTC **E** female nymph observed and photographed in Digboi, Assam by Rajib Rudra Tariang (India).

**Figure 66.**
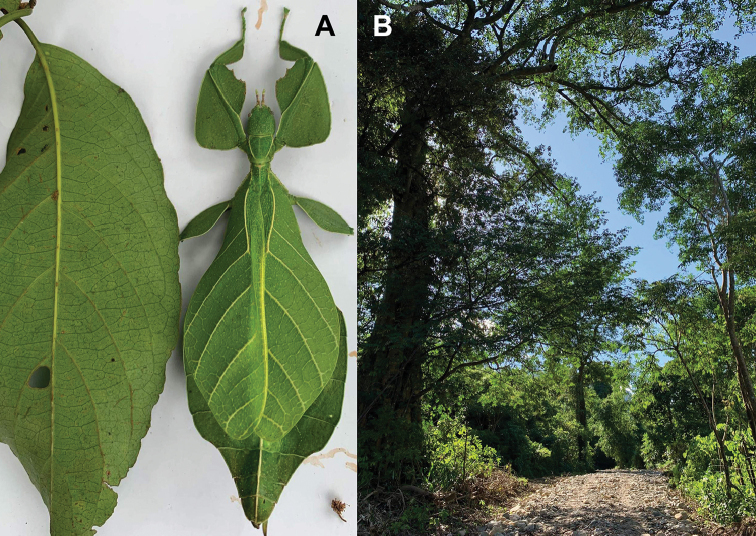
**A***Cryptophylliumtibetense* comb. nov. from Pasighat in the East Siang district, observed and photographed by Oken Tayeng (India) next to a visually similar leaf from the area the female was found **B** habitat near where the female was found.

Figures 8C , 8D , 9D , 63 , 64 , 65 , 66

###### Material examined.

(7 ♀♀, 4 ♂♂, 10 eggs): 1 ♂: “Tibet, Nyingchi Area, De’Ergong Village, Motutown 2020” (Coll SLT); 1 ♂: “China, Tibet, Motuo, Beibeng. 2019. 6-8.” (Coll RC 20-001); 1 ♀ nymph: “Crowley Bequest. 1901-78. Sikkim” (NHMUK); 1 ♀ nymph: “Arunachal Pradesh from the Mishmi Hills. Delei River. 1,700ft. 28.i.1935, M.Steele.” (NHMUK); 1 ♂ nymph: “Arunachal Pradesh from the Mishmi Hills. Lohit River. 22.iii.1935, M.Steele.” (NHMUK); 1 ♂, 1 ♀: “bred from material collected in: Tibet China: Beibeng Township,Medog County, IX-2016, Jin Chen.” (Coll ZD); 10 eggs: “China: Tibet: Medong Region: Bred by Bruno Kneubühler,2018” (Coll RC 18-396–18-405).

***Photographic records***:1 ♀: “Kalimpong, West Bengal, India, September, 2019, photographed by Vandana Wadwa Sood (West Bengal, India)”; 1 ♀nymph: “Digboi, Assam 786171, India, iNaturalist user @rajib, by Rajib Rudra Tariang” (https://www.inaturalist.org/observations/61945900); 1 ♀: “Samthar, iNaturalist user @ripbumlepcha” (https://www.inaturalist.org/observations/35911522); 1 ♀: “Pasighat in the East Siang district, observed and photographed by Oken Tayeng”

###### Remarks.

*Cryptophylliumtibetense* comb. nov. is the highest latitude phylliid species known at present, and interestingly it is also one of the largest known species of *Cryptophyllium* gen. nov. (although the unknown female for *Cryptophylliumanimatum* sp. nov. may be larger as the holotype male is 89.4 mm long suggesting a significantly sized female). The region where *Cryptophylliumtibetense* comb. nov. is found is more temperate (Fig. 66B) than the tropics where phylliids are more typically known, and we hope that future ecological studies will reveal more about their generation time and general ecology in this region. This species was described from a female holotype, and it has entered the phasmid breeding community, however, as a parthenogenetic culture (Fig. 63), therefore records> of males are absent from culture and rarely collected. We have examined few male specimens, but all have matched morphologically to the male we illustrate from Beibengxiang, Mêdog County, Nyingchi, Tibet (Fig. 64B). The first image we are aware of for this species is a beautiful color image from Inglis (1930) which was meant to be illustrating ‘*Phylliumscythe*’ taken by Mr. T. A. Baldry (a mammologist active in the Darjeeling area, West Bengal in the 1920’s and 30’s). Inglis (1930) although confused as to which species he was illustrating (although to be fair we are aware of Phyllium (Pulchriphyllium) bioculatum scythe Gray, 1843 is also known from this area so he may have at one time seen the species he was intending to mention), he did note that leaf insects found in this area (although rare) were found feeding on *Castanopsishystrix*, the Common Chestnut. This is the first record of phylliids feeding on *Castanopsis*, the second being of *Cryptophylliumoyae* comb. nov. from northern Laos (Cumming and Le Tirant 2020).

###### Differentiation.

Females are morphologically similar to *Cryptophylliumdrunganum* comb. nov. and *Cryptophylliumliyananae* sp. nov. based on the general abdominal and femoral lobe shapes, the shape and spination of the thorax (Fig. 63C), long alae at least extending abdominal segment VI (Fig. 63D) and the presence of small exterior lobes on the tibiae (Fig. 63C). *Cryptophylliumtibetense* comb. nov. can be differentiated from both however by the shape of the subgenital plate which is long and projecting beyond the tip of the abdomen in *Cryptophylliumtibetense* comb. nov. (Fig. 63B) and shorter, only ca. ½ the length of abdominal segment X in the other two species (Fig. 47E).

Male *Cryptophylliumtibetense* comb. nov. are morphologically similar to *Cryptophylliumrarum* comb. nov. and *Cryptophylliumbankoi* sp. nov. due to the thorax shape and fine granular mesopleura margins (Fig. 64C), tegmina lengths (Fig. 64D), femoral lobe shapes (Fig. 64C), and general abdominal shape. Both of these species can be differentiated from *Cryptophylliumtibetense* comb. nov. by the absence of small but distinct exterior, anteriorly situated protibial and metatibial lobes in the former two species.

###### Distribution.

The type locality for *Cryptophylliumtibetense* comb. nov. stated by Liu (1993) is Tibet (Xizang Autonomous Region), Mêdog County (Motuo). There do appear to be additional distribution records> for this high elevation species however. Unfortunately, we have only been presented with photographs of individuals or antique subadults/nymphs, and no fresh adult specimens to examine or include in our molecular phylogeny at this time. Therefore, these additional distribution records> are only presumed to be *Cryptophylliumtibetense* comb. nov. due to their morphological similarity to bred nymphs of this species and are here presented to give as thorough a view into the *Cryptophyllium* gen. nov. distribution as possible. From Southeast Tibet, Tenga Valley, we have been presented with an image of an adult male which looks to be the right size and has morphological features which suggest it could represent the *Cryptophylliumtibetense* comb. nov. male. Additionally, there is a pair of nymphs in the NHMUK from “Arunachal Pradesh from the Mishmi Hills” from 1935, a female from Anjaw District “Delei River. 1,700ft. 28.i.1935, M.Steele.” (Fig. 65C) and a male from the Lohit District “Lohit River. 22.iii.1935, M.Steele.” (Fig. 65D). Recently we also were sent photos of a female from Pasighat in the East Siang district, which matches well with the *Cryptophylliumtibetense* comb. nov. morphology (Fig. 66A).

From Nepal we are aware of two records>, both female subadults, one found in Gandaki Pradesh, Tanahun District and the other found in Province No. 1, Ilam. Both of these subadult females appear to have *Cryptophylliumtibetense* comb. nov. shaped abdomens, profemoral lobes, and importantly small exterior tibial lobes which help to characterize *Cryptophylliumtibetense* comb. nov. females.

From India we have located a nymph from within the NHMUK collection from Sikkim (Fig. 65B), and we have been lucky enough to be presented with photographs of an adult female from West Bengal, Kalimpong (Fig. 65A) both of which have *Cryptophylliumtibetense* comb. nov. like features. From the state of Assam we have been sent an image of a female nymph from the town of Digboi which can clearly be seen as having distinct but small exterior tibial lobes (Fig. 65E).

Interestingly, we have yet to be presented with records> from Bhutan, but as it lies between areas where *Cryptophyllium* gen. nov. species have been confirmed we expect that there likely is at least one species present, just not yet officially recorded.

##### Cryptophyllium wennae

Taxon classificationAnimalia

gen. et sp. nov.

5B088CA6-B89C-59CD-808B-3FABF59F3EC1

http://zoobank.org/84EC5BCA-460D-4DD6-A4CC-0A974879A2C4

Figure 67

###### Material examined.

***Holotype*** ♀: “CHINA: Yunnan Province, Chashan Park, Simao District, Puer City, VI-2017, leg. Xiao-Yu Zhu”. deposited in the Kunming Institute of Zoology (KIZ), Yunnan, China.

###### Remarks.

This species was unveiled through our molecular analysis to be distinct from the sympatrically occurring *Cryptophylliumyunnanense* comb. nov. but unfortunately, only this singular holotype is known to us at this time. Hopefully future collecting in this region will reveal additional specimens and the extent of their morphological variation.

###### Differentiation.

**Figure 67.**
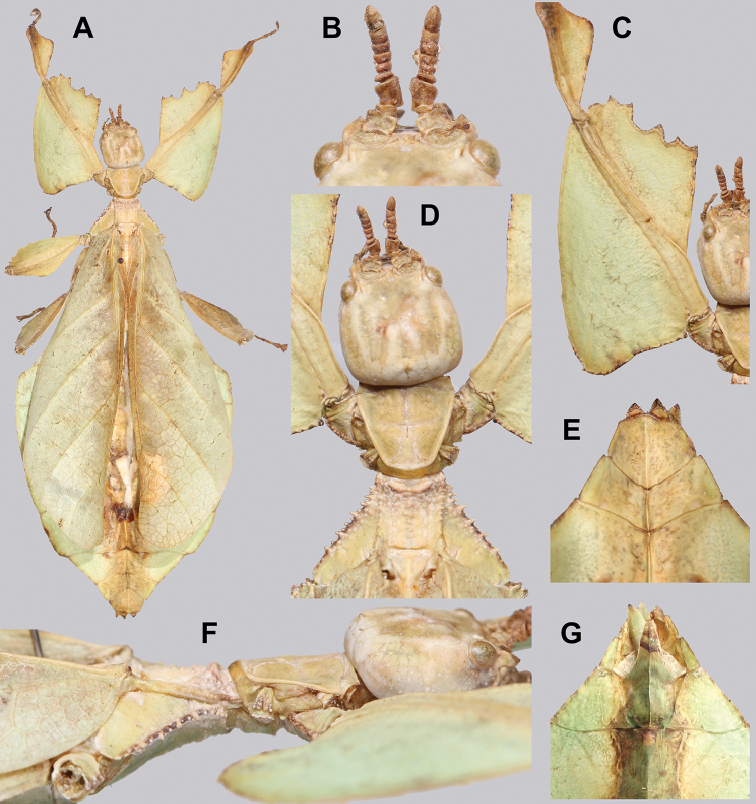
Holotype female *Cryptophylliumwennae* gen. et sp. nov., photographs by Zhiwei Dong (KIZ) **A** habitus, dorsal **B** details of antennae, dorsal **C** details of profemoral lobes **D** details of the head and thorax, dorsal **E** terminalia, dorsal **F** details of the head and thorax, lateral **G** genitalia, ventral.

Females are morphologically most similar to *Cryptophylliumoyae* comb. nov. and *Cryptophylliumtibetense* comb. nov. due to the presence of small exterior tibial lobes, similar shaped exterior profemoral lobes (Fig. 67C), similar thorax shape and spination (Fig. 67D), and similar abdominal shape (Fig. 67A). Due to morphological variation noted within these two species and the damaged alae and tip of the subgenital plate in the holotype *Cryptophylliumwennae* sp. nov. (both morphological features frequently helpful for differentiation), no consistent feature has yet been found to differentiate *Cryptophylliumwennae* sp. nov. from them except for size as the holotype *Cryptophylliumwennae* sp. nov. is only 78.5 mm long vs. *Cryptophylliumtibetense* comb. nov. which is noted as 106.5 mm long (Liu 1993) or *Cryptophylliumoyae* comb. nov. which are 82.1 to 94.2 mm long (Cumming and Le Tirant 2020). Hopefully once the egg and male morphology is known more reliable morphological features can be found to differentiate this species more easily.

Males are presently unknown.

###### Distribution.

At present only known from the type locality of Puer City in Yunnan Province, China.

###### Description.

**Female. *Coloration.*** Based upon the singular dried holotype specimen (Fig. 67). Living individuals are always a more vibrant green and this specimen appears to have been fully green, without patches of variable brown. The dried holotype female is pale green throughout with discoloration of tan along the shafts of the legs and head, thorax, and abdomen likely due to the drying process.

***Morphology.****Head.* Head capsule slightly longer than wide, vertex relatively smooth with only slight granulation throughout the surface, all relatively well-spaced (Fig. 67D). The posteromedial tubercle is broader and taller than any other nodes on the head capsule (Fig. 67F). Frontal convexity broad and about as long as the first antennomere, with a slightly lumpy surface, and with several setae present throughout (Fig. 67D). Compound eyes slightly protruding from the head capsule, not notably large, taking up slightly < ¼ of the length of the lateral head capsule margins (Fig. 67D). Ocelli absent. Antennal fields wider than the first antennomere (Fig. 67D). *Antennae.* Antennae consisting of nine segments, with the terminal segment about the same length as the preceding two segments’ lengths combined (Fig. 67B). Antennomeres I–VIII sparsely marked with small transparent setae, the terminal antennomere has darker, shorter, and denser setae than the other segments (Fig. 67B). *Thorax.* Pronotum with gently concave anterior margin and straight lateral margins, which converge to a convex posterior margin that is half the width of the anterior margin (Fig. 67D). The pronotum surface and moderately formed pronotum rims are smooth, lacking significant granulation, with only a prominent pit in the center, and slight furrows anterior, posterior, and lateral to the pit (Fig. 67D). Prosternum with moderate granulation, mesosternum anterior margin and the anterior half of the sagittal plane with moderate granulation. Metasternum relatively smooth, lacking notable nodes. Prescutum as long as wide, lateral rims with nine or ten irregularly shaped but not large tubercles with those on the anterior slightly larger than those on the posterior (Fig. 67D). Prescutum anterior rim prominent but not strongly protruding, surface marked throughout with irregular granulation, no prominent singular sagittal spine present (Fig. 67D). Prescutum surface covered irregularly by moderate nodes, with slightly larger nodes along the sagittal plane (Fig. 67D). Mesopleura beginning on the anterior margin of the prescutum and evenly diverging; lateral margin with five larger tubercles, and eight or nine smaller tubercles interspersed unevenly throughout with some tubercles touching side by side or with a slight gap between them (Fig. 67D). Face of the mesopleura is mostly smooth but with lateral margins that have a slightly granular surface (Fig. 67D). *Wings.* Tegmina long, reaching ½ through abdominal segment VII. The subcosta (Sc) is the first vein in the forewing and runs parallel with the tegmina lateral margin for the first ½ of the vein, then bends gently and runs to the lateral margin of the wing where it terminates ca. ¼ of the way through the length. The radius (R) spans the central portion of the forewing with two subparallel branched veins; radius 1 (R1) terminates ca. ⅓ of the way through the wing length, and the radial sector (Rs) terminates ca. ⅔ of the way through the wing length. There is a weak continuation of the radius following the prominent Rs branching which continues on as a short and thinner R–M crossvein that does not solidly connect the two veins as it reaches the media. The media (M) is simply bifurcate with both the media anterior (MA) and media posterior (MP) terminating near the posterior ¼ of the wing. The cubitus (Cu) is also bifurcate, branching near the posterior ⅕ of the wing into the cubitus anterior (CuA) and cubitus posterior (CuP) which both terminate at or very near the wing posterior apex. The first anal vein (1A) is simple and fuses with the cubitus early on, near the branching distance of the R1 from R. Alae in the holotype are unfortunately deformed, so we do not yet know what length alae this species has. *Abdomen.* Abdominal segments II through the anterior ⅓ of IV diverging slowly at first and then more strongly towards the posterior, with the posterior ⅔ of segment IV the widest segment. Segments V–VI are subparallel, and segment VII converges slightly more prominently and ends in a slight lobe. Segments VIII–X are notably narrower than the previous segments and converge uniformly to the rounded apex. *Genitalia.* Subgenital plate starts at the anterior margin of segment VIII, is long and narrow reaching significantly onto the terminal segment (Fig. 67G). Gonapophyses VIII are long and moderately broad, exceeding the apex of abdominal segment X, gonapophyses IX are smaller and slender, hidden below the gonapophyses VIII (Fig. 67G). Cerci flat, not strongly cupped, with a heavily granular surface and few detectable setae (Fig. 67E). *Legs.* Profemoral exterior lobes notably wider than the interior lobe with an acute angle due to a slight recurve of the lobe (Fig. 67C). Proximal edge of the profemoral exterior lobe slightly granular, and the bend and distal edge is marked by six or seven small serrate teeth (Fig. 67C). Profemoral interior lobe ca. 3× as wide as the greatest width of the profemoral shaft, with an obtuse angle, and marked with five prominent teeth arranged in a two-one-two pattern with large looping gaps between the teeth (Fig. 67C). Mesofemoral exterior lobe arcs from end to end in a slightly bent lobe weighted on the distal half and marked with two serrate teeth on the distal half only. Interior and exterior lobes are of a similar width. Mesofemoral interior lobe arcs smoothly end to end, is marked with four or five serrate teeth only on the distal half of the arc, and is about as wide as the mesofemoral shaft. Metafemoral interior lobe arcs end to end, but is wider on the distal half, and has four or five serrate teeth on the distal half of the lobe only. Metafemoral exterior lobe is thin and smooth, hugging the metafemoral shaft. Protibiae interior lobe spans the entire length of the protibiae and is at least 2× as wide as the protibial shaft. The lobe is distinctly triangular with the broadest point distal to the midline. Pro-, meso-, and meta- tibiae with small anteriorly situated exterior lobes, meso-, and meta- tibiae lack interior lobes.

***Measurements of holotype female* [mm].** Length of body (including cerci and head, excluding antennae) 78.5, length/width of head 7.9/7.5, antennae 4.6, pronotum 5.5, mesonotum 6.2, length of tegmina 54.5, length of alae (unknown, deformed in the holotype), greatest width of abdomen 31.8, profemora 17.5, mesofemora 13.0, metafemora 14.2, protibiae 9.7, mesotibiae 9.0, metatibiae 12.5.

###### Etymology.

Patronym. Named in honor of Zhiwei Dong’s wife, Ms. Wen-Na Chen, for her support and love over the years.

##### Cryptophyllium westwoodii

Taxon classificationAnimalia

(Wood-Mason, 1875) comb. nov.

602EFA0F-6D16-5D66-9074-356F7C502A98

Figures 5A , 6G , 6H , 8A , 8B , 9F , 68 , 69 , 70 , 71 , 72

###### Material examined.

***Neotype*** ♂: “THAILAND: Chiang Mai Province: October 2010. Coll RC 16-148”. Deposited within the Montreal Insectarium (IMQC). Molecular sample 16-148 within this study.

###### Additional material examined.

(15 ♀♀, 21 ♂♂, 4 eggs): 3 ♀♀: “Thailand: Chiang Mai, July 2017.” (Coll RC 18-145, 18-146, 18-147); 1 ♀: “Chiang Mai, Fang: February, 2011” (Coll RC 16-211); 1 ♀: “Thailand, Fang, II-2011” (Coll RC 16-212); 1 ♀: “Lamphun Province, Maetha: September, 2011” (Coll RC 16-080); 1 ♀: “Thailand, Lamphun Province, 2009 November” (Coll RC 16-078); 1 ♀: “Northern Thailand, Chiang Mai Province, 2010, October” (Coll RC 16-079); 1 ♀: “Laos: Luang Prabang Province, Kiew Mak Nao Village, 900m.: June, 2014” (Coll RC 16-077); 1 ♂: “Thailand: Chiangmai, Doi Pui, 25 May 1985” (Coll RC 16-082); 1 ♂: “Thailand: Lampon, Mae Tha, 09/2011” (Coll RC 16-083); 1 ♂: “Thailand: Chiangmai, Doi Pui, 19 May 1985” (Coll RC 16-214); 1 ♂: “Thailand: Chiangmai, Doi Pui, 28 May 1985” (Coll RC 16-215); 1 ♂: “Thailand: Chiangmai, Doi Pui, 24 May 1985” (Coll RC 16-216); 1 ♂: “Thailand: Chiangmai, Doi Pui, 25 May 1985” (Coll RC 16-217); 1 ♂: “Thailand: Lampang, May 2001” (Coll RC 16-218); 1 ♂: “North Laos: Kiew Mak Nao, VII.2015, 900m. S. Collard leg” (Coll RC 18-030); 2 ♂♂: “Thailand: Lampoon, Mae Tha, 09-2011” (Coll RC 16-147, 16-213); 1 ♂: “Burma: 4km E. Karathuri, Top of Hill, 350 to 400m., VI. 2011., Coll. A. Banko/ Collected by beating tree in Forest” (Coll RC 18-029); 1 ♂: “Coll. I.R.Sc.N.B., Thailande (Loei), Na Haeo (bio station), 05-12.V.2001, Light trap, Leg. J. Constant & P. Grootaert” [vomer dissected] (RBINS); 2 ♂♂: “Coll. I.R.Sc.N.B., Thailande (Loei), Na Haeo (field res stat)), 15-19.V.2003, Light trap, Leg. J. Constant, K. Smets & P. Grootaert” (RBINS); 1 ♂: “Coll. I.R.Sc.N.B., Thailande (Loei), Na Haeo , light trap, 15-19.V.2003, Light trap, Leg. J. Constant & K. Smets” (RBINS); 1 ♂: “Coll. I.R.Sc.N.B., Thailande (Loei), Na Haeo, light trap, 15-19.V.2003, Light trap, Leg. J. Constant & K. Smets, RBINS-PHYLLIUM DNA sample 0002” (RBINS); 1 ♂: “Coll. I.R.Sc.N.B., Thailande (Loei), Na Haeo, forest clearing, light trap, 16.V.2003, Light trap, Leg. J. Constant & K. Smets” (RBINS); 1 ♂: “Coll. I.R.Sc.N.B., Thailand, Loei, Na Haeo, 22.V.2000, Station 20007, Leg P. Grootaert” (RBINS); 1 ♂: “Coll. I.R.Sc.N.B., Laos, Bokeo prov., Ban Muang Kan, 1-15.vi.2012, local collectors, I.G.: 32.213, RBINS-PHYLLIUM DNA sample 0012” (RBINS); 1 ♀: “Coll. I.R.Sc.N.B., Laos, Bokeo prov., Ban Muang Kan, 1-15.vi.2012, local collectors, I.G.: 32.213, RBINS-PHYLLIUM DNA sample 0011” (RBINS); 2 ♀♀: “Coll. I.R.Sc.N.B., Laos, Bokeo prov., Ban Muang Kan, 1-15.vi.2012, local collectors, I.G.: 32.213” (RBINS); 1 ♀: “Coll. I.R.Sc.N.B., Thailand, SE Chiang Mai, Salok, Wang Chin, Near Lamphang, ex breeding A. & C. Bauduin, 2015” (RBINS); 1 ♀, 1 ♂: “Coll. I.R.Sc.N.B., Ex breeding Bruno Kneubühler, 2017, Thailand, Lamphun prov., Tha Pla Duk” (RBINS); 1 ♀, 1 ♂: “Thailand, Ex Culture Kristien Rabaey (RBINS); 4 eggs: N-Thailand, Cultured F.Hennemann 1995-2001. Ex. Coll. Frank Hennemann (Germany)” (Coll RC 18-242–18-245).

###### Type material and discussion.

Unfortunately, the male/female pair of syntypes which were originally deposited within the NZSI are considered lost (Hennemann et al. 2009; Brock et al. 2020). Philip E. Bragg, well-known phasmid researcher from the United Kingdom, several years ago was able to obtain photographs of what was assumed to be the female syntype within the NZSI collection (the possible male type could not be located). However, the female labeled as such within the collection is the wrong size, does not have an original Wood-Mason collection label, and when compared with the illustration in Wood-Mason (1875) instead clearly represents a different species (a Phyllium (Pulchriphyllium) female). While it is possible that the original syntypes are still in the NZSI collection, if their original data labels were accidentally removed, or moved to other specimens, then there would be no way to positively identify either the male or the female. Besides their lost status, with our understanding of *Cryptophyllium* gen. nov. morphology now consolidated and extensively reviewed, we believe that Wood-Mason’s (1875) original syntype pair actually represented two different species, one from the mainland, and a second species from the Andaman Islands. Additionally, with the identification of a cryptic, nearly morphologically indiscernible *westwoodii*-like species from Cambodia (*Cryptophylliumkhmer* sp. nov.), a clear identification of the population which corresponds to this original name is necessary. A male specimen could not be located from the exact type locality “Pahpoon (Hpapun, Papun), 150 miles north of Moulmein (Mawlamyine, formerly Moulmein) in Salween Country (Salween River, officially Thanlwin River)” therefore we instead chose a rather morphologically average male from Chiang Mai, Thailand. Although this location is across an international border, it is only 175 kilometers away, a distance which is negligible when considering the range over which we have found *Cryptophylliumwestwoodii* comb. nov.; we have specimens genetically confirmed as the same species across a distance of over 1,000 kilometers (from northern Laos to southern Myanmar; Fig. 2). Additionally, this neotype location is the closest to the syntype locality for which we have seen specimens recorded, and Chiang Mai is a well-known breeding site for *Cryptophylliumwestwoodii* comb. nov., therefore many museums and collections around the world have ample specimens of this particular population. Also, obtaining specimens from Myanmar is exceedingly difficult and no museum specimens could be located during our review. Therefore, due to the above complex taxonomic problems involved with the original name (Article 75.1, ICZN 1999), we here establish a neotype male which matches the morphological description in Wood-Mason (1875) and the illustration of the syntype presented in Wood-Mason (1877). Consequently, due to our above reasonings and the fact that historically a lectotype was never designated from the syntype pair, the qualifying conditions for designating a neotype are satisfied in accordance with Article 75.3 of the ICZN (1999).

###### Remarks.

**Figure 68.**
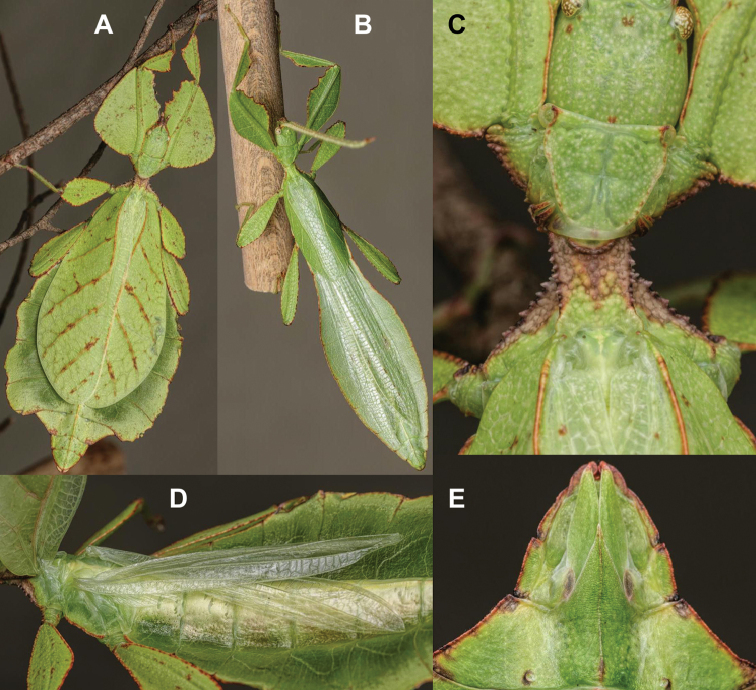
Live adult *Cryptophylliumwestwoodii* comb. nov. bred and photographed by Bruno Kneubühler (Switzerland) from stock collected in Tha Pla Duk subdistrict, Mae Tha district, Lamphun, Thailand, collected in May / June 2014 by Suttah Ek-Amnuay (Thailand) **A** female habitus **B** male habitus **C** female details of the thorax, dorsal **D** lateral view female with tegmina lifted to show long alae **E** female genitalia details, ventral.

The female and egg morphology of *Cryptophylliumwestwoodii* comb. nov. were well-described by Hennemann et al. (2009) and this species is common within the phasmid breeding community (Fig. 68) and therefore we only describe the neotype male morphology herein. This species appears to be one of the most widespread and most commonly encountered *Cryptophyllium* gen. nov. species as the range from north to south is over 1,000 km and from our review of museum collections and citizen scientist records> this is by far the most commonly observed species.

###### Differentiation.

**Figure 69.**
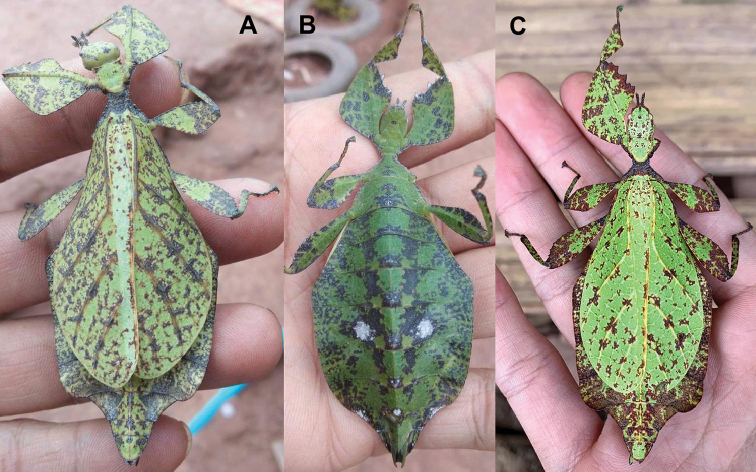
Strikingly colored live adult female *Cryptophylliumwestwoodii* comb. nov. observed in Thailand, Nan Province, Bo Kluea Tai **A** habitus, dorsal and **B** habitus, ventral, photographed by Lek Karton (Thailand) in November 2019 **C** habitus, dorsal, photographed by Tatsatorn Dharithai (Thailand) in October 2020.

**Figure 70.**
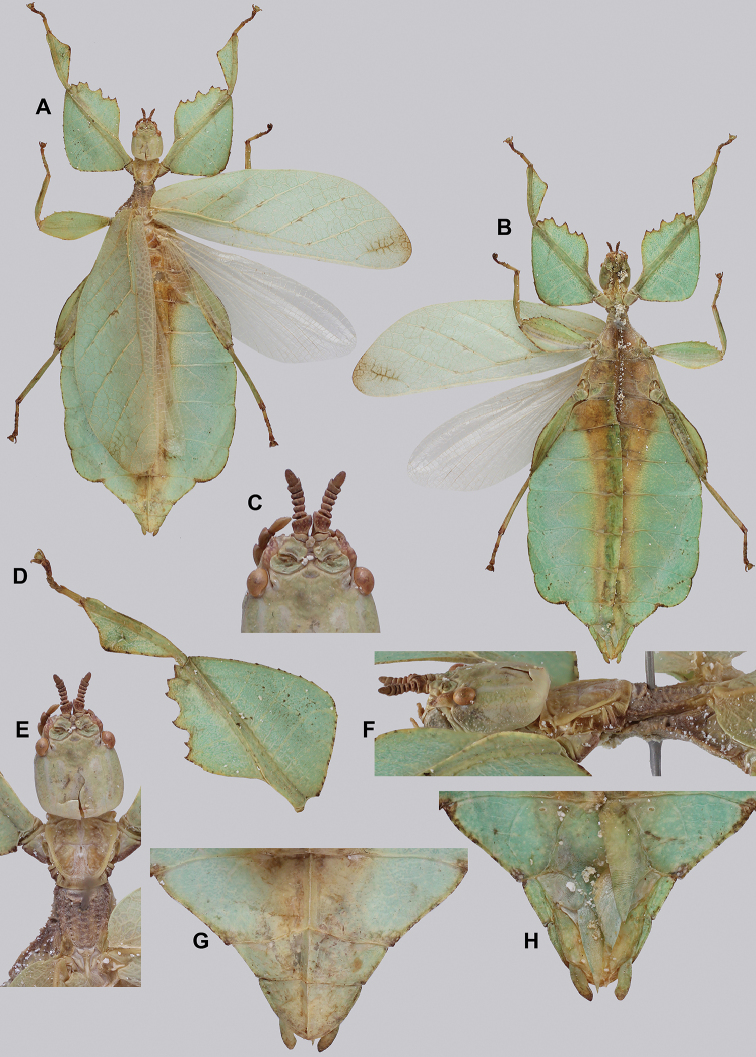
Female *Cryptophylliumwestwoodii* comb. nov. from Laos, Bokeo Province, Ban Muang Kan (RBINS), photographs by Jérôme Constant (RBINS) **A** habitus, dorsal **B** habitus, ventral **C** details of the antennae **D** pro- tibial and femoral lobes, dorsal **E** details of the antennae, head, and thorax, dorsal **F** details of the antennae, head, and thorax, lateral **G** terminalia, dorsal **H** genitalia, ventral.

Female *Cryptophylliumwestwoodii* comb. nov. are morphologically inseparable from *Cryptophylliumkhmer* sp. nov. due to the wide range of morphological forms observed within *Cryptophylliumwestwoodii* comb. nov. (both in shape and coloration; Fig. 69) which do not allow a reliable morphological feature to be identified for differentiation. When only comparing females, solely through molecular comparison can these two species be differentiated with confidence (see Fig. 4). Thankfully, males of these two species can be morphologically differentiated, discussed further below. Additionally, *Cryptophylliumwestwoodii* comb. nov. is also morphologically similar to the three southern Vietnam species: *Cryptophylliumbollensi* sp. nov., *Cryptophylliumphami* sp. nov., and *Cryptophylliumnuichuaense* sp. nov. females. All of these species share similar femoral and tibial lobe shape and serration (Fig. 70D), narrow anterior margin of the mesopleura and serration (Fig. 70E), and an abdominal shape which is boxy with a rounded lobe VII (Fig. 70A). From all of these southern Vietnam species however *Cryptophylliumwestwoodii* comb. nov. can be differentiated by the length of the alae which are long, reaching onto abdominal segment VI (Fig. 68D), vs. all these others which have shorter alae, reaching segments II or III only.

**Figure 71.**
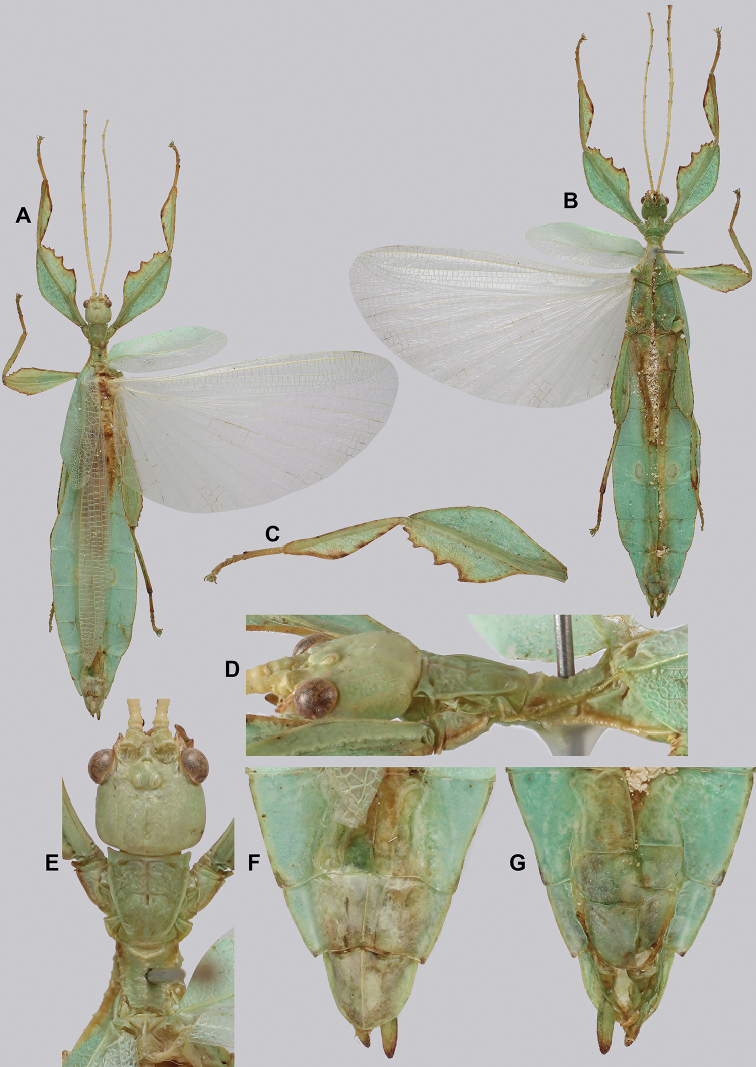
Male *Cryptophylliumwestwoodii* comb. nov. from Laos, Bokeo Province, Ban Muang Kan (RBINS), photographs by Jérôme Constant (RBINS) **A** habitus, dorsal **B** habitus, ventral **C** front leg, dorsal **D** details of the antennae, head, and thorax, lateral **E** details of the antennae, head, and thorax, dorsal **F** terminalia, dorsal **H** genitalia, ventral.

**Male.***Cryptophylliumwestwoodii* comb. nov. are morphologically similar to *Cryptophylliumathanysus* comb. nov. and *Cryptophylliumchrisangi* comb. nov. due to their similar femoral lobe shape and serration, their shorter tegmina length (only reaching abdominal segment IV), and their general abdominal shape (Fig. 71A). *Cryptophylliumathanysus* comb. nov. can immediately be differentiated by the presence of fully spanning metatibial exterior lobes, as *Cryptophylliumwestwoodii* comb. nov. lacks exterior lobes on all tibiae. *Cryptophylliumwestwoodii* comb. nov. and *Cryptophylliumchrisangi* comb. nov. are very similar in morphology, and the only consistent feature we have seen between these to differentiate them is the size, with *Cryptophylliumchrisangi* comb. nov. slightly larger (73–74 mm long; Seow-Choen 2017) and *Cryptophylliumwestwoodii* comb. nov. smaller (63–69 mm long; Hennemann et al. 2009). Despite female *Cryptophylliumwestwoodii* comb. nov. being inseparable morphologically from *Cryptophylliumkhmer* sp. nov., the males do consistently differ in the width of their abdomen, with *Cryptophylliumwestwoodii* comb. nov. having an abdominal shape that is thinly elliptical, with a maximum width only 30–34% of the abdominal length (Fig. 71A), vs. *Cryptophylliumkhmer* sp. nov. which has an abdominal shape broadly elliptical or broadly spade-shaped with a maximum width ca. 38–45% of the abdominal length (Fig. 40A).

###### Distribution.

*Cryptophylliumwestwoodii* comb. nov. has only been confirmed through genetic analysis from northern Thailand, northern Laos, and southern Myanmar. With the description of *Cryptophylliumkhmer* sp. nov. which morphologically cannot be differentiated from photographs of nymphs or females, we are unsure where these two species biogeographically are separated, but at this time we only know of *Cryptophylliumkhmer* sp. nov. from Cambodia and are unsure if *Cryptophylliumwestwoodii* comb. nov. also occurs in this country. Until additional *Cryptophylliumwestwoodii* comb. nov. samples from throughout the range are also sequenced, the true distribution must remain somewhat vague at this point (as indicated by the bi-colored symbols in our distribution map; Fig. 2).

###### Neotype male.

**Figure 72.**
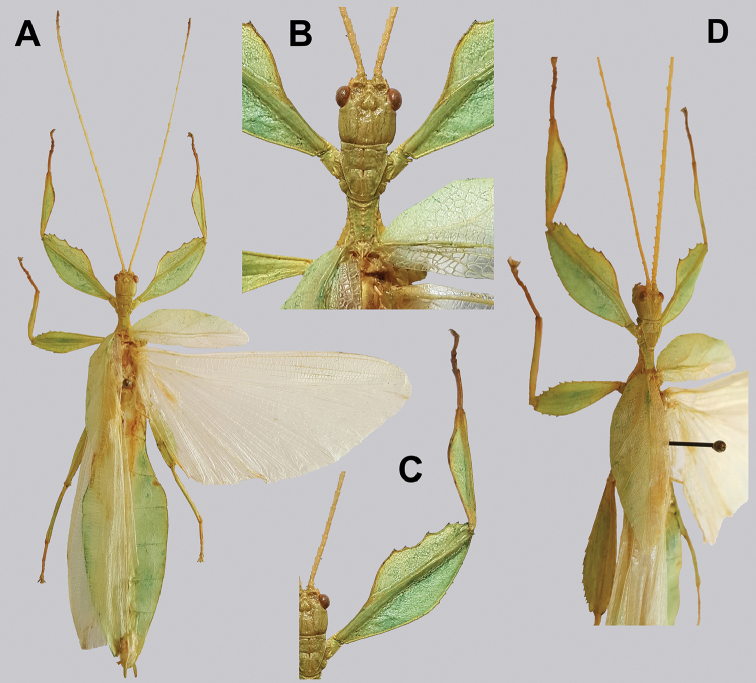
Neotype male *Cryptophylliumwestwoodii* comb. nov. from Chiang Mai Province, Thailand (Coll RC 16-148), photographed by RTC **A** habitus, dorsal **B** details of the base of the antennae, head, and thorax, dorsal **C** front leg details, dorsal **D** lateral view of the anterior portion of the specimen.

***Coloration.*** Coloration description is based upon the dried neotype specimen (Fig. 72A), living individuals are more vibrant. Overall coloration pale green with variable patches of straw yellow throughout due to the drying process (primarily around the center of the body and the antennae). Compound eyes burnt red in color and basitarsi are orange.

***Morphology.****Head.* Head capsule about as long as wide, with a vertex that is smooth except for the posteromedial tubercle which is not broad but is distinctly raised from the head capsule (Fig. 72B). Frontal convexity stout with a narrow point and marked with sparse thin setae. Compound eyes large and bulbous, taking up ca. ⅖ of the head capsule lateral margins (Fig. 72B). There are three moderately developed ocelli located between and slightly posterior to the compound eyes. Antennal fields as wide and as long as the scapus. *Antennae.* Antennae (including the scapus and pedicellus) consists of 27 segments, all segments except the scapus and pedicellus and terminal three segments are covered in dense pale setae that are as long as or longer than the antennae segment is wide. The terminal four segments are covered in dense, dark, short setae and the scapus and pedicellus are nearly completely bare. *Thorax.* Pronotum with anterior margin slightly concave and lateral margins that are nearly straight and converging to a straight posterior margin that is ½ the width of the anterior rim (Fig. 72B). Anterior and lateral margins have moderate rims, and the posterior margin lacks a rim (Fig. 72B). Face of the pronotum is marked by a distinct furrow in the center, short furrows lateral to this central sagittal furrow, and a smooth but slightly lumpy surface (Fig. 72B). Prosternum is moderately granular throughout with small nodes of even size. Mesosternum surface marked with slightly more prominent nodes on the anterior half and the posterior half has similar small nodes as those on the prosternum. Metasternum with a slightly wrinkled surface and small sparse nodes. Prescutum slightly longer than wide, with lateral margins slightly converging to the posterior (Fig. 72B). Lateral rims with eight or nine nodes giving the surface a rough textured appearance, not very large or prominent (Fig. 72B). Prescutum surface rather smooth except for along the sagittal plane which has seven or eight small nodes of about even size (Fig. 72B). Prescutum anterior margin prominent but not strongly raised above the surface, margin slightly granular and lacking a prominent central tubercle (Fig. 72D). Mesopleura rather narrow, gently diverging throughout the length, lateral margin with only slight granulation throughout, no prominent tubercles, at most three or four nodes and slight interspersed granulation throughout (Fig. 72B). Face of the mesopleura slightly wrinkled and with two distinct divots, one on the anterior ⅓ and one near the middle. *Wings*. Tegmina of moderate length, extending ½ through abdominal segment III. Tegmina wing venation: the subcosta (Sc) is the first vein, is simple, and terminates slightly < ½ through the overall tegmina length. The radius (R) spans the entire length of the tegmina with the first radius (R1) branching ca. ⅖ of the way through the wing length and terminating ca. ⅗ of the way through the wing length, followed by the branching and termination of the second radius (R2) near the distal ⅓ of the wing, and then the radial sector runs to the wing apex. The media (M) also spans the entire length of the tegmina with the first media posterior (MP1) branching off ca. ⅖ of the way through the wing length, and then the second media posterior (MP2) branching near the midline, and the media anterior (MA) runs to the wing apex. The cubitus (Cu) runs along the edge of the wing as the two media posterior veins fuse with it and as the cubitus reaches the apex it fades. The first anal (1A) vein terminates upon reaching the cubitus slightly < ⅓ of the way through the wing length. Alae well-developed in an oval fan configuration, long, reaching onto abdominal segments IX. Alae wing venation: the costa (C) is present along the entire foremargin giving stability to the wing. The subcosta (Sc) is long, spanning ca. ⅔ of the wing length and is mostly fused with the radius in the beginning but terminates when it meets the costa. The radius (R) spans the entire wing and branches ca. ⅖ of the way through into the first radius (R1) and radial sector (Rs) which run gently diverging for most of their length and then converge at the apex of the wing where they terminate near each other but not touching. The media (M) branches early, ca. ⅙ of the way through the wing into the media anterior (MA) and the media posterior (MP) which run parallel with each other throughout the wing until the distal ⅙ of the wing where the media posterior fuses with the media anterior which then run fused together to the wing apex where they terminate near the radial sector. The cubitus (Cu) runs unbranched and terminates at the wing apex. Of the anterior anal veins, the first anterior anal (1AA) fuses with the cubitus near the point where the media branches into the media anterior and media posterior and then the first anterior anal branches from the cubitus ⅔ of the way through the wing length where it uniformly diverges from the cubitus until it terminates at the wing margin. The anterior anal veins two–seven (2AA–7AA) have a common origin and run unbranched in a folding fan pattern of relatively uniform spacing to the wing margin. The posterior anal veins (1PA–6PA) share a common origin separate from the anterior anal veins and run unbranched to the wing margin with slightly thinner spacing than the anterior anal veins. *Abdomen.* Abdominal segment II slightly converging, III through the anterior half of segment IV diverging to the widest portion. The posterior of IV and the anterior half of V parallel, the remainder of segments V–X gently converging to the rounded apex. *Genitalia.* Poculum broad and ends in a straight margined apex that slightly passes the anterior margin of segment X. Cerci long and slender, with slightly > ½ their length extending from under the anal abdominal segment, relatively flat, covered in a granulose surface and numerous short setae. Vomer broad and stout with straight sides evenly converging, and a thick single apical hook which hooks upwards into the paraproct and a notable smaller hook near the base of the primary hook, situated to the left of the primary hook when viewed ventrally. *Legs.* Profemoral exterior lobe slightly wider than the interior lobe (ca. 2½× as wide as the greatest width of the profemoral shaft), smoothly arcing end to end without a distinct bend and marked with a slightly granular margin and four small teeth on the distal half (Fig. 72C). Profemoral interior lobe roundly triangular and marked with five teeth arranged in a two-one-two pattern with shallow looping gaps between them (Fig. 72C). Mesofemoral exterior lobe arcs end to end but is significantly weighted more so on the distal half which is marked with three serrate teeth and the proximal half that is rather thin, lacking dentation. Mesofemoral interior lobe is slightly thinner than the exterior lobe, is broader on the distal end and is marked with six or seven small serrate teeth on the distal end. Metafemoral exterior lobe lacks dentation and has a straight margin along the metafemoral shaft. Metafemoral interior lobe smoothly arcs end to end with nine small serrate teeth on the distal half which is wider than the proximal half (Fig. 72D). Protibiae lacking exterior lobe, interior lobe reaching end to end in a rounded triangle with the widest portion on the distal half ca. 2× the width of the protibial shaft (Fig. 72C). Meso- and metatibiae simple, lacking lobes completely.

**Measurements of neotype male [mm].** Length of body (including cerci and head, excluding antennae) 70.5, length/width of head 3.9/3.8, antennae 47.8, pronotum 3.3, mesonotum 4.1, length of tegmina 20.5, length of alae 51.8, greatest width of abdomen 14.7, profemora 15.0, mesofemora 12.7, metafemora 15.4, protibiae 11.1, mesotibiae 18.5, metatibiae 11.5.

##### Cryptophyllium yapicum

Taxon classificationAnimalia

(Cumming & Teemsma, 2018) comb. nov.

2547FE3A-5146-576C-863A-BAB416DE4B82

Figures 73 , 74

###### Material examined.

**Figure 73.**
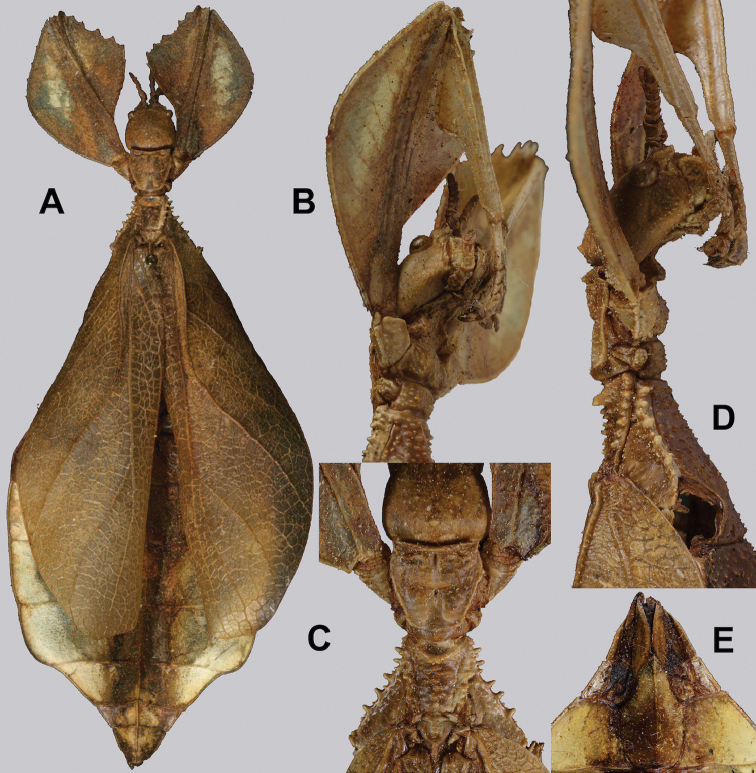
Holotype female *Cryptophylliumyapicum* comb. nov. photographed by RTC **A** habitus, dorsal **B** ventrolateral view of the protibiae showing the unique lobe shape **C** details of the thorax and head, dorsal **D** lateral view of the thorax–head **E** terminalia, ventral.

**Figure 74.**
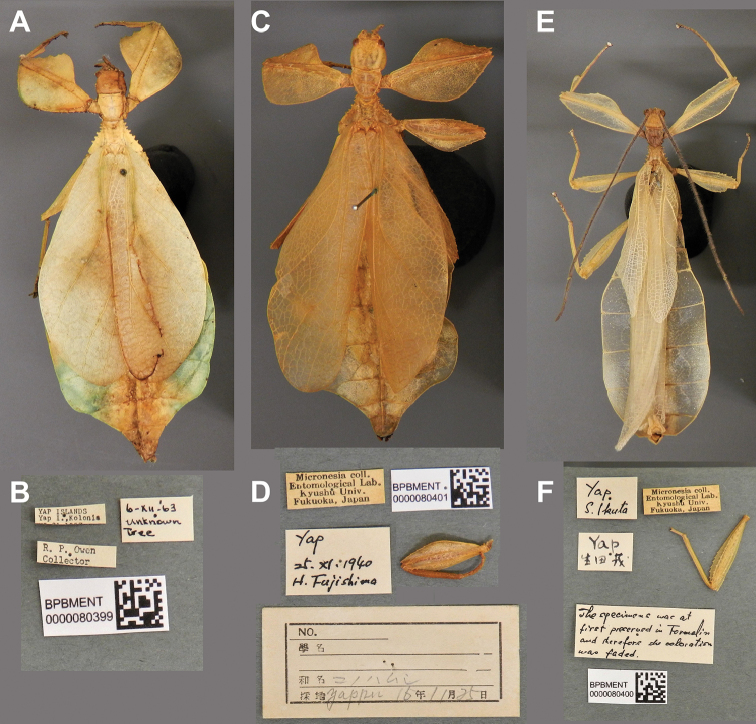
Three additional non-type specimens of *Cryptophylliumyapicum* comb. nov., photographs by Miho Maeda, Jerilynn Chun, and James Boone, 2020 (BPBM). Corresponding data labels below the specimen **A** female habitus, dorsal **B** associated data labels **C** female habitus, dorsal **D** associated data labels **E** male habitus, dorsal **F** associated data labels.

(3 ♀♀, 1 ♂): At present only the holotype within the CAS collection, and three additional specimens within the BPBM collection are known to us. 1 ♀: “M.R.Lundgren, Collector. Kaday, Yap, Xi.10.1980. CASTYPE #19438, Holotype” (Fig. 73); 1 ♀: “Yap Islands, Yap I., Kolonia. R. P. Owen, Collector. 6-xii-’63, unknown tree. BPBMENT, 0000080399” (Fig. 74A); 1 ♀: “Yap, 25.XI.1940, H. Fujishima. Micronesia coll., Entomology Lab., Kyushu univ., Fukuoka, Japan. BPBMENT, 0000080401.” (Fig. 74C); 1 ♂: “Yap, S. Ikuta. Micronesia coll., Entomology Lab., Kyushu univ., Fukuoka, Japan. Yap. The specimens was at, first preserved in Formalin, and therefore the coloration, was faded. BPBMENT, 0000080400.” (Fig. 74E).

###### Remarks.

This species is apparently rather elusive as it is one of the largest insects in Micronesia but also one of the rarest (as it is only known from four museum specimens). This poorly known species is only represented by old/degraded specimens collected between 1940 and 1980 and therefore it is one of only two species excluded from our molecular analysis (the other being *Cryptophylliumathanysus* comb. nov. only known from antique specimens and recent photographs). Hopefully further collection efforts in Micronesia will locate this species again so it can be observed live, and a tissue sample can be recovered in order to help place the unique species phylogenetically.

###### Differentiation.

Both sexes have the unique feature of having the interior protibial lobe reduced on the distal half with a majority of the lobe and the widest point on the proximal half only (Fig. 73B) which allows differentiation from all congenerics.

Females with their boxy rounded abdomen and rounded but broad exterior profemoral lobes are visually similar to *Cryptophylliumbollensi* sp. nov. and *Cryptophylliumwestwoodii* comb. nov. moderate form females. From both species *Cryptophylliumyapicum* comb. nov. can be differentiated by the unique protibial interior lobe as the other species have fully spanning lobes with the broadest point on the distal half of the protibiae. Additionally, these species do not have mesopleura with large tubercles (Fig. 73C), instead they have granular surfaces.

Males are quite unique and do not readily resemble congenerics. With an abdomen that has abdominal segments V and VI parallel-sided, their abdomen is boxy and unlike the typical *Cryptophyllium* gen. nov. abdomen (which are generally ovoid or spade-shaped). The closest that other *Cryptophyllium* gen. nov. species get to a boxy abdomen like this is in *Cryptophylliumcelebicum* comb. nov. but in this species typically only segment V is parallel-sided and VI is generally bent and converging on the posterior half. These two species can be differentiated by their significantly differing tegmina lengths as *Cryptophylliumcelebicum* comb. nov. tegmina only reach to the anterior margin of abdominal segment III but *Cryptophylliumyapicum* comb. nov. have the longest tegmina recorded in this genus. *Cryptophylliumyapicum* comb. nov. tegmina reach onto abdominal segment V and is therefore a feature which differentiates this species from congenerics as the next longest are recorded in *Cryptophylliumoyae* comb. nov. and *Cryptophylliumyunnanense* comb. nov. which only reach to the middle of abdominal segment IV.

###### Distribution.

Only known from the areas of Colonia and Kaday on Yap Island, Micronesia.

##### Cryptophyllium yunnanense

Taxon classificationAnimalia

(Liu, 1993) comb. nov.

933CCA9F-6C54-533E-BA59-DD0BC6428B28

**Figure 75.**
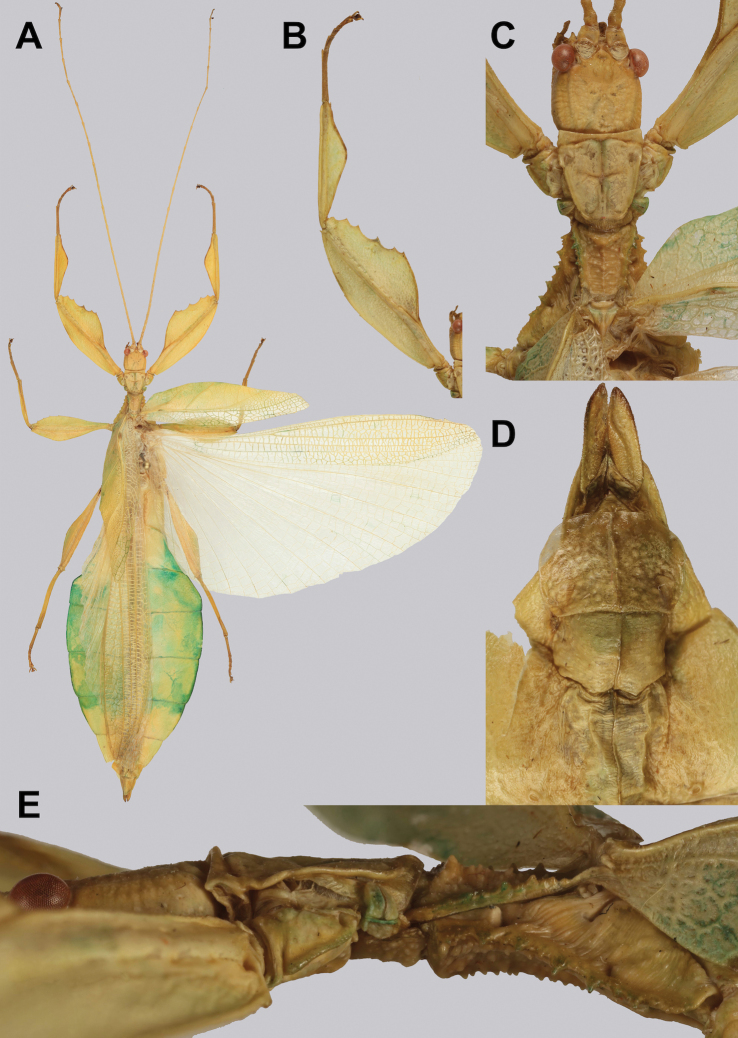
Male *Cryptophylliumyunnanense* comb. nov. (Coll RC 16-120), photographed by RTC **A** habitus, dorsal **B** left front leg details **C** head–thorax details **D** genitalia details, ventral **E** head–thorax details, lateral.

**Figure 76.**
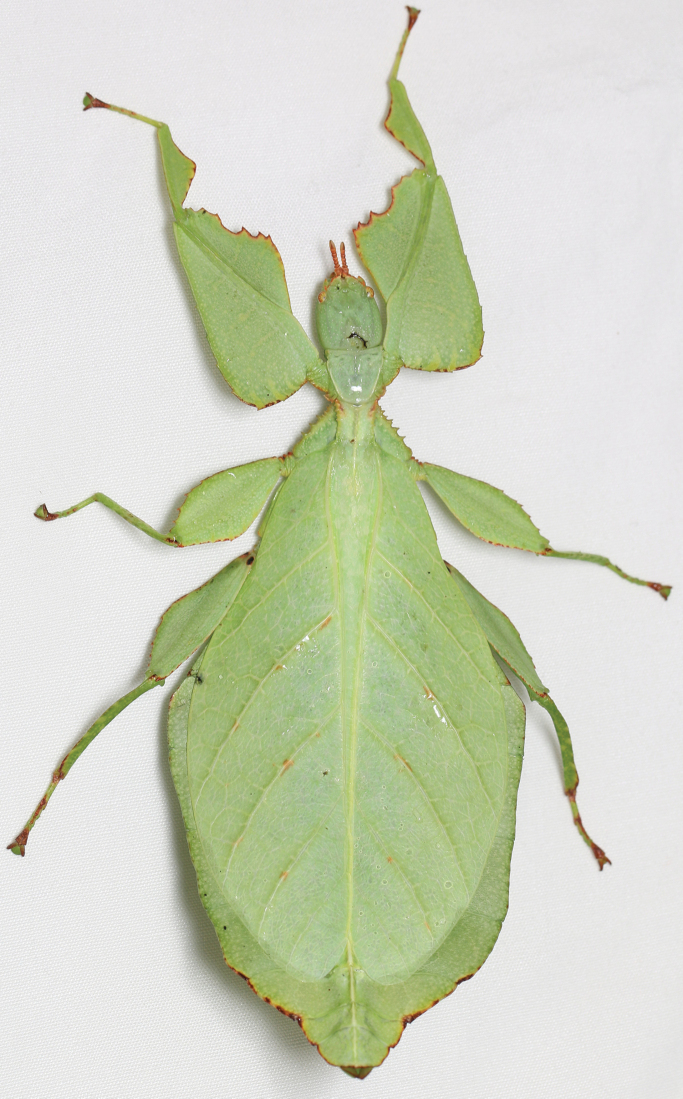
Live female *Cryptophylliumyunnanense* comb. nov. collected and photographed in China, Yunnan, Daweishan, Pinbian County, Honghe Prefecture by Zhiwei Dong (tissue sample DZW04 within our molecular analysis).

**Figure 77.**
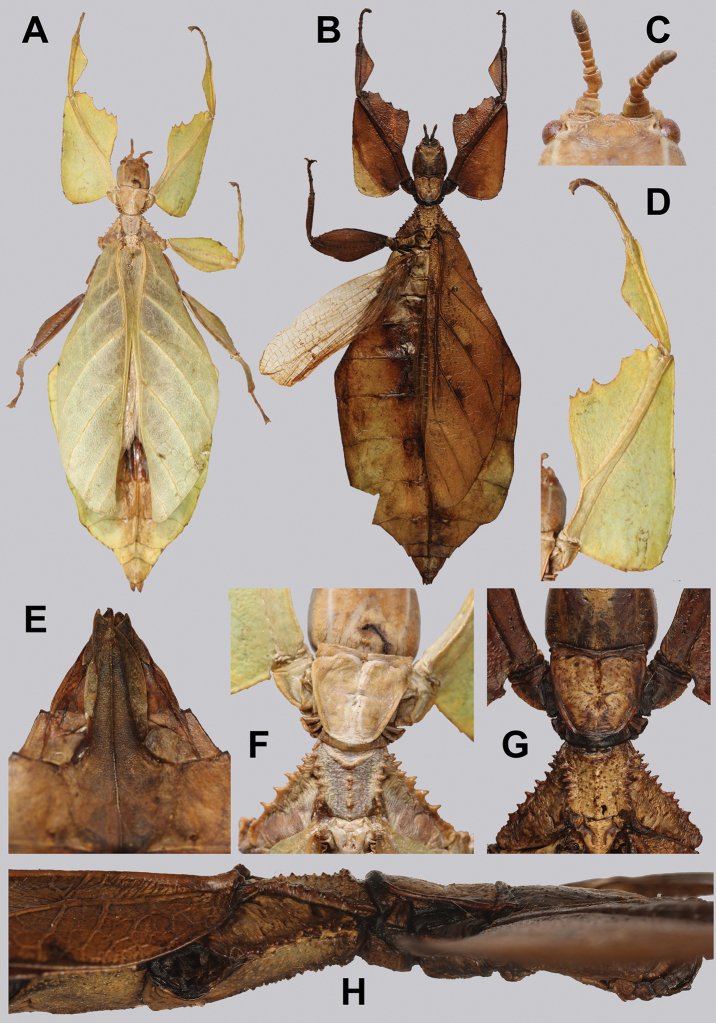
Female *Cryptophylliumyunnanense* comb. nov. **A, C, D, F** are photographs of the female used in our molecular analysis (DZW04) (the same female in Fig. 76) collected by Zhiwei Dong in China, Yunnan, Daweishan, Pinbian County, Honghe Prefecture, photographs by Zhiwei Dong **B, E, G, H** of a female from Vietnam, Lao Cai Province, Mt. Fan-si-pan (Coll RC 17-270), photographs by RTC **A** habitus, dorsal **B** habitus, dorsal **C** details of antennae, dorsal **D** details of front leg, dorsal **E** genitalia, ventral **F** details of thorax, dorsal **G** details of thorax, dorsal **H** details of the thorax, lateral.

Figures 75 , 76 , 77

###### Material examined.

(3 ♀♀, 6 ♂♂): 1 ♀: “Vietnam: Lao Cai Prov. Mt.Fan-si-pan, North Side, 1,600m., 22 17’N 103 44’E, Primary Forest, 28.October-3.November 1994.” (Coll RC 17-270); 1 ♀ nymph: “Lao Cai Province, Sapa mt. 1,600 m.: May 2015” (Coll RC 16-081); 1 ♀: “Yunnan China: Daweishan, Pinbian County, Honghe Prefecture, 3-VI-2017, Zhiwei Dong. (Coll ZD). Molecular sample: DZW04.” (Coll ZD); 3 ♂♂: “Vietnam: Lao Cai Prov. Mt.Fan-si-pan, North Side, 1,600m., 22 17’N 103 44’E, Primary Forest, 20-30. October, 1995” (Coll RC 17-271, 17-272, 17-273); 1 ♂: “Vietnam: Lao Cai Prov. Mt.Fan-si-pan, North Side, 1,600 m., 22 17’N 103 44’E, Primary Forest, 1-7, November, 1995” (Coll RC 17-274); 1 ♂: “Yunnan Province, Xinping Country, Mt. Ailao: June 2015” (Coll RC 16-120); 1 ♂: “Vietnam: Yen Bai, Nghia Lo: June, 2017” (Coll RC 17-240).

###### Remarks.

This species was originally only described from a male holotype from Mongla, Yunnan Province. Liu (1993) differentiated this species from others by the profemoral shape (with an exterior lobe which is narrower than the interior lobe; Fig. 75B) and long antennae with 28 segments. Only the narrow exterior profemoral lobe appears to be a unique feature within the *Cryptophyllium* gen. nov. (as *Cryptophyllium* gen. nov. typically have exterior profemoral lobes broader than the interior), but the 28-segmented antennae are now known to not be unique to *Cryptophylliumyunnanense* comb. nov. alone.

We were able to identify the female sex of *Cryptophylliumyunnanense* comb. nov. within our molecular analysis and illustrate the morphology for the first time (Fig. 76).

###### Differentiation.

Males are large with a broad spade-shaped abdomen, and long antennae and thus are most morphologically similar to *Cryptophylliumoyae* comb. nov. and *Cryptophylliumlimogesi* sp. nov. males. From both species *Cryptophylliumyunnanense* comb. nov. can be differentiated by the shape of the profemoral lobes as the exterior lobe is narrower than the interior lobe (Fig. 75B), vs. a similar width or wider on the exterior in *Cryptophylliumoyae* comb. nov. and *Cryptophylliumlimogesi* sp. nov. males. Additionally, all *Cryptophylliumyunnanense* comb. nov. examined and the holotype illustration clearly lack small anterior exterior tibial lobes (Fig. 75B), whereas *Cryptophylliumoyae* comb. nov. and *Cryptophylliumlimogesi* sp. nov. both have distinct (but small) anterior exterior tibial lobes.

Female *Cryptophylliumyunnanense* comb. nov. are large with mesopleura which are marked with large spine-like tubercles (Fig. 77F, G), and broad boxy abdomen. These features morphologically make them appear similar to *Cryptophylliumtibetense* comb. nov. and *Cryptophylliumlimogesi* sp. nov. females. *Cryptophylliumyunnanense* comb. nov. can be differentiated from *Cryptophylliumlimogesi* sp. nov. by the shape of abdominal segment VII which in *Cryptophylliumlimogesi* sp. nov. is distinctly projecting and slightly recurved vs. *Cryptophylliumyunnanense* comb. nov. which is only gently rounded, not recurved. Additionally, the proximal margin of the exterior profemoral lobe in *Cryptophylliumyunnanense* comb. nov. is straight or slightly rounded (Fig. 77D), not like in *Cryptophylliumlimogesi* sp. nov. where it is distinctly concave, giving the profemoral lobe a slight recurved appearance (Fig. 42F). *Cryptophylliumyunnanense* comb. nov. and *Cryptophylliumtibetense* comb. nov. females share many morphological similarities such as: femoral and tibial lobe shapes and serration on all legs; mesopleura which are marked with prominent spine-like tubercles; alae which are long, reaching abdominal segment VI; rounded boxy abdominal shape; broad gonapophyses VIII; and a long subgenital plate which exceeds the apex of the abdomen (Fig. 77E). The only morphological feature which we have found consistent between these species is the overall shape of the mesopleura which are almost perfectly straight in *Cryptophylliumyunnanense* comb. nov. (Fig. 77F, G) but in *Cryptophylliumtibetense* comb. nov. are slightly narrowed on the anterior, with slightly inward curved margins on the anterior ⅓ (Fig. 63C).

###### Distribution.

Presently only known from southern China (Yunnan Province) and adjacent northern Vietnam (Lai Chau and Yen Bai Provinces).

#### *Cryptophyllium* spp. which are not yet described or identified

***Cryptophyllium* sp. “Andaman**”

With the formal designation of a neotype specimen from the mainland to anchor the name *Cryptophylliumwestwoodii* comb. nov. to, this leaves the second syntype of Wood-Mason (1875) without formal description. Unfortunately, just like the male syntype the female syntype from “South Andaman” is considered lost (Hennemann et al. 2009). Although a rare locality for phylliids in general, all records> we have seen thus far represent a Phyllium (Pulchriphyllium) bioculatum-like species and we have never seen a specimen or photograph of a *Cryptophyllium* gen. nov. species from the Andaman Islands. Therefore, until actual specimens are recorded confidently from the Andaman Islands, we must leave this locality as simply speculative based on historic literature for the time being.

***Cryptophyllium* sp. “Cebu**”

**Figure 78.**
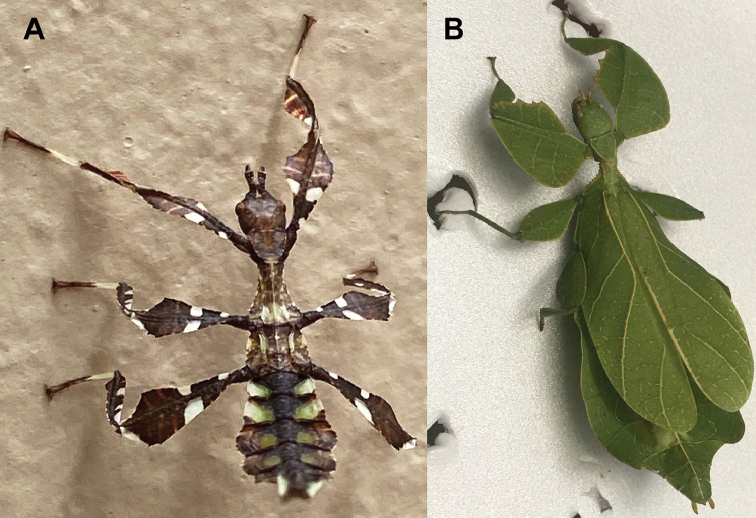
Live undescribed *Cryptophyllium* gen. nov. species which could not be described herein **A** freshly hatched nymph from Cebu, Philippines. Observed and photographed by Annalyn Gabutan Lazaro (Cebu, Philippines) March 2020 **B** adult *Cryptophyllium* gen. nov. species “Zamboanga” observed and photographed by iNaturalist user jonasg1985 (https://www.inaturalist.org/photos/60733716) in August 2019 and used under Creative Commons license (CC BY-NC 4.0).

With the removal of *Phylliumericoriai* and *Phylliumbonifacioi* from the “*celebicum* group”, there are now no formally described *Cryptophyllium* gen. nov. from the Philippines. We have however seen several records> of freshly hatched nymphs (Fig. 78A) from throughout the island of Cebu, Philippines which have broad exterior profemoral lobes and abdominal segments II and III with distinct green patches, features which morphologically places them within the *Cryptophyllium* gen. nov., not the true *Phyllium* that dominate the Philippines.

The coloration present on these Cebu nymph observations likens them most to *Cryptophylliumcelebicum* comb. nov. from Sulawesi, Indonesia (Fig. 9A). Both species have distinct green patches on abdominal segments II and III, as well as broad white bands across the pro-, meso-, and metafemoral lobes, a feature not seen in other *Cryptophyllium* comb. nov. species. A close relationship between these species geographically makes sense as several phylogenetic studies of non-phylliid organisms show support for a southern route of colonization between the southern Philippine Islands and Sulawesi via the Sangihe Islands (hypothesized by Dickerson (1928) and supported by examples such as Evans et al. (2003) with their study on Fanged Frogs). Hopefully adult specimens of this yet to be described species can be located and formally described to help clarify the distribution of the *Cryptophyllium* gen. nov.

***Cryptophyllium* sp. “Zamboanga**”

This observational record also gives support for a *Cryptophyllium* species in the southern Philippines, this time on the Zamboanga Peninsula on the southern end of the island of Mindanao (Fig. 78B). Morphologically this species also suggests a close relationship to *Cryptophylliumcelebicum* comb. nov. from Sulawesi, Indonesia (Fig. 2) due to the broad angular profemoral exterior lobes and the mesopleura which are extremely narrow on the anterior margin instead of reaching fully to the anterior. With no specimens at hand we do not yet know if this Zamboanga species is the same as that found on Cebu, but with them inhabiting separate islands we expect they are sister species and likely not identical. Hopefully additional specimens are located and can be included in a phylogenetic analysis to elucidate this species taxonomic placement.

***Cryptophyllium* sp. “Bangka Island**”

No phylliids are yet officially recorded from Bangka Island, Indonesia, but we have seen one photo of an adult female reportedly from this island. The individual observed was also like *Cryptophylliumcelebicum* comb. nov. with strongly angled profemoral exterior lobes and mesopleura which are extremely narrow on the anterior margin. Interestingly, however, the female in the photo appeared to be rather small, not as large as typical *Cryptophylliumcelebicum* comb. nov. and we hope that one day specimens can be properly photographed and collected to confirm this observation.

***Cryptophyllium* sp. “Myanmar**”

Photos of two males from Myanmar, Mandalay Region, Myingyan District, were shared with us by Chih-Ting Hsu (Taiwan) from within the collection of the National Museum of Natural Sciences, Taiwan. These two males are geographically from an area where the phylliid knowledge is poor at the present and therefore these are the only records> we have seen from central Myanmar. Morphologically they appear similar to *Cryptophylliumtibetense* comb. nov. but they lack small exterior lobes which help to characterize that species. It is possible that these specimens represent an undescribed species or the unknown male for one of the herein described species only known from females. Hopefully future molecular analysis can reveal the identity of these specimens.

### Key to known males in the *Cryptophyllium* gen. nov.

Male *Cryptophylliumdaparo* sp. nov., *Cryptophylliumechidna* sp. nov., *Cryptophylliumliyananae* sp. nov., *Cryptophylliumwennae* sp. nov., and *Cryptophylliumnuichuaense* sp. nov. are presently unknown and therefore excluded.

**Table:** 

1	Protibial interior lobe distributed across the entire length of the shaft either with even weighting on the distal and proximal halves, or with the weighting on the distal ½, not on the proximal ½; tegmina at most reaching onto abdominal segment IV, but typically only onto segment II or III	**2**
–	Protibial interior lobe unevenly distributed, almost entirely on the proximal ½, with the distal ½ greatly reduced; tegmina long, reaching at least onto abdominal segment V	***Cryptophylliumyapicum* (Cumming & Teemsman, 2018), comb. nov.**
2	Profemoral interior lobe with seven or eight small teeth of even size and spacing, with shallow gaps between them	***Cryptophylliumanimatum* sp. nov.**
–	Profemoral interior lobe with teeth which are uneven in size, generally with the middle tooth larger than the teeth on either end, generally teeth arranged in a two-one-two pattern with large looping gaps between the sets	**3**
3	Metatibial exterior with a fully developed lobe spanning the entire length	***Cryptophylliumathanysus* (Westwood, 1859), comb. nov.**
–	Metatibial exterior either lacking a lobe completely, or with at most a small lobe on the distal tip of the metatibiae, not fully developed	**4**
4	Profemoral exterior lobe strongly angled, ca. 90°	***Cryptophylliumcelebicum* (de Haan, 1842), comb. nov.**
–	Profemoral exterior lobe arcing roundly or with a slight bend in the middle which is distinctly obtusely angled	**5**
5	Profemoral exterior lobe with distinct serration throughout the entire length (eight or nine sharp teeth)	***Cryptophylliumdrunganum* (Yang, 1995), comb. nov.**
–	Profemoral exterior lobe either lacking serration or with two to seven teeth on the distal half only, generally only weakly formed, proximal half lacking serration	**6**
6	Tegmina long, reaching the anterior margin of abdominal segment IV or onto it	**7**
–	Tegmina short, only reaching abdominal segment II or at most ca. ¾ of the way onto segment III	**9**
7	Profemoral exterior lobe notably thinner than the interior lobe	***Cryptophylliumyunnanense* (Liu, 1993), comb. nov.**
–	Profemoral exterior and interior lobes about the same width	**8**
8	Tegmina radial sector terminating near the apex of the wing	***Cryptophylliumtibetense* (Liu, 1993), comb. nov.**
–	Tegmina radial sector terminating near the posterior ⅓ of the wing length	***Cryptophylliumoyae* (Cumming & Le Tirant, 2020), comb. nov.**
9	Tegmina, radial sector notably diverging towards the wing margin away from the media anterior near the middle of the wing and terminating ca. ⅓ of the way through the wing length, not near the apex	***Cryptophylliumparum* (Liu, 1993), comb. nov.**
–	Tegmina, radial sector running side by side with the medial for a majority of the length (generally for most of the length but always notably > ½ the length) and terminating near the wing apex near where the media terminates (either at the apex or no more than ⅕ of the way from the apex)	**10**
10	Large species (length from front of head to apex of abdomen > 80.0 mm)	**11**
–	Small to medium species (length from front of head to apex of abdomen 60.0–74.0 mm, but typically < 70.0 mm)	**13**
11	Abdomen thin and dagger-shaped, at its widest < ½ of overall abdominal length	***Cryptophylliumfaulkneri* sp. nov.**
–	Abdomen broad and ovular or broad and spade-shaped with the greatest width at least half the length of the abdomen	**12**
12	Protibial exterior with a small but distinct lobe on the distal ⅕	***Cryptophylliumlimogesi* sp. nov.**
–	Protibial exterior smooth, lacking a lobe	***Cryptophylliumrarum* (Liu, 1993), comb. nov.**
13	Profemoral interior lobe broader than the exterior lobe	**14**
–	Profemoral interior lobe the same width as the exterior lobe or slightly thinner	**15**
14	Profemoral exterior lobe at its widest ca. 2 ⅓× the greatest width of the profemoral shaft	***Cryptophylliumicarus* sp. nov.**
–	Profemoral exterior lobe at its widest ca. 3× the greatest width of the profemoral shaft	***Cryptophylliumbankoi* sp. nov.**
15	Abdomen shape thinly elliptical, with a maximum width only 30–34% the abdominal length	**16**
–	Abdominal shape broadly elliptical or broadly spade-shaped with a maximum width ca. 38–45% the abdominal length	**17**
16	Prescutum surface shorter than the length of the pronotum; overall size generally not as large, 63 to 70 mm long	***Cryptophylliumwestwoodii* (Wood-Mason, 1875), comb. nov.**
–	Prescutum surface longer than the pronotum length; overall size generally larger, 73 to 74 mm long	***Cryptophylliumchrisangi* (Seow-Choen, 2017), comb. nov.**
17	Due to intraspecific variation of the following three species we could not identify a reliable morphological feature for differentiation within the males. Female morphology between these species does allow differentiation, and of course molecular analysis (Fig. 4) allows reliable differentiation even between these variable and difficult to distinguish species	***Cryptophylliumbollensi* sp. nov., *Cryptophylliumphami* sp. nov., *Cryptophylliumkhmer* sp. nov.**

### Key to known females in the *Cryptophyllium* gen. nov.

Female *Cryptophylliumanimatum* sp. nov., *Cryptophylliumbankoi* sp. nov., and *Cryptophylliumfaulkneri* sp. nov. are unknown and therefore excluded.

**Table:** 

1	Metatibial exterior with a fully developed lobe spanning the entire length	***Cryptophylliumathanysus* (Westwood, 1859), comb. nov.**
–	Metatibial exterior either lacking a lobe completely or with at most a small lobe on the distal tip of the metatibiae only, not fully developed	**2**
2	Protibial interior lobe unevenly distributed, almost entirely on the proximal half, with the distal half greatly reduced	***Cryptophylliumyapicum* (Cumming & Teemsman, 2018), comb. nov.**
–	Protibial interior lobe distributed across the entire length of the shaft either with even weighting on the distal and proximal halves, or with the weighting on the distal half, not on the proximal half	**3**
3	Alae under-developed, not reaching abdominal segment II	***Cryptophylliumicarus* sp. nov.**
–	Alae moderately to well-developed, at least reaching onto abdominal segment II	**4**
4	Profemoral exterior lobe proximal margin slightly recurved giving the lobe an acute recurved shape	**5**
–	Profemoral exterior lobe proximal margin straight or roundly arcing, not recurved	**7**
5	Mesopleura anterior half notably narrower than the posterior, giving the margin a distinct bend, with the anterior half only about as wide as the prescutum width	***Cryptophylliumcelebicum* (de Haan, 1842), comb. nov.**
–	Mesopleura broad with straight margins throughout the length, anterior half wider than the prescutum width	**6**
6	Terminal antennal segment about as long as the preceding two segments combined	***Cryptophylliumwennae* sp. nov.**
–	Terminal antennal segment about as long as the preceding three segments combined	***Cryptophylliumlimogesi* sp. nov.**
7	Mesopleura with straight margins which reach fully to the anterior of the prescutum, not significantly narrowed on the anterior ⅓ to ½	**8**
–	Mesopleura with margins that are distinctly bent because the anterior ⅓ to ½ is narrow, the same width as the prescutum	**13**
8	Tibial exteriors simple, lacking lobes	**9**
–	Tibial exteriors with small but distinct lobes on the distal ⅕ (those on the pro- and metatibiae are generally best formed, mesotibiae not very well formed)	**11**
9	Mesopleura lateral margins with numerous granules of approximately uniform size, no prominent tubercles	***Cryptophylliumparum* (Liu, 1993), comb. nov.**
–	Mesopleura with eight or nine distinct tubercles of varying sizes, of those usually four or five are notably larger than the rest	**10**
10	Subgenital plate long, with the apex reaching the tip of the abdomen	***Cryptophylliumyunnanense* (Liu, 1993), comb. nov.**
–	Subgenital plate shorter, with the apex not reaching the tip of the abdomen	***Cryptophylliumoyae* (Cumming & Le Tirant, 2020), comb. nov.**
11	Subgenital plate long, apex exceeding the tip of the abdomen	***Cryptophylliumtibetense* (Liu, 1993), comb. nov.**
–	Subgenital plate short, apex not exceeding the tip of the abdomen	**12**
12	Prescutum lateral margins parallel from the anterior to the posterior; abdominal segment VII with distinct recurved lobes which project past the posterior margin of the segment	***Cryptophylliumdrunganum* (Yang, 1995), comb. nov.**
–	Prescutum lateral margins flared out slightly on the anterior margin so that the posterior margin is slightly thinner; abdominal segment VII only slightly rounded, lacking a strongly curved lobe that projects	***Cryptophylliumliyananae* sp. nov.**
13	Alae short, only reaching abdominal segments III or IV	**14**
–	Alae long, reaching abdominal segments VI or VII	**18**
14	Profemoral exterior lobe with straight margins and a right angle	***Cryptophylliumechidna* sp. nov.**
–	Profemoral exterior lobe rounded, arcing end to end with an obtuse angle	**15**
15	Alae short, only reaching the anterior margin of abdominal segment III	***Cryptophylliumphami* sp. nov.**
–	Alae moderate length, reaching at least onto abdominal segment IV	**16**
16	Ventral surface of the antennae with segments VI, VII, and VIII flush	***Cryptophylliumbollensi* sp. nov.**
–	Ventral surface of the antennae with segments VI and VII projecting beyond segment VIII, giving the antennae a slight lamellate appearance	**17**
17	Prescutum equal in length to the pronotum; alae reaching ½–¾ of the way onto abdominal segment IV	***Cryptophylliumchrisangi* (Seow-Choen, 2017), comb. nov.**
–	Prescutum length shorter than the length of the pronotum; alae only reaching the anterior margin of abdominal segment IV, not significantly onto it	***Cryptophylliumnuichuaense* sp. nov.**
18	Abdominal segments V, VI, and VII with lateral margins which are converging, VII lacking a distinct bend	**19**
–	Abdominal segments V and VI with lateral margins that are parallel, giving the abdomen a boxy appearance, VII distinctly rounded or with a slight projecting lobe	**20**
19	Profemoral exterior lobe obtusely angled	***Cryptophylliumdaparo* sp. nov.**
–	Profemoral exterior lobe distinctly right angled	***Cryptophylliumrarum* (Liu, 1993), comb. nov.**
20	For these last two species we were not able to identify a stable morphological feature to allow reliable differentiation of the females. *Cryptophylliumwestwoodii* comb. nov. females are rather morphologically variable thus preventing us from identifying a single feature which was consistently different from *Cryptophylliumkhmer* sp. nov. females. Only through molecular analysis can females reliably be differentiated (Fig. 4), but clues from male morphology (which do allow differentiation of these two species) and geographic distribution (Fig. 2) can help to differentiate these species	***Cryptophylliumkhmer* sp. nov., *Cryptophylliumwestwoodii* (Wood-Mason, 1875), comb. nov.**

### Phylliidae conservation in Vietnam

Using the IUCN’s Red List criteria and categories (www.iucnredlist.org), the Vietnam red data book consists of a list of endangered species of flora and fauna from Vietnam and forms the legal basis for protecting biodiversity in Vietnam. One species of phylliid, *Phylliumsiccifolium* (Linnaeus, 1758), is currently included as vulnerable in the Vietnam red data book and was recorded from the provinces of Quang Binh, Lao Cai, Vinh Phuc, Hoa Binh, Ninh Binh, and Ha Tay (Thinh and Tru 2008). Later, Thinh and Tru (2011) removed *Phylliumsiccifolium* from the country’s species list and identified most the phylliid diversity within Vietnam as ‘*Phylliumwestwoodii* Wood-Mason, 1875’ based on Hennemann et al. (2009) as well as adding ‘*Phylliumparum*’ and ‘*Phylliumyunnanense*’ to the species recorded from Vietnam. We now know that the widespread distribution mentioned by Thinh and Tru (2008; 2011) instead refers to multiple species now placed within the *Cryptophyllium* gen. nov. Our present study has not identified any possible records> of *Cryptophylliumwestwoodii* comb. nov. from Vietnam, despite the examination of numerous specimens.

Red data species are used as indicator species to assess the effectiveness of protected areas and their conservation. Derunkov et al. (2019) compared the effectiveness of protected areas in Belarus and Vietnam and noted that the poorly studied Vietnamese insect fauna, the large number of endemics, and undescribed species makes it problematic to use this criterion. Adding a species to the red list remains difficult as many species are known from few specimens and current sampling is therefore inadequate to understand their true degree of rarity.

Constant et al. (2018) highlighted the degree of endemism in Vietnam by the collecting three different leaf insect species in three national parks over a short distance of 60 km, which are herein described as *Cryptophylliumicarus* sp. nov. (Bidoup-Nui Ba National Park), *Cryptophylliumbollensi* sp. nov. (Phuoc Binh National Park, and *Cryptophylliumnuichuaense* sp. nov. (Nui Chua National Park). The authors conclude that due to the high endemism, more protected areas need to be created and existing areas should be connected through corridors to properly protect Vietnam’s biodiversity.

**Table 1.** Summary of which adult sex, egg, and freshly hatched nymph morphologies are presently known as well as notes on the adult morphological differentiation. Corresponding figures are referenced in the columns with hyphens “-” indicating an unknown for that species. Note that for *C.bankoi* sp. nov. only nymph females are known, not adults, and for *C.athanysus* comb. nov. eggs only are known from an illustration in Greene (1906).

Species	♀	♂	Egg	Nymph	Morphological differentiation
*C.animatum*	–	10A	–	–	♂: Couplet 2, profemoral interior lobe
*C.athanysus*	11A	12C	11D	–	♀: Couplet 1, metatibial exterior lobe
					♂: Couplet 3, metatibial exterior lobe
*C.bankoi*	13B	14C	–	–	♀: Adult female unknown and therefore excluded from the key
					♂: Couplet 14, profemoral exterior lobe
*C.bollensi*	16	17B	21A	9H	♀: Couplet 17, ventral antennae
					♂: Morphologically indistinguishable from *C.phami* and *C.khmer*
*C.celebicum*	22A	23D	8O	9A	♀: Couplet 5, mesopleura anterior
					♂: Couplet 4, profemoral exterior lobe
*C.chrisangi*	24A	24C	8M	9B	♀: Couplet 17, prescutum and alae
					♂: Couplet 16, prescutum and overall length
*C.daparo*	27A	–	–	–	♀: Couplet 19, profemoral exterior lobe
*C.drunganum*	28A	29A	–	–	♀: Couplet 12, prescutum lateral margins
					♂: Couplet 5, profemoral exterior lobe
*C.echidna*	30A	–	–	–	♀: Couplet 14, profemoral exterior lobe
*C.faulkneri*	–	31B	–	–	♂: Couplet 11, abdomen shape
*C.icarus*	32A	33B	36A	9I	♀: Couplet 3, alae under-developed
					♂: Couplet 14, profemoral exterior lobe
*C.khmer*	38A	38C	41A	9C	♀: Morphologically indistinguishable from *C.westwoodii*
					♂: Morphologically indistinguishable from *C.phami* and *C.bollensi*
*C.limogesi*	42A	43A	45A	–	♀: Couplet 6, terminal antennal segment
					♂: Couplet 12, protibial exterior
*C.liyananae*	46A	–	8K	–	♀: Couplet 12, prescutum lateral margins
*C.nuichuaense*	49A	–	–	–	♀: Couplet 17, prescutum and alae length
*C.oyae*	50A	50B	8G	9E	♀: Couplet 10, subgenital plate
					♂: Couplet 8, tegmina venation
*C.parum*	53E	53A	–	–	♀: Couplet 9, mesopleura lateral margins
					♂: Couplet 9, tegmina venation
*C.phami*	55A	55C	58A	9G	♀: Couplet 15, alae length
					♂: Morphologically indistinguishable from *C.khmer* and *C.bollensi*
*C.rarum*	59C	59A	–	–	♂: Couplet 12, protibial exterior
*C.tibetense*	63A	64B	8C	9D	♀: Couplet 11, subgenital plate
					♂: Couplet 8, tegmina venation
*C.wennae*	67A	–	–	–	♀: Couplet 6, terminal antennal segment
*C.westwoodii*	68A	68B	8A	9F	♀: Morphologically indistinguishable from *C.khmer*
					♂: Couplet 16, prescutum length
*C.yapicum*	73A	74E	–	–	♀: Couplet 2, protibial interior lobe
					♂: Couplet 1, protibial interior lobe
*C.yunnanense*	75A	76	–	–	♀: Couplet 10, subgenital plate
					♂: Couplet 7, profemoral exterior lobe

## Discussion

Intensive morphological investigation of the female, male, and egg morphology has revealed the presence of diagnostic morphological characters to distinguish the members of the ‘Phyllium (Phyllium) celebicum species group’ from the remaining phylliid species. The respective species are those with females that have the fourth antennal segment short and disk-like (Fig. 6B–H), males with an apically two-hooked vomer (Fig. 5), and eggs, which are rectangular with short moss-like pinnae (Fig. 8). Two former *celebicum* species group members, *Phylliumericoriai* and *P.bonifacioi*, do not match these characteristics and are morphologically more similar to the remaining Phyllium (Phyllium) species.

In agreement with the morphological results, our molecular phylogeny recovers the species of the *celebicum* species group (excluding *P.ericoriai* and *P.bonifacioi*) as a strongly supported monophyletic group distinct from the remaining *Phyllium* species (Fig. 4). In fact, *Phyllium* is rendered paraphyletic, which has already been demonstrated in previous molecular studies, albeit in regard to *Chitoniscus* instead of *Microphyllium* + *Pseudomicrophyllium* (Buckley et al. 2009; Bradler et al. 2015; Robertson et al. 2018). *Phylliumericoriai* + *P.bonifacioi* are not recovered within the *celebicum* species group, but as sister taxon to the remaining Phyllium (Phyllium), which corresponds to our assumptions made based on morphology. Our results also corroborate that the possession of well-developed hind wings, which was originally characterizing the females of the Phyllium (Phyllium) celebicum species group (Hennemann et al. 2009), is an inappropriate character to define taxonomic groups since it must be interpreted *a priori* as a plesiomorphic trait. In consequence, our combined results of morphological examination and molecular analysis justify the erection of a new genus, *Cryptophyllium* gen. nov., into which the members of the former *celebicum* species group (excl. *P.ericoriai* and *P.bonifacioi*) are transferred.

Within *Cryptophyllium* gen. nov., we discovered a high species diversity. Most of the 55 specimens used for the phylogenetic analysis could be morphologically distinguished into nine of the already described species (with *C.athanysus* comb. nov. and *C.yapicum* comb. nov. being the only members without representatives in our molecular analysis). The 13 new species described herein have all been recovered as distinct clades in the molecular phylogeny. In two cases, the phylogenetic analysis unveiled morphologically unrecognized (cryptic) species: Based on morphology alone, *C.khmer* sp. nov. and *C.bollensi* sp. nov. had originally been considered to belong to *C.westwoodii* comb. nov. and *C.phami* sp. nov., respectively. Although morphologically nearly inseparable, we recovered *C.chrisangi* comb. nov. and *C.nuichuaense* sp. nov. as sister taxa to *C.westwoodii* comb. nov. and *C.phami* sp. nov., respectively. The presence of distinct species is further supported by their geographic distribution. As a consequence of recognizing *C.khmer* sp. nov. as a distinct species, records> from the region previously believed to be within the distributional range of *C.westwoodii* comb. nov. have to be considered uncertain until genetically confirmed (see multi-colored symbols in Fig. 2). The possibility of additional unrecognized species existing within the wide range of *C.westwoodii* comb. nov. cannot be excluded. This potentially also applies to *C.rarum* comb. nov. and its range spanning from southern China to central Vietnam. Despite the significant genetic distances, the specimens examined in this study are morphologically indistinguishable, but due to the lack of sufficient specimens and representatives from across the whole range, we decided to treat them as one single species. Similar issues may be identified regarding several other species (e.g., *C.celebicum* comb. nov., *C.tibetense* comb. nov.) and can only be resolved with a more comprehensive taxon sampling across species’ ranges.

Identifying suitable diagnostic characters in Phylliidae has long been difficult due to the poor understanding of their true diversity as more species have been described in the last twenty years than the previous entire taxonomic history of this group (for overview of the taxonomic progress see Brock et al. 2020). Only recent extensive morphological comparisons of sometimes subtle features such as wing venation and egg morphology (Cumming et al. 2020a, b, c) revealed traits of potential taxonomic value. Cumming et al. (2020c) proposed the significance of using wing venation for differentiation among the *Phyllium* subgenera, which we confirm to be applicable for *Cryptophyllium* gen. nov. males but not to differentiate females. In the male *Cryptophyllium* gen. nov. hind wings, the MA and MP fuse and run to the wing margin (first noted in Bradler 2009: fig. 12a), which is a derived character distinguishable from the remaining *Phyllium* species. Interestingly, this pattern is also exhibited by the apparently unrelated *Nanophylliumpygmaeum* species group (Cumming et al. 2020c).

A truly unique characteristic of *Cryptophyllium* gen. nov. is the vomer, which in contrast to other extant leaf insects bears two hooks instead of one (Fig. 5; see also Wedmann et al. 2007; Bradler 2009; Größer 2011). The two-hooked vomer was also detected in the leaf insect fossil *Eophylliummesselense* (Wedmann et al. 2007) and might represent the plesiomorphic condition in Phylliidae. Since the vomer can also be two-hooked in some African and Malagasy non-phylliid phasmid species (Bradler 2009) that are likely to be closely related to Phylliidae (Simon et al. 2019), these lineages might share this ancestral trait, albeit under consideration of multiple secondary losses. However, the more parsimonious explanation appears to be convergent evolution, but this needs to be inferred more formally in a broader phylogenetic investigation. Although Wedmann et al. (2007) hypothesized *Eophyllium* to be sister taxon to all extant leaf insects, the shared two-hooked vomer could alternatively be an indicator of common ancestry with *Cryptophyllium* gen. nov., questioning the assumed phylogenetic position of *Eophyllium* and in consequence also the minimum age of Phylliidae radiation.

The combination of genetic and morphological analyses has been shown to be a valuable tool for phylliid taxonomy, but in order to illuminate trait evolution in the Phylliidae, we are in need of a robust phylogeny including a sufficient number of representatives of all lineages. Only through additional extensive fieldwork and targeted collecting (such as through the collaborative GTI expeditions which yielded many of the specimens utilized within this study) will sufficient material be collected to elucidate the hidden phylliid diversity and provide material for future molecular analyses. Future molecular analyses will be essential to further match up opposite sexes of known *Cryptophyllium* gen. nov. members and in particular to unveil the cryptic diversity of Phylliidae.

## Supplementary Material

XML Treatment for Cryptophyllium

XML Treatment for Cryptophyllium animatum

XML Treatment for Cryptophyllium athanysus

XML Treatment for Cryptophyllium bankoi

XML Treatment for Cryptophyllium bollensi

XML Treatment for Cryptophyllium celebicum

XML Treatment for Cryptophyllium chrisangi

XML Treatment for Cryptophyllium daparo

XML Treatment for Cryptophyllium drunganum

XML Treatment for Cryptophyllium echidna

XML Treatment for Cryptophyllium faulkneri

XML Treatment for Cryptophyllium icarus

XML Treatment for Cryptophyllium khmer

XML Treatment for Cryptophyllium limogesi

XML Treatment for Cryptophyllium liyananae

XML Treatment for Cryptophyllium nuichuaense

XML Treatment for Cryptophyllium oyae

XML Treatment for Cryptophyllium parum

XML Treatment for Cryptophyllium phami

XML Treatment for Cryptophyllium rarum

XML Treatment for Cryptophyllium tibetense

XML Treatment for Cryptophyllium wennae

XML Treatment for Cryptophyllium westwoodii

XML Treatment for Cryptophyllium yapicum

XML Treatment for Cryptophyllium yunnanense

## Footnotes

Antennae of the holotype broken, measurement is only until segment 23.

Only measured on the largest specimen, the smallest are missing their front legs.

Smallest paratype female missing the middle legs and the alae are covered by the tegmina.

Only one paratype in the RBINS collection.

Wrinkled in the paratype.

## Supplementary materials

Supplementary material 1 Figures S1, S2 Data type Additional images of molecularly sampled specimens. Explanation note With this work including a large phylogeny not every specimen sampled was included as a figure within the main article. To ensure reproducibility/traceability of our work for future authors we have illustrated additional specimens within two supplementary plates. https://binary.pensoft.net/file/510543This dataset is made available under the Open Database License (http://opendatacommons.org/licenses/odbl/1.0/). The Open Database License (ODbL) is a license agreement intended to allow users to freely share, modify, and use this Dataset while maintaining this same freedom for others, provided that the original source and author(s) are credited.Jérôme Constant

Supplementary material 2 Multiple sequence alignment of concatenated dataset Data type phylogenetic data https://binary.pensoft.net/file/510544This dataset is made available under the Open Database License (http://opendatacommons.org/licenses/odbl/1.0/). The Open Database License (ODbL) is a license agreement intended to allow users to freely share, modify, and use this Dataset while maintaining this same freedom for others, provided that the original source and author(s) are credited.Sarah Bank

Supplementary material 3 TreeFile Data type phylogenetic data Explanation note This is the resulting treefile generatted from our phylogenetic analysis. https://binary.pensoft.net/file/510545This dataset is made available under the Open Database License (http://opendatacommons.org/licenses/odbl/1.0/). The Open Database License (ODbL) is a license agreement intended to allow users to freely share, modify, and use this Dataset while maintaining this same freedom for others, provided that the original source and author(s) are credited.Sarah Bank

Supplementary material 4 Table S1. Specimen data and deposition for localities used within the distribution map (Fig. 2) Data type specimen data https://binary.pensoft.net/file/510546This dataset is made available under the Open Database License (http://opendatacommons.org/licenses/odbl/1.0/). The Open Database License (ODbL) is a license agreement intended to allow users to freely share, modify, and use this Dataset while maintaining this same freedom for others, provided that the original source and author(s) are credited.Sarah Bank, Royce Cumming

Supplementary material 5 Table S2. All sampled specimens are listed with their collection data, deposition location, and GenBank accession numbers for the generated sequences Data type specimen data Explanation note Taxon information for each specimen used in the phylogenetic analysis including sex, type designation, sample ID, sampling site, and GenBank accession numbers. Newly generated molecular data in bold. https://binary.pensoft.net/file/510547This dataset is made available under the Open Database License (http://opendatacommons.org/licenses/odbl/1.0/). The Open Database License (ODbL) is a license agreement intended to allow users to freely share, modify, and use this Dataset while maintaining this same freedom for others, provided that the original source and author(s) are credited.Sarah Bank, Royce Cumming
