# Supplementary material for: Cryptophyllium, the hidden leaf insects – descriptions of a new leaf insect genus and thirteen species from the former celebicum species group (Phasmatodea, Phylliidae)
Source: Zookeys. 2021 Feb 18;1018:1–179. doi: 10.3897/zookeys.1018.61033 (PMC7907054; doi:10.3897/zookeys.1018.61033)
Supplement: Supplementary material 1 — Figures S1, S2 [file zookeys-1018-001-s001.docx]

**
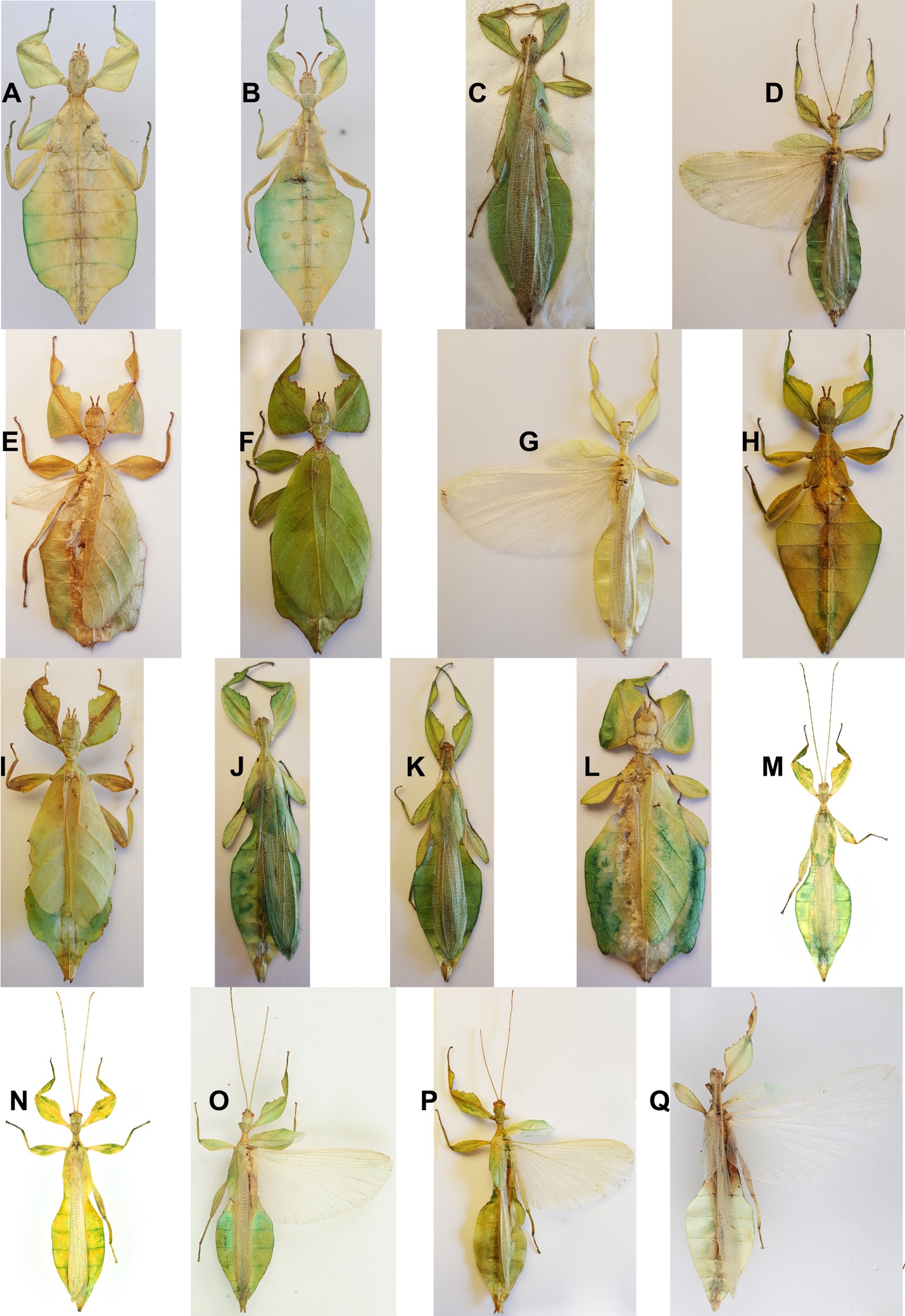
**

**Supplementary Figure 1:** Additional *Cryptophyllium* gen. nov. specimens which were molecularly sampled but not figured within the primary work.

**A** *Cryptophyllium bankoi* gen. et sp. nov. female nymph, Vietnam, Bach Ma National Park, RBINS-PHYLLIUM DNA sample 0006 (RBINS).

**B** *Cryptophyllium bankoi* gen. et sp. nov. male nymph, Vietnam, Kon Chu Rang Nature Reserve, RBINS-PHYLLIUM DNA sample 0008 (RBINS).

**C** *Cryptophyllium bankoi* gen. et sp. nov. male paratype, Vietnam, Dak Nong, 17-338 (Coll RC).

**D** *Cryptophyllium bollensi* gen. et sp. nov. male paratype, Vietnam, Phuoc Binh National Park, 18-217 (Coll RC).

**E** *Cryptophyllium celebicum* comb. nov. female, Indonesia, Sulawesi, 16-075 (Coll RC).

**F** *Cryptophyllium celebicum* comb. nov. female, Indonesia, Peleng Island, 19-181 (Coll RC).

**G** *Cryptophyllium chrisangi* comb. nov. male, Singapore, Pulau Ubin, 18-279 (Coll RC).

**H** *Cryptophyllium faulkneri* gen. et sp. nov. male nymph, Vietnam, Lam Dong Province, 16-236 (Coll RC).

**I** *Cryptophyllium icarus* gen. et sp. nov. female, Vietnam, Bidoup-Nui Ba National Park, 18-226 (Coll RC).

**J** *Cryptophyllium icarus* gen. et sp. nov. male, Vietnam, Dak Lak Province, 18-408 (Coll RC).

**K** *Cryptophyllium icarus* gen. et sp. nov. male, Vietnam, Lam Dong Province, Bao Loc, 17-267 (Coll RC).

**L** *Cryptophyllium icarus* gen. et sp. nov. male, Vietnam, Lam Dong Province, Bao Loc, SLT005 (Coll SLT).

**M** *Cryptophyllium oyae* comb. nov. female paratype, Laos, Hua Phan Province, Ban Saleui, 18-423 (Coll RC).

**N** *Cryptophyllium phami* gen. et sp. nov., Vietnam, Bin Thuan Province, Dong Tien, SLT03 (Coll SLT).

**O** *Cryptophyllium rarum* comb. nov. male, Vietnam, Kon Tum Province, Ngoc Linh Mt, 16-116 (Coll RC).

**P** *Cryptophyllium rarum* comb. nov. male, Vietnam, Da Nang Province, Ba Na Mt 16-119 (Coll RC).

**Q** *Cryptophyllium rarum* comb. nov. male, Vietnam, Tay Yen Tu Nature Reserve, RBINS-PHYLLIUM DNA sample 0005 (RBINS).


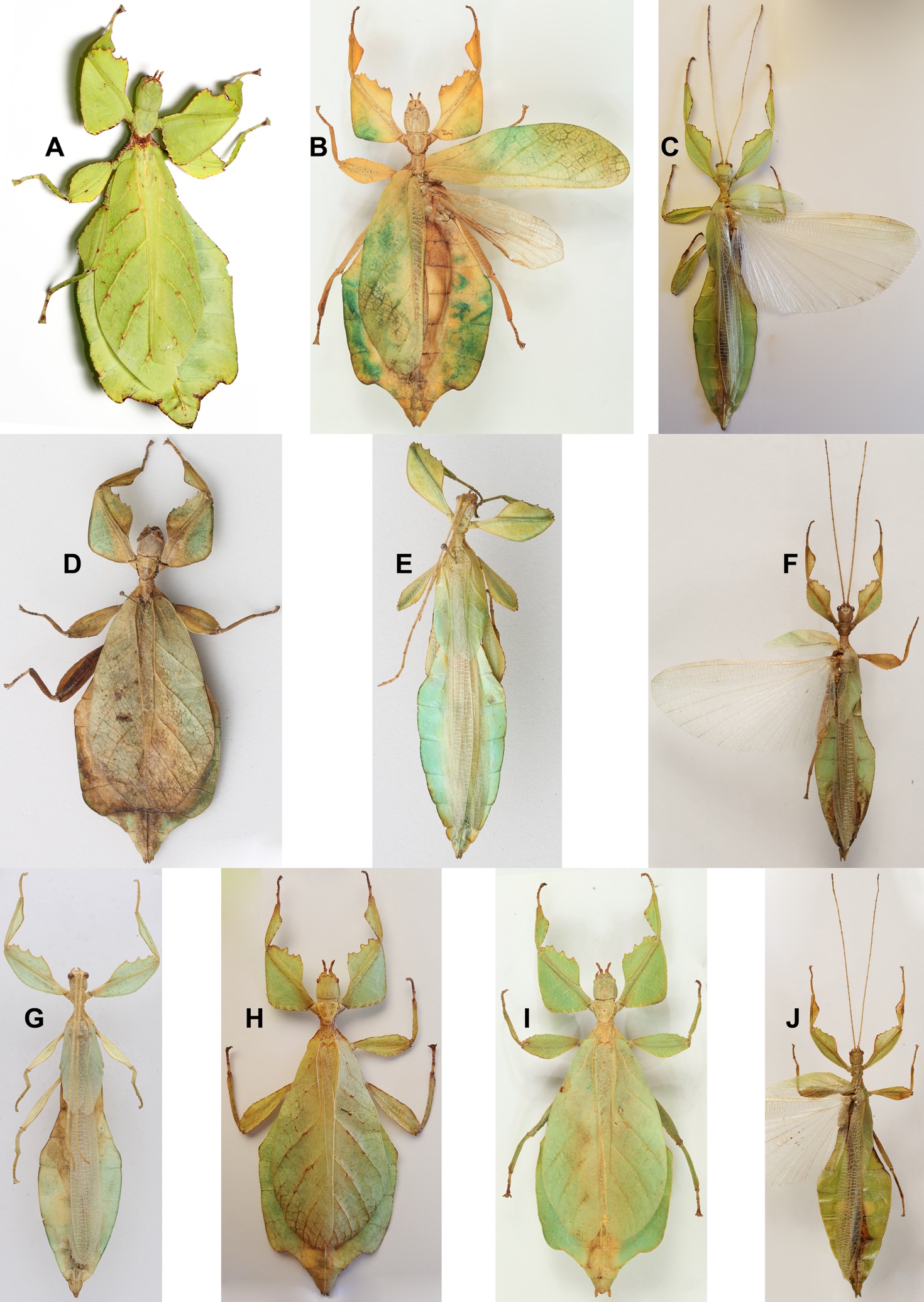


**Supplementary Figure 2:**

**A** *Cryptophyllium tibetense* comb. nov. female, Tibet, Medog, DZW007 (Coll ZD).

**B** *Cryptophyllium westwoodii* comb. nov. female, Laos, Kiew Mak Nao, 16-077 (Coll RC).

**C** *Cryptophyllium westwoodii* comb. nov. male, Laos, Kiew Mak Nao, 18-030 (Coll RC).

**D** *Cryptophyllium westwoodii* comb. nov. female, Laos, Muang Feuang, DZW013 (Coll ZD).

**E** *Cryptophyllium westwoodii* comb. nov. male, Laos, Muang Feuang, DZW010 (Coll ZD).

**F** *Cryptophyllium westwoodii* comb. nov. male, Myanmar, Karathuri, 18-029 (Coll RC).

**G** *Cryptophyllium weswoodii* comb. nov. male, Thailand, Loei Province, Na Haeo, RBINS-PHYLLIUM DNA sample 0010 (RBINS).

**H** *Cryptophyllium westwoodii* comb. nov. female, Thailand, Fang, 16-211 (Coll RC).

**I***Cryptophyllium westwoodii* comb. nov. female, Thailand, Maetha, 16-080 (Coll RC).

**J***Cryptophyllium yunnanense* comb. nov. male, Vietnam, Nghia Lo, 17-240 (Coll RC).
